# A catalog of bacterial reference genomes from cultivated human oral bacteria

**DOI:** 10.1038/s41522-023-00414-3

**Published:** 2023-07-03

**Authors:** Wenxi Li, Hewei Liang, Xiaoqian Lin, Tongyuan Hu, Zhinan Wu, Wenxin He, Mengmeng Wang, Jiahao Zhang, Zhuye Jie, Xin Jin, Xun Xu, Jian Wang, Huanming Yang, Wenwei Zhang, Karsten Kristiansen, Liang Xiao, Yuanqiang Zou

**Affiliations:** 1 BGI-Shenzhen, 518083 Shenzhen, China; 2 School of Biology and Biological Engineering, South China University of Technology, 510006 Guangzhou, China; 3 College of Life Sciences, University of Chinese Academy of Sciences, 100049 Beijing, China; 4 Guangdong Provincial Key Laboratory of Genome Read and Write, BGI-Shenzhen, 518120 Shenzhen, China; 5 James D. Watson Institute of Genome Sciences, 310058 Hangzhou, China; 6 Laboratory of Genomics and Molecular Biomedicine, Department of Biology, University of Copenhagen, Universitetsparken 13, 2100 Copenhagen, Denmark; 7 Qingdao-Europe Advanced Institute for Life Sciences, BGI-Shenzhen, 266555 Qingdao, China; 8 PREDICT, Center for Molecular Prediction of Inflammatory Bowel Disease, Faculty of Medicine, Aalborg University, 2450 Copenhagen, Denmark; 9 Shenzhen Engineering Laboratory of Detection and Intervention of Human Intestinal Microbiome, BGI-Shenzhen, Shenzhen, China

**Keywords:** Bacteria, Microbial genetics

## Abstract

The oral cavity harbors highly diverse communities of microorganisms. However, the number of isolated species and high-quality genomes is limited. Here we present a Cultivated Oral Bacteria Genome Reference (COGR), comprising 1089 high-quality genomes based on large-scale aerobic and anaerobic cultivation of human oral bacteria isolated from dental plaques, tongue, and saliva. COGR covers five phyla and contains 195 species-level clusters of which 95 include 315 genomes representing species with no taxonomic annotation. The oral microbiota differs markedly between individuals, with 111 clusters being person-specific. Genes encoding CAZymes are abundant in the genomes of COGR. Members of the *Streptococcus* genus make up the largest proportion of COGR and many of these harbor entire pathways for quorum sensing important for biofilm formation. Several clusters containing unknown bacteria are enriched in individuals with rheumatoid arthritis, emphasizing the importance of culture-based isolation for characterizing and exploiting oral bacteria.

## Introduction

The human oral cavity, the gut, and the skin are major niches for colonization by symbiotic microorganisms. Collections of gut bacterial genomes have been published^1^, and evidence has accumulated that gut bacteria exhibit clear associations with several human diseases including inflammatory bowel disease^2^, type 2 diabetes^3^, colorectal cancer^4,5^, and cardiometabolic diseases^6^. Specific pathogenic bacteria may cause diseases, but common gut bacterial species may also contribute to the development or progression of diseases, and accordingly, probiotics have been considered for therapeutic interventions^7^.

The oral cavity is, next to the gut, the compartment harboring the highest abundance and diversity of microorganisms^8^, but the number of cultivated oral microbial isolates and genome collections is still limited. Specific bacteria have been associated with oral diseases including dental caries. *Streptococcus mutans*, able to form biofilms and release toxic factors, is widely considered as a caries-causing pathogen^9,10^. Many oral diseases are the result of a complex interactions between pathogenic microorganisms and the host^11^. A community named as the “red complex” including *Porphyromonas gingivalis*, *Treponema denticola* and *Tannerella forsythia* has been considered as a major peri-odontopathic pathogen^12^. Members of this community can release factors attacking periodontal tissues, and elicit intrinsic immune and inflammatory responses^13^. In addition to oral diseases, the oral microbiota has also been associated with systemic diseases such as type 2 diabetes (T2D)^14^, rheumatoid arthritis (RA)^15,16^, cardiovascular disease^17^ and Crohn’s disease (CD)^18^.

The expanded Human Oral Microbiome Database (eHOMD)^19^ is a large genome collection including 2123 bacterial genomes of which nearly half represents bacteria from the human oral cavity. A dataset comprising more than 50,000 metagenome-assembled genomes (MAGs) of the human oral microbiome was published in 2021^20^. Of note, 2313 out of 3589 species-level genome bins of these MAGs represented unknown species testifying to the need for further analysis of the oral microbiota.

Here we present the establishment of a collection of human oral bacteria isolates and genomes (termed the Cultivated Oral Bacteria Genome Reference (COGR)) containing 1089 high-quality reference genomes of cultivated oral bacteria. The genomes were clustered into 195 clusters of which 95 comprised 315 genomes representing unknown species. Combining these genomes and MAGs of oral bacteria, gene and protein catalogs were constructed. We predicted functions related to carbohydrate-active enzymes (CAZymes), biosynthetic gene clusters (BGCs), virulence genes, and quorum sensing in COGR. Our work provides a rich resource for the in-depth research of oral bacteria of potential clinical importance.

## Results

### The diversity of cultured human oral microbes

Due to the complex and diverse environments in the oral cavity^21^, oral microbes colonize many distinct microbial habitats. Some oral microbes adhere to the teeth and tongue while others reside in the saliva. Accordingly, we collected samples of saliva (ORS), from dental plaques (ODP), and from the tongue (ORT) (Supplementary Fig. 1a) of 13 healthy volunteers. About five thousand bacterial isolates were obtained using 34 different culture conditions (including aerobic and anaerobic conditions), and the DNA from ~1500 strains were selected for sequencing. One thousand and eighty-nine genomes were high quality with more than 95% completeness and less than 5% contamination evaluated by CheckM (Supplementary Table 1 and Supplementary Fig. 2), and these genomes were initially annotated according to the 16S rRNA gene sequences predicted from the whole genome.

Amongst the different culturing conditions, the highest numbers of isolates from one condition were obtained using blood-brain heart infusion (BHI) (aerobic) and MPYG (anaerobic) media (Supplementary Fig. 1b). The composition of the isolates differed according to culture conditions, reflecting the nutritional or environmental preferences of the bacterial species. Although the number of strains isolated using BHI (anaerobic) did not rank as the highest among the 34 different culture conditions, the genera collected using BHI (anaerobic) exhibited the highest diversity comprising in total 16 genera. Using MPYG (anaerobic), 14 genera were obtained, second only to BHI (anaerobic) (Supplementary Fig. 1c, d).

**Fig. 1:**
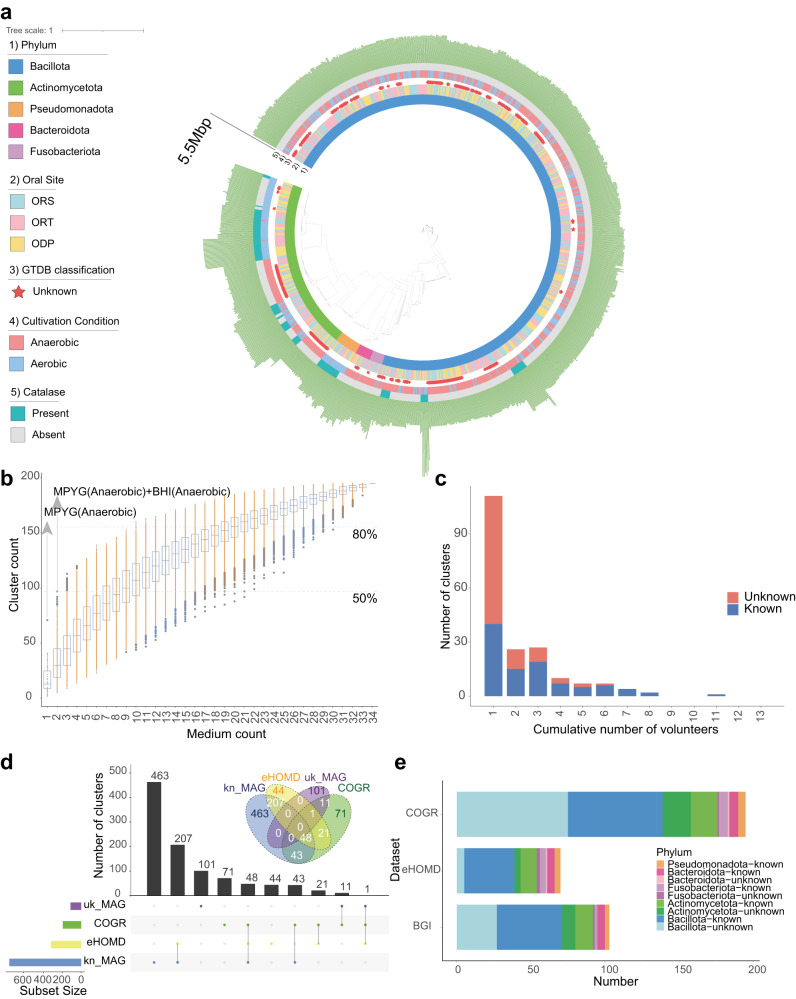
The genome profile of COGR. **a** Phylogenetic tree of 1089 COGR genomes based on GTDB annotation. The first circle is colored according to phyla, the second circle is colored according to the origin of the sample, the third circle highlights unknown genomes, the fourth circle is colored according to culture condition, the fifth circle is colored according to presence/absence of catalase, and the outermost circle represents genome length. **b** Rarefaction curve for the number of clusters obtained from different culture conditions. The MPYG (anaerobic) resulted in the highest count of clusters using one medium, the combination of MPYG (anaerobic) and BHI (anaerobic) resulted in the highest count of clusters using two media. The blue dash line marks the condition that provided 50% and 80% of the clusters of COGR. **c** The number of clusters shared by different numbers of volunteers. For example, when the cumulative number is 2, the ordinate indicates the number of clusters shared by two volunteers. **d** The upset plot and the Venn diagram of the comparison of different oral genome datasets. **e** Number of genomes of COGR mapped to the other two datasets.

To confirm the taxonomy of the isolated strains, we annotated their genomes using GTDB (Genome Taxonomy Database^22^, https://gtdb.ecogenomic.org/). Three hundred and fifteen genomes could not be classified into any known species representing potentially novel species. We noticed that most of the genera in our collection, except *Streptococcus*, had distinctly different preferences for oxygen (Supplementary Fig. 1f) and many strains belonging to unknown clusters were obtained using anaerobic conditions (Fig. 1a and Supplementary Fig. 1b), indicating that the oral cavity harbors a plethora of aerobic and anaerobic microbes, pointing to the importance of including anaerobic conditions for culturing oral bacteria. In addition, we noticed that the proportion of obtained bacteria species differed among different locations of the oral cavity, different media, and whether the medium included blood or not (Supplementary Fig. 1e, g, h).

### The establishment of the Cultivated Oral Bacteria Genome Reference, COGR

Based on the isolates, we were able to assemble 1089 high-quality genomes of oral microbes establishing the human Cultivated Oral Bacteria Genome Reference (COGR). The phyla in COGR included Bacillota (73.46%, 800 genomes), Actinomycetota (20.39%, 222 genomes), Pseudomonadota, Bacteroidota, and Fusobacteriota (Supplementary Table 2). Almost 58% of the genomes were annotated as *Streptococcus* (625 genomes), and 126 genomes were *Streptococcus salivarius*, a species which has been used as a commercial probiotic^23^. *Granulicatella* was the second most abundant genus in our collection (7.62%, 83 genomes). Mining the genetic information, we found that most genes encoding catalase were present in the strains of Actinomycetota and Pseudomonadota, isolated using aerobic conditions (Fig. 1a). With the criterion of 95% average nucleotide identity (ANI) as the threshold for distinction at the species level, the genomes were classified into 195 clusters, and 95 of these were without any known species annotations representing potentially novel species.

The cumulative curve illustrating the number of clusters using the 34 different conditions showed that 97 clusters, almost half of all clusters, could be cultured using a combination of BHI (anaerobic) and MPYG (anaerobic) conditions (Fig. 1b). However, an *α*-value of 0.617 also showed that saturation was not reached, emphasizing the importance of using a variety of culture condition for acquiring more oral microbial species. To explore the species diversity in different individuals, we assessed the cluster prevalence in the 13 volunteers. 111 clusters were obtained only from any one volunteer pointing to a highly personalized oral microbiota. Nearly 64% of these person-specific clusters were unknown clusters, indicating that massive culture-based isolation is necessary for discovering a comprehensive representation of oral microorganisms. One cluster, *Streptococcus salivarius*, was present in 11 out of 13 volunteers, pointing to its high prevalence in healthy individuals (Fig. 1c).

Strains isolated from the three different oral samplings could hardly be distinguished in the phylogenetic tree (Fig. 1a) and 41 clusters were shared between the three types of oral sampling (Supplementary Fig. 3a). In addition, principal co-ordinates analysis (PCoA) based on ANI or KEGG annotation profiles showed little differences among the three types of sampling. Despite a *P* value < 0.05, the variance (R^2^) was too low to clearly distinguish between genomes at the overall ANI level and KO level, and at the same levels for *Streptococcus* among the three types of samplings, reflecting that microbial diversity and functional diversity might be similar in different locations of the oral cavity (Supplementary Fig. 3b–f). However, we also observed differences, indicating that certain clusters preferred adhesion to tissues whereas this was not observed for others. Thus, the clusters of *Prevotella histicola*, *Rothia aeria*, *Actinomyces naeslundii*, *Rothia mucilaginosa*, *Neisseria sicca*, *Streptococcus intermedius*, and *Veillonella atypica* were found in ORT and ODP, but not in ORS, indicating that they may prefer solid surfaces. Still, ORS harbored the most diverse microbiome (Supplementary Fig. 3a).

We next compared the COGR genomes with the expanded Human Oral Microbiome Database (eHOMD)^24^, the largest public oral culturable microbiome dataset by far. Most genomes of eHOMD were from European individuals, and less than 36% (70/195) of the clusters in COGR isolated from Chinese individuals matched with eHOMD. To further explore the contribution of COGR, we mapped COGR genomes to 3589 metagenome-assembled genomes (MAGs) assembled from 4154 oral metagenomic samples^20^. 91 known species-level genome bins (kSGBs) and 12 unknown species-level genome bins (uSGBs) could be mapped to COGR (Fig. 1d). A comparison further revealed that COGR comprised 71 unique clusters and contributed several unknown clusters within the Bacillota and Actinomycetota phyla (Fig. 1e).

### A protein catalog of the human oral microbiome

**Fig. 2:**
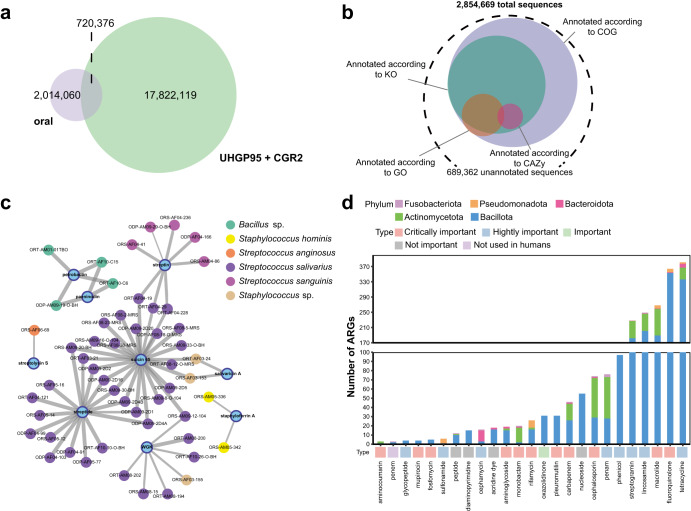
Functional profile of COGR. **a** Venn diagram of unique and shared protein sequences between oral and gut catalogs. **b** Annotation of 2,854,669 protein sequences according to COG, KO, GO and CAZy databases. **c** Sequence similarity network of our identified BGCs with known BGCs (similarity >70%,). The nodes in blue represent the known BGCs in the MiBIG database, and the remaining nodes represent identified BGCs in COGR, annotated with the genome name and colored by their species. The width of edges reflects degree of similarity. **d** Number of annotated ARGs and their distribution. The bottom square is colored according to the importance of drug usage.

Few studies have explored the overall functional diversity of the oral microbiota by constructing gene or protein catalogs^25^. To construct a human oral microbiome protein catalog, we combined protein-coding sequences (CDS) predicted from the genomes of COGR, eHOMD, and MAGs. After clustering and collecting representative CDSs based on 95% amino acid identity, we generated a non-redundant human oral microbiome protein catalog containing 2,854,669 CDSs (Supplementary Fig. 4a). COGR contributed 313,778 non-redundant CDSs, of which 106,729 were unique, representing CDSs identified by the culture-based approach using samples from Chinese individuals or CDSs of low abundance, difficult to detect by metagenomic methods. We found that 63.15% of these non-redundant CDSs were singletons (Supplementary Fig. 4b). Since the gut is a rich and intensely studied source of commensal microbes^26,27^, we compared the constructed human oral microbe protein catalog with the Unified Human Gastrointestinal Protein (UHGP) catalog and the protein sequences of the recent catalog of reference genomes of cultivated human gut bacteria (CGR2)^28^, which we grouped into 18,542,495 protein clusters at 95% protein identity (Fig. 2a). The result showed that oral microbes only shared 3.89% of the sequences with the gut microbes, but also that the oral microbes harbored 2,014,060 specific protein sequences not identified in the gut microbiome.

To investigate the functional profile of the oral microbiome, we annotated the protein sequences using eggNOG. The results showed that 75.71% (2,161,230), 44.20% (1,261,760), 8.41% (240,136), and 1.06% (30,388) of the sequences were annotated to the cluster of orthologous groups of protein (COGs), KEGG orthologous groups (KOs), gene ontology (GOs), and carbohydrate-active enzymes (CAZymes), respectively, while 25% lacked any annotation, representing genes of unknown function (Fig. 2b). The annotations based on MAGs, eHOMD, and COGR were similar (Supplementary Fig. 4c). In general, even though most of the sequences were annotated in the COG database, about 22.84% of the sequences were still annotated with unknown functions. Most proteins were involved in functions related to cell growth and development such as DNA replication, cell wall and membrane biogenesis, and metabolism of carbohydrates and amino acids. For carbohydrate metabolism, glycoside hydrolases (GHs) and glycoside transferases (GTs) were dominant, while COGR contributed the only one AA family (AA10) encoding a binding protein for chitin and cellulose catalyzing the cleavage of glycosidic bonds^29,30^, providing new insights into the initial digestion of dietary fibers by oral microorganisms.

### Functional characteristics of COGR

To illustrate the functional potential of the isolated oral bacteria, we performed an extensive functional exploration of the genomes of COGR. Regarding CAZyme gene prediction, CAZyme genes belonging to GH13, GT1, GT2, GT4, GT51 and CBM48 families were widely present in genomes of the COGR (Supplementary Fig. 5a). Compared to the expanded Culturable Genome Reference (CGR2)^28^, COGR included fewer types of CAZyme genes and families (Supplementary Fig. 5b). Among the CAZyme gene families, the proportion of GH13, GT4, CBM40 families in COGR and CGR2 was comparable. The GH13 family includes genes encoding α-amylase (CBM48 is appended to GH13 modules), while GT4 includes genes encoding sucrose synthase, pointing to the ability of the oral microbes to digest starch and sucrose.

Secondary metabolites produced by biosynthetic gene clusters (BGCs) have been recognized as major sources for discovery of novel drugs^31^. In addition, secondary metabolites also function as signaling molecules in microbe–microbe and microbe–host interactions^32^. Here, we performed an in-depth exploration of BGCs and identified a total of 2787 BGCs (33 types) from 996 genomes (Supplementary Table 3 and Supplementary Fig. 6a). The unspecified ribosomally synthesized and post-translationally modified peptides (RiPPs-like) were the most abundant BGC types, derived from Bacillota, Actinomycetota, and Pseudomonadota. RiPPs-like BGCs encode proteins involved in the generation of highly diverse natural products, including bacteriocins^33^. Previous studies^34^ have reported that aryl polyenes, which can increase protection against oxidative stress and contribute to biofilm formation, are abundant in the gingiva and on the tongue. In this study, we identified 108 aryl polyene BGCs in Bacillota, Bacteroidota, and Pseudomonadota isolated from tongue, dental plaques, and saliva, mainly from the genera *Streptococcus*, *Neisseria* and *Capnocytophaga*. We further identified BGCs encoding nine products with experimentally validated functions, two of which were present in potentially new species of *Bacillus*, whereas the remaining BGCs were present in various members of the genus *Streptococcus* (Fig. 2c). Streptolysin S, originally produced by *S. pyogenes*, is a potent cytolytic toxin and virulence factor, and we found that the potential pathogen *S. anginosus*^35^ also had the ability to encode streptolysin S. Suicin 65 and salivaricin A, produced by members of *S. salivarius* and potentially new species, are bacteriocins that are active against *S. suis*^36^ and *S. pyogenes*^37^, respectively. This result revealed the potential of oral microbes for production of bio-active small molecules.

We identified 108 antibiotic resistance genes (ARGs) conferring resistance to 25 drugs in the oral microbes, of which 31 were multi-drug resistant. Most of the drugs were listed by WHO as extremely important for human use^38^, such as tetracyclines, fluoroquinolones, and macrolides, which can be used as orally administrated antibiotics. The ARGs were widely distributed in five phyla (Fig. 2d). Most ARGs were identified in Bacillota, and more than 50% of the genes conferring resistance to penams, cephalosporins, monobactams, and aminocoumarins were identified in Actinomycetota, 75% of the genes conferring resistance to cephamycins were identified in Bacteroidota, and 83.33% of genes conferring resistance to sulfonamides were identified in Pseudomonadota.

We identified 12 types of virulence factors (VFs) in 17 genera (Supplementary Fig. 6b). *Enterococcus* contained the highest abundance of VFs, and all members of this genus had at least one VF. Here, we found that *S. anginosus* strain ORS-AF06-69 had the potential to encode streptolysin S, an exotoxin involved in infection.

### Quorum sensing of oral bacteria in COGR

**Fig. 3:**
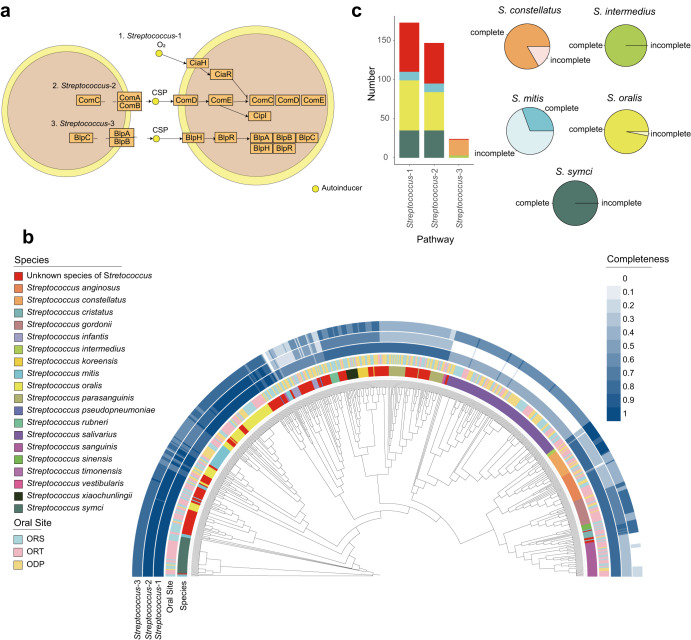
Quorum sensing in Streptococcus. **a** Schematic overview of quorum sensing pathways in Streptococcus (KEGG map02024 (https://www.genome.jp/pathway/map02024)). Genes are represented as orange boxes and the small yellow circles represent autoinducers. Two cells are depicted. **b** Phylogenetic tree of *Streptococcus* strains in COGR. The innermost circle is colored according to species and the second circle is colored by according to the oral sampling site. The outer three circles are colored according to the completeness of three quorum sensing pathways in *Streptococcus*. **c** The bar plot on the left shows the number of species harboring the complete quorum sensing pathway. The pie chart on the right shows the proportion of complete and incomplete coverage of the quorum sensing pathway in the indicated species of COGR. The color code in (**c**) is the same as that used in (**b**).

Bacterial quorum sensing is a communication system, within and between different cells, regulating gene expressions in response to population cell density^39^. Quorum sensing is also involved in functions such as bioluminescence^40^, bacteriocins production^41^, and importantly, biofilm formation^42^. Thus, the caries-inducing bacterium *Streptococcus mutans* can form biofilms and release virulence factors^9,10^. Quorum sensing plays an important role in colonization and survival of *Streptococcus*. Since we obtained 625 genomes of *Streptococcus*, we decided to perform an extensive analysis on the quorum sensing function in the orally residing *Streptococci*. We therefore mapped genes from the genomes in COGR to the quorum sensing pathway (KEGG map02024, https://www.genome.jp/pathway/map02024) (Fig. 3a). 197 strains from 38 clusters in COGR harboring the three pathways of quorum sensing were all from the *Streptococcus* genus (referred to as *Streptococcus*-1, *Streptococcus*-2, *Streptococcus*-3) (Supplementary Table 4a). We noticed that species harboring genes involved in quorum sensing pathways did not exhibit specific associations with the three oral sites investigated (Fig. 3b). Most strains of *Streptococcus* exhibited at least 50% coverage of the *Streptococcus*-3 pathway and many of the unknown strains in COGR harbored all three pathways. The species harboring the three pathways are presented in Fig. 3c, showing that the distribution of *Streptococcus*-1 was similar to *Streptococcus*-2 while the distribution of *Streptococcus*-3 differed. Among the three pathways, *Streptococcus*-1 was covered by most strains (174/197 strains). Apart from *Streptococcus mitis*, most of the strains of *Streptococcus symci*, *Streptococcus oralis*, *Streptococcus constellatus*, and *Streptococcus intermedius* harbor genes covering the three pathways, reflecting the ability of these species for quorum sensing.

In the pathways *Streptococcus*-1 and *Streptococcus*-2, *comD* and *comE*, the two-component signal transduction system, enable *Streptococcus* to form biofilm^43^. In addition, the ComDE and the CiaRH systems contribute to acid tolerance to resist environmental stress^44^. In the pathway *Streptococcus*-3, the *blp* locus is responsible for the production of bacteriocins and proteins involved in immune responses, limiting the growth of other sensitive microorganisms and protecting themselves from their own bacteriocins^45,46^.

To examine the importance of the quorum sensing pathways for biofilm formation, we selected several strains that harbored or did not harbor the complete quorum sensing pathways and tested their ability to form biofilms using the crystal violet assay^47^ (Supplementary Table 4b and Supplementary Fig. 7). Using *S. mitis*_ORS-AM05-478 which does not harbor the complete set of genes involved in the pathway of quorum sensing as a reference, we observed significant biofilm formation for one strain of *S. constellatus* and one strain of *S. oralis*, both harboring genes encoding the entire pathway of quorum sensing. Notably, we found that three strains of *S. salivarius* lacking genes in the three pathways of quorum sensing also efficiently formed biofilms, suggesting the existence of quorum sensing-independent pathways for biofilm formation in these strains. Thus, it has been reported that BglB, CshA, Asp1, GtfG, SecA2, and other associated proteins present in *S. salivariius* may contribute to bacterial auto-aggregation and adhesion to host cells^48^. Finally, it is noteworthy that *S. salivarius* can inhibit the aggregation and biofilm formation of specific pathogens^49,50^, which suggests that *S. salivarius* may play an important role in the human oral cavity, and that further studies on quorum sensing and biofilm formation are warranted.

### Distribution of oral species in the human population

**Fig. 4:**
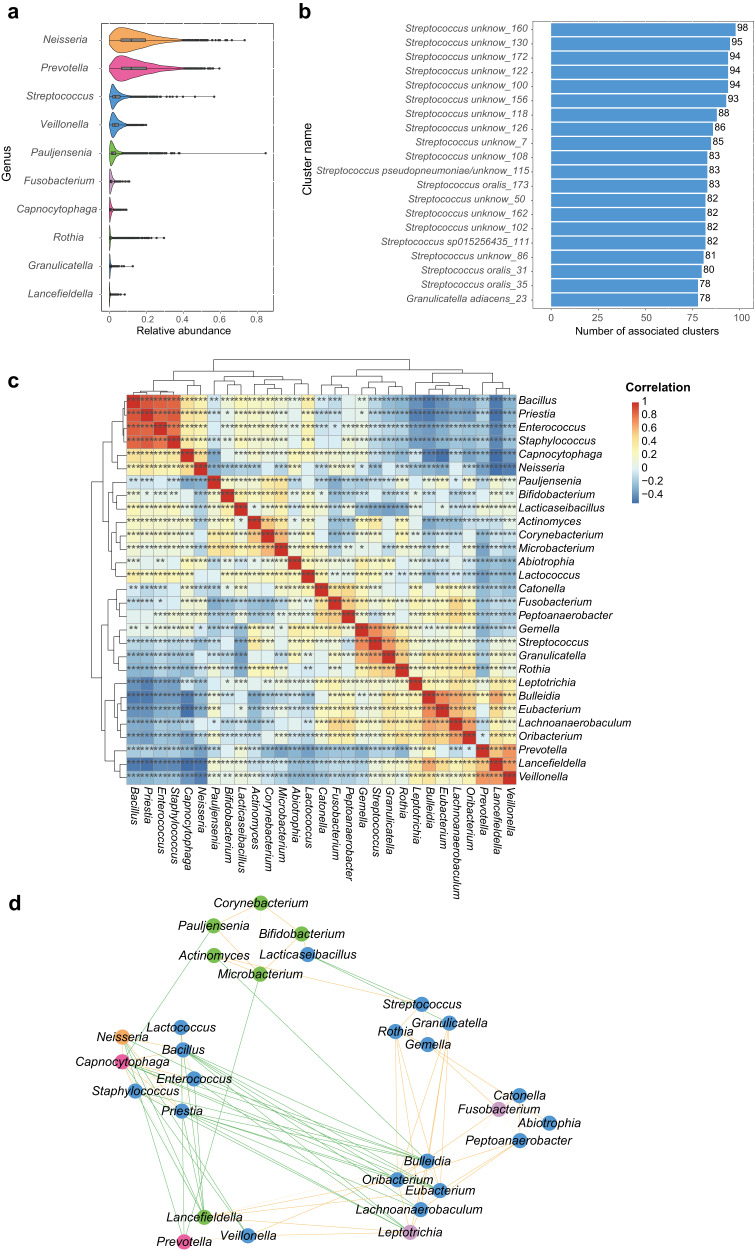
Mapping of 195 representative strain genomes of each cluster from COGR to 4362 oral metagenomes. **a** Genera with relative abundance ranking in top 10 in 4362 metagenomes, colored by phylum. **b** The top 20 clusters with the highest number of associations to other clusters in COGR in a co-occurrence analysis between the 195 clusters. The clusters are named as “GTDB species_cluster number.” **c** Co-occurrence heatmap of 29 genera based on the relative abundances in the metagenomes. Red color represents positive relationships while blue represents negative relationships. The stars marked in the boxes represent significance. **d** Network of 29 genera based on the correlation analysis (*r* > 0.3). The nodes are colored by phylum. Positive correlations are shown by orange lines and negative correlations by green lines. The width of the lines reflects the strength of the correlation. The phyla color codes are as in Fig. 1.

In order to explore the distribution of members of COGR in the oral microbiota of humans, we mapped 195 representative genomes of each cluster of COGR to 3971 salivary metagenomes and 391 tongue metagenomes^20^. The clusters in COGR covered 2.20–91.21% of the species abundance in the 4362 oral samples and the unknown species comprised a median of 10.57% of the abundance per metagenomic sample. *Neisseria* exhibited the highest relative abundance (11.93%) of the COGR genomes mapped to the 4,362 metagenomes, followed by *Prevotella* (11.90%) and *Streptococcus* (3.26%) (Fig. 4a). Although *Streptococcus* made up the largest culture proportion in COGR, its relative abundance ranked third in the genera profile. *Rothia, Granulicatella*, *Actinomyces*, and *Microbacterium* were low abundant genera in the metagenomes, but these four genera were readily cultured in COGR. This indicated that culture-based approaches might enable the acquisition of genera with low relative abundance in the oral cavity.

We conducted a bacteria co-occurrence analysis among the clusters in COGR based on their relative abundance in the 4362 metagenomes and found that 15 of the top 20 clusters with the most associations with others in COGR were unknown clusters (Fig. 4b and Supplementary Table 5). We also conducted a co-occurrence analysis and a correlation network analysis among 29 genera in COGR. According to the heatmap and network, the genera could be clustered into six groups, of which clusters within the same group were positively associated. Even though some genera were from different phyla, they clustered together. The group harboring *Neisseria* exhibited a pronounced negative correlation with other groups, indicating that the genera in this group might communicate closely with each other and form a stable group (Fig. 4c, d). We envisage that our work demonstrating specific correlations between oral species will serve as a resource for further studies.

### Associations between species of COGR and rheumatoid arthritis

**Fig. 5:**
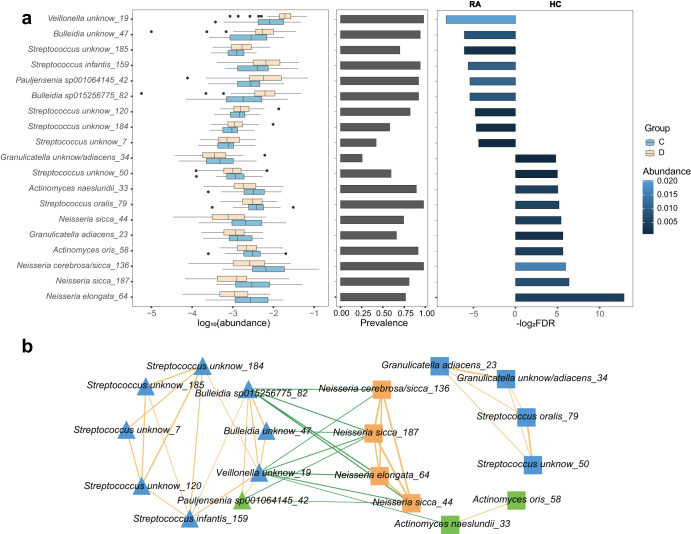
Differential patterns of clusters of oral microbes in 47 healthy controls (HC) and 50 patients with rheumatoid arthritis (RA). **a** The logarithm of abundance (base 10) in each group and the prevalence of differential clusters. The percentage of samples with abundance of clusters higher than 0.1% was considered as the prevalence. The logarithm of FDR (base 2) between RA and HC is presented, colored according to the average abundance in corresponding group. **b** Correlation network of clusters differing in abundance between HC and RA, with nodes colored according to phylum. Square nodes are clusters enriched in HC, while triangle nodes are clusters enriched in RA. Positive correlations are indicated by orange lines and negative correlations by green lines. The width of the lines indicates strength of the correlation.

Previous studies have reported on specific difference between the oral microbiome of healthy human individuals and patients with rheumatoid arthritis (RA)^15^. In order to study the association of the genomes in COGR with RA, 47 metagenomes of healthy control and 50 metagenomes of patients with RA were downloaded from a public database^15^ and mapped to 195 representative genomes of COGR. Based on the abundance profiles, 9 clusters were significantly enriched in the disease group (RA), while 10 clusters were significantly enriched in healthy controls (HC), not considering clusters whose prevalence was zero (eBayes, adjusted *P* value < 0.05) (Fig. 5a). The most significantly enriched clusters in the oral microbiome of HC were from *Neisseria*, while the most significantly enriched cluster in RA patients was from *Veillonella*, consistent with previous studies^15,51^. Two clusters of *Bulleidia* in COGR, both unknown species, were significantly enriched in RA patients. Notably, many of the clusters enriched in the RA group were unknown species (8/9 clusters), emphasizing the value of the culture-based approach.

The correlation network based on the abundance of each cluster in the 97 metagenomes showed that the clusters enriched in HC and the clusters enriched in RA patients were positively associated with each other in the same group and negatively associated with clusters in the other group (Fig. 5b). The correlations between these clusters not only differed significantly between healthy and diseased individuals, but also exhibited close associations with other clusters, suggesting that they might play a role in the pathogenesis of RA and might serve as biomarkers for RA.

### Comparison between COGR and CGR2

**Fig. 6:**
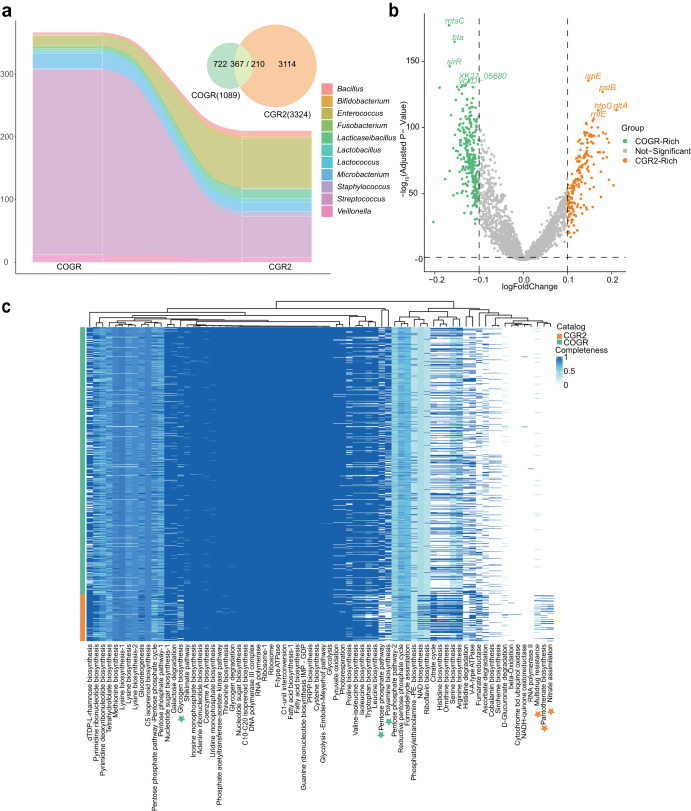
Comparison between CGR2 and COGR. **a** Genome-wide comparison of COGR (oral) and CGR2 (gut). The number of matched genomes is shown at the genus level using a Sankey diagram. 367 genomes of COGR match 210 genomes of CGR2. **b** Differential proteins encoded by COGR and CGR2. The top 5 -log_10_ (Adjusted p-value) proteins are marked. **c** KEGG module completeness heatmap of *Streptococcus*. The modules exhibiting significant differences in COGR or CGR2 are highlighted by stars in green or orange.

To get insight into species characterizing COGR and CGR2, and providing information on the ability of oral bacteria to colonize the gut, we compared the microbiomes of COGR and CGR2. All 15 annotated orders in COGR were present in CGR2, and 367 COGR genomes matched 210 CGR2 genomes by an ANI ≧ 95% (Fig. 6a). 11 of 29 genera in COGR matched CGR2. 16 genomes in COGR of *Enterococcus*, a widespread genus in human niches, matched 79 genomes in CGR2. 295/625 genomes of *Streptococcus*, the most abundant genus in COGR, matched 65 genomes in CGR2 (Supplementary Fig. 8a). Many species including *Streptococcus oralis*, *Streptococcus anginosus* were abundant in COGR but were not included in CGR2, and a species such as *Streptococcus macedonicus* was not found in COGR. Of note, all 25 COGR genomes of *Microbacterium* were assigned to *Microbacterium algeriense*, and they matched the genomes of *Microbacterium algeriense* in CGR2 with an ANI higher than 99.9% suggesting a possible transmission from the oral cavity to the gut of this bacterium (Supplementary Fig. 8b).

To get further insight into the differences between species isolated from the oral cavity and the gut, we focused on proteins encoded by genomes of both collections. The differential proteins analysis revealed that 1706 types of proteins were enriched in COGR and 3955 types of proteins were enriched in CGR2 (Fig. 6b). For the proteins encoded by *Streptococcus*, the analysis showed that *N*-acetylmuramoyl-L-alanine amidase, *amiC, amiD*, and *amiF*, were significant enriched in COGR (Supplementary Fig. 8c). To investigate the protein difference reflected in functional units, we computed the KEGG modules completeness of *Streptococcus* genomes in COGR and CGR2 (Fig. 6c). *Streptococcus* exhibited specific functional changes to adapt to different habitats. Protein encoded by *Streptococcus* in COGR had high completeness in module M00006, which is responsible for the oxidative phase in pentose phosphate pathway. By contrast, only bacteria in CGR2 harbored complete modules of M00119 and M00615, which are responsible for pantothenate biosynthesis and nitrate assimilation, respectively. M00705, a module of the efflux pump MepA related to multidrug resistance was more prevalent in CGR2 than in COGR.

## Discussion

Similar to gut-residing microorganisms, a large number of oral microorganisms are closely related to human health, but in-depth studies and culturing of oral microbes are still limited. The COGR substantially increases the number of cultivated bacterial species with high quality genomes from three location of the oral cavity. Thus, COGR comprises 1089 cultivated bacteria isolated by using 34 culture conditions. Of the 195 species-level clusters included in COGR, 95 include 315 genomes of species with no taxonomic annotation. The large-scale culturing approach resulted not only in the isolation of the more abundant species present in the oral cavity, including member of the *Streptococcus* genus, but also several low-abundant species from the genera *Pauljensenia*, *Rothia*, *Granulicatella* and *Actinomyces*, demonstrating the value of large-scale culture-based approaches for characterizing the oral microbiome. Our analyses also demonstrated remarkable differences between the oral microbiome of the 13 volunteers with 111 clusters exhibiting person-specific distribution.

We constructed a protein catalog with more than 2.8 M sequences from 5716 oral microbial genomes, and interestingly, 47.84% of the proteins are without functional annotation, further pointing to the importance of culture-based characterization for elucidating the functional potential also for the oral microbiota.

Genes encoding CAZymes are abundant in the genomes of COGR, and in addition, more than 2000 BGCs were identified in COGR, pointing to the potential of oral microbes for production of bio-active small molecules. Bacterial quorum sensing is important for establishment and survival in different niches^39^. We found that 197 strains of 38 clusters from *Streptococcus* harbored the three pathways of quorum sensing. Thus, in vitro experiment confirmed the ability of *S. constellatus* and *S. oralis*, both of which harbor the complete quorum sensing pathways. Of note, our biofilm formation experiment also showed that the strains of *S. salivarius*, which do not harbor complete pathways of quorum sensing were efficient biofilm formers showing that effective biofilm formation may occur independently of quorum sensing.

The culture-based approach also proved of value in relation to using the oral microbiota for clinical purposes. We have previously, reported that the oral microbiota differs between healthy individuals and individuals suffering from RA^15^. We found that four clusters from *Neisseria* were significantly enriched in healthy individuals, while 8 unknown clusters were enriched in the RA group, suggesting that these clusters might be related to RA and potentially used for diagnosing or even treating RA.

In conclusion, we envisage that COGR will serve as a valuable and useful resource for future exploitation of the potential for the isolation of novel bio-actives as well as clinical treatment of not only oral diseases but also other systemic diseases.

## Methods

### Sample collection and culturing

Thirty-nine oral samples were collected from 13 healthy volunteers not taking any antibiotics in the last six months prior to sampling or suffering from oral diseases such as aphthous ulcerations and caries. The volunteers were instructed not to brush teeth, drink alcohol, or eat spicy food within 12 h prior to sample collection. Sample collection: ORT, a sterile cotton swab was rolled several times on the tongue and the tip was placed in sterile PBS. ODP, the buccal plaque of the premolars was swabbed with a sterile swab and the tip was placed in sterile PBS. ORS, 2–5 ml of saliva were collected in a sterile tube (Supplementary Fig. 1a). Plates were incubated using 34 different culturing conditions for 2–3 days (Supplementary Table 6) and single colonies were picked and streaked onto new plates to obtain single strains. All the strains were stored in a glycerol suspension (20%, v/v) at −80 °C.

### Genome sequencing, assembly, quality assessment

The methods of whole-genome sequencing and de novo assembly were as described by Zou et al.^1^. Genome quality was evaluated by CheckM (v1.1.2)^52^, and genomes with >95% completeness and <5% contamination were selected as high-quality genomes.

### Phylogenetic and taxonomic analyses

16S rRNA gene sequences were predicted from the whole genome using RNAmmer (v1.2)^53^, and the predictions were annotated using EzBioCloud’s 16S database^54^ with MOTHUR(v1.45.3)^55^. GTDB-TK (v1.5.0)^22^ with database release207^22^ was used to perform taxonomic annotation of each genome and construct the maximum-likelihood phylogenetic tree based on 120 conserved single-copy genes. The pairwise alignment ANI was calculated using fastANI (v1.32)^56^, and hclust from the R package was used to cluster at the proposed cutoff species level (ANI ≥ 95%). The phylogenetic trees were visualized by iTOL (v6.5.6, https://itol.embl.de/). Multi-lineage taxonomy was not considered in this context.

### Alignment with other genome collections

We downloaded 3324 gut bacterial genomes from the Culturable Genome Reference V2 (CGR2)^28^, 3589 species-level genome bins (SGBs) from an oral metagenomically assembled draft genomes dataset^20^, and 1089 oral cavity genomes from the expanded Human Oral Microbiome Database V3^19^. All the downloaded genomes were quality evaluated by CheckM, and selected with >95% completeness and <5% contamination. The genome alignment was executed by fastANI (v1.32), and the pair alignment with ANI ≥ 95% was identified as a species-level match.

### Protein catalog construction and functional annotation

Protein-coding sequences (CDS) of each genome were predicted and annotated with Prokka (v1.14.6)^57^. The protein catalog of the human oral microbiome was generated by integrating all predicted CDSs derived from 1089 COGR genomes, 1089 eHOMD genomes, and 3589 MAGs^20^. The “linclust” function of MMseqs2^58^ (Version 13.45111) was used to construct a non-redundant protein catalog, with options “-ov-mode 1 -c 0.8 -kmer-per-seq 80 -min-seq-id 0.95.” This tool was additionally used to cluster the human oral protein catalog with UHGP-95^27^ and CGR2, representing the human gut genomic protein catalog.

The preliminary functional annotation was carried out by eggNOG-mapper v2^59^ (eggNOG database version: 5.0.2^60^). The COGs, KOs, GOs, and CAZymes were extracted from the eggNOG-mapper result, and counted by functional categories. CAZymes were annotated with dbCAN (v2.0)^61^.

### Identification of BGCs

A total of 2787 BGCs were explored by antiSMASH 6.0^33^, one of the most widely used tools for the detection and characterization of BGCs in bacteria. The predicted BGCs were mapped against the MiBIG database^62^ to characterize BGCs with >70% identity as known functions. The relationship between SMBGs with known functions and cognate genome regions was displayed by Cytoscape (v3.8.2)^63^.

### Annotation of ARGs and VFs

The “*main*” feature with default parameter of Resistance Gene Identifier (RGI) version 5.2.0 and the Comprehensive Antibiotic Resistance Database (CARD^64^, v3.1.2) was used to annotate ARGs. The VFs annotation of all CDS was performed by BLAST v2.2.26 (−evalue 0.01) against the Virulence Factor Database (VFDB^65^, setB, 2021-07) with identity higher than 60% and coverage higher than 50%.

### Crystal violet staining for determination of biofilm formation

The crystal violet assay was performed as described by O'Toole^47^. Selected strains were cultured overnight in brain heart infusion (BHI) medium at 37 °C. After diluting 1:10 into fresh medium, 100 μl dilutions were added into a 96-well plate and incubated overnight at 37 °C. Four replicates for each strain. BHI medium was used for control. The culture medium was removed and the wells were washed by 125 μl PBS 1–2 times. After drying for about half an hour, 125 μl of a 0.1% solution of crystal violet were added to each well, and incubation was continued for 15–20 min. Liquid was removed and the wells were washed with 125 μl double distilled water 1–2 times. The plates were dried for a few hours or overnight. 125 μl of 30% acetic acid in water were added to each well and the liquid was transferred to a new plate for measuring the OD values using a microplate reader at 595 nm.

### Calculation of the relative abundance of COGR clusters in metagenomes

For investigating the distribution of oral species in a larger human population, 3691 metagenomics samples were acquired from the 4D-SZ cohort^20^ (CNP0000687, https://db.cngb.org/search/project/CNP0000687/) and 671 metagenomics samples were acquired from CNP0001221 in the CNGB database (https://db.cngb.org/search/project/CNP0001221/). Forty-seven oral metagenomes from healthy control individuals and 50 oral metagenomes from individuals with RA^15^ are all public data, downloaded from https://www.ebi.ac.uk/ena/browser/view/PRJEB6997. They were acquired for analyzing the associations of members of the COGR with human diseases. Fastp (v0.23.1)^66^ was used to filter out low quality reads and bases with partial parameters “-qualified_quality_phred 15 -complexity_threshold 30 -length_required 30.” Bowtie (v2.4.4)^67^ was used to remove host contamination by mapping reads to the human genome (GRCh38). In order to calculate the abundances of COGR clusters across the samples, we selected the genomes with the longest genome sequence of each cluster in the COGR, under the premise of the highest completeness, as the representative genomes of COGR. A representative genome regarded as a bacterial genome reference of Kraken2^68^ (v2.1.2) database and Kraken2 combined with Bracken (v2.6.2)^69^ was used to estimate the abundances of representative genomes. Relative abundances were calculated and samples or genomes without any reads mapped were filtered out using the R software.

### Statistical analysis

Statistical tests were performed using R v4.1.2. The package micropan was used for plotting the rarefaction curve and calculating the α-value. For the PCoA, Bray–Curtis dissimilarities were calculated by the vegdist function. The confidence interval was 95%. For detecting differentially abundant species and genes, the packages edgeR and limma were used for the differential analysis and the FDR were calculated by Empirical Bayes Statistics (two-sided). The R function “corr.test” was used to calculate the correlation coefficient for bacteria co-occurrence analysis, and subsequently, Cytoscape (v3.9.1)^63^ was used for data visualization in a network. The package ggplot2 for R was used for plotting. Adobe Illustrator CC 2018 was used to adjust colors and construct figures.

### Reporting summary

Further information on research design is available in the Nature Research Reporting Summary linked to this article.

## Supplementary information

Supplementary information

Reporting Summary

## Data Availability

The data that support the findings of this study have been deposited into CNGB Sequence Archive (CNSA)^70^ of China National GeneBank DataBase (CNGBdb)^71^ with accession number CNP0003047 (https://db.cngb.org/search/project/CNP0003047/, 10.26036/CNP0003047). All the bacterial strains in COGR have been deposited in China National GeneBank (CNGB), a non-profit, public-service-oriented organization in China. All relevant data are available from the authors.
