# Supplementary material for: A catalog of bacterial reference genomes from cultivated human oral bacteria
Source: NPJ Biofilms Microbiomes. 2023 Jul 3;9:45. doi: 10.1038/s41522-023-00414-3 (PMC10318035; doi:10.1038/s41522-023-00414-3)
Supplement: Supplementary file 1 — Supplementary information [file 41522_2023_414_MOESM1_ESM.pdf]

Supplementary Figures:

A collection of bacterial genomes from cultivated human oral bacteria

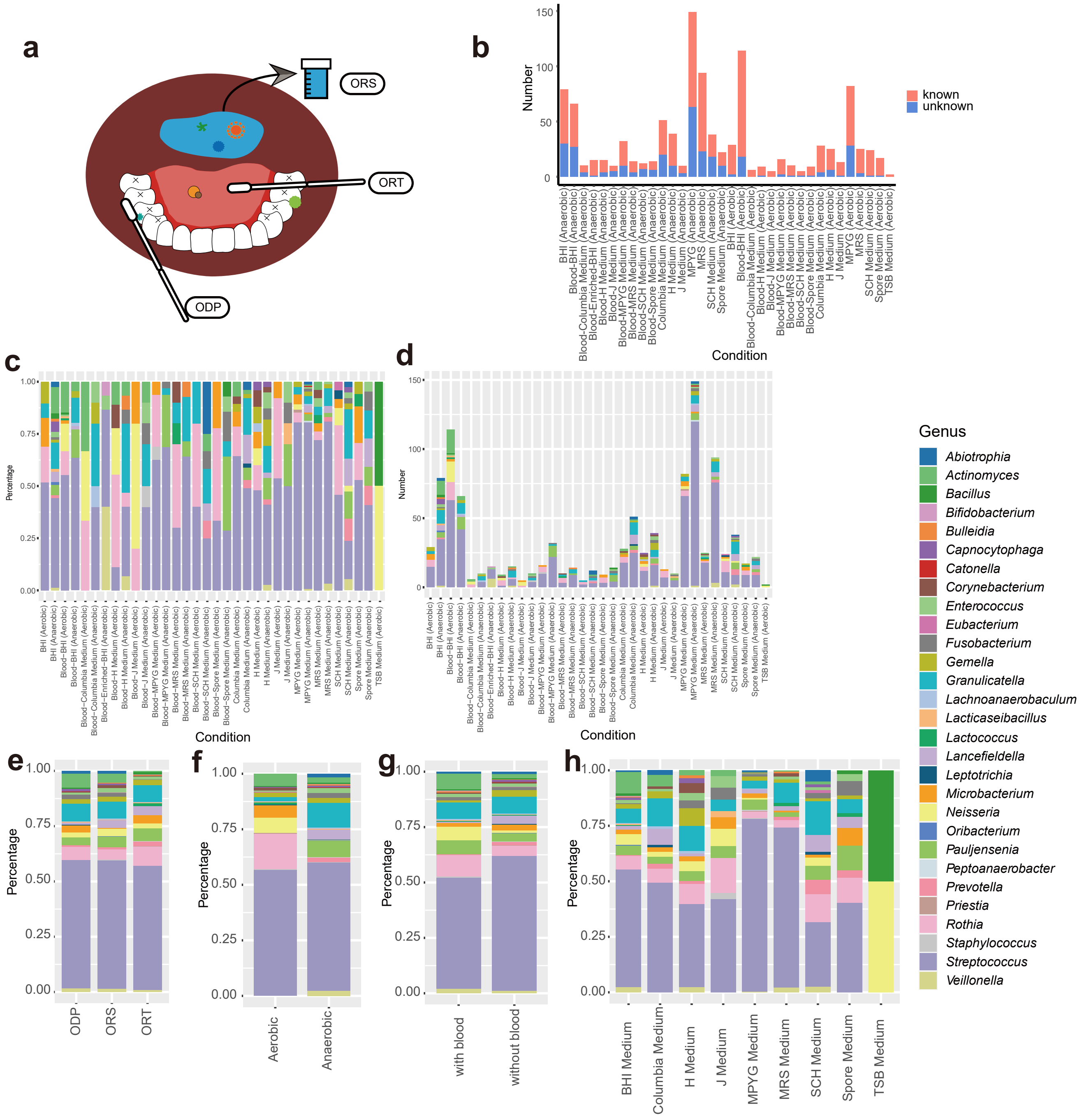

Supplementary Fig. 1 | Cultured diversity of the oral microbiota. a, Microbiota samples were collected from three different oral location of each volunteer, namely, saliva (ORS), oral dental plaque (ODP), tongue (ORT). **b**, Number of known and unknown strains cultured from 34 culture conditions. Left half represent anaerobic conditions. **c &d**, percentage (**c**) and number (**d**) of each genus using the 34 conditions.

Percentage of oral strains of each genus was also calculated by samplings (**e**), aerobic or anaerobic conditions (**f**), conditions with or without blood (**g**) and different media (**h**).

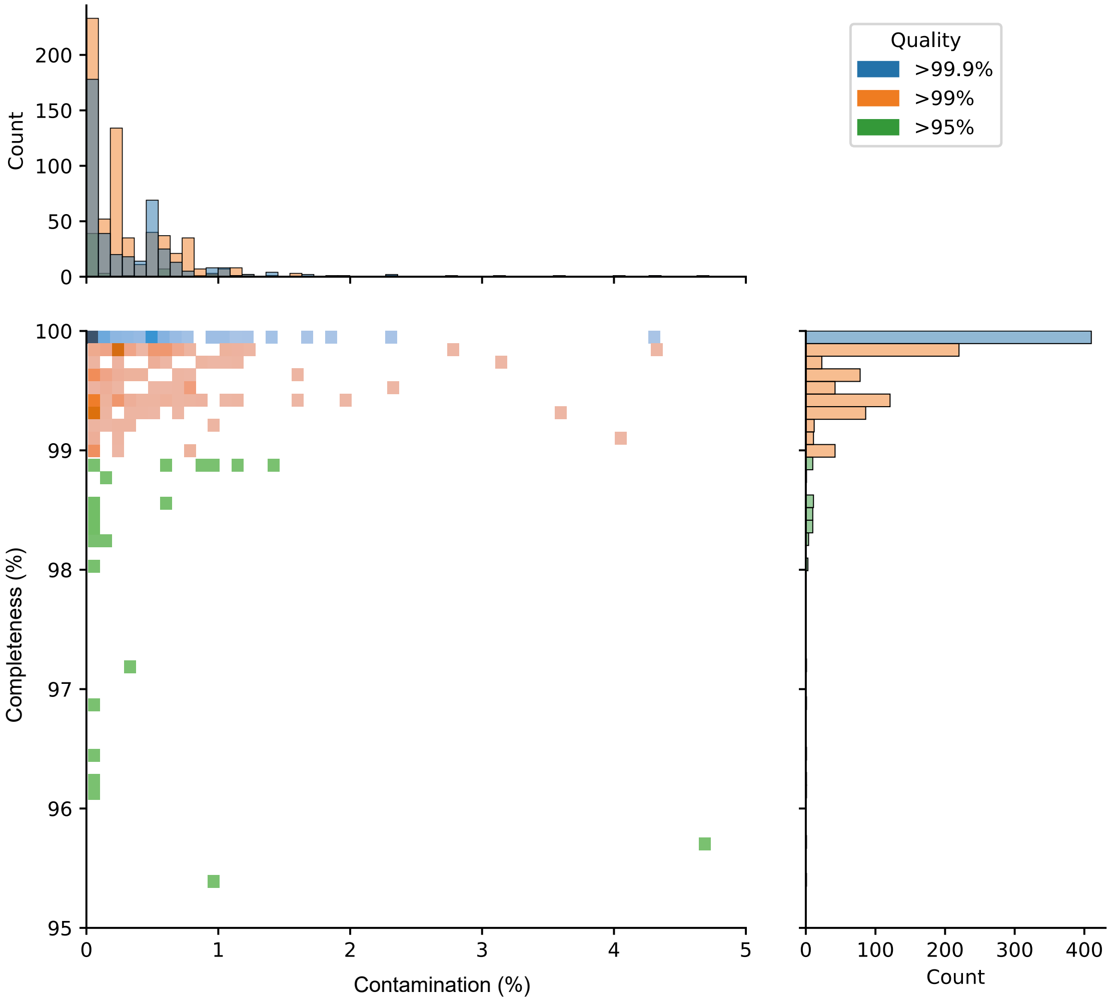

Supplementary Fig. 2 | Assessment of 1,089 COGR genomes using CheckM. The colors mark the different levels of completeness, and were deepened by the count of genomes. The height of marginal distribution denotes the counts of different completeness or contamination level.

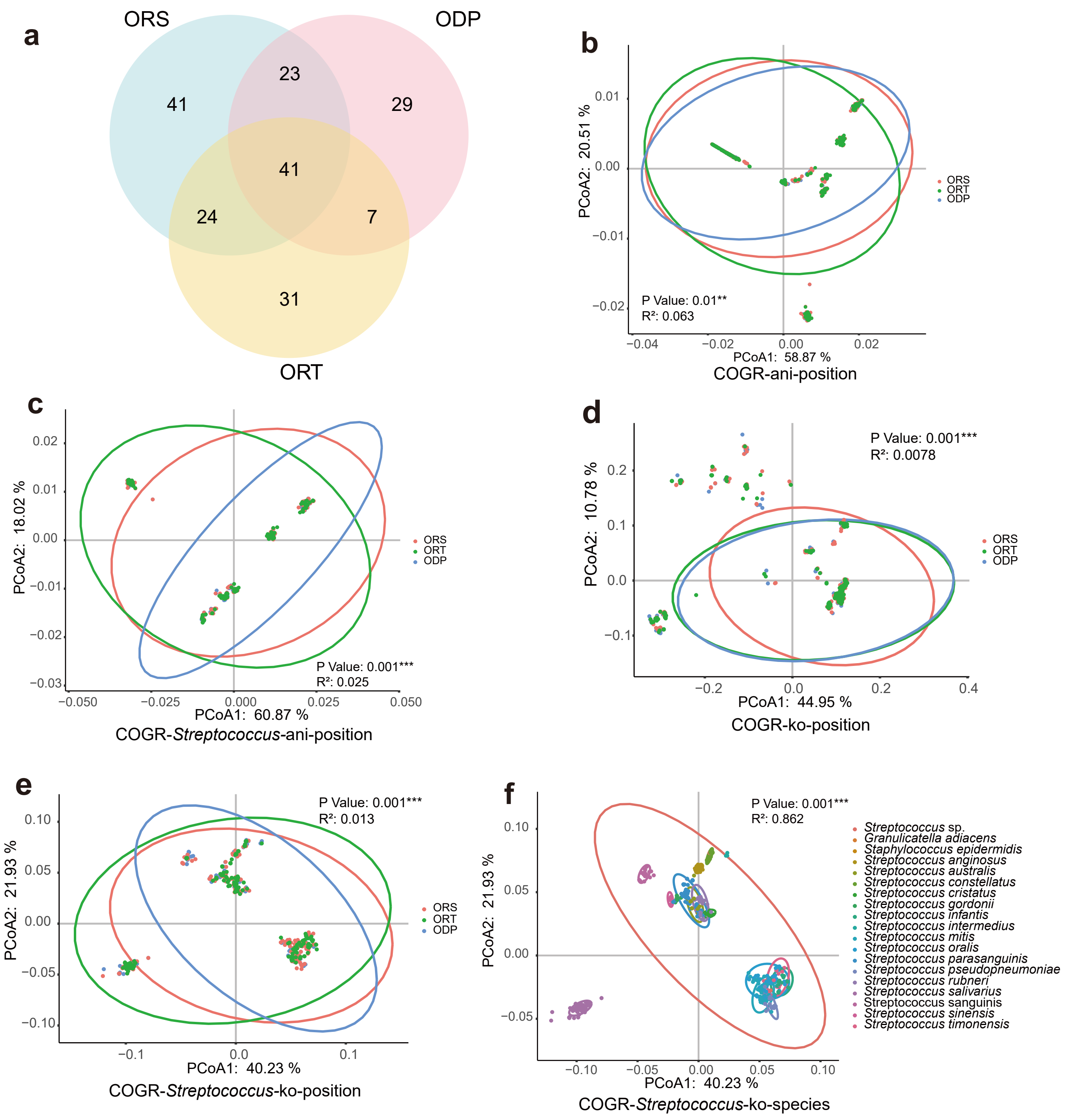

Supplementary Fig. 3 | Differences between microbial communities of the three oral locations. a, Venn diagram of clusters isolated from three different samplings. **b-f**, Principal Coordinates Analysis (PCoA) of taxonomic (**b**, **c**) and functional (**d**, **e**) profiles among microbial communities from different sites using Bray-Curtis dissimilarity calculated based on ANI and KO numbers annotated by KEGG. **b & d** present PCoA among all genomes in COGR while **c & e** present PCoA among genomes of *Streptococcus*. P-value and R^2 were calculated based on adonis test and are presented in each plot. Although the p-value showed significant differences between microbial

communities of three oral positions, the R^2 were so low that we could not make a definite conclusion. PCoA among genomes of different species (**f**) showed that significant and reliable differences exist, indicating that the results above may be caused by the distinct difference between the taxonomy of different species. All genomes of novel strains are grouped together (*Streptococcus* sp.).

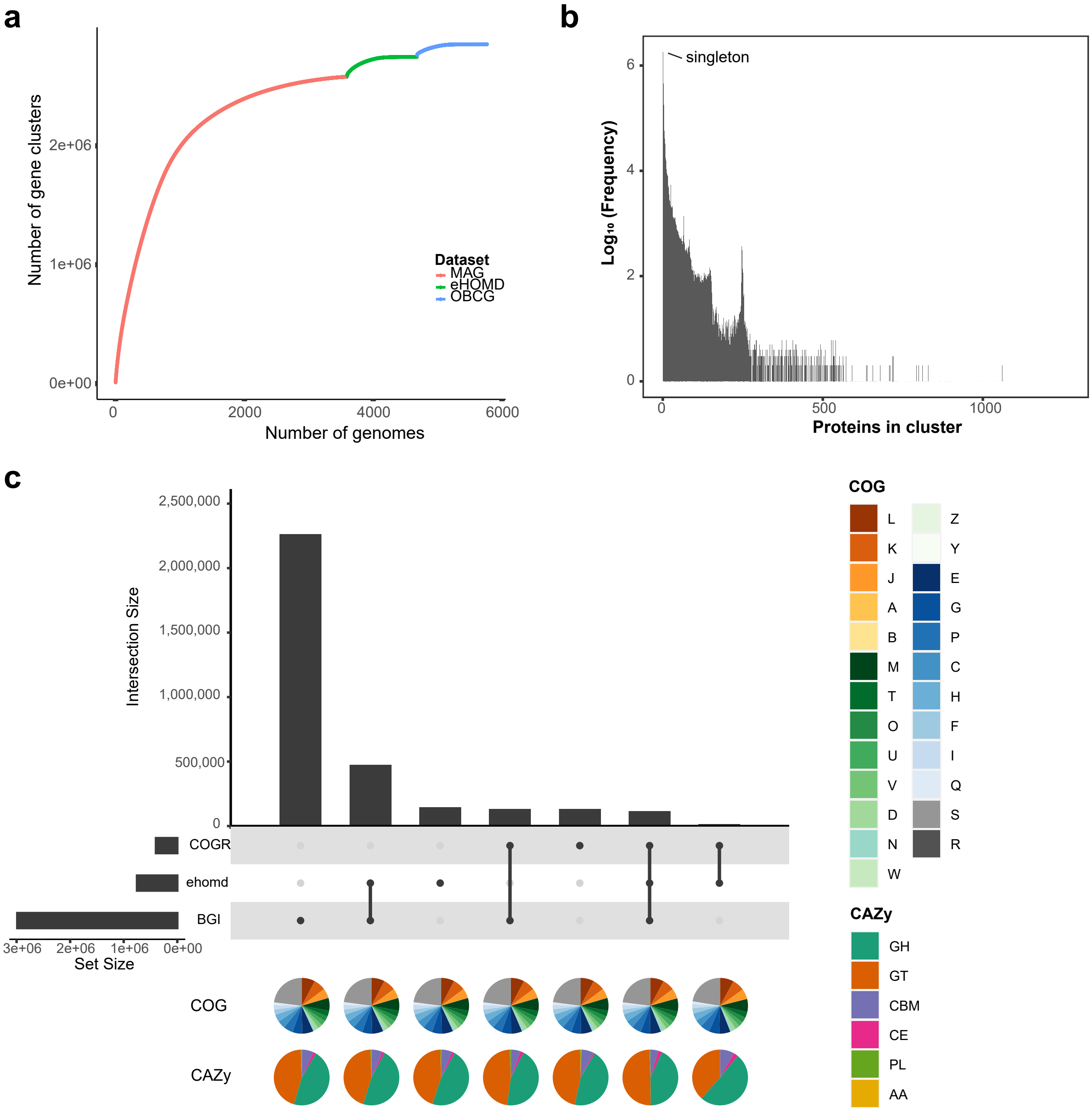

Supplementary Fig. 4 | Non-redundant protein sequences from MAGs, eHOMD and COGR. a, Gene accumulation curve of MAGs, eHOMD, and COGR. Genomes are sorted according to gene increase rate. **b,** Numbers of protein sequences contained in each protein cluster, most protein clusters contained only one sequence, termed singleton. **c**, Contribution of the three datasets to non-redundant protein sequences. Pie charts depict annotation categories in COG and CAZymers databases.

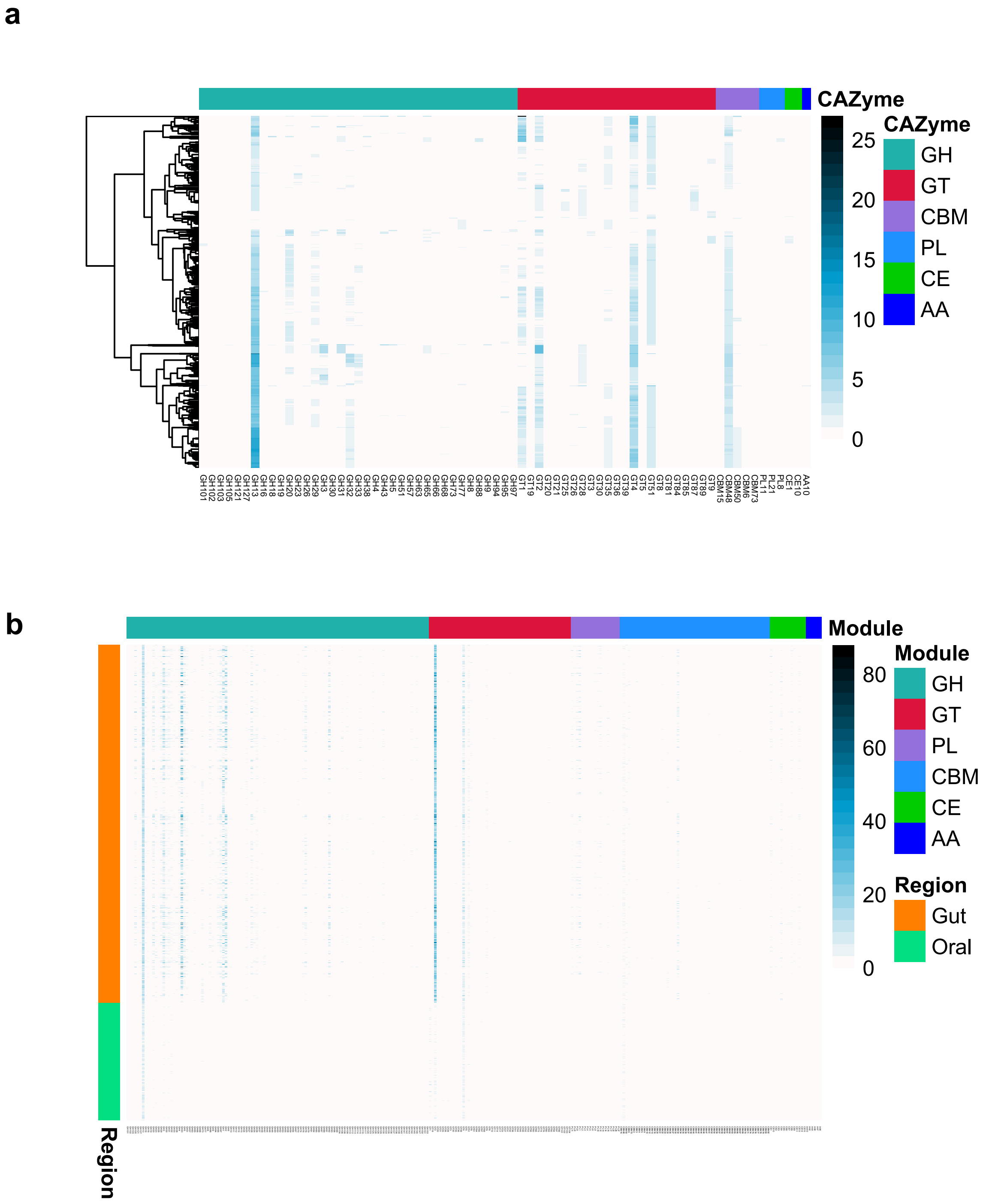

Supplementary Fig. 5 | Annotation of CAZymes in the oral and gut genome. a, The distribution of genes annotated to each CAZyme family in each COGR genome. **b**, The distribution of genes annotated to each CAZyme family in each COGR and gut (CGR2) genome.

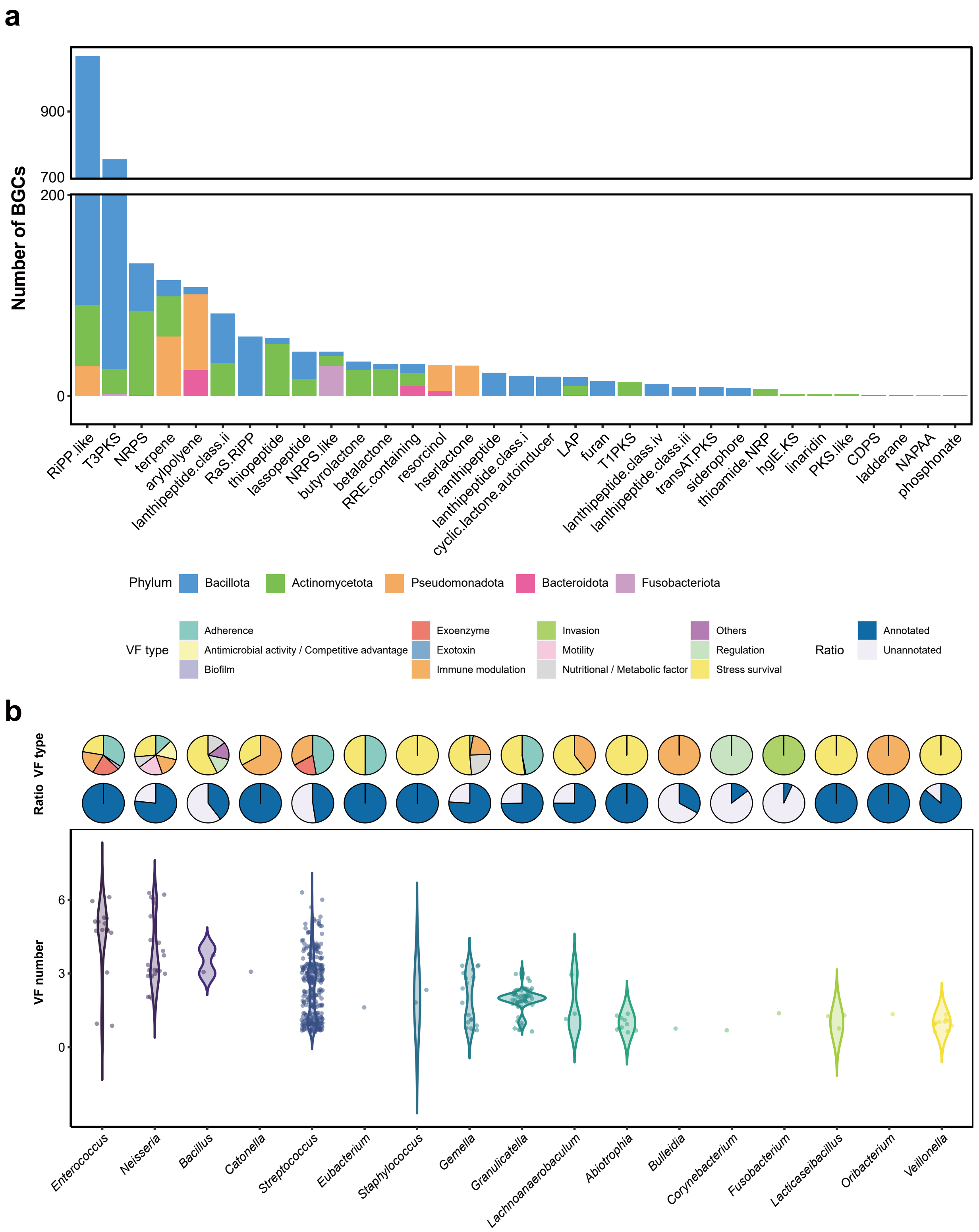

Supplementary Fig. 6 | Annotation of BGCs and VFs in the COGR genomes. a, The distribution of BGCs in different phyla. **b,** The distribution of VFs at the genus level. The Y-axis represents the number of VFs, and the pie chart represents the categories of VFs and the annotation rate at the genus level.

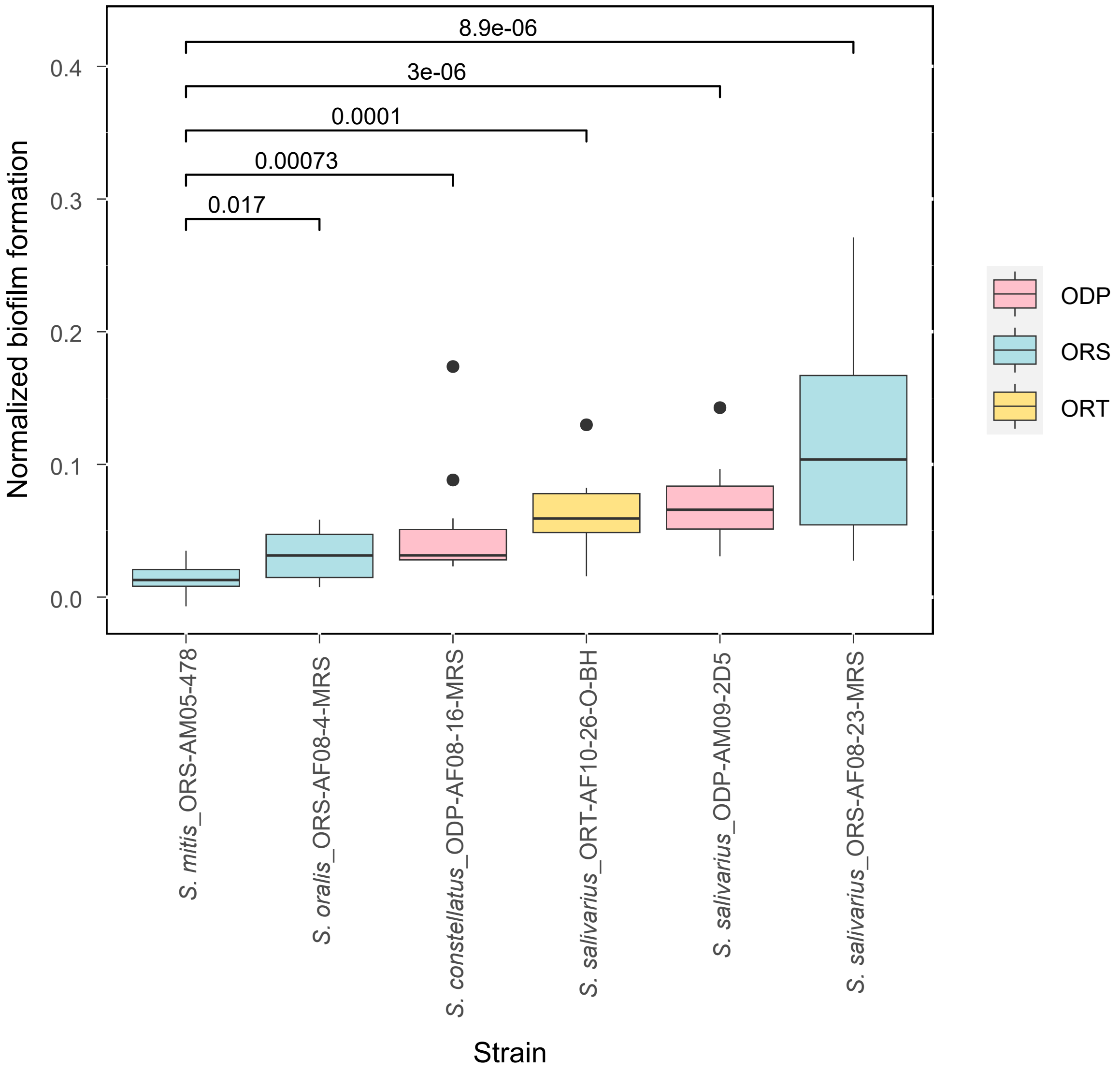

Supplementary Fig. 7 | Crystal violet assay for determining biofilm formation of selected strains. The boxplot of OD values of selected strains was colored by their species. *S. mitis*_ORS-AM05-478, which does not harbor intact quorum sensing pathways was used as a reference. The p-values presented in the figure were calculated using the wilcoxon test (two-sided), 12 replicates for each strain.

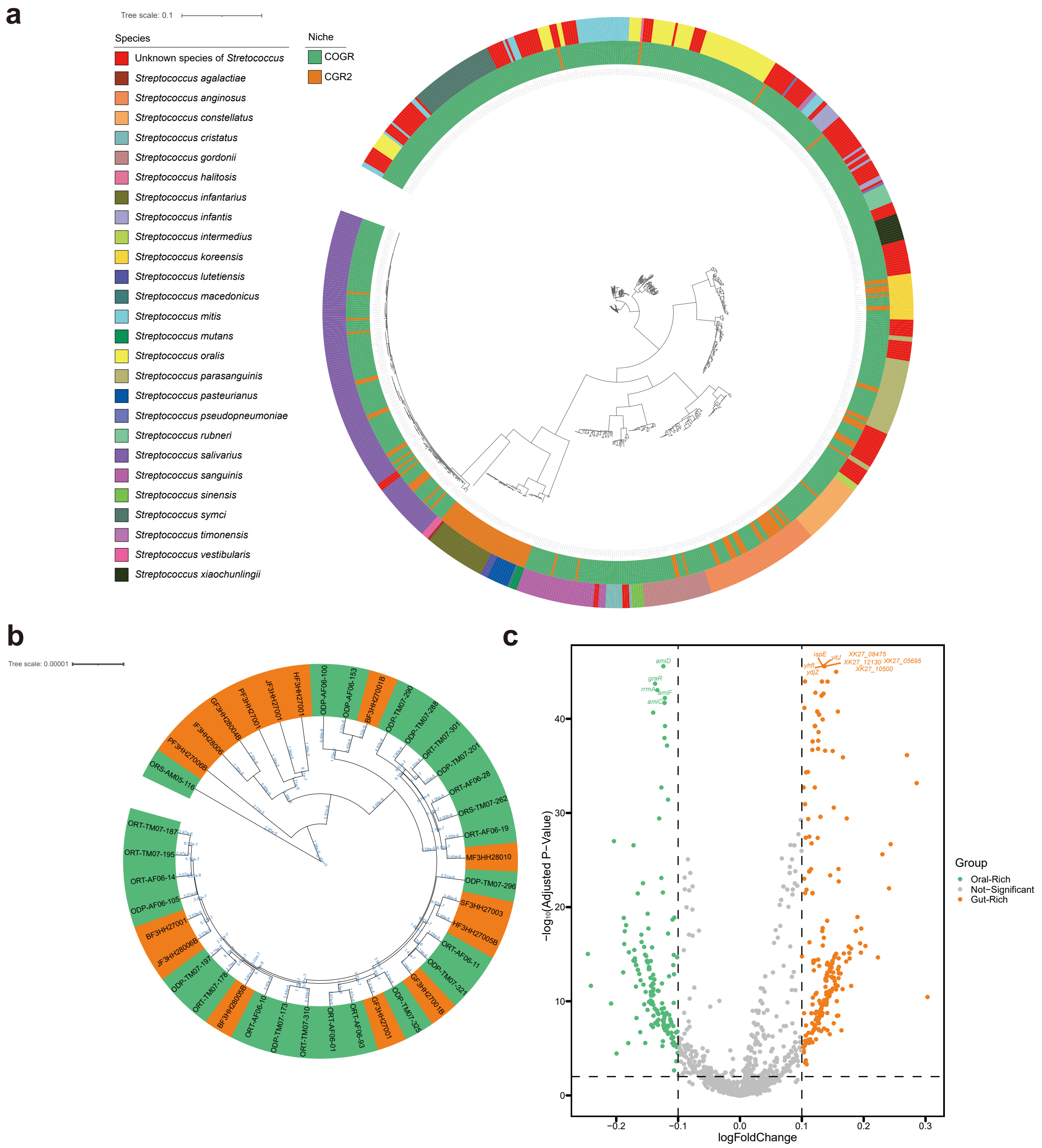

Supplementary Fig. 8 | Comparison between COGR and CGR2. a, Phylogenetic tree of genomes assigned to Streptococcus in COGR and CGR2. The inner circle is colored according to the oral location for sampling, and the outer circle is colored by assigned species **b**, Phylogenetic tree of genomes of *Microbacterium algeriense*. The circle is colored according to the origin of the bacterial isolates, and the Jaccard distance of genomes is shown on the branches. **c**, Differential proteins coded by *Streptococcus* in COGR and CGR2, and top 5 -$\log_{10}$ (Adjusted p-value) proteins are marked.

Supplementary Table 1. Information of cultivated strains and statistics for sequencing data of 1,089 high-quality genomes

Genome ID	Strain ID	Position	Gender	Age	Medium	Completeness	Contamination
BF3KA08004A	ORS-AF03-140	Oral- saliva	Female	Adult	BHI (Anaerobic)	99.25	0.96
BF3KA09001A	ORS-AF04-126	Oral- saliva	Female	Adult	BHI (Anaerobic)	100	0
BF3KA09002A	ORS-AF04-178	Oral- saliva	Female	Adult	BHI (Anaerobic)	100	0
BF3KA09004	ORS-AF04-35	Oral- saliva	Female	Adult	BHI (Aerobic)	100	0
BF3KA09004A	ORS-AF04-180	Oral- saliva	Female	Adult	BHI (Anaerobic)	100	0
BF3KA09005	ORS-AF04-36	Oral- saliva	Female	Adult	BHI (Aerobic)	99.83	0.58
BF3KA09008A	ORS-AF04-232	Oral- saliva	Female	Adult	BHI (Anaerobic)	99.66	0.34
BF3KA09009A	ORS-AF04-233	Oral- saliva	Female	Adult	BHI (Anaerobic)	100	0.07
BF3KA09011A	ORS-AF04-252	Oral- saliva	Female	Adult	BHI (Anaerobic)	99.62	0.07
BF3KA10007A	ORS-AF05-07	Oral- saliva	Female	Adult	BHI (Anaerobic)	99.77	1.1
BF3KA10010A	ORS-AF05-10	Oral- saliva	Female	Adult	BHI (Anaerobic)	99.83	0.58
BF3KA10011A	ORS-AF05-11	Oral- saliva	Female	Adult	BHI (Anaerobic)	99.82	0.26
BF3KA10012A	ORS-AF05-12	Oral- saliva	Female	Adult	BHI (Anaerobic)	99.9	1.08
BF3KA10014A	ORS-AF05-220	Oral- saliva	Female	Adult	BHI (Anaerobic)	96.18	0
BF3KA11001	ORS-AM04-31	Oral- saliva	Male	Adult	BHI (Aerobic)	99.79	0.64
BF3KA12001	ORS-AM05-116	Oral- saliva	Male	Adult	BHI (Aerobic)	99.24	0.25
BF3KA12001AB	ORS-AM05-183	Oral- saliva	Male	Adult	Blood-BHI (Anaerobic)	99.87	0.7
BF3KA12001B	ORS-AM05-155	Oral- saliva	Male	Adult	Blood-BHI (Aerobic)	99.94	0.28
BF3KA12002	ORS-AM05-117	Oral- saliva	Male	Adult	BHI (Aerobic)	99.34	0
BF3KA12003A	ORS-AM05-344	Oral- saliva	Male	Adult	BHI (Anaerobic)	99.66	0.2
BF3KA12004B	ORS-AM05-325	Oral- saliva	Male	Adult	Blood-BHI (Aerobic)	99.92	0.23
BF3KA12005AB	ORS-AM05-355	Oral- saliva	Male	Adult	Blood-BHI (Anaerobic)	99.66	0.2
BF3KA12006A	ORS-AM05-478	Oral- saliva	Male	Adult	BHI (Anaerobic)	99.32	0
BF3KA12007AB	ORS-AM05-484	Oral- saliva	Male	Adult	Blood-BHI (Anaerobic)	99.84	0.15
BF3KA13001A	ORS-TM06-82	Oral- saliva	Male	Teenager	BHI (Anaerobic)	98.56	0
BF3KA13002A	ORS-TM06-83	Oral- saliva	Male	Teenager	BHI (Anaerobic)	99.45	0
BF3KA13003A	ORS-TM06-84	Oral- saliva	Male	Teenager	BHI (Anaerobic)	99.94	0.15
BF3KA13009A	ORS-TM06-215	Oral- saliva	Male	Teenager	BHI (Anaerobic)	100	0.6
BF3KA13013A	ORS-TM06-219	Oral- saliva	Male	Teenager	BHI (Anaerobic)	100	0.51
BF3KA14002A	ORS-TM07-02	Oral- saliva	Male	Teenager	BHI (Anaerobic)	99.63	0
BF3KA14002B	ORS-TM07-230	Oral- saliva	Male	Teenager	Blood-BHI (Aerobic)	99.9	0.15
BF3KA14003	ORS-TM07-215	Oral- saliva	Male	Teenager	BHI (Aerobic)	99.63	0.31
BF3KA14003A	ORS-TM07-03	Oral- saliva	Male	Teenager	BHI (Anaerobic)	99.45	0.18
BF3KA14003AB	ORS-TM07-38	Oral- saliva	Male	Teenager	Blood-BHI (Anaerobic)	100	0.34
BF3KA14004AB	ORS-TM07-39	Oral- saliva	Male	Teenager	Blood-BHI (Anaerobic)	99.66	0.68
BF3KA15001AB	ORS-AM08-50	Oral- saliva	Male	Adult	Blood-BHI (Anaerobic)	100	0
BF3KA15002A	ORS-AM08-24	Oral- saliva	Male	Adult	BHI (Anaerobic)	99.45	0.77
BF3KA15002AB	ORS-AM08-51	Oral- saliva	Male	Adult	Blood-BHI (Anaerobic)	99.45	0
BF3KA15004AB	ORS-AM08-167	Oral- saliva	Male	Adult	Blood-BHI (Anaerobic)	100	0
BF3KC09003A	ODP-AF04-151	Oral-tooth	Female	Adult	BHI (Anaerobic)	99.45	0
BF3KC09004A	ODP-AF04-152	Oral-tooth	Female	Adult	BHI (Anaerobic)	99.45	0
BF3KC09005	ODP-AF04-71	Oral-tooth	Female	Adult	BHI (Aerobic)	99.83	0.58
BF3KC09006A	ODP-AF04-188	Oral-tooth	Female	Adult	BHI (Anaerobic)	100	0.47
BF3KC09008	ODP-AF04-74	Oral-tooth	Female	Adult	BHI (Aerobic)	99.34	0
BF3KC09008A	ODP-AF04-238	Oral-tooth	Female	Adult	BHI (Anaerobic)	100	0
BF3KC11001	ODP-AM04-38	Oral-tooth	Male	Adult	BHI (Aerobic)	99.79	0.64
BF3KC11001A	ODP-AM04-93	Oral-tooth	Male	Adult	BHI (Anaerobic)	99.32	0.34
BF3KC12001A	ODP-AM05-237	Oral-tooth	Male	Adult	BHI (Anaerobic)	99.9	0.15
BF3KC12001AB	ODP-AM05-254	Oral-tooth	Male	Adult	Blood-BHI (Anaerobic)	100	0
BF3KC12002	ODP-AM05-18	Oral-tooth	Male	Adult	BHI (Aerobic)	99.34	0
BF3KC12002AB	ODP-AM05-255	Oral-tooth	Male	Adult	Blood-BHI (Anaerobic)	99.45	0
BF3KC12003	ODP-AM05-19	Oral-tooth	Male	Adult	BHI (Aerobic)	99.87	0.46
BF3KC12004B	ODP-AM05-113	Oral-tooth	Male	Adult	Blood-BHI (Aerobic)	98.34	0
BF3KC12006	ODP-AM05-22	Oral-tooth	Male	Adult	BHI (Aerobic)	99.45	0.11
BF3KC12006A	ODP-AM05-411	Oral-tooth	Male	Adult	BHI (Anaerobic)	100	1.18
BF3KC13001A	ODP-TM06-143	Oral-tooth	Male	Teenager	BHI (Anaerobic)	99.9	0.67
BF3KC13003A	ODP-TM06-145	Oral-tooth	Male	Teenager	BHI (Anaerobic)	99.45	0.55
BF3KC14001	ODP-TM07-197	Oral-tooth	Male	Teenager	BHI (Aerobic)	99.49	0.76
BF3KC14001A	ODP-TM07-115	Oral-tooth	Male	Teenager	BHI (Anaerobic)	99.45	0.18
BF3KC14001AB	ODP-TM07-145	Oral-tooth	Male	Teenager	Blood-BHI (Anaerobic)	99.45	0
BF3KC14001B	ODP-TM07-209	Oral-tooth	Male	Teenager	Blood-BHI (Aerobic)	99.83	0.2
BF3KC14002	ODP-TM07-198	Oral-tooth	Male	Teenager	BHI (Aerobic)	100	0
BF3KC14003B	ODP-TM07-325	Oral-tooth	Male	Teenager	Blood-BHI (Aerobic)	99.49	0.76
BF3KC15001A	ODP-AM08-279	Oral-tooth	Male	Adult	BHI (Anaerobic)	99.84	0.25
BF3KC15002A	ODP-AM08-280	Oral-tooth	Male	Adult	BHI (Anaerobic)	99.45	0.77
BF3KC15004A	ODP-AM08-282	Oral-tooth	Male	Adult	BHI (Anaerobic)	98.46	0
BF3KC15004C	ODP-AM08-246	Oral-tooth	Male	Adult	BHI (Aerobic)	99.34	0
BF3KC15006A	ODP-AM08-284	Oral-tooth	Male	Adult	BHI (Anaerobic)	100	0.47
BF3KC15011A	ODP-AM08-377	Oral-tooth	Male	Adult	BHI (Anaerobic)	100	0.47
BF3KC15013A	ODP-AM08-379	Oral-tooth	Male	Adult	BHI (Anaerobic)	100	0.47
BF3KC15015A	ODP-AM08-381	Oral-tooth	Male	Adult	BHI (Anaerobic)	100	1.18
BF3KC15016A	ODP-AM08-382	Oral-tooth	Male	Adult	BHI (Anaerobic)	100	0.71
BF3KC15020A	ODP-AM08-386	Oral-tooth	Male	Adult	BHI (Anaerobic)	100	0
BF3KC16005	ODP-AF06-100	Oral-tooth	Female	Adult	BHI (Aerobic)	99.49	0.76
BF3KT08002	ORT-AF03-65	Oral-tongue coat	Female	Adult	BHI (Aerobic)	99.11	0
BF3KT08005	ORT-AF03-68	Oral-tongue coat	Female	Adult	BHI (Aerobic)	97.22	0.29
BF3KT09001	ORT-AF04-01	Oral-tongue coat	Female	Adult	BHI (Aerobic)	99.83	0.58
BF3KT09001A	ORT-AF04-115	Oral-tongue coat	Female	Adult	BHI (Anaerobic)	100	0
BF3KT09002A	ORT-AF04-116	Oral-tongue coat	Female	Adult	BHI (Anaerobic)	100	0.17
BF3KT09003A	ORT-AF04-169	Oral-tongue coat	Female	Adult	BHI (Anaerobic)	100	0.47
BF3KT09005A	ORT-AF04-215	Oral-tongue coat	Female	Adult	BHI (Anaerobic)	99.46	0
BF3KT09006A	ORT-AF04-216	Oral-tongue coat	Female	Adult	BHI (Anaerobic)	100	0.17
BF3KT09007A	ORT-AF04-246	Oral-tongue coat	Female	Adult	BHI (Anaerobic)	99.62	0.07

BF3KT11001	ORT-AM04-01	Oral-tongue coat	Male	Adult	BHI (Aerobic)	99.79	0.64
BF3KT11001A	ORT-AM04-53	Oral-tongue coat	Male	Adult	BHI (Anaerobic)	99.9	0.58
BF3KT11003A	ORT-AM04-76	Oral-tongue coat	Male	Adult	BHI (Anaerobic)	99.45	0
BF3KT12001B	ORT-AM05-52	Oral-tongue coat	Male	Adult	Blood-BHI (Aerobic)	99.67	0.23
BF3KT12005	ORT-AM05-291	Oral-tongue coat	Male	Adult	BHI (Aerobic)	99.4	0.68
BF3KT12005AB	ORT-AM05-406	Oral-tongue coat	Male	Adult	Blood-BHI (Anaerobic)	100	0.47
BF3KT12006A	ORT-AM05-398	Oral-tongue coat	Male	Adult	BHI (Anaerobic)	100	0
BF3KT12006AB	ORT-AM05-453	Oral-tongue coat	Male	Adult	BHI (Aerobic)	99	0.09
BF3KT12007A	ORT-AM05-445	Oral-tongue coat	Male	Adult	BHI (Aerobic)	99.45	0.27
BF3KT12008A	ORT-AM05-446	Oral-tongue coat	Male	Adult	BHI (Aerobic)	100	0.34
BF3KT13002	ORT-TM06-63	Oral-tongue coat	Male	Teenager	BHI (Aerobic)	98.08	0
BF3KT14001	ORT-TM07-175	Oral-tongue coat	Male	Teenager	BHI (Aerobic)	96.84	0
BF3KT14001A	ORT-TM07-80	Oral-tongue coat	Male	Teenager	BHI (Anaerobic)	99.45	0.27
BF3KT14001B	ORT-TM07-301	Oral-tongue coat	Male	Teenager	Blood-BHI (Aerobic)	99.49	0.76
BF3KT14002AB	ORT-TM07-98	Oral-tongue coat	Male	Teenager	Blood-BHI (Anaerobic)	99.45	0
BF3KT14003AB	ORT-TM07-99	Oral-tongue coat	Male	Teenager	Blood-BHI (Anaerobic)	100	0.34
BF3KT15001	ORT-AM08-194	Oral-tongue coat	Male	Adult	BHI (Aerobic)	99.79	0.15
BF3KT15002	ORT-AM08-195	Oral-tongue coat	Male	Adult	BHI (Aerobic)	99.17	0.3
BF3KT15003B	ORT-AM08-219	Oral-tongue coat	Male	Adult	Blood-BHI (Aerobic)	100	0
BF3KT16001	ORT-AF06-01	Oral-tongue coat	Female	Adult	BHI (Aerobic)	99.49	0.76
GF3KA08002B	ORS-AF03-49	Oral- saliva	Female	Adult	Blood-Columbia Medium (Aerobic)	100	0.47
GF3KA09001A	ORS-AF04-127	Oral- saliva	Female	Adult	Columbia Medium (Anaerobic)	100	0
GF3KA09003A	ORS-AF04-129	Oral- saliva	Female	Adult	Columbia Medium (Anaerobic)	100	0.41
GF3KA09004	ORS-AF04-41	Oral- saliva	Female	Adult	Columbia Medium (Aerobic)	100	0
GF3KA09004A	ORS-AF04-183	Oral- saliva	Female	Adult	Columbia Medium (Anaerobic)	100	0
GF3KA09005	ORS-AF04-42	Oral- saliva	Female	Adult	Columbia Medium (Aerobic)	99.82	0.26
GF3KA09006A	ORS-AF04-234	Oral- saliva	Female	Adult	Columbia Medium (Anaerobic)	100	0.18
GF3KA10002A	ORS-AF05-14	Oral- saliva	Female	Adult	Columbia Medium (Anaerobic)	99.9	1.08
GF3KA10003A	ORS-AF05-15	Oral- saliva	Female	Adult	Columbia Medium (Anaerobic)	99.83	0.58
GF3KA10004A	ORS-AF05-16	Oral- saliva	Female	Adult	Columbia Medium (Anaerobic)	99.9	1.08
GF3KA10006	ORS-AF05-242	Oral- saliva	Female	Adult	Columbia Medium (Aerobic)	99.46	0.58
GF3KA10007A	ORS-AF05-19	Oral- saliva	Female	Adult	Columbia Medium (Anaerobic)	98.28	0.14
GF3KA11002A	ORS-AM04-85	Oral- saliva	Male	Adult	Columbia Medium (Anaerobic)	99.45	0
GF3KA12001	ORS-AM05-121	Oral- saliva	Male	Adult	Columbia Medium (Aerobic)	99.94	0.28
GF3KA12002	ORS-AM05-122	Oral- saliva	Male	Adult	Columbia Medium (Aerobic)	99.87	0.46
GF3KA12004	ORS-AM05-124	Oral- saliva	Male	Adult	Columbia Medium (Aerobic)	99.4	0
GF3KA12006AB	ORS-AM05-357	Oral- saliva	Male	Adult	Blood-Columbia Medium (Anaerobic)	100	0.17
GF3KA12007AB	ORS-AM05-485	Oral- saliva	Male	Adult	Blood-Columbia Medium (Anaerobic)	99.66	0.2
GF3KA13001A	ORS-TM06-89	Oral- saliva	Male	Teenager	Columbia Medium (Anaerobic)	99.63	0.37
GF3KA13002	ORS-TM06-15	Oral- saliva	Male	Teenager	Columbia Medium (Aerobic)	99.95	0.51
GF3KA13002A	ORS-TM06-90	Oral- saliva	Male	Teenager	Columbia Medium (Anaerobic)	98.46	0
GF3KA13003	ORS-TM06-16	Oral- saliva	Male	Teenager	Columbia Medium (Aerobic)	100	1.42
GF3KA13003A	ORS-TM06-91	Oral- saliva	Male	Teenager	Columbia Medium (Anaerobic)	98.91	0.55
GF3KA13005A	ORS-TM06-93	Oral- saliva	Male	Teenager	Columbia Medium (Anaerobic)	99.45	0
GF3KA13006	ORS-TM06-240	Oral- saliva	Male	Teenager	Columbia Medium (Aerobic)	99.11	0
GF3KA13006A	ORS-TM06-94	Oral- saliva	Male	Teenager	Columbia Medium (Anaerobic)	98.91	0.55
GF3KA13011A	ORS-TM06-222	Oral- saliva	Male	Teenager	Columbia Medium (Anaerobic)	99.84	0.15
GF3KA14001AB	ORS-TM07-40	Oral- saliva	Male	Teenager	Blood-Columbia Medium (Anaerobic)	99.63	0
GF3KA15001A	ORS-AM08-28	Oral- saliva	Male	Adult	Columbia Medium (Anaerobic)	100	0.17
GF3KA15003A	ORS-AM08-30	Oral- saliva	Male	Adult	Columbia Medium (Anaerobic)	100	0
GF3KA15005AB	ORS-AM08-168	Oral- saliva	Male	Adult	Blood-Columbia Medium (Anaerobic)	99.66	0.11
GF3KA16003	ORS-AF06-69	Oral- saliva	Female	Adult	Columbia Medium (Aerobic)	100	0
GF3KC09002A	ODP-AF04-155	Oral-tooth	Female	Adult	Columbia Medium (Anaerobic)	99.45	0
GF3KC09003	ODP-AF04-78	Oral-tooth	Female	Adult	Columbia Medium (Aerobic)	99.11	0
GF3KC09005A	ODP-AF04-158	Oral-tooth	Female	Adult	Columbia Medium (Anaerobic)	99.45	0
GF3KC09008A	ODP-AF04-190	Oral-tooth	Female	Adult	Columbia Medium (Anaerobic)	100	0.47
GF3KC10001A	ODP-AF05-68	Oral-tooth	Female	Adult	Columbia Medium (Anaerobic)	100	0.23
GF3KC10002A	ODP-AF05-69	Oral-tooth	Female	Adult	Columbia Medium (Anaerobic)	100	0.23
GF3KC11001A	ODP-AM04-97	Oral-tooth	Male	Adult	Columbia Medium (Anaerobic)	99.32	0
GF3KC11002A	ODP-AM04-98	Oral-tooth	Male	Adult	Columbia Medium (Anaerobic)	99.32	0
GF3KC11003A	ODP-AM04-99	Oral-tooth	Male	Adult	Columbia Medium (Anaerobic)	100	0
GF3KC11005A	ODP-AM04-101	Oral-tooth	Male	Adult	Columbia Medium (Anaerobic)	100	0
GF3KC12001	ODP-AM05-24	Oral-tooth	Male	Adult	Columbia Medium (Aerobic)	100	0
GF3KC12002	ODP-AM05-25	Oral-tooth	Male	Adult	Columbia Medium (Aerobic)	99.87	0.2
GF3KC12002A	ODP-AM05-415	Oral-tooth	Male	Adult	Columbia Medium (Anaerobic)	100	0
GF3KC12002B	ODP-AM05-306	Oral-tooth	Male	Adult	Blood-Columbia Medium (Aerobic)	99.66	0.45
GF3KC12004B	ODP-AM05-321	Oral-tooth	Male	Adult	Blood-Columbia Medium (Aerobic)	99.7	0
GF3KC12005AB	ODP-AM05-388	Oral-tooth	Male	Adult	Blood-Columbia Medium (Anaerobic)	98.37	0
GF3KC12006A	ODP-AM05-419	Oral-tooth	Male	Adult	Columbia Medium (Anaerobic)	100	0.47
GF3KC12007A	ODP-AM05-420	Oral-tooth	Male	Adult	Columbia Medium (Anaerobic)	100	1.42
GF3KC12007AB	ODP-AM05-461	Oral-tooth	Male	Adult	Columbia Medium (Aerobic)	100	1.42
GF3KC12010A	ODP-AM05-423	Oral-tooth	Male	Adult	Columbia Medium (Anaerobic)	100	1.7
GF3KC12011A	ODP-AM05-501	Oral-tooth	Male	Adult	Columbia Medium (Anaerobic)	100	0
GF3KC13001A	ODP-TM06-148	Oral-tooth	Male	Teenager	Columbia Medium (Anaerobic)	99.49	0.17
GF3KC13002A	ODP-TM06-149	Oral-tooth	Male	Teenager	Columbia Medium (Anaerobic)	100	0.17
GF3KC13003A	ODP-TM06-150	Oral-tooth	Male	Teenager	Columbia Medium (Anaerobic)	99.49	0.17
GF3KC14001A	ODP-TM07-125	Oral-tooth	Male	Teenager	Columbia Medium (Anaerobic)	99.45	0
GF3KC14002	ODP-TM07-288	Oral-tooth	Male	Teenager	Columbia Medium (Aerobic)	99.49	0.76
GF3KC14002AB	ODP-TM07-152	Oral-tooth	Male	Teenager	Blood-Columbia Medium (Anaerobic)	99.45	0
GF3KC15001A	ODP-AM08-289	Oral-tooth	Male	Adult	Columbia Medium (Anaerobic)	99.45	0.82
GF3KC15002	ODP-AM08-343	Oral-tooth	Male	Adult	Columbia Medium (Aerobic)	100	0
GF3KC15004A	ODP-AM08-292	Oral-tooth	Male	Adult	Columbia Medium (Anaerobic)	100	0
GF3KC15006B	ODP-AM08-257	Oral-tooth	Male	Adult	Blood-Columbia Medium (Aerobic)	100	0.47
GF3KC15009A	ODP-AM08-297	Oral-tooth	Male	Adult	Columbia Medium (Anaerobic)	98.46	0
GF3KT09001A	ORT-AF04-117	Oral-tongue coat	Female	Adult	Columbia Medium (Anaerobic)	100	0.23
GF3KT09002A	ORT-AF04-118	Oral-tongue coat	Female	Adult	Columbia Medium (Anaerobic)	100	0.23

GF3KT09003A	ORT-AF04-170	Oral-tongue coat	Female	Adult	Columbia Medium (Anaerobic)	100	0
GF3KT09005	ORT-AF04-192	Oral-tongue coat	Female	Adult	Columbia Medium (Aerobic)	99.83	0.58
GF3KT09005A	ORT-AF04-217	Oral-tongue coat	Female	Adult	Columbia Medium (Anaerobic)	100	0.15
GF3KT10001A	ORT-AF05-103	Oral-tongue coat	Female	Adult	Columbia Medium (Anaerobic)	100	0.41
GF3KT10002A	ORT-AF05-104	Oral-tongue coat	Female	Adult	Columbia Medium (Anaerobic)	100	0
GF3KT10003A	ORT-AF05-105	Oral-tongue coat	Female	Adult	Columbia Medium (Anaerobic)	100	0
GF3KT10004A	ORT-AF05-106	Oral-tongue coat	Female	Adult	Columbia Medium (Anaerobic)	99.83	0.58
GF3KT10006A	ORT-AF05-108	Oral-tongue coat	Female	Adult	Columbia Medium (Anaerobic)	99.83	0.58
GF3KT11001	ORT-AM04-05	Oral-tongue coat	Male	Adult	Columbia Medium (Aerobic)	100	0
GF3KT11002	ORT-AM04-06	Oral-tongue coat	Male	Adult	Columbia Medium (Aerobic)	99.33	0
GF3KT11003	ORT-AM04-07	Oral-tongue coat	Male	Adult	Columbia Medium (Aerobic)	100	0.34
GF3KT11003A	ORT-AM04-127	Oral-tongue coat	Male	Adult	Columbia Medium (Anaerobic)	100	0
GF3KT12001	ORT-AM05-05	Oral-tongue coat	Male	Adult	Columbia Medium (Aerobic)	99.32	0.47
GF3KT12002AB	ORT-AM05-94	Oral-tongue coat	Male	Adult	Blood-Columbia Medium (Anaerobic)	99.87	0.3
GF3KT12002B	ORT-AM05-110	Oral-tongue coat	Male	Adult	Blood-Columbia Medium (Aerobic)	99.33	3.56
GF3KT12003AB	ORT-AM05-95	Oral-tongue coat	Male	Adult	Blood-Columbia Medium (Anaerobic)	96.2	0
GF3KT12004A	ORT-AM05-449	Oral-tongue coat	Male	Adult	Columbia Medium (Aerobic)	99.32	0
GF3KT12004AB	ORT-AM05-96	Oral-tongue coat	Male	Adult	Blood-Columbia Medium (Anaerobic)	99.45	0.27
GF3KT12005A	ORT-AM05-450	Oral-tongue coat	Male	Adult	Columbia Medium (Aerobic)	99.66	0
GF3KT12006AB	ORT-AM05-288	Oral-tongue coat	Male	Adult	Blood-Columbia Medium (Anaerobic)	99.45	0
GF3KT12008A	ORT-AM05-520	Oral-tongue coat	Male	Adult	Columbia Medium (Anaerobic)	100	0.47
GF3KT12009AB	ORT-AM05-455	Oral-tongue coat	Male	Adult	Columbia Medium (Aerobic)	98.28	0
GF3KT13001A	ORT-TM06-169	Oral-tongue coat	Male	Teenager	Columbia Medium (Anaerobic)	99.49	0.17
GF3KT13002A	ORT-TM06-170	Oral-tongue coat	Male	Teenager	Columbia Medium (Anaerobic)	99.45	0.27
GF3KT13003	ORT-TM06-66	Oral-tongue coat	Male	Teenager	Columbia Medium (Aerobic)	100	0.07
GF3KT14001	ORT-TM07-178	Oral-tongue coat	Male	Teenager	Columbia Medium (Aerobic)	99.49	0.76
GF3KT15003	ORT-AM08-200	Oral-tongue coat	Male	Adult	Columbia Medium (Aerobic)	99.79	0.15
GF3KT15004B	ORT-AM08-223	Oral-tongue coat	Male	Adult	Blood-Columbia Medium (Aerobic)	99.33	0
GF3KT16003	ORT-AF06-92	Oral-tongue coat	Female	Adult	Columbia Medium (Aerobic)	99.33	0
HF3KA08001A	ORS-AF03-141	Oral- saliva	Female	Adult	H Medium (Anaerobic)	99.83	1.06
HF3KA08001B	ORS-AF03-52	Oral- saliva	Female	Adult	Blood-H Medium (Aerobic)	99.34	0
HF3KA08002A	ORS-AF03-142	Oral- saliva	Female	Adult	H Medium (Anaerobic)	99.25	0.96
HF3KA08006B	ORS-AF03-251	Oral- saliva	Female	Adult	Blood-H Medium (Aerobic)	100	0.47
HF3KA09001A	ORS-AF04-136	Oral- saliva	Female	Adult	H Medium (Anaerobic)	99.83	0.58
HF3KA09002A	ORS-AF04-137	Oral- saliva	Female	Adult	H Medium (Anaerobic)	99.83	0.58
HF3KA09005A	ORS-AF04-140	Oral- saliva	Female	Adult	H Medium (Anaerobic)	99.45	0
HF3KA10004A	ORS-AF05-23	Oral- saliva	Female	Adult	H Medium (Anaerobic)	99.83	0.58
HF3KA10005A	ORS-AF05-24	Oral- saliva	Female	Adult	H Medium (Anaerobic)	99.83	0.58
HF3KA11001A	ORS-AM04-86	Oral- saliva	Male	Adult	H Medium (Anaerobic)	100	0
HF3KA12001	ORS-AM05-127	Oral- saliva	Male	Adult	H Medium (Aerobic)	99.4	0
HF3KA12001A	ORS-AM05-172	Oral- saliva	Male	Adult	H Medium (Anaerobic)	99.4	0
HF3KA12002	ORS-AM05-128	Oral- saliva	Male	Adult	H Medium (Aerobic)	99	0.09
HF3KA12002AB	ORS-AM05-193	Oral- saliva	Male	Adult	Blood-H Medium (Anaerobic)	100	0.34
HF3KA12003AB	ORS-AM05-194	Oral- saliva	Male	Adult	Blood-H Medium (Anaerobic)	99.87	0.3
HF3KA12004B	ORS-AM05-167	Oral- saliva	Male	Adult	Blood-H Medium (Aerobic)	99.4	0
HF3KA12005AB	ORS-AM05-196	Oral- saliva	Male	Adult	Blood-H Medium (Anaerobic)	99.34	0
HF3KA12006A	ORS-AM05-374	Oral- saliva	Male	Adult	H Medium (Anaerobic)	99.52	0
HF3KA12006AB	ORS-AM05-358	Oral- saliva	Male	Adult	Blood-H Medium (Anaerobic)	100	0.56
HF3KA12009AB	ORS-AM05-381	Oral- saliva	Male	Adult	Blood-H Medium (Anaerobic)	100	0
HF3KA13001	ORS-TM06-19	Oral- saliva	Male	Teenager	H Medium (Aerobic)	99.34	0
HF3KA13002A	ORS-TM06-96	Oral- saliva	Male	Teenager	H Medium (Anaerobic)	99.63	0
HF3KA13003A	ORS-TM06-97	Oral- saliva	Male	Teenager	H Medium (Anaerobic)	98.08	0
HF3KA13005A	ORS-TM06-99	Oral- saliva	Male	Teenager	H Medium (Anaerobic)	99.45	0
HF3KA13006A	ORS-TM06-100	Oral- saliva	Male	Teenager	H Medium (Anaerobic)	98.37	0
HF3KA13012A	ORS-TM06-224	Oral- saliva	Male	Teenager	H Medium (Anaerobic)	99.4	1.02
HF3KA13014A	ORS-TM06-226	Oral- saliva	Male	Teenager	H Medium (Anaerobic)	100	0
HF3KA14001	ORS-TM07-219	Oral- saliva	Male	Teenager	H Medium (Aerobic)	99.82	0.2
HF3KA14004A	ORS-TM07-11	Oral- saliva	Male	Teenager	H Medium (Anaerobic)	99.63	0
HF3KA15001A	ORS-AM08-152	Oral- saliva	Male	Adult	H Medium (Anaerobic)	100	0.47
HF3KA15001AB	ORS-AM08-56	Oral- saliva	Male	Adult	Blood-H Medium (Anaerobic)	99.88	0
HF3KA15004AB	ORS-AM08-171	Oral- saliva	Male	Adult	Blood-H Medium (Anaerobic)	99.45	0
HF3KA15004B	ORS-AM08-101	Oral- saliva	Male	Adult	Blood-H Medium (Aerobic)	99.33	0
HF3KA16008	ORS-AF06-121	Oral- saliva	Female	Adult	H Medium (Aerobic)	100	0
HF3KA16011	ORS-AF06-159	Oral- saliva	Female	Adult	H Medium (Aerobic)	99.66	0.17
HF3KC09003	ODP-AF04-83	Oral-tooth	Female	Adult	H Medium (Aerobic)	99.77	1.1
HF3KC12001AB	ODP-AM05-262	Oral-tooth	Male	Adult	Blood-H Medium (Anaerobic)	99	0.09
HF3KC12002A	ODP-AM05-425	Oral-tooth	Male	Adult	H Medium (Anaerobic)	100	0
HF3KC12002AB	ODP-AM05-263	Oral-tooth	Male	Adult	Blood-H Medium (Anaerobic)	99.57	0.6
HF3KC12003AB	ODP-AM05-390	Oral-tooth	Male	Adult	Blood-H Medium (Anaerobic)	100	0.95
HF3KC12004AB	ODP-AM05-456	Oral-tooth	Male	Adult	H Medium (Aerobic)	99.37	0
HF3KC12005A	ODP-AM05-463	Oral-tooth	Male	Adult	H Medium (Aerobic)	100	0
HF3KC12005AB	ODP-AM05-457	Oral-tooth	Male	Adult	H Medium (Aerobic)	99.82	0.54
HF3KC12005B	ODP-AM05-322	Oral-tooth	Male	Adult	Blood-H Medium (Aerobic)	99.11	0
HF3KC12006AB	ODP-AM05-458	Oral-tooth	Male	Adult	H Medium (Aerobic)	99.82	0.54
HF3KC12007	ODP-AM05-296	Oral-tooth	Male	Adult	H Medium (Aerobic)	99.34	0
HF3KC13001	ODP-TM06-47	Oral-tooth	Male	Teenager	H Medium (Aerobic)	100	0
HF3KC13001A	ODP-TM06-151	Oral-tooth	Male	Teenager	H Medium (Anaerobic)	99.84	0.15
HF3KC13002A	ODP-TM06-152	Oral-tooth	Male	Teenager	H Medium (Anaerobic)	100	0.47
HF3KC13003A	ODP-TM06-153	Oral-tooth	Male	Teenager	H Medium (Anaerobic)	98.37	0
HF3KC13005A	ODP-TM06-155	Oral-tooth	Male	Teenager	H Medium (Anaerobic)	99.45	0
HF3KC14001AB	ODP-TM07-155	Oral-tooth	Male	Teenager	Blood-H Medium (Anaerobic)	99.45	0
HF3KC14002	ODP-TM07-290	Oral-tooth	Male	Teenager	H Medium (Aerobic)	100	0.76
HF3KC14003AB	ODP-TM07-157	Oral-tooth	Male	Teenager	Blood-H Medium (Anaerobic)	100	0
HF3KC15001AB	ODP-AM08-403	Oral-tooth	Male	Adult	Blood-H Medium (Anaerobic)	99.45	0
HF3KC15002	ODP-AM08-350	Oral-tooth	Male	Adult	H Medium (Aerobic)	99.9	0.15
HF3KC15002A	ODP-AM08-302	Oral-tooth	Male	Adult	H Medium (Anaerobic)	99.75	0.25

HF3KC15004A	ODP-AM08-304	Oral-tooth	Male	Adult	H Medium (Anaerobic)	98.56	0.57
HF3KC15009A	ODP-AM08-392	Oral-tooth	Male	Adult	H Medium (Anaerobic)	100	0
HF3KC15010A	ODP-AM08-393	Oral-tooth	Male	Adult	H Medium (Anaerobic)	98.56	0
HF3KC16002B	ODP-AF06-108	Oral-tooth	Female	Adult	Blood-H Medium (Aerobic)	99.78	0.6
HF3KT08002	ORT-AF03-76	Oral-tongue coat	Female	Adult	H Medium (Aerobic)	98.37	0
HF3KT08003A	ORT-AF03-129	Oral-tongue coat	Female	Adult	H Medium (Anaerobic)	100	1.44
HF3KT09001	ORT-AF04-11	Oral-tongue coat	Female	Adult	H Medium (Aerobic)	99.83	0.58
HF3KT09002A	ORT-AF04-173	Oral-tongue coat	Female	Adult	H Medium (Anaerobic)	99.32	0.34
HF3KT09003A	ORT-AF04-221	Oral-tongue coat	Female	Adult	H Medium (Anaerobic)	100	0.07
HF3KT10001A	ORT-AF05-109	Oral-tongue coat	Female	Adult	H Medium (Anaerobic)	99.63	0
HF3KT10005A	ORT-AF05-113	Oral-tongue coat	Female	Adult	H Medium (Anaerobic)	99.45	0.27
HF3KT10010A	ORT-AF05-230	Oral-tongue coat	Female	Adult	H Medium (Anaerobic)	100	0
HF3KT11004	ORT-AM04-12	Oral-tongue coat	Male	Adult	H Medium (Aerobic)	98.56	0.57
HF3KT12001A	ORT-AM05-89	Oral-tongue coat	Male	Adult	H Medium (Anaerobic)	99.47	0.2
HF3KT12001B	ORT-AM05-54	Oral-tongue coat	Male	Adult	Blood-H Medium (Aerobic)	99.67	0.23
HF3KT12002	ORT-AM05-111	Oral-tongue coat	Male	Adult	H Medium (Aerobic)	99.92	0.45
HF3KT12003	ORT-AM05-314	Oral-tongue coat	Male	Adult	H Medium (Aerobic)	99.96	0.49
HF3KT12003AB	ORT-AM05-235	Oral-tongue coat	Male	Adult	Blood-H Medium (Anaerobic)	99.45	0.27
HF3KT12004	ORT-AM05-320	Oral-tongue coat	Male	Adult	H Medium (Aerobic)	99.4	0
HF3KT12004A	ORT-AM05-507	Oral-tongue coat	Male	Adult	H Medium (Anaerobic)	99.84	0.15
HF3KT12004B	ORT-AM05-317	Oral-tongue coat	Male	Adult	Blood-H Medium (Aerobic)	99.96	0.49
HF3KT13001A	ORT-TM06-175	Oral-tongue coat	Male	Teenager	H Medium (Anaerobic)	99.84	2.77
HF3KT13002A	ORT-TM06-176	Oral-tongue coat	Male	Teenager	H Medium (Anaerobic)	99.45	0
HF3KT13003A	ORT-TM06-177	Oral-tongue coat	Male	Teenager	H Medium (Anaerobic)	100	0.47
HF3KT14001	ORT-TM07-185	Oral-tongue coat	Male	Teenager	H Medium (Aerobic)	100	0
HF3KT14001A	ORT-TM07-82	Oral-tongue coat	Male	Teenager	H Medium (Anaerobic)	100	0.59
HF3KT14002B	ORT-TM07-206	Oral-tongue coat	Male	Teenager	Blood-H Medium (Aerobic)	99.33	0.67
HF3KT15001AB	ORT-AM08-461	Oral-tongue coat	Male	Adult	Blood-H Medium (Anaerobic)	99.45	0
HF3KT16001	ORT-AF06-04	Oral-tongue coat	Female	Adult	H Medium (Aerobic)	100	0
HF3KT16004	ORT-AF06-93	Oral-tongue coat	Female	Adult	H Medium (Aerobic)	99.49	0.76
HF3KT16005	ORT-AF06-141	Oral-tongue coat	Female	Adult	H Medium (Aerobic)	99.33	0
IF3KA08003BA	ORS-AF03-170	Oral- saliva	Female	Adult	Blood-MPYG Medium (Anaerobic)	99.82	0.66
IF3KA09001BA	ORS-AF04-147	Oral- saliva	Female	Adult	Blood-MPYG Medium (Anaerobic)	100	0.47
IF3KA09003BA	ORS-AF04-237	Oral- saliva	Female	Adult	Blood-MPYG Medium (Anaerobic)	100	0.47
IF3KA09004A	ORS-AF04-133	Oral- saliva	Female	Adult	MPYG Medium (Anaerobic)	100	0
IF3KA09005A	ORS-AF04-134	Oral- saliva	Female	Adult	MPYG Medium (Anaerobic)	100	0
IF3KA11001	ORS-AM04-35	Oral- saliva	Male	Adult	MPYG Medium (Aerobic)	99.9	0.58
IF3KA12001AB	ORS-AM05-197	Oral- saliva	Male	Adult	Blood-MPYG Medium (Anaerobic)	99.42	0.2
IF3KA12002AB	ORS-AM05-198	Oral- saliva	Male	Adult	Blood-MPYG Medium (Anaerobic)	100	0
IF3KA12002B	ORS-AM05-342	Oral- saliva	Male	Adult	Blood-MPYG Medium (Aerobic)	99.38	1.14
IF3KA12003	ORS-AM05-133	Oral- saliva	Male	Adult	MPYG Medium (Aerobic)	99.62	0.17
IF3KA12003A	ORS-AM05-177	Oral- saliva	Male	Adult	MPYG Medium (Anaerobic)	99	0.09
IF3KA12004AB	ORS-AM05-382	Oral- saliva	Male	Adult	Blood-MPYG Medium (Anaerobic)	100	0.47
IF3KA12007A	ORS-AM05-481	Oral- saliva	Male	Adult	MPYG Medium (Anaerobic)	99.42	0.44
IF3KA12008AB	ORS-AM05-490	Oral- saliva	Male	Adult	Blood-MPYG Medium (Anaerobic)	99.66	0.2
IF3KA12009AB	ORS-AM05-491	Oral- saliva	Male	Adult	Blood-MPYG Medium (Anaerobic)	99.57	0.2
IF3KA13001	ORS-TM06-24	Oral- saliva	Male	Teenager	MPYG Medium (Aerobic)	99.9	0.15
IF3KA13001B	ORS-TM06-04	Oral- saliva	Male	Teenager	Blood-MPYG Medium (Aerobic)	99.11	0
IF3KA13002A	ORS-TM06-104	Oral- saliva	Male	Teenager	MPYG Medium (Anaerobic)	98.46	0
IF3KA13002B	ORS-TM06-05	Oral- saliva	Male	Teenager	Blood-MPYG Medium (Aerobic)	99.88	0.18
IF3KA13003A	ORS-TM06-105	Oral- saliva	Male	Teenager	MPYG Medium (Anaerobic)	99.07	0.2
IF3KA13003B	ORS-TM06-06	Oral- saliva	Male	Teenager	Blood-MPYG Medium (Aerobic)	100	0
IF3KA13004	ORS-TM06-27	Oral- saliva	Male	Teenager	MPYG Medium (Aerobic)	100	0
IF3KA13004A	ORS-TM06-106	Oral- saliva	Male	Teenager	MPYG Medium (Anaerobic)	99.45	0.27
IF3KA13004B	ORS-TM06-07	Oral- saliva	Male	Teenager	Blood-MPYG Medium (Aerobic)	100	0
IF3KA13005A	ORS-TM06-107	Oral- saliva	Male	Teenager	MPYG Medium (Anaerobic)	100	0.47
IF3KA13005B	ORS-TM06-08	Oral- saliva	Male	Teenager	Blood-MPYG Medium (Aerobic)	99.33	0
IF3KA13007A	ORS-TM06-109	Oral- saliva	Male	Teenager	MPYG Medium (Anaerobic)	98.91	0.55
IF3KA13008A	ORS-TM06-110	Oral- saliva	Male	Teenager	MPYG Medium (Anaerobic)	98.37	0
IF3KA13016A	ORS-TM06-231	Oral- saliva	Male	Teenager	MPYG Medium (Anaerobic)	99.66	0.11
IF3KA14004AB	ORS-TM07-51	Oral- saliva	Male	Teenager	Blood-MPYG Medium (Anaerobic)	99.46	0.23
IF3KA14005A	ORS-TM07-18	Oral- saliva	Male	Teenager	MPYG Medium (Anaerobic)	99.66	0
IF3KA14005AB	ORS-TM07-52	Oral- saliva	Male	Teenager	Blood-MPYG Medium (Anaerobic)	99.66	0
IF3KA14005B	ORS-TM07-240	Oral- saliva	Male	Teenager	Blood-MPYG Medium (Aerobic)	99.87	0.24
IF3KA14006A	ORS-TM07-19	Oral- saliva	Male	Teenager	MPYG Medium (Anaerobic)	100	0
IF3KA14006B	ORS-TM07-241	Oral- saliva	Male	Teenager	Blood-MPYG Medium (Aerobic)	99.63	0.31
IF3KA14008A	ORS-TM07-21	Oral- saliva	Male	Teenager	MPYG Medium (Anaerobic)	99.66	0.68
IF3KA15001	ORS-AM08-15	Oral- saliva	Male	Adult	MPYG Medium (Aerobic)	99.79	0.15
IF3KA15001A	ORS-AM08-32	Oral- saliva	Male	Adult	MPYG Medium (Anaerobic)	99.88	0
IF3KA15001AB	ORS-AM08-59	Oral- saliva	Male	Adult	Blood-MPYG Medium (Anaerobic)	99.88	0
IF3KA15002A	ORS-AM08-33	Oral- saliva	Male	Adult	MPYG Medium (Anaerobic)	100	0
IF3KA15002AB	ORS-AM08-60	Oral- saliva	Male	Adult	Blood-MPYG Medium (Anaerobic)	99.66	0.34
IF3KA15003A	ORS-AM08-34	Oral- saliva	Male	Adult	MPYG Medium (Anaerobic)	99.45	0.82
IF3KA15006A	ORS-AM08-153	Oral- saliva	Male	Adult	MPYG Medium (Anaerobic)	100	0.47
IF3KA15007A	ORS-AM08-154	Oral- saliva	Male	Adult	MPYG Medium (Anaerobic)	100	0.59
IF3KA15007B	ORS-AM08-119	Oral- saliva	Male	Adult	Blood-MPYG Medium (Aerobic)	95.75	4.72
IF3KC09001	ODP-AF04-87	Oral-tooth	Female	Adult	MPYG Medium (Aerobic)	99.83	0.58
IF3KC09001A	ODP-AF04-161	Oral-tooth	Female	Adult	MPYG Medium (Anaerobic)	99.45	0
IF3KC09002	ODP-AF04-88	Oral-tooth	Female	Adult	MPYG Medium (Aerobic)	99.82	0.26
IF3KC10005A	ODP-AF05-77	Oral-tooth	Female	Adult	MPYG Medium (Anaerobic)	99.9	1.08
IF3KC12001AB	ODP-AM05-264	Oral-tooth	Male	Adult	Blood-MPYG Medium (Anaerobic)	99.87	0.2
IF3KC12001B	ODP-AM05-72	Oral-tooth	Male	Adult	Blood-MPYG Medium (Aerobic)	99.84	0.15
IF3KC12002	ODP-AM05-34	Oral-tooth	Male	Adult	MPYG Medium (Aerobic)	99.65	0.12
IF3KC12002AB	ODP-AM05-265	Oral-tooth	Male	Adult	Blood-MPYG Medium (Anaerobic)	99.65	0.12
IF3KC12002B	ODP-AM05-73	Oral-tooth	Male	Adult	Blood-MPYG Medium (Aerobic)	100	0
IF3KC12003AB	ODP-AM05-266	Oral-tooth	Male	Adult	Blood-MPYG Medium (Anaerobic)	99	0

IF3KC12004	ODP-AM05-444	Oral-tooth	Male	Adult	MPYG Medium (Aerobic)	98.34	0
IF3KC12004A	ODP-AM05-243	Oral-tooth	Male	Adult	MPYG Medium (Anaerobic)	100	0.12
IF3KC12004AB	ODP-AM05-267	Oral-tooth	Male	Adult	Blood-MPYG Medium (Anaerobic)	99.87	0.2
IF3KC12006A	ODP-AM05-503	Oral-tooth	Male	Adult	MPYG Medium (Anaerobic)	100	0
IF3KC12006AB	ODP-AM05-269	Oral-tooth	Male	Adult	Blood-MPYG Medium (Anaerobic)	100	0.47
IF3KC12007A	ODP-AM05-533	Oral-tooth	Male	Adult	MPYG Medium (Anaerobic)	100	0.47
IF3KC13001A	ODP-TM06-159	Oral-tooth	Male	Teenager	MPYG Medium (Anaerobic)	99.07	0.2
IF3KC13003	ODP-TM06-50	Oral-tooth	Male	Teenager	MPYG Medium (Aerobic)	100	0
IF3KC13006A	ODP-TM06-164	Oral-tooth	Male	Teenager	MPYG Medium (Anaerobic)	100	0
IF3KC14001	ODP-TM07-201	Oral-tooth	Male	Teenager	MPYG Medium (Aerobic)	99.49	0.76
IF3KC14001A	ODP-TM07-131	Oral-tooth	Male	Teenager	MPYG Medium (Anaerobic)	99.45	0
IF3KC14001AB	ODP-TM07-158	Oral-tooth	Male	Teenager	Blood-MPYG Medium (Anaerobic)	100	0
IF3KC14002AB	ODP-TM07-159	Oral-tooth	Male	Teenager	Blood-MPYG Medium (Anaerobic)	99.66	0
IF3KC14004A	ODP-TM07-134	Oral-tooth	Male	Teenager	MPYG Medium (Anaerobic)	100	0
IF3KC14005	ODP-TM07-296	Oral-tooth	Male	Teenager	MPYG Medium (Aerobic)	99.49	0.76
IF3KC14005A	ODP-TM07-173	Oral-tooth	Male	Teenager	MPYG Medium (Anaerobic)	99.49	0.76
IF3KC14005AB	ODP-TM07-162	Oral-tooth	Male	Teenager	Blood-MPYG Medium (Anaerobic)	99.87	0.2
IF3KC14006A	ODP-TM07-271	Oral-tooth	Male	Teenager	MPYG Medium (Anaerobic)	100	0
IF3KC14007A	ODP-TM07-272	Oral-tooth	Male	Teenager	MPYG Medium (Anaerobic)	99.87	0.2
IF3KC15002A	ODP-AM08-308	Oral-tooth	Male	Adult	MPYG Medium (Anaerobic)	100	0
IF3KC15003A	ODP-AM08-396	Oral-tooth	Male	Adult	MPYG Medium (Anaerobic)	98.46	0
IF3KC15005	ODP-AM08-466	Oral-tooth	Male	Adult	MPYG Medium (Aerobic)	99.33	0
IF3KC15005A	ODP-AM08-398	Oral-tooth	Male	Adult	MPYG Medium (Anaerobic)	99.83	0.73
IF3KC16001B	ODP-AF06-62	Oral-tooth	Female	Adult	Blood-MPYG Medium (Aerobic)	99.66	0
IF3KC16002	ODP-AF06-103	Oral-tooth	Female	Adult	MPYG Medium (Aerobic)	99.45	0
IF3KT08003	ORT-AF03-214	Oral-tongue coat	Female	Adult	MPYG Medium (Aerobic)	100	0.17
IF3KT08006BA	ORT-AF03-21	Oral-tongue coat	Female	Adult	Blood-MPYG Medium (Anaerobic)	99.96	0.97
IF3KT09001	ORT-AF04-16	Oral-tongue coat	Female	Adult	MPYG Medium (Aerobic)	100	0.11
IF3KT09001A	ORT-AF04-172	Oral-tongue coat	Female	Adult	MPYG Medium (Anaerobic)	100	0
IF3KT09001BA	ORT-AF04-123	Oral-tongue coat	Female	Adult	Blood-MPYG Medium (Anaerobic)	100	0.47
IF3KT09002	ORT-AF04-193	Oral-tongue coat	Female	Adult	MPYG Medium (Aerobic)	99.83	0.58
IF3KT09003A	ORT-AF04-219	Oral-tongue coat	Female	Adult	MPYG Medium (Anaerobic)	100	0.07
IF3KT09003BA	ORT-AF04-125	Oral-tongue coat	Female	Adult	Blood-MPYG Medium (Anaerobic)	100	0.47
IF3KT10001A	ORT-AF05-117	Oral-tongue coat	Female	Adult	MPYG Medium (Anaerobic)	99.63	0
IF3KT10005A	ORT-AF05-121	Oral-tongue coat	Female	Adult	MPYG Medium (Anaerobic)	99.45	0.27
IF3KT11001A	ORT-AM04-57	Oral-tongue coat	Male	Adult	MPYG Medium (Anaerobic)	99.9	0.58
IF3KT11003A	ORT-AM04-59	Oral-tongue coat	Male	Adult	MPYG Medium (Anaerobic)	99.87	1.26
IF3KT12001	ORT-AM05-07	Oral-tongue coat	Male	Adult	MPYG Medium (Aerobic)	99.84	0.15
IF3KT12002AB	ORT-AM05-499	Oral-tongue coat	Male	Adult	Blood-MPYG Medium (Anaerobic)	99.66	0.2
IF3KT12003A	ORT-AM05-281	Oral-tongue coat	Male	Adult	MPYG Medium (Anaerobic)	99.32	0
IF3KT12003ABC	ORT-AM05-525	Oral-tongue coat	Male	Adult	Blood-MPYG Medium (Anaerobic)	99.87	0.2
IF3KT12004A	ORT-AM05-282	Oral-tongue coat	Male	Adult	MPYG Medium (Anaerobic)	99.87	0.2
IF3KT12004ABC	ORT-AM05-527	Oral-tongue coat	Male	Adult	Blood-MPYG Medium (Anaerobic)	99.87	0.2
IF3KT12009A	ORT-AM05-511	Oral-tongue coat	Male	Adult	MPYG Medium (Anaerobic)	99	0.09
IF3KT12009AC	ORT-AM05-512	Oral-tongue coat	Male	Adult	MPYG Medium (Anaerobic)	99	0.09
IF3KT13002A	ORT-TM06-179	Oral-tongue coat	Male	Teenager	MPYG Medium (Anaerobic)	99.9	0.15
IF3KT13004A	ORT-TM06-181	Oral-tongue coat	Male	Teenager	MPYG Medium (Anaerobic)	100	0
IF3KT13004B	ORT-TM06-80	Oral-tongue coat	Male	Teenager	Blood-MPYG Medium (Aerobic)	99.11	0
IF3KT13012A	ORT-TM06-189	Oral-tongue coat	Male	Teenager	MPYG Medium (Anaerobic)	100	0
IF3KT14001	ORT-TM07-187	Oral-tongue coat	Male	Teenager	MPYG Medium (Aerobic)	99.49	0.76
IF3KT14001A	ORT-TM07-84	Oral-tongue coat	Male	Teenager	MPYG Medium (Anaerobic)	99.9	0.15
IF3KT14001AB	ORT-TM07-100	Oral-tongue coat	Male	Teenager	Blood-MPYG Medium (Anaerobic)	99.46	0.23
IF3KT14002AB	ORT-TM07-101	Oral-tongue coat	Male	Teenager	Blood-MPYG Medium (Anaerobic)	100	0.47
IF3KT14003AB	ORT-TM07-102	Oral-tongue coat	Male	Teenager	Blood-MPYG Medium (Anaerobic)	99.05	4.07
IF3KT14004A	ORT-TM07-87	Oral-tongue coat	Male	Teenager	MPYG Medium (Anaerobic)	100	0.34
IF3KT14006A	ORT-TM07-89	Oral-tongue coat	Male	Teenager	MPYG Medium (Anaerobic)	99.32	0.17
IF3KT14007A	ORT-TM07-90	Oral-tongue coat	Male	Teenager	MPYG Medium (Anaerobic)	100	0
IF3KT14008A	ORT-TM07-91	Oral-tongue coat	Male	Teenager	MPYG Medium (Anaerobic)	100	0.34
IF3KT14008AB	ORT-TM07-107	Oral-tongue coat	Male	Teenager	Blood-MPYG Medium (Anaerobic)	99.66	0
IF3KT14009AB	ORT-TM07-108	Oral-tongue coat	Male	Teenager	Blood-MPYG Medium (Anaerobic)	100	0.34
IF3KT15001	ORT-AM08-202	Oral-tongue coat	Male	Adult	MPYG Medium (Aerobic)	99.79	0.15
IF3KT15002B	ORT-AM08-226	Oral-tongue coat	Male	Adult	Blood-MPYG Medium (Aerobic)	99.34	0
IF3KT15004	ODP-AM08-337	Oral-tooth	Male	Adult	MPYG Medium (Aerobic)	98.56	0
IF3KT15004A	ORT-AM08-425	Oral-tongue coat	Male	Adult	MPYG Medium (Anaerobic)	99.88	0
IF3KT15006	ORT-AM08-480	Oral-tongue coat	Male	Adult	MPYG Medium (Aerobic)	99.33	0
IF3KT15007A	ORT-AM08-428	Oral-tongue coat	Male	Adult	MPYG Medium (Anaerobic)	100	0.59
IF3KT15008AB	ORT-AM08-458	Oral-tongue coat	Male	Adult	Blood-MPYG Medium (Anaerobic)	99.45	0.77
IF3KT15013A	ORT-AM08-434	Oral-tongue coat	Male	Adult	MPYG Medium (Anaerobic)	100	0.17
IF3KT16001B	ORT-AF06-28	Oral-tongue coat	Female	Adult	Blood-MPYG Medium (Aerobic)	100	0.76
IF3KT16003B	ORT-AF06-30	Oral-tongue coat	Female	Adult	Blood-MPYG Medium (Aerobic)	99.66	0.17
IF3KT16004	ORT-AF06-10	Oral-tongue coat	Female	Adult	MPYG Medium (Aerobic)	99.49	0.76
IF3KT16005	ORT-AF06-36	Oral-tongue coat	Female	Adult	MPYG Medium (Aerobic)	100	0
JF3KA10001A	ORS-AF05-30	Oral- saliva	Female	Adult	J Medium (Anaerobic)	99.46	0
JF3KA12003	ORS-AM05-138	Oral- saliva	Male	Adult	J Medium (Aerobic)	99.87	0.7
JF3KA12004B	ORS-AM05-334	Oral- saliva	Male	Adult	Blood-J Medium (Aerobic)	99.96	0.49
JF3KA12006AB	ORS-AM05-362	Oral- saliva	Male	Adult	Blood-J Medium (Anaerobic)	100	0.56
JF3KA12006B	ORS-AM05-474	Oral- saliva	Male	Adult	J Medium (Aerobic)	99.33	0
JF3KA12007AB	ORS-AM05-363	Oral- saliva	Male	Adult	Blood-J Medium (Anaerobic)	99.25	0.12
JF3KA13004	ORS-TM06-31	Oral- saliva	Male	Teenager	J Medium (Aerobic)	99.34	0
JF3KA14001AB	ORS-TM07-54	Oral- saliva	Male	Teenager	Blood-J Medium (Anaerobic)	99.63	0.37
JF3KA15002AB	ORS-AM08-63	Oral- saliva	Male	Adult	Blood-J Medium (Anaerobic)	99.62	0.29
JF3KC08001A	ODP-AF03-102	Oral-tooth	Female	Adult	J Medium (Anaerobic)	99.87	0.2
JF3KC09001	ODP-AF04-91	Oral-tooth	Female	Adult	J Medium (Aerobic)	99.9	1.08
JF3KC09004	ODP-AF04-94	Oral-tooth	Female	Adult	J Medium (Aerobic)	99.82	0.26
JF3KC11001	ODP-AM04-107	Oral-tooth	Male	Adult	J Medium (Aerobic)	99.9	0.58
JF3KC12001A	ODP-AM05-244	Oral-tooth	Male	Adult	J Medium (Anaerobic)	99.65	0.12

JF3KC12002AB	ODP-AM05-271	Oral-tooth	Male	Adult	Blood-J Medium (Anaerobic)	99.45	0
JF3KC12002B	ODP-AM05-303	Oral-tooth	Male	Adult	Blood-J Medium (Aerobic)	99.67	0.23
JF3KC12003AB	ODP-AM05-272	Oral-tooth	Male	Adult	Blood-J Medium (Anaerobic)	99.45	0
JF3KC12004AB	ODP-AM05-391	Oral-tooth	Male	Adult	Blood-J Medium (Anaerobic)	100	0.95
JF3KC12005A	ODP-AM05-504	Oral-tooth	Male	Adult	J Medium (Anaerobic)	99.82	0.6
JF3KC14002B	ODP-TM07-321	Oral-tooth	Male	Teenager	Blood-J Medium (Aerobic)	99.49	0.76
JF3KC16002	ODP-AF06-99	Oral-tooth	Female	Adult	J Medium (Aerobic)	99.42	0.2
JF3KC16004	ODP-AF06-105	Oral-tooth	Female	Adult	J Medium (Aerobic)	99.49	0.76
JF3KT08002A	ORT-AF03-133	Oral-tongue coat	Female	Adult	J Medium (Anaerobic)	100	0.56
JF3KT08002BA	ORT-AF03-24	Oral-tongue coat	Female	Adult	Blood-J Medium (Anaerobic)	99.61	1.56
JF3KT09001A	ORT-AF04-174	Oral-tongue coat	Female	Adult	J Medium (Anaerobic)	100	0.47
JF3KT09003	ORT-AF04-19	Oral-tongue coat	Female	Adult	J Medium (Aerobic)	99.76	0.83
JF3KT09003A	ORT-AF04-225	Oral-tongue coat	Female	Adult	J Medium (Anaerobic)	100	0.47
JF3KT10001	ORT-AF05-174	Oral-tongue coat	Female	Adult	J Medium (Aerobic)	99.11	0
JF3KT10001A	ORT-AF05-123	Oral-tongue coat	Female	Adult	J Medium (Anaerobic)	99.63	0
JF3KT12003B	ORT-AM05-319	Oral-tongue coat	Male	Adult	Blood-J Medium (Aerobic)	99.97	0.71
JF3KT12005AB	ORT-AM05-529	Oral-tongue coat	Male	Adult	Blood-J Medium (Anaerobic)	100	0.73
JF3KT12007A	ORT-AM05-514	Oral-tongue coat	Male	Adult	J Medium (Anaerobic)	99.4	0.58
JF3KT14002	ORT-TM07-192	Oral-tongue coat	Male	Teenager	J Medium (Aerobic)	99.33	0
JF3KT15001	ORT-AM08-205	Oral-tongue coat	Male	Adult	J Medium (Aerobic)	99.33	0
JF3KT15001A	ORT-AM08-438	Oral-tongue coat	Male	Adult	J Medium (Anaerobic)	99.46	0.15
JF3KT15002	ORT-AM08-206	Oral-tongue coat	Male	Adult	J Medium (Aerobic)	99.66	0.42
JF3KT15002B	ODP-AM08-363	Oral-tooth	Male	Adult	Blood-J Medium (Aerobic)	99.33	0
MF3KA09001A	ORS-AF04-142	Oral- saliva	Female	Adult	MRS Medium (Anaerobic)	99.87	1.26
MF3KA09002A	ORS-AF04-143	Oral- saliva	Female	Adult	MRS Medium (Anaerobic)	100	0.34
MF3KA09005A	ORS-AF04-236	Oral- saliva	Female	Adult	MRS Medium (Anaerobic)	100	0
MF3KA11001	ORS-AM04-111	Oral- saliva	Male	Adult	MRS Medium (Aerobic)	99.45	0.27
MF3KA11001A	ORS-AM04-88	Oral- saliva	Male	Adult	MRS Medium (Anaerobic)	100	0.47
MF3KA11003A	ORS-AM04-90	Oral- saliva	Male	Adult	MRS Medium (Anaerobic)	100	0
MF3KA12001B	ORS-AM05-336	Oral- saliva	Male	Adult	Blood-J Medium (Anaerobic)	99.38	1.14
MF3KA12002	ORS-AM05-140	Oral- saliva	Male	Adult	MRS Medium (Aerobic)	100	0.34
MF3KA12002AB	ORS-AM05-205	Oral- saliva	Male	Adult	Blood-MRS Medium (Anaerobic)	100	0.71
MF3KA12002B	ORS-AM05-475	Oral- saliva	Male	Adult	MRS Medium (Aerobic)	98.95	0
MF3KA12003AB	ORS-AM05-364	Oral- saliva	Male	Adult	Blood-MRS Medium (Anaerobic)	100	0.48
MF3KA12004AB	ORS-AM05-365	Oral- saliva	Male	Adult	Blood-MRS Medium (Anaerobic)	99.32	0.17
MF3KA12007AB	ORS-AM05-494	Oral- saliva	Male	Adult	Blood-MRS Medium (Anaerobic)	99.94	0.28
MF3KA12008AB	ORS-AM05-495	Oral- saliva	Male	Adult	Blood-MRS Medium (Anaerobic)	99	0
MF3KA13001A	ORS-TM06-111	Oral- saliva	Male	Teenager	MRS Medium (Anaerobic)	99.53	0.09
MF3KA13001AB	ORS-TM06-118	Oral- saliva	Male	Teenager	Blood-MRS Medium (Anaerobic)	99.53	0.09
MF3KA13002A	ORS-TM06-112	Oral- saliva	Male	Teenager	MRS Medium (Anaerobic)	100	0.76
MF3KA13003A	ORS-TM06-113	Oral- saliva	Male	Teenager	MRS Medium (Anaerobic)	99.62	0
MF3KA13004A	ORS-TM06-114	Oral- saliva	Male	Teenager	MRS Medium (Anaerobic)	98.37	0
MF3KA13014A	ORS-TM06-235	Oral- saliva	Male	Teenager	MRS Medium (Anaerobic)	100	0
MF3KA14001	ORS-TM07-224	Oral- saliva	Male	Teenager	MRS Medium (Aerobic)	99.46	0.23
MF3KA14002	ORS-TM07-225	Oral- saliva	Male	Teenager	MRS Medium (Aerobic)	99.82	0.2
MF3KA14006B	ORS-TM07-255	Oral- saliva	Male	Teenager	Blood-MRS Medium (Aerobic)	98.91	0.55
MF3KA15001AB	ORS-AM08-69	Oral- saliva	Male	Adult	Blood-MRS Medium (Anaerobic)	99.45	0.82
MF3KA15005B	ORS-AM08-136	Oral- saliva	Male	Adult	Blood-MRS Medium (Aerobic)	99.33	0
MF3KC09001	ODP-AF04-99	Oral-tooth	Female	Adult	MRS Medium (Aerobic)	99.9	1.08
MF3KC09001B	ODP-AF04-114	Oral-tooth	Female	Adult	Blood-BHI (Aerobic)	99.46	0
MF3KC09002A	ODP-AF04-250	Oral-tooth	Female	Adult	MRS Medium (Anaerobic)	99.88	0
MF3KC11001A	ODP-AM04-44	Oral-tooth	Male	Adult	MRS Medium (Anaerobic)	99.84	0.58
MF3KC11002A	ODP-AM04-45	Oral-tooth	Male	Adult	MRS Medium (Anaerobic)	100	0.37
MF3KC12001A	ODP-AM05-247	Oral-tooth	Male	Adult	MRS Medium (Anaerobic)	100	0.12
MF3KC12001AB	ODP-AM05-273	Oral-tooth	Male	Adult	Blood-MRS Medium (Anaerobic)	99.65	0.12
MF3KC12001B	ODP-AM05-76	Oral-tooth	Male	Adult	Blood-MRS Medium (Aerobic)	99.34	0
MF3KC12002A	ODP-AM05-248	Oral-tooth	Male	Adult	MRS Medium (Anaerobic)	99.65	0.12
MF3KC12002AB	ODP-AM05-535	Oral-tooth	Male	Adult	Blood-MRS Medium (Anaerobic)	99.87	0.2
MF3KC12003	ODP-AM05-41	Oral-tooth	Male	Adult	MRS Medium (Aerobic)	99.65	0.12
MF3KC12003A	ODP-AM05-249	Oral-tooth	Male	Adult	MRS Medium (Anaerobic)	99.45	0
MF3KC12003B	ODP-AM05-78	Oral-tooth	Male	Adult	Blood-MRS Medium (Aerobic)	100	0
MF3KC12004A	ODP-AM05-429	Oral-tooth	Male	Adult	MRS Medium (Anaerobic)	99.84	0.15
MF3KC12006B	ODP-AM05-440	Oral-tooth	Male	Adult	Blood-MRS Medium (Aerobic)	99.78	0
MF3KC13003	ODP-TM06-55	Oral-tooth	Male	Teenager	MRS Medium (Aerobic)	100	0
MF3KC15001A	ODP-AM08-309	Oral-tooth	Male	Adult	MRS Medium (Anaerobic)	100	0
MF3KC15002A	ODP-AM08-310	Oral-tooth	Male	Adult	MRS Medium (Anaerobic)	99.45	0
MF3KC15010AB	ODP-AM08-335	Oral-tooth	Male	Adult	Blood-MRS Medium (Anaerobic)	99.45	0
MF3KT08001	ORT-AF03-215	Oral-tongue coat	Female	Adult	MRS Medium (Aerobic)	99.96	0.76
MF3KT08001A	ORT-AF03-01	Oral-tongue coat	Female	Adult	MRS Medium (Anaerobic)	100	0
MF3KT08004A	ORT-AF03-04	Oral-tongue coat	Female	Adult	MRS Medium (Anaerobic)	100	0
MF3KT08008A	ORT-AF03-08	Oral-tongue coat	Female	Adult	MRS Medium (Anaerobic)	100	0.08
MF3KT09001	ORT-AF04-23	Oral-tongue coat	Female	Adult	MRS Medium (Aerobic)	99.83	0.58
MF3KT09001A	ORT-AF04-222	Oral-tongue coat	Female	Adult	MRS Medium (Anaerobic)	100	0
MF3KT09003A	ORT-AF04-247	Oral-tongue coat	Female	Adult	MRS Medium (Anaerobic)	99.32	0.42
MF3KT10001A	ORT-AF05-128	Oral-tongue coat	Female	Adult	MRS Medium (Anaerobic)	99.63	0
MF3KT10005A	ORT-AF05-132	Oral-tongue coat	Female	Adult	MRS Medium (Anaerobic)	100	0
MF3KT10008A	ORT-AF05-232	Oral-tongue coat	Female	Adult	MRS Medium (Anaerobic)	100	0.47
MF3KT11002A	ORT-AM04-65	Oral-tongue coat	Male	Adult	MRS Medium (Anaerobic)	99.83	0.58
MF3KT12001A	ORT-AM05-287	Oral-tongue coat	Male	Adult	MRS Medium (Anaerobic)	99.87	0.3
MF3KT12001AB	ORT-AM05-101	Oral-tongue coat	Male	Adult	Blood-MRS Medium (Anaerobic)	99.45	0.46
MF3KT12002A	ORT-AM05-515	Oral-tongue coat	Male	Adult	MRS Medium (Anaerobic)	99.9	0.15
MF3KT12002AB	ORT-AM05-102	Oral-tongue coat	Male	Adult	Blood-MRS Medium (Anaerobic)	99.87	0.3
MF3KT12003A	ORT-AM05-521	Oral-tongue coat	Male	Adult	MRS Medium (Anaerobic)	99.87	0.2
MF3KT12004AB	ORT-AM05-104	Oral-tongue coat	Male	Adult	Blood-MRS Medium (Anaerobic)	99.87	0.3
MF3KT12004B	ORT-AM05-63	Oral-tongue coat	Male	Adult	Blood-MRS Medium (Aerobic)	99.4	0.68
MF3KT12008B	ORT-AM05-310	Oral-tongue coat	Male	Adult	Blood-MRS Medium (Aerobic)	99.33	0

MF3KT13001A	ORT-TM06-193	Oral-tongue coat	Male	Teenager	MRS Medium (Anaerobic)	99.32	0.4
MF3KT13002A	ORT-TM06-194	Oral-tongue coat	Male	Teenager	MRS Medium (Anaerobic)	99.66	0.17
MF3KT13003A	ORT-TM06-195	Oral-tongue coat	Male	Teenager	MRS Medium (Anaerobic)	100	0
MF3KT13008A	ORT-TM06-200	Oral-tongue coat	Male	Teenager	MRS Medium (Anaerobic)	98.56	0
MF3KT13011A	ORT-TM06-203	Oral-tongue coat	Male	Teenager	MRS Medium (Anaerobic)	100	0.51
MF3KT14001B	ORT-TM07-207	Oral-tongue coat	Male	Teenager	Blood-MRS Medium (Aerobic)	99.46	0.23
MF3KT15001A	ORT-AM08-450	Oral-tongue coat	Male	Adult	MRS Medium (Anaerobic)	99.88	0
MF3KT15001AB	ORT-AM08-463	Oral-tongue coat	Male	Adult	Blood-MRS Medium (Anaerobic)	99.88	0
MF3KT15001B	ORT-AM08-229	Oral-tongue coat	Male	Adult	Blood-MRS Medium (Aerobic)	100	0
MF3KT15004	ORT-AM08-210	Oral-tongue coat	Male	Adult	MRS Medium (Aerobic)	100	0
MF3KT16001	ORT-AF06-11	Oral-tongue coat	Female	Adult	MRS Medium (Aerobic)	99.49	0.76
MF3KT16003B	ORT-AF06-97	Oral-tongue coat	Female	Adult	Blood-MRS Medium (Aerobic)	99.33	0
ODP-AM01-02BB	ODP-AM01-02BB	Oral-tooth	Male	Adult	Blood-BHI (Anaerobic)	99.87	0.2
ODP-AM01-02O	ODP-AM01-02O	Oral-tooth	Male	Adult	MPYG Medium (Aerobic)	99.92	0.23
ODP-AM01-04	ODP-AM01-04	Oral-tooth	Male	Adult	MPYG Medium (Anaerobic)	99.63	0.75
ODP-AM01-04MBO	ODP-AM01-04MBO	Oral-tooth	Male	Adult	MRS Medium (Aerobic)	99.56	0.15
ODP-AM01-05	ODP-AM01-05	Oral-tooth	Male	Adult	MPYG Medium (Anaerobic)	98.56	0
ODP-AM01-05BB	ODP-AM01-05BB	Oral-tooth	Male	Adult	Blood-BHI (Anaerobic)	100	0.47
ODP-AM01-06	ODP-AM01-06	Oral-tooth	Male	Adult	MPYG Medium (Anaerobic)	100	0
ODP-AM01-08	ODP-AM01-08	Oral-tooth	Male	Adult	MPYG Medium (Anaerobic)	99.87	0.2
ODP-AM01-10	ODP-AM01-10	Oral-tooth	Male	Adult	MPYG Medium (Anaerobic)	98.88	0
ORS-AM01-01	ORS-AM01-01	Oral- saliva	Male	Adult	MPYG Medium (Anaerobic)	99.56	2.33
ORS-AM01-01BB	ORS-AM01-01BB	Oral- saliva	Male	Adult	Blood-BHI (Anaerobic)	100	0.47
ORS-AM01-01O	ORS-AM01-01O	Oral- saliva	Male	Adult	MPYG Medium (Aerobic)	99.92	0.45
ORS-AM01-02	ORS-AM01-02	Oral- saliva	Male	Adult	MPYG Medium (Anaerobic)	100	0.56
ORS-AM01-02BB	ORS-AM01-02BB	Oral- saliva	Male	Adult	Blood-BHI (Anaerobic)	99.66	0.07
ORS-AM01-02BBO	ORS-AM01-02BBO	Oral- saliva	Male	Adult	MRS Medium (Aerobic)	100	0.47
ORS-AM01-02MB	ORS-AM01-02MB	Oral- saliva	Male	Adult	MRS Medium (Anaerobic)	100	0.37
ORS-AM01-03BB	ORS-AM01-03BB	Oral- saliva	Male	Adult	Blood-BHI (Anaerobic)	99.66	0.11
ORS-AM01-04BBO	ORS-AM01-04BBO	Oral- saliva	Male	Adult	Blood-BHI (Aerobic)	99.64	0.11
ORS-AM01-04MB	ORS-AM01-04MB	Oral- saliva	Male	Adult	MRS Medium (Anaerobic)	99	0
ORS-AM01-13TBO	ORS-AM01-13TBO	Oral- saliva	Male	Adult	TSB Medium (Aerobic)	99.92	0
ORT-AM01-01TBO	ORT-AM01-01TBO	Oral-tongue coat	Male	Adult	TSB Medium (Aerobic)	99.01	0.81
ORT-AM01-02	ORT-AM01-02	Oral-tongue coat	Male	Adult	MPYG Medium (Anaerobic)	96.5	0
ORT-AM01-03O	ORT-AM01-03O	Oral-tongue coat	Male	Adult	MPYG Medium (Aerobic)	99.33	0
ORT-AM01-05MB	ORT-AM01-05MB	Oral-tongue coat	Male	Adult	MRS Medium (Anaerobic)	99	0
ORT-AM01-06O	ORT-AM01-06O	Oral-tongue coat	Male	Adult	MPYG Medium (Aerobic)	99.45	0.27
ORT-AM01-08MB	ORT-AM01-08MB	Oral-tongue coat	Male	Adult	MRS Medium (Anaerobic)	100	0
ORT-AM01-12O	ORT-AM01-12O	Oral-tongue coat	Male	Adult	MPYG Medium (Aerobic)	99.43	0.03
PF3KA08003A	ORS-AF03-153	Oral- saliva	Female	Adult	Spore Medium (Anaerobic)	99.61	1.56
PF3KA08005A	ORS-AF03-155	Oral- saliva	Female	Adult	Spore Medium (Anaerobic)	99.82	0.24
PF3KA09001A	ORS-AF04-146	Oral- saliva	Female	Adult	Spore Medium (Anaerobic)	99.83	0.58
PF3KA10004A	ORS-AF05-45	Oral- saliva	Female	Adult	Spore Medium (Anaerobic)	100	0
PF3KA11001A	ORS-AM04-42	Oral- saliva	Male	Adult	Spore Medium (Anaerobic)	100	0.47
PF3KA12001	ORS-AM05-141	Oral- saliva	Male	Adult	Spore Medium (Aerobic)	99.87	0.3
PF3KA12002AB	ORS-AM05-207	Oral- saliva	Male	Adult	Blood-Spore Medium (Anaerobic)	100	0
PF3KA12003AB	ORS-AM05-208	Oral- saliva	Male	Adult	Blood-Spore Medium (Anaerobic)	100	1.69
PF3KA12005B	ORS-AM05-477	Oral- saliva	Male	Adult	Spore Medium (Aerobic)	99.33	0
PF3KA12007	ORS-AM05-147	Oral- saliva	Male	Adult	Spore Medium (Aerobic)	99.34	0
PF3KA13002	ORS-TM06-36	Oral- saliva	Male	Teenager	Spore Medium (Aerobic)	99.87	0.2
PF3KA13004B	ORS-TM06-246	Oral- saliva	Male	Teenager	Blood-Spore Medium (Aerobic)	99.34	0
PF3KA14002	ORS-TM07-227	Oral- saliva	Male	Teenager	Spore Medium (Aerobic)	99.87	0.27
PF3KA14002AB	ORS-TM07-61	Oral- saliva	Male	Teenager	Blood-Spore Medium (Anaerobic)	99.46	0.23
PF3KA14003B	ORS-TM07-262	Oral- saliva	Male	Teenager	Blood-Spore Medium (Aerobic)	99.49	0.76
PF3KA14005AB	ORS-TM07-64	Oral- saliva	Male	Teenager	Blood-Spore Medium (Anaerobic)	100	0.59
PF3KA14007AB	ORS-TM07-66	Oral- saliva	Male	Teenager	Blood-Spore Medium (Anaerobic)	100	0.59
PF3KA14014AB	ORS-TM07-73	Oral- saliva	Male	Teenager	Blood-Spore Medium (Anaerobic)	99.63	0
PF3KA15001A	ORS-AM08-42	Oral- saliva	Male	Adult	Spore Medium (Anaerobic)	100	0.45
PF3KA15002A	ORS-AM08-43	Oral- saliva	Male	Adult	Spore Medium (Anaerobic)	100	0
PF3KA15003A	ORS-AM08-44	Oral- saliva	Male	Adult	Spore Medium (Anaerobic)	99.45	0
PF3KA15006AB	ORS-AM08-178	Oral- saliva	Male	Adult	Blood-Spore Medium (Anaerobic)	100	0.47
PF3KC08001A	ODP-AF03-106	Oral-tooth	Female	Adult	Spore Medium (Anaerobic)	99.63	0
PF3KC08007BA	ODP-AF03-248	Oral-tooth	Female	Adult	Blood-Spore Medium (Anaerobic)	99.59	0
PF3KC09001	ODP-AF04-102	Oral-tooth	Female	Adult	Spore Medium (Aerobic)	99.83	0.58
PF3KC09001A	ODP-AF04-243	Oral-tooth	Female	Adult	Spore Medium (Anaerobic)	100	0
PF3KC09002	ODP-AF04-103	Oral-tooth	Female	Adult	Spore Medium (Aerobic)	99.9	1.08
PF3KC09002A	ODP-AF04-244	Oral-tooth	Female	Adult	Spore Medium (Anaerobic)	99.45	0
PF3KC09004	ODP-AF04-105	Oral-tooth	Female	Adult	Spore Medium (Aerobic)	98.28	0.14
PF3KC09005	ODP-AF04-106	Oral-tooth	Female	Adult	Spore Medium (Aerobic)	99.83	0.58
PF3KC10001A	ODP-AF05-85	Oral-tooth	Female	Adult	Spore Medium (Anaerobic)	100	0.47
PF3KC11001A	ODP-AM04-46	Oral-tooth	Male	Adult	Spore Medium (Anaerobic)	99.9	0.58
PF3KC12001	ODP-AM05-43	Oral-tooth	Male	Adult	Spore Medium (Aerobic)	99.34	0
PF3KC12001B	ODP-AM05-80	Oral-tooth	Male	Adult	Blood-Spore Medium (Aerobic)	99.87	0.46
PF3KC12002A	ODP-AM05-432	Oral-tooth	Male	Adult	Spore Medium (Anaerobic)	100	0
PF3KC12003B	ODP-AM05-115	Oral-tooth	Male	Adult	Blood-Spore Medium (Aerobic)	99.33	0
PF3KC12004A	ODP-AM05-434	Oral-tooth	Male	Adult	Spore Medium (Anaerobic)	100	0
PF3KC12005A	ODP-AM05-506	Oral-tooth	Male	Adult	Spore Medium (Anaerobic)	99.82	0.54
PF3KC15001B	ODP-AM08-275	Oral-tooth	Male	Adult	Blood-Spore Medium (Aerobic)	95.35	0.97
PF3KC16003	ODP-AF06-153	Oral-tooth	Female	Adult	Spore Medium (Aerobic)	99.49	0.76
PF3KT08008BA	ORT-AF03-41	Oral-tongue coat	Female	Adult	Blood-Spore Medium (Anaerobic)	100	0.47
PF3KT09001	ORT-AF04-26	Oral-tongue coat	Female	Adult	Spore Medium (Aerobic)	99.83	0.73
PF3KT09003A	ORT-AF04-176	Oral-tongue coat	Female	Adult	Spore Medium (Anaerobic)	99.32	0
PF3KT09004A	ORT-AF04-177	Oral-tongue coat	Female	Adult	Spore Medium (Anaerobic)	99.32	0
PF3KT09005A	ORT-AF04-227	Oral-tongue coat	Female	Adult	Spore Medium (Anaerobic)	100	0.07
PF3KT10003A	ORT-AF05-233	Oral-tongue coat	Female	Adult	Spore Medium (Anaerobic)	100	0
PF3KT12001	ORT-AM05-13	Oral-tongue coat	Male	Adult	Spore Medium (Aerobic)	99.87	0.3

PF3KT12001AB	ORT-AM05-105	Oral-tongue coat	Male	Adult	Blood-Spore Medium (Anaerobic)	99.45	0.27
PF3KT12002AB	ORT-AM05-106	Oral-tongue coat	Male	Adult	Blood-Spore Medium (Anaerobic)	99.94	0.28
PF3KT12003AB	ORT-AM05-500	Oral-tongue coat	Male	Adult	Blood-Spore Medium (Anaerobic)	99.87	0.2
PF3KT12003B	ORT-AM05-112	Oral-tongue coat	Male	Adult	Blood-Spore Medium (Aerobic)	100	0
PF3KT12005B	ORT-AM05-304	Oral-tongue coat	Male	Adult	Blood-Spore Medium (Aerobic)	99.33	0
PF3KT12006A	ORT-AM05-451	Oral-tongue coat	Male	Adult	Spore Medium (Aerobic)	100	0.34
PF3KT14001	ORT-TM07-195	Oral-tongue coat	Male	Teenager	Spore Medium (Aerobic)	99.49	0.76
PF3KT14001A	ORT-TM07-93	Oral-tongue coat	Male	Teenager	Spore Medium (Anaerobic)	99.46	0.23
PF3KT14001AB	ORT-TM07-110	Oral-tongue coat	Male	Teenager	Blood-Spore Medium (Anaerobic)	100	0.59
PF3KT14001B	ORT-TM07-310	Oral-tongue coat	Male	Teenager	Blood-Spore Medium (Aerobic)	99.49	0.76
PF3KT14002A	ORT-TM07-94	Oral-tongue coat	Male	Teenager	Spore Medium (Anaerobic)	99.45	0.46
PF3KT14003AB	ORT-TM07-112	Oral-tongue coat	Male	Teenager	Blood-Spore Medium (Anaerobic)	99.46	1.98
PF3KT15003	ORT-AM08-214	Oral-tongue coat	Male	Adult	Spore Medium (Aerobic)	100	0
PF3KT16002	ORT-AF06-14	Oral-tongue coat	Female	Adult	Spore Medium (Aerobic)	99.49	0.76
PF3KT16002B	ORT-AF06-148	Oral-tongue coat	Female	Adult	Blood-Spore Medium (Aerobic)	99.33	0
SF3KA08003A	ORS-AF03-239	Oral- saliva	Female	Adult	SCH Medium (Anaerobic)	100	0
SF3KA09001	ORS-AF04-52	Oral- saliva	Female	Adult	SCH Medium (Aerobic)	99.34	0
SF3KA09003	ORS-AF04-54	Oral- saliva	Female	Adult	SCH Medium (Aerobic)	99.83	0.2
SF3KA09008	ORS-AF04-59	Oral- saliva	Female	Adult	SCH Medium (Aerobic)	99.83	0.4
SF3KA12006A	ORS-AM05-380	Oral- saliva	Male	Adult	SCH Medium (Anaerobic)	99.05	0
SF3KA12009AB	ORS-AM05-383	Oral- saliva	Male	Adult	Blood-SCH Medium (Anaerobic)	100	0.95
SF3KA12011AB	ORS-AM05-471	Oral- saliva	Male	Adult	SCH Medium (Aerobic)	100	0.71
SF3KA13001	ORS-TM06-37	Oral- saliva	Male	Teenager	SCH Medium (Aerobic)	99.92	0
SF3KA13001A	ORS-TM06-130	Oral- saliva	Male	Teenager	SCH Medium (Anaerobic)	100	0
SF3KA13003	ORS-TM06-39	Oral- saliva	Male	Teenager	SCH Medium (Aerobic)	99.95	0.51
SF3KA13005A	ORS-TM06-134	Oral- saliva	Male	Teenager	SCH Medium (Anaerobic)	99.45	0
SF3KA13011A	ORS-TM06-140	Oral- saliva	Male	Teenager	SCH Medium (Anaerobic)	100	0
SF3KA13014A	ORS-TM06-237	Oral- saliva	Male	Teenager	SCH Medium (Anaerobic)	99.66	0
SF3KA14003AB	ORS-TM07-76	Oral- saliva	Male	Teenager	Blood-SCH Medium (Anaerobic)	98.46	0
SF3KA14004A	ORS-TM07-35	Oral- saliva	Male	Teenager	SCH Medium (Anaerobic)	99.63	0
SF3KA14004AB	ORS-TM07-77	Oral- saliva	Male	Teenager	Blood-SCH Medium (Anaerobic)	98.46	0
SF3KA14006AB	ORS-TM07-79	Oral- saliva	Male	Teenager	Blood-SCH Medium (Anaerobic)	98.46	0
SF3KA14007B	ORS-TM07-269	Oral- saliva	Male	Teenager	Blood-SCH Medium (Aerobic)	99.87	0.24
SF3KA14008B	ORS-TM07-270	Oral- saliva	Male	Teenager	Blood-SCH Medium (Aerobic)	99.45	0
SF3KA15001A	ORS-AM08-46	Oral- saliva	Male	Adult	SCH Medium (Anaerobic)	99.45	0.27
SF3KA15002AB	ORS-AM08-79	Oral- saliva	Male	Adult	Blood-SCH Medium (Anaerobic)	100	0
SF3KA15003A	ORS-AM08-48	Oral- saliva	Male	Adult	SCH Medium (Anaerobic)	99.45	0.27
SF3KA15003AB	ORS-AM08-180	Oral- saliva	Male	Adult	Blood-SCH Medium (Anaerobic)	99.32	0
SF3KA15004A	ORS-AM08-49	Oral- saliva	Male	Adult	SCH Medium (Anaerobic)	100	0.17
SF3KA15005A	ORS-AM08-163	Oral- saliva	Male	Adult	SCH Medium (Anaerobic)	100	0
SF3KA16005	ORS-AF06-137	Oral- saliva	Female	Adult	SCH Medium (Aerobic)	99.33	0
SF3KC09001	ODP-AF04-107	Oral-tooth	Female	Adult	SCH Medium (Aerobic)	99.83	0.58
SF3KC09001A	ODP-AF04-166	Oral-tooth	Female	Adult	SCH Medium (Anaerobic)	100	0
SF3KC09002	ODP-AF04-108	Oral-tooth	Female	Adult	SCH Medium (Aerobic)	99.83	0.58
SF3KC10002	ODP-AF05-197	Oral-tooth	Female	Adult	SCH Medium (Aerobic)	99.34	0
SF3KC10002A	ODP-AF05-91	Oral-tooth	Female	Adult	SCH Medium (Anaerobic)	100	0.47
SF3KC10006A	ODP-AF05-95	Oral-tooth	Female	Adult	SCH Medium (Anaerobic)	100	0
SF3KC10008	ODP-AF05-203	Oral-tooth	Female	Adult	SCH Medium (Aerobic)	100	0.07
SF3KC11001A	ODP-AM04-51	Oral-tooth	Male	Adult	SCH Medium (Anaerobic)	98.56	0
SF3KC11002	ODP-AM04-150	Oral-tooth	Male	Adult	SCH Medium (Aerobic)	99.33	0
SF3KC11002A	ODP-AM04-52	Oral-tooth	Male	Adult	SCH Medium (Anaerobic)	99.45	0.18
SF3KC11004A	ODP-AM04-126	Oral-tooth	Male	Adult	SCH Medium (Anaerobic)	99.92	0.45
SF3KC12001	ODP-AM05-46	Oral-tooth	Male	Adult	SCH Medium (Aerobic)	99.7	0.23
SF3KC12002A	ODP-AM05-252	Oral-tooth	Male	Adult	SCH Medium (Anaerobic)	99.42	0.44
SF3KC12002AB	ODP-AM05-394	Oral-tooth	Male	Adult	Blood-SCH Medium (Anaerobic)	99.63	0.12
SF3KC12003AB	ODP-AM05-395	Oral-tooth	Male	Adult	Blood-SCH Medium (Anaerobic)	99	0.09
SF3KC12004	ODP-AM05-49	Oral-tooth	Male	Adult	SCH Medium (Aerobic)	99.87	0.3
SF3KC12004AB	ODP-AM05-396	Oral-tooth	Male	Adult	Blood-SCH Medium (Anaerobic)	100	0.16
SF3KC12005AB	ODP-AM05-464	Oral-tooth	Male	Adult	SCH Medium (Aerobic)	100	1.14
SF3KC13002A	ODP-TM06-166	Oral-tooth	Male	Teenager	SCH Medium (Anaerobic)	100	0.47
SF3KC13003A	ODP-TM06-167	Oral-tooth	Male	Teenager	SCH Medium (Anaerobic)	100	0.47
SF3KC14001B	ODP-TM07-212	Oral-tooth	Male	Teenager	Blood-SCH Medium (Aerobic)	99.87	0.24
SF3KC15002	ODP-AM08-250	Oral-tooth	Male	Adult	SCH Medium (Aerobic)	99.34	0
SF3KC15004A	ODP-AM08-316	Oral-tooth	Male	Adult	SCH Medium (Anaerobic)	98.46	0
SF3KC15013A	ODP-AM08-325	Oral-tooth	Male	Adult	SCH Medium (Anaerobic)	99.45	0
SF3KC16006	ODP-AF06-107	Oral-tooth	Female	Adult	SCH Medium (Aerobic)	100	0
SF3KT08005A	ORT-AF03-190	Oral-tongue coat	Female	Adult	SCH Medium (Anaerobic)	100	0
SF3KT09001	ORT-AF04-29	Oral-tongue coat	Female	Adult	SCH Medium (Aerobic)	99.76	0.83
SF3KT09001A	ORT-AF04-121	Oral-tongue coat	Female	Adult	SCH Medium (Anaerobic)	99.9	1.08
SF3KT09002	ORT-AF04-30	Oral-tongue coat	Female	Adult	SCH Medium (Aerobic)	99.83	0.58
SF3KT09003A	ORT-AF04-228	Oral-tongue coat	Female	Adult	SCH Medium (Anaerobic)	99.76	0.83
SF3KT09004A	ORT-AF04-248	Oral-tongue coat	Female	Adult	SCH Medium (Anaerobic)	99.45	0.55
SF3KT10004A	ORT-AF05-139	Oral-tongue coat	Female	Adult	SCH Medium (Anaerobic)	100	0
SF3KT10008A	ORT-AF05-234	Oral-tongue coat	Female	Adult	SCH Medium (Anaerobic)	100	0.47
SF3KT11001	ORT-AM04-26	Oral-tongue coat	Male	Adult	SCH Medium (Aerobic)	99.33	0
SF3KT11002A	ORT-AM04-73	Oral-tongue coat	Male	Adult	SCH Medium (Anaerobic)	99.45	0
SF3KT11005A	ORT-AM04-81	Oral-tongue coat	Male	Adult	SCH Medium (Anaerobic)	99.45	0
SF3KT12001AB	ORT-AM05-107	Oral-tongue coat	Male	Adult	Blood-SCH Medium (Anaerobic)	99.45	0.27
SF3KT12001B	ORT-AM05-308	Oral-tongue coat	Male	Adult	Blood-SCH Medium (Aerobic)	99.33	0
SF3KT12002A	ORT-AM05-404	Oral-tongue coat	Male	Adult	SCH Medium (Anaerobic)	100	0
SF3KT12002AB	ORT-AM05-108	Oral-tongue coat	Male	Adult	Blood-SCH Medium (Anaerobic)	99.94	0.28
SF3KT12003AB	ORT-AM05-109	Oral-tongue coat	Male	Adult	Blood-SCH Medium (Anaerobic)	99.45	0.27
SF3KT13001A	ORT-TM06-205	Oral-tongue coat	Male	Teenager	SCH Medium (Anaerobic)	99.84	0.15
SF3KT13003A	ORT-TM06-207	Oral-tongue coat	Male	Teenager	SCH Medium (Anaerobic)	99.32	0
SF3KT13004A	ORT-TM06-208	Oral-tongue coat	Male	Teenager	SCH Medium (Anaerobic)	100	0
SF3KT13006A	ORT-TM06-210	Oral-tongue coat	Male	Teenager	SCH Medium (Anaerobic)	99.32	0

SF3KT13007A	ORT-TM06-211	Oral-tongue coat	Male	Teenager	SCH Medium (Anaerobic)	100	0.47
SF3KT13008A	ORT-TM06-212	Oral-tongue coat	Male	Teenager	SCH Medium (Anaerobic)	99.32	0
SF3KT13012A	ORT-TM06-249	Oral-tongue coat	Male	Teenager	SCH Medium (Anaerobic)	99.45	0.27
SF3KT14001B	ORT-TM07-208	Oral-tongue coat	Male	Teenager	Blood-SCH Medium (Aerobic)	99.33	0
SF3KT15002	ORT-AM08-487	Oral-tongue coat	Male	Adult	SCH Medium (Aerobic)	99.33	0
SF3KT16002	ORT-AF06-16	Oral-tongue coat	Female	Adult	SCH Medium (Aerobic)	100	0.48
SF3KT16005	ORT-AF06-19	Oral-tongue coat	Female	Adult	SCH Medium (Aerobic)	99.49	0.76
SF3KT16008	ORT-AF06-143	Oral-tongue coat	Female	Adult	SCH Medium (Aerobic)	99.33	0
T2011065150	ORS-AM09-1-2-O-BH	Oral- saliva	Male	Adult	Blood-BHI (Aerobic)	99.96	0.22
T2011065152	ORS-AM09-3-O-BH	Oral- saliva	Male	Adult	Blood-BHI (Aerobic)	100	0.47
T2011065153	ORS-AM09-4-O-BH	Oral- saliva	Male	Adult	Blood-BHI (Aerobic)	100	0.38
T2011065154	ORS-AM09-5-O-BH	Oral- saliva	Male	Adult	Blood-BHI (Aerobic)	100	0
T2011065155	ORS-AM09-6-O-BH	Oral- saliva	Male	Adult	Blood-BHI (Aerobic)	100	0.38
T2011065157	ORS-AM09-8-O-BH	Oral- saliva	Male	Adult	Blood-BHI (Aerobic)	99.82	0.47
T2011065158	ORS-AM09-9-O-BH	Oral- saliva	Male	Adult	Blood-BHI (Aerobic)	99.82	0.47
T2011065159	ORS-AM09-10-O-BH	Oral- saliva	Male	Adult	Blood-BHI (Aerobic)	99.82	0.47
T2011065161	ORS-AM09-12-2-O-BH	Oral- saliva	Male	Adult	Blood-BHI (Aerobic)	100	0
T2011065165	ORS-AM09-16-O-BH	Oral- saliva	Male	Adult	Blood-BHI (Aerobic)	99.62	0
T2011065166	ORS-AM09-17-O-BH	Oral- saliva	Male	Adult	Blood-BHI (Aerobic)	99.82	0.47
T2011065167	ORS-AM09-18-O-BH	Oral- saliva	Male	Adult	Blood-BHI (Aerobic)	100	0.18
T2011065168	ORS-AM09-19-O-BH	Oral- saliva	Male	Adult	Blood-BHI (Aerobic)	99.82	0.25
T2011065169	ORS-AM09-20-O-BH	Oral- saliva	Male	Adult	Blood-BHI (Aerobic)	100	0
T2011065170	ORS-AM09-22-O-BH	Oral- saliva	Male	Adult	Blood-BHI (Aerobic)	100	0
T2011065171	ORS-AM09-23-O-BH	Oral- saliva	Male	Adult	Blood-BHI (Aerobic)	100	0.95
T2011065172	ORS-AM09-24-O-BH	Oral- saliva	Male	Adult	Blood-BHI (Aerobic)	100	0
T2011065173	ORS-AM09-25-O-BH	Oral- saliva	Male	Adult	Blood-BHI (Aerobic)	99.33	0
T2011065174	ORS-AM09-26-O-BH	Oral- saliva	Male	Adult	Blood-BHI (Aerobic)	100	0.18
T2011065176	ORS-AM09-28-O-BH	Oral- saliva	Male	Adult	Blood-BHI (Aerobic)	100	0.63
T2011065177	ORS-AM09-29-O-BH	Oral- saliva	Male	Adult	Blood-BHI (Aerobic)	100	0.38
T2011065178	ORS-AM09-30-O-BH	Oral- saliva	Male	Adult	Blood-BHI (Aerobic)	100	0.95
T2011065179	ORS-AM09-31-1-O-BH	Oral- saliva	Male	Adult	Blood-BHI (Aerobic)	99.92	0.23
T2011065180	ORS-AM09-31-2-O-BH	Oral- saliva	Male	Adult	Blood-BHI (Aerobic)	99.92	0.23
T2011065182	ORS-AM09-33-O-BH	Oral- saliva	Male	Adult	Blood-BHI (Aerobic)	99.9	0.31
T2011065185	ORS-AM09-36-O-BH	Oral- saliva	Male	Adult	Blood-BHI (Aerobic)	100	0
T2011065187	ORS-AM09-39-O-BH	Oral- saliva	Male	Adult	Blood-BHI (Aerobic)	100	0
T2011065188	ORS-AM09-40-O-BH	Oral- saliva	Male	Adult	Blood-BHI (Aerobic)	99.96	0.27
T2011065189	ORS-AM09-41-O-BH	Oral- saliva	Male	Adult	Blood-BHI (Aerobic)	99.92	0.23
T2011065191	ORS-AM09-43-O-BH	Oral- saliva	Male	Adult	Blood-BHI (Aerobic)	99.87	1
T2011065192	ORS-AM09-45-O-BH	Oral- saliva	Male	Adult	Blood-BHI (Aerobic)	99.42	0.2
T2011065196	ODP-AM09-1-1-O-BH	Oral-tooth	Male	Adult	Blood-BHI (Aerobic)	99.17	0.24
T2011065197	ODP-AM09-1-2-O-BH	Oral-tooth	Male	Adult	Blood-BHI (Aerobic)	99.17	0.24
T2011065200	ODP-AM09-3-O-BH	Oral-tooth	Male	Adult	Blood-BHI (Aerobic)	100	0.47
T2011065201	ODP-AM09-4-O-BH	Oral-tooth	Male	Adult	Blood-BHI (Aerobic)	100	0.19
T2011065202	ODP-AM09-5-O-BH	Oral-tooth	Male	Adult	Blood-BHI (Aerobic)	100	0.19
T2011065206	ODP-AM09-9-O-BH	Oral-tooth	Male	Adult	Blood-BHI (Aerobic)	99.82	0.47
T2011065207	ODP-AM09-10-O-BH	Oral-tooth	Male	Adult	Blood-BHI (Aerobic)	100	0
T2011065209	ODP-AM09-12-O-BH	Oral-tooth	Male	Adult	Blood-BHI (Aerobic)	99.82	0.47
T2011065210	ODP-AM09-13-O-BH	Oral-tooth	Male	Adult	Blood-BHI (Aerobic)	99.81	0
T2011065213	ODP-AM09-16-O-BH	Oral-tooth	Male	Adult	Blood-BHI (Aerobic)	99.82	0.64
T2011065214	ODP-AM09-17-O-BH	Oral-tooth	Male	Adult	Blood-BHI (Aerobic)	100	0.18
T2011065216	ODP-AM09-19-O-BH	Oral-tooth	Male	Adult	Blood-BHI (Aerobic)	98.9	1.39
T2011065217	ODP-AM09-20-O-BH	Oral-tooth	Male	Adult	Blood-BHI (Aerobic)	99.82	0.47
T2011065220	ODP-AM09-23-O-BH	Oral-tooth	Male	Adult	Blood-BHI (Aerobic)	100	0.18
T2011065222	ODP-AM09-25-O-BH	Oral-tooth	Male	Adult	Blood-BHI (Aerobic)	100	4.29
T2011065225	ODP-AM09-28-O-BH	Oral-tooth	Male	Adult	Blood-BHI (Aerobic)	100	0.47
T2011065226	ODP-AM09-29-O-BH	Oral-tooth	Male	Adult	Blood-BHI (Aerobic)	100	0
T2011065228	ODP-AM09-31-O-BH	Oral-tooth	Male	Adult	Blood-BHI (Aerobic)	100	0
T2011065230	ODP-AM09-33-O-BH	Oral-tooth	Male	Adult	Blood-BHI (Aerobic)	100	0
T2011065231	ODP-AM09-34-O-BH	Oral-tooth	Male	Adult	Blood-BHI (Aerobic)	100	0.95
T2011065233	ODP-AM09-36-O-BH	Oral-tooth	Male	Adult	Blood-BHI (Aerobic)	99.82	0.47
T2011065234	ODP-AM09-37-O-BH	Oral-tooth	Male	Adult	Blood-BHI (Aerobic)	100	0
T2011065235	ODP-AM09-38-O-BH	Oral-tooth	Male	Adult	Blood-BHI (Aerobic)	99.74	0.47
T2011065236	ODP-AM09-39-O-BH	Oral-tooth	Male	Adult	Blood-BHI (Aerobic)	99.74	1.03
T2011065238	ODP-AM09-41-O-BH	Oral-tooth	Male	Adult	Blood-BHI (Aerobic)	99.66	0
T2011065239	ODP-AM09-42-O-BH	Oral-tooth	Male	Adult	Blood-BHI (Aerobic)	99.82	0.47
T2011065241	ODP-AM09-44-O-BH	Oral-tooth	Male	Adult	Blood-BHI (Aerobic)	100	0.47
T2011065242	ODP-AM09-45-O-BH	Oral-tooth	Male	Adult	Blood-BHI (Aerobic)	99.82	0.47
T2011065243	ODP-AM09-46-O-BH	Oral-tooth	Male	Adult	Blood-BHI (Aerobic)	99.82	0.64
T2011065244	ODP-AM09-47-O-BH	Oral-tooth	Male	Adult	Blood-BHI (Aerobic)	99.82	0.47
T2011065245	ODP-AM09-48-O-BH	Oral-tooth	Male	Adult	Blood-BHI (Aerobic)	100	0
T2011065246	ODP-AM09-49-O-BH	Oral-tooth	Male	Adult	Blood-BHI (Aerobic)	99.87	0.6
T2011068060	ORS-AM09-2-BH	Oral- saliva	Male	Adult	Blood-BHI (Anaerobic)	100	0.47
T2011068063	ORS-AM09-5-BH	Oral- saliva	Male	Adult	Blood-BHI (Anaerobic)	99.37	0
T2011068065	ORS-AM09-7-BH	Oral- saliva	Male	Adult	Blood-BHI (Anaerobic)	99.45	0.27
T2011068069	ORS-AM09-12-BH	Oral- saliva	Male	Adult	Blood-BHI (Anaerobic)	100	0.47
T2011068071	ORS-AM09-16-BH	Oral- saliva	Male	Adult	Blood-BHI (Anaerobic)	99.82	0.64
T2011068073	ORS-AM09-18-BH	Oral- saliva	Male	Adult	Blood-BHI (Anaerobic)	100	0.47
T2011068074	ORS-AM09-19-BH	Oral- saliva	Male	Adult	Blood-BHI (Anaerobic)	98.56	0
T2011068075	ORS-AM09-20-BH	Oral- saliva	Male	Adult	Blood-BHI (Anaerobic)	99.44	0.29
T2011068076	ORS-AM09-22-BH	Oral- saliva	Male	Adult	Blood-BHI (Anaerobic)	100	0.95
T2011068079	ORS-AM09-26-BH	Oral- saliva	Male	Adult	Blood-BHI (Anaerobic)	100	0.47
T2011068080	ORS-AM09-27-BH	Oral- saliva	Male	Adult	Blood-BHI (Anaerobic)	100	0.19
T2011068081	ORS-AM09-28-BH	Oral- saliva	Male	Adult	Blood-BHI (Anaerobic)	99.66	0.11
T2011068082	ORS-AM09-29-BH	Oral- saliva	Male	Adult	Blood-BHI (Anaerobic)	100	0.47
T2011068083	ORS-AM09-30-BH	Oral- saliva	Male	Adult	Blood-BHI (Anaerobic)	99.44	0.29
T2011068084	ORS-AM09-31-BH	Oral- saliva	Male	Adult	Blood-BHI (Anaerobic)	100	0.47

T2011068085	ORS-AM09-32-BH	Oral- saliva	Male	Adult	Blood-BHI (Anaerobic)	98.28	0
T2011068087	ODP-AM09-1-BH	Oral-tooth	Male	Adult	Blood-BHI (Anaerobic)	99.74	0.47
T2011068089	ODP-AM09-3-BH	Oral-tooth	Male	Adult	Blood-BHI (Anaerobic)	99.82	0.47
T2011068093	ODP-AM09-7-BH	Oral-tooth	Male	Adult	Blood-BHI (Anaerobic)	99.82	0.47
T2011068096	ODP-AM09-11-BH	Oral-tooth	Male	Adult	Blood-BHI (Anaerobic)	100	0
T2011068099	ODP-AM09-14-BH	Oral-tooth	Male	Adult	Blood-BHI (Anaerobic)	100	0
T2011068107	ODP-AM09-22-BH	Oral-tooth	Male	Adult	Blood-BHI (Anaerobic)	99.82	0.47
T2011068108	ODP-AM09-23-BH	Oral-tooth	Male	Adult	Blood-BHI (Anaerobic)	99.87	0.2
T2011068109	ODP-AM09-24-BH	Oral-tooth	Male	Adult	Blood-BHI (Anaerobic)	99.82	0.47
T2011068112	ODP-AM09-27-BH	Oral-tooth	Male	Adult	Blood-BHI (Anaerobic)	99.42	0.2
T2011068113	ODP-AM09-30-BH	Oral-tooth	Male	Adult	Blood-BHI (Anaerobic)	100	0
T2011068114	ODP-AM09-32-BH	Oral-tooth	Male	Adult	Blood-BHI (Anaerobic)	99.82	0.47
T2011068115	ODP-AM09-33-BH	Oral-tooth	Male	Adult	Blood-BHI (Anaerobic)	99.54	0.71
T2011068116	ODP-AM09-34-BH	Oral-tooth	Male	Adult	Blood-BHI (Anaerobic)	99	0.09
T2011068119	ODP-AM09-37-BH	Oral-tooth	Male	Adult	Blood-BHI (Anaerobic)	100	1.9
T2011068120	ODP-AM09-39-BH	Oral-tooth	Male	Adult	Blood-BHI (Anaerobic)	100	0
T2011068121	ODP-AM09-41-BH	Oral-tooth	Male	Adult	Blood-BHI (Anaerobic)	100	0.47
T2011068122	ODP-AM09-43-BH	Oral-tooth	Male	Adult	Blood-BHI (Anaerobic)	100	0
T2101076727	ORT-AM09-2-O-104	Oral-tongue coat	Male	Adult	MPYG Medium (Aerobic)	99.82	0.24
T2101076728	ORT-AM09-3-O-104	Oral-tongue coat	Male	Adult	MPYG Medium (Aerobic)	99.82	0.32
T2101076730	ORT-AM09-5-O-104	Oral-tongue coat	Male	Adult	MPYG Medium (Aerobic)	100	0
T2101076731	ORT-AM09-6-O-104	Oral-tongue coat	Male	Adult	MPYG Medium (Aerobic)	99.87	1
T2101076732	ORT-AM09-7-O-104	Oral-tongue coat	Male	Adult	MPYG Medium (Aerobic)	100	0
T2101076733	ORT-AM09-8-O-104	Oral-tongue coat	Male	Adult	MPYG Medium (Aerobic)	100	0.07
T2101076734	ORT-AM09-9-O-104	Oral-tongue coat	Male	Adult	MPYG Medium (Aerobic)	100	0
T2101076735	ORT-AM09-10-O-104	Oral-tongue coat	Male	Adult	MPYG Medium (Aerobic)	99.45	0.27
T2101076737	ORT-AM09-12-O-104	Oral-tongue coat	Male	Adult	MPYG Medium (Aerobic)	99.32	0
T2101076738	ORT-AM09-13-O-104	Oral-tongue coat	Male	Adult	MPYG Medium (Aerobic)	99.49	0.17
T2101076739	ORT-AM09-14-O-104	Oral-tongue coat	Male	Adult	MPYG Medium (Aerobic)	99.83	0
T2101076741	ORT-AM09-18-O-104	Oral-tongue coat	Male	Adult	MPYG Medium (Aerobic)	99.92	0.55
T2101076743	ORT-AM09-20-O-104	Oral-tongue coat	Male	Adult	MPYG Medium (Aerobic)	100	0.07
T2101076744	ORT-AM09-21-O-104	Oral-tongue coat	Male	Adult	MPYG Medium (Aerobic)	99.17	0.33
T2101076745	ORT-AM09-22-O-104	Oral-tongue coat	Male	Adult	MPYG Medium (Aerobic)	99.82	0.2
T2101076746	ORT-AM09-23-O-104	Oral-tongue coat	Male	Adult	MPYG Medium (Aerobic)	99.83	0.17
T2101076747	ORT-AM09-24-O-104	Oral-tongue coat	Male	Adult	MPYG Medium (Aerobic)	99.82	0.2
T2101076748	ORT-AM09-25-O-104	Oral-tongue coat	Male	Adult	MPYG Medium (Aerobic)	98.56	0.57
T2101076749	ORT-AM09-26-O-104	Oral-tongue coat	Male	Adult	MPYG Medium (Aerobic)	99.83	0.17
T2101076750	ORT-AM09-28-O-104	Oral-tongue coat	Male	Adult	MPYG Medium (Aerobic)	99.83	0.17
T2101076751	ORT-AM09-29-O-104	Oral-tongue coat	Male	Adult	MPYG Medium (Aerobic)	99.83	0
T2101076754	ODP-AM09-2-O-104	Oral-tooth	Male	Adult	MPYG Medium (Aerobic)	99.82	0.24
T2101076755	ODP-AM09-3-O-104	Oral-tooth	Male	Adult	MPYG Medium (Aerobic)	99.82	0.24
T2101076756	ODP-AM09-4-O-104	Oral-tooth	Male	Adult	MPYG Medium (Aerobic)	99	0.09
T2101076757	ODP-AM09-5-O-104	Oral-tooth	Male	Adult	MPYG Medium (Aerobic)	100	0
T2101076758	ODP-AM09-6-O-104	Oral-tooth	Male	Adult	MPYG Medium (Aerobic)	99.87	0.2
T2101076761	ODP-AM09-9-O-104	Oral-tooth	Male	Adult	MPYG Medium (Aerobic)	99.82	0.2
T2101076762	ODP-AM09-10-O-104	Oral-tooth	Male	Adult	MPYG Medium (Aerobic)	99.82	0.2
T2101076764	ODP-AM09-12-O-104	Oral-tooth	Male	Adult	MPYG Medium (Aerobic)	99.35	0
T2101076767	ODP-AM09-16-O-104	Oral-tooth	Male	Adult	MPYG Medium (Aerobic)	99.82	0.2
T2101076768	ODP-AM09-18-O-104	Oral-tooth	Male	Adult	MPYG Medium (Aerobic)	99.82	0.32
T2101076769	ODP-AM09-19-O-104	Oral-tooth	Male	Adult	MPYG Medium (Aerobic)	99.82	0.32
T2101076770	ODP-AM09-20-O-104	Oral-tooth	Male	Adult	MPYG Medium (Aerobic)	99.82	0.24
T2101076771	ODP-AM09-21-O-104	Oral-tooth	Male	Adult	MPYG Medium (Aerobic)	99.87	0.2
T2101076772	ODP-AM09-22-O-104	Oral-tooth	Male	Adult	MPYG Medium (Aerobic)	99.87	0.2
T2101076773	ODP-AM09-23-O-104	Oral-tooth	Male	Adult	MPYG Medium (Aerobic)	99.82	0.2
T2101076774	ORS-AM09-1-O-104	Oral- saliva	Male	Adult	MPYG Medium (Aerobic)	100	0.4
T2101076775	ORS-AM09-2-O-104	Oral- saliva	Male	Adult	MPYG Medium (Aerobic)	99.7	0.25
T2101076776	ORS-AM09-3-O-104	Oral- saliva	Male	Adult	MPYG Medium (Aerobic)	99.49	0.17
T2101076777	ORS-AM09-4-O-104	Oral- saliva	Male	Adult	MPYG Medium (Aerobic)	99.82	0.2
T2101076780	ORS-AM09-7-O-104	Oral- saliva	Male	Adult	MPYG Medium (Aerobic)	100	0.19
T2101076781	ORS-AM09-8-O-104	Oral- saliva	Male	Adult	MPYG Medium (Aerobic)	99.9	0.68
T2101076782	ORS-AM09-9-O-104	Oral- saliva	Male	Adult	MPYG Medium (Aerobic)	99.82	0.2
T2101076783	ORS-AM09-10-O-104	Oral- saliva	Male	Adult	MPYG Medium (Aerobic)	100	0
T2101076784	ORS-AM09-11-O-104	Oral- saliva	Male	Adult	MPYG Medium (Aerobic)	99.82	0.2
T2101076786	ORS-AM09-13-O-104	Oral- saliva	Male	Adult	MPYG Medium (Aerobic)	99.96	0.55
T2101076787	ORS-AM09-14-O-104	Oral- saliva	Male	Adult	MPYG Medium (Aerobic)	99.92	0.55
T2101076788	ORS-AM09-15-O-104	Oral- saliva	Male	Adult	MPYG Medium (Aerobic)	99.9	0.15
T2101076789	ORS-AM09-16-O-104	Oral- saliva	Male	Adult	MPYG Medium (Aerobic)	99.9	0.68
T2101076791	ORS-AM09-21-O-104	Oral- saliva	Male	Adult	MPYG Medium (Aerobic)	99.96	2.33
T2101076792	ORS-AM09-23-O-104	Oral- saliva	Male	Adult	MPYG Medium (Aerobic)	100	0
T2101076793	ORS-AM09-24-O-104	Oral- saliva	Male	Adult	MPYG Medium (Aerobic)	99.49	0.17
T2101076794	ORS-AM09-27-O-104	Oral- saliva	Male	Adult	MPYG Medium (Aerobic)	99.82	0.2
T2101076795	ORS-AF08-1-MRS	Oral- saliva	Female	Adult	MRS Medium (Anaerobic)	100	0.34
T2101076796	ORS-AF08-2-MRS	Oral- saliva	Female	Adult	MRS Medium (Anaerobic)	99.9	0.68
T2101076797	ORS-AF08-3-MRS	Oral- saliva	Female	Adult	MRS Medium (Anaerobic)	98.91	0
T2101076798	ORS-AF08-4-MRS	Oral- saliva	Female	Adult	MRS Medium (Anaerobic)	99.82	0.2
T2101076799	ORS-AF08-5-MRS	Oral- saliva	Female	Adult	MRS Medium (Anaerobic)	99.9	0.68
T2101076801	ORS-AF08-7-MRS	Oral- saliva	Female	Adult	MRS Medium (Anaerobic)	99.96	0.15
T2101076802	ORS-AF08-8-MRS	Oral- saliva	Female	Adult	MRS Medium (Anaerobic)	99.82	0.24
T2101076803	ORS-AF08-9-MRS	Oral- saliva	Female	Adult	MRS Medium (Anaerobic)	99.32	0
T2101076804	ORS-AF08-12-MRS	Oral- saliva	Female	Adult	MRS Medium (Anaerobic)	99.82	0.32
T2101076805	ORS-AF08-14-MRS	Oral- saliva	Female	Adult	MRS Medium (Anaerobic)	99.45	0.82
T2101076807	ORS-AF08-16-MRS	Oral- saliva	Female	Adult	MRS Medium (Anaerobic)	99.2	0
T2101076808	ORS-AF08-17-MRS	Oral- saliva	Female	Adult	MRS Medium (Anaerobic)	99.82	0.32
T2101076809	ORS-AF08-18-MRS	Oral- saliva	Female	Adult	MRS Medium (Anaerobic)	99.83	0
T2101076810	ORS-AF08-19-MRS	Oral- saliva	Female	Adult	MRS Medium (Anaerobic)	99.32	0
T2101076811	ORS-AF08-20-MRS	Oral- saliva	Female	Adult	MRS Medium (Anaerobic)	99.96	2.33

T2101076813	ORS-AF08-23-MRS	Oral- saliva	Female	Adult	MRS Medium (Anaerobic)	99.9	0.68
T2101076815	ORS-AF08-25-MRS	Oral- saliva	Female	Adult	MRS Medium (Anaerobic)	100	0
T2101076817	ORS-AF08-27-MRS	Oral- saliva	Female	Adult	MRS Medium (Anaerobic)	99.45	1.55
T2101076820	ORS-AF08-30-MRS	Oral- saliva	Female	Adult	MRS Medium (Anaerobic)	99.9	0.68
T2101076821	ODP-AF08-1-MRS	Oral-tooth	Female	Adult	MRS Medium (Anaerobic)	100	0
T2101076822	ODP-AF08-2-MRS	Oral-tooth	Female	Adult	MRS Medium (Anaerobic)	100	0
T2101076825	ODP-AF08-7-MRS	Oral-tooth	Female	Adult	MRS Medium (Anaerobic)	99.82	0.2
T2101076826	ODP-AF08-8-MRS	Oral-tooth	Female	Adult	MRS Medium (Anaerobic)	99.82	0.2
T2101076827	ODP-AF08-10-MRS	Oral-tooth	Female	Adult	MRS Medium (Anaerobic)	99.82	0.2
T2101076828	ODP-AF08-11-MRS	Oral-tooth	Female	Adult	MRS Medium (Anaerobic)	100	0
T2101076829	ODP-AF08-13-MRS	Oral-tooth	Female	Adult	MRS Medium (Anaerobic)	99.35	0
T2101076831	ODP-AF08-15-MRS	Oral-tooth	Female	Adult	MRS Medium (Anaerobic)	99.29	0.07
T2101076832	ODP-AF08-16-MRS	Oral-tooth	Female	Adult	MRS Medium (Anaerobic)	99.35	0
T2101076833	ODP-AF08-17-MRS	Oral-tooth	Female	Adult	MRS Medium (Anaerobic)	100	0
T2101076834	ODP-AF08-18-MRS	Oral-tooth	Female	Adult	MRS Medium (Anaerobic)	100	0
T2101076835	ODP-AF08-20-MRS	Oral-tooth	Female	Adult	MRS Medium (Anaerobic)	99.35	0
T2101076836	ODP-AF08-21-MRS	Oral-tooth	Female	Adult	MRS Medium (Anaerobic)	99.82	0.2
T2101076837	ODP-AF08-22-MRS	Oral-tooth	Female	Adult	MRS Medium (Anaerobic)	100	0
T2101076838	ODP-AF08-23-MRS	Oral-tooth	Female	Adult	MRS Medium (Anaerobic)	99.82	0.2
T2101076840	ODP-AF08-27-MRS	Oral-tooth	Female	Adult	MRS Medium (Anaerobic)	100	0
T2101076841	ODP-AF08-28-MRS	Oral-tooth	Female	Adult	MRS Medium (Anaerobic)	99.45	0.55
T2101076844	ODP-AF08-35-MRS	Oral-tooth	Female	Adult	MRS Medium (Anaerobic)	99.82	0.2
T2101076845	ODP-AF08-36-MRS	Oral-tooth	Female	Adult	MRS Medium (Anaerobic)	99.82	0.2
T2101076846	ODP-AF08-37-MRS	Oral-tooth	Female	Adult	MRS Medium (Anaerobic)	99.45	0.55
T2101076847	ODP-AF08-38-MRS	Oral-tooth	Female	Adult	MRS Medium (Anaerobic)	99.82	0.2
T2101076848	ODP-AF08-39-MRS	Oral-tooth	Female	Adult	MRS Medium (Anaerobic)	99.82	0.24
T2101076849	ODP-AF08-40-MRS	Oral-tooth	Female	Adult	MRS Medium (Anaerobic)	99.35	0
T2101076850	ODP-AF08-41-MRS	Oral-tooth	Female	Adult	MRS Medium (Anaerobic)	99.82	0.2
T2101076851	ODP-AF08-42-MRS	Oral-tooth	Female	Adult	MRS Medium (Anaerobic)	99.82	0.24
T2101076854	ORT-AF08-11-MRS	Oral-tongue coat	Female	Adult	MRS Medium (Anaerobic)	100	0
T2101076857	ORT-AF08-19-MRS	Oral-tongue coat	Female	Adult	MRS Medium (Anaerobic)	100	0
T2101076858	ORT-AF08-24-MRS	Oral-tongue coat	Female	Adult	MRS Medium (Anaerobic)	99.9	0.15
T2101076859	ORT-AF08-28-MRS	Oral-tongue coat	Female	Adult	MRS Medium (Anaerobic)	99.35	0
T2101076860	ORT-AF08-32-MRS	Oral-tongue coat	Female	Adult	MRS Medium (Anaerobic)	99.35	0
T2101076861	ORT-AF08-34-MRS	Oral-tongue coat	Female	Adult	MRS Medium (Anaerobic)	99.82	0.2
T2101076862	ORT-AF08-35-MRS	Oral-tongue coat	Female	Adult	MRS Medium (Anaerobic)	100	0
T2101076866	ORT-AF08-43-MRS	Oral-tongue coat	Female	Adult	MRS Medium (Anaerobic)	99.87	0.2
T2101078470	ORT-AM09-5D4	Oral-tongue coat	Male	Adult	Blood-Enriched-BHI (Anaerobic)	100	0
T2101078472	ORT-AM09-5D6	Oral-tongue coat	Male	Adult	Blood-Enriched-BHI (Anaerobic)	99.91	0.61
T2101078474	ODP-AM09-2D1	Oral-tooth	Male	Adult	Blood-Enriched-BHI (Anaerobic)	99.44	0.29
T2101078475	ODP-AM09-2D2	Oral-tooth	Male	Adult	Blood-Enriched-BHI (Anaerobic)	99.44	0.29
T2101078477	ODP-AM09-2D4A	Oral-tooth	Male	Adult	Blood-Enriched-BHI (Anaerobic)	99.44	0.29
T2101078478	ODP-AM09-2D4B	Oral-tooth	Male	Adult	Blood-Enriched-BHI (Anaerobic)	99.44	0.29
T2101078479	ODP-AM09-2D5	Oral-tooth	Male	Adult	Blood-Enriched-BHI (Anaerobic)	99.46	0.22
T2101078481	ODP-AM09-2D6	Oral-tooth	Male	Adult	Blood-Enriched-BHI (Anaerobic)	99.63	0
T2101078490	ODP-AM09-2D16	Oral-tooth	Male	Adult	Blood-Enriched-BHI (Anaerobic)	99.44	0.29
T2101078492	ODP-AM09-2D18	Oral-tooth	Male	Adult	Blood-Enriched-BHI (Anaerobic)	100	0
T2101078494	ODP-AM09-2D20	Oral-tooth	Male	Adult	Blood-Enriched-BHI (Anaerobic)	99.88	0.29
T2101078495	ODP-AM09-2D21	Oral-tooth	Male	Adult	Blood-Enriched-BHI (Anaerobic)	100	0
T2101078500	ODP-AF08-11-O-MRS	Oral-tooth	Female	Adult	MRS Medium (Aerobic)	99.65	0.07
T2101078504	ODP-AF08-18-O-MRS	Oral-tooth	Female	Adult	MRS Medium (Aerobic)	99.9	0.68
T2101078508	ODP-AF08-25-O-MRS	Oral-tooth	Female	Adult	MRS Medium (Aerobic)	99.65	0.07
T2101078509	ODP-AF08-26-O-MRS	Oral-tooth	Female	Adult	MRS Medium (Aerobic)	100	0
T2101078510	ODP-AF08-27-O-MRS	Oral-tooth	Female	Adult	MRS Medium (Aerobic)	99.65	0.07
T2101078518	ORT-AF08-12-O-MRS	Oral-tongue coat	Female	Adult	MRS Medium (Aerobic)	99.9	0.68
T2101078520	ORT-AF08-14-O-MRS	Oral-tongue coat	Female	Adult	MRS Medium (Aerobic)	100	0
T2101078525	ORT-AF08-20-O-MRS	Oral-tongue coat	Female	Adult	MRS Medium (Aerobic)	100	0
T2101078526	ORT-AF08-21-O-MRS	Oral-tongue coat	Female	Adult	MRS Medium (Aerobic)	99.82	0.2
T2101081681	ORS-AM09-1-104	Oral- saliva	Male	Adult	MPYG Medium (Anaerobic)	99.82	0.25
T2101081682	ORS-AM09-2-104	Oral- saliva	Male	Adult	MPYG Medium (Anaerobic)	99.37	0
T2101081683	ORS-AM09-3-104	Oral- saliva	Male	Adult	MPYG Medium (Anaerobic)	98.37	0
T2101081684	ORS-AM09-4-104	Oral- saliva	Male	Adult	MPYG Medium (Anaerobic)	100	0.11
T2101081685	ORS-AM09-5-104	Oral- saliva	Male	Adult	MPYG Medium (Anaerobic)	99.82	0.8
T2101081686	ORS-AM09-6-104	Oral- saliva	Male	Adult	MPYG Medium (Anaerobic)	100	0
T2101081688	ORS-AM09-8-104	Oral- saliva	Male	Adult	MPYG Medium (Anaerobic)	99.96	0.27
T2101081689	ORS-AM09-9-104	Oral- saliva	Male	Adult	MPYG Medium (Anaerobic)	99.82	0.47
T2101081690	ORS-AM09-10-104	Oral- saliva	Male	Adult	MPYG Medium (Anaerobic)	99.62	0.11
T2101081692	ORS-AM09-12-104	Oral- saliva	Male	Adult	MPYG Medium (Anaerobic)	99.44	0.29
T2101081693	ORS-AM09-13-104	Oral- saliva	Male	Adult	MPYG Medium (Anaerobic)	100	0
T2101081695	ORS-AM09-15-104	Oral- saliva	Male	Adult	MPYG Medium (Anaerobic)	100	0.17
T2101081696	ORS-AM09-16-104	Oral- saliva	Male	Adult	MPYG Medium (Anaerobic)	99.82	0.64
T2101081698	ORS-AM09-18-104	Oral- saliva	Male	Adult	MPYG Medium (Anaerobic)	100	0
T2101081699	ORS-AM09-19-104	Oral- saliva	Male	Adult	MPYG Medium (Anaerobic)	99.82	0.47
T2101081702	ORS-AM09-22-104	Oral- saliva	Male	Adult	MPYG Medium (Anaerobic)	99.82	1.03
T2101081708	ODP-AM09-3-104	Oral-tooth	Male	Adult	MPYG Medium (Anaerobic)	99.82	4.32
T2101081710	ODP-AM09-5-104	Oral-tooth	Male	Adult	MPYG Medium (Anaerobic)	99.82	0.64
T2101081711	ODP-AM09-6-104	Oral-tooth	Male	Adult	MPYG Medium (Anaerobic)	99.82	0.25
T2101081713	ODP-AM09-8-104	Oral-tooth	Male	Adult	MPYG Medium (Anaerobic)	100	0
T2101081714	ODP-AM09-9-104	Oral-tooth	Male	Adult	MPYG Medium (Anaerobic)	99.82	0.25
T2101081715	ODP-AM09-10-104	Oral-tooth	Male	Adult	MPYG Medium (Anaerobic)	99.82	0.47
T2101081716	ODP-AM09-11-104	Oral-tooth	Male	Adult	MPYG Medium (Anaerobic)	100	0
T2101081717	ODP-AM09-12-104	Oral-tooth	Male	Adult	MPYG Medium (Anaerobic)	99.82	0.25
T2101081718	ODP-AM09-13-104	Oral-tooth	Male	Adult	MPYG Medium (Anaerobic)	99.82	0.25
T2101081719	ODP-AM09-14-104	Oral-tooth	Male	Adult	MPYG Medium (Anaerobic)	100	0.42
T2101081720	ODP-AM09-15-104	Oral-tooth	Male	Adult	MPYG Medium (Anaerobic)	99.82	0.47
T2101081722	ODP-AM09-17-104	Oral-tooth	Male	Adult	MPYG Medium (Anaerobic)	99.87	0.2

T2101081723	ODP-AM09-18-104	Oral-tooth	Male	Adult	MPYG Medium (Anaerobic)	100	0
T2101081725	ODP-AM09-20-104	Oral-tooth	Male	Adult	MPYG Medium (Anaerobic)	99.82	0.25
T2101081726	ODP-AM09-21-104	Oral-tooth	Male	Adult	MPYG Medium (Anaerobic)	99.82	0.25
T2101081727	ODP-AM09-22-104	Oral-tooth	Male	Adult	MPYG Medium (Anaerobic)	100	0
T2101081728	ODP-AM09-23-104	Oral-tooth	Male	Adult	MPYG Medium (Anaerobic)	99.82	0.64
T2101081729	ODP-AM09-24-104	Oral-tooth	Male	Adult	MPYG Medium (Anaerobic)	100	0
T2101081731	ODP-AM09-26-104	Oral-tooth	Male	Adult	MPYG Medium (Anaerobic)	99.87	0.2
T2101081732	ODP-AM09-27-104	Oral-tooth	Male	Adult	MPYG Medium (Anaerobic)	99.82	0.64
T2101081734	ORT-AM09-3-104	Oral-tongue coat	Male	Adult	MPYG Medium (Anaerobic)	100	0
T2101081735	ORT-AM09-5-104	Oral-tongue coat	Male	Adult	MPYG Medium (Anaerobic)	100	0.18
T2101081736	ORT-AM09-6-104	Oral-tongue coat	Male	Adult	MPYG Medium (Anaerobic)	100	0.18
T2101081738	ORT-AM09-8-104	Oral-tongue coat	Male	Adult	MPYG Medium (Anaerobic)	100	0
T2101081739	ORT-AM09-9-104	Oral-tongue coat	Male	Adult	MPYG Medium (Anaerobic)	100	0
T2101081740	ORT-AM09-10-104	Oral-tongue coat	Male	Adult	MPYG Medium (Anaerobic)	100	0
T2101081744	ORT-AM09-14-104	Oral-tongue coat	Male	Adult	MPYG Medium (Anaerobic)	100	0.47
T2101081746	ORT-AM09-17-104	Oral-tongue coat	Male	Adult	MPYG Medium (Anaerobic)	100	0.38
T2101081747	ORT-AM09-19-104	Oral-tongue coat	Male	Adult	MPYG Medium (Anaerobic)	100	0
T2101081750	ORT-AM09-23-104	Oral-tongue coat	Male	Adult	MPYG Medium (Anaerobic)	100	0
T2102085941	ORS-AF08-20	Oral- saliva	Female	Adult	MPYG Medium (Anaerobic)	99.52	0.2
T2102085942	ORS-AF08-21	Oral- saliva	Female	Adult	MPYG Medium (Anaerobic)	99.82	0.2
T2102085943	ORS-AF08-22	Oral- saliva	Female	Adult	MPYG Medium (Anaerobic)	99.82	0.2
T2102085944	ORS-AF08-23	Oral- saliva	Female	Adult	MPYG Medium (Anaerobic)	99.2	0
T2102085945	ORS-AF08-24	Oral- saliva	Female	Adult	MPYG Medium (Anaerobic)	99.82	0.2
T2102085947	ORT-AF08-2	Oral-tongue coat	Female	Adult	MPYG Medium (Anaerobic)	99.65	0.07
T2102085948	ORT-AF08-3	Oral-tongue coat	Female	Adult	MPYG Medium (Anaerobic)	98.01	0.02
T2102085949	ORT-AF08-4	Oral-tongue coat	Female	Adult	MPYG Medium (Anaerobic)	99.17	0.33
T2102085950	ORT-AF08-5	Oral-tongue coat	Female	Adult	MPYG Medium (Anaerobic)	99.2	0
T2102085951	ORT-AF08-6	Oral-tongue coat	Female	Adult	MPYG Medium (Anaerobic)	99.96	0.15
T2102085952	ORT-AF08-7	Oral-tongue coat	Female	Adult	MPYG Medium (Anaerobic)	100	0
T2102085953	ORT-AF08-8	Oral-tongue coat	Female	Adult	MPYG Medium (Anaerobic)	99.96	0.15
T2102085954	ORT-AF08-9	Oral-tongue coat	Female	Adult	MPYG Medium (Anaerobic)	99.96	0.15
T2102085955	ORT-AF08-10	Oral-tongue coat	Female	Adult	MPYG Medium (Anaerobic)	98.79	0.17
T2102085956	ORT-AF08-11	Oral-tongue coat	Female	Adult	MPYG Medium (Anaerobic)	99.82	0.27
T2102085957	ORT-AF08-12	Oral-tongue coat	Female	Adult	MPYG Medium (Anaerobic)	99.9	0.15
T2102085958	ORT-AF08-13	Oral-tongue coat	Female	Adult	MPYG Medium (Anaerobic)	99.82	0.27
T2102085960	ORT-AF08-15	Oral-tongue coat	Female	Adult	MPYG Medium (Anaerobic)	99.96	0.15
T2102085961	ORT-AF08-16	Oral-tongue coat	Female	Adult	MPYG Medium (Anaerobic)	99.87	1
T2102085962	ORT-AF08-17	Oral-tongue coat	Female	Adult	MPYG Medium (Anaerobic)	99.66	0
T2102085963	ORT-AF08-18	Oral-tongue coat	Female	Adult	MPYG Medium (Anaerobic)	99.49	0.17
T2102085964	ORT-AF08-19	Oral-tongue coat	Female	Adult	MPYG Medium (Anaerobic)	99.82	0
T2102085965	ODP-AF08-2	Oral-tooth	Female	Adult	MPYG Medium (Anaerobic)	99.69	3.1
T2102085966	ODP-AF08-3	Oral-tooth	Female	Adult	MPYG Medium (Anaerobic)	99.35	0
T2102085968	ORS-AM09-5D1B	Oral- saliva	Male	Adult	Blood-Enriched-BHI (Anaerobic)	100	0
T2102085969	ORS-AM09-5D2A	Oral- saliva	Male	Adult	Blood-Enriched-BHI (Anaerobic)	98.6	0
T2102085974	ODP-AM09-2D3B	Oral-tooth	Male	Adult	Blood-Enriched-BHI (Anaerobic)	99.78	0
T2102085975	ORS-AF08-13B	Oral- saliva	Female	Adult	MRS Medium (Anaerobic)	98.91	0.82
T2102085978	ORT-AF08-8-MRS	Oral-tongue coat	Female	Adult	MRS Medium (Aerobic)	99.33	0
T2102085980	ODP-AF08-20	Oral-tooth	Female	Adult	MRS Medium (Aerobic)	99.55	0.75
T2103096290	ORS-AF10-1-O-BH	Oral- saliva	Female	Adult	Blood-BHI (Aerobic)	100	0.59
T2103096291	ORS-AF10-5-O-BH	Oral- saliva	Female	Adult	Blood-BHI (Aerobic)	100	0.59
T2103096292	ORS-AF10-6-O-BH	Oral- saliva	Female	Adult	Blood-BHI (Aerobic)	99.92	0.23
T2103096301	ORS-AF10-17-O-BH	Oral- saliva	Female	Adult	Blood-BHI (Aerobic)	99.7	0.23
T2103096302	ORS-AF10-18-O-BH	Oral- saliva	Female	Adult	Blood-BHI (Aerobic)	99.34	0
T2103096304	ORS-AF10-20-O-BH	Oral- saliva	Female	Adult	Blood-BHI (Aerobic)	99.87	0.2
T2103096306	ORS-AF10-23-O-BH	Oral- saliva	Female	Adult	Blood-BHI (Aerobic)	100	0.47
T2103096307	ORS-AF10-24-O-BH	Oral- saliva	Female	Adult	Blood-BHI (Aerobic)	99.92	0.23
T2103096310	ORS-AF10-28-O-BH	Oral- saliva	Female	Adult	Blood-BHI (Aerobic)	100	0.47
T2103096316	ODP-AF10-3-O-BH	Oral-tooth	Female	Adult	Blood-BHI (Aerobic)	100	0.59
T2103096318	ODP-AF10-6-O-BH	Oral-tooth	Female	Adult	Blood-BHI (Aerobic)	99.34	0
T2103096320	ODP-AF10-9-O-BH	Oral-tooth	Female	Adult	Blood-BHI (Aerobic)	99.34	0
T2103096321	ODP-AF10-10-O-BH	Oral-tooth	Female	Adult	Blood-BHI (Aerobic)	99.34	0
T2103096323	ODP-AF10-12-O-BH	Oral-tooth	Female	Adult	Blood-BHI (Aerobic)	100	0.59
T2103096327	ODP-AF10-17-O-BH	Oral-tooth	Female	Adult	Blood-BHI (Aerobic)	99.87	0
T2103096348	ORT-AF10-10-O-BH	Oral-tongue coat	Female	Adult	Blood-BHI (Aerobic)	99.88	0.15
T2103096349	ORT-AF10-11-O-BH	Oral-tongue coat	Female	Adult	Blood-BHI (Aerobic)	99.92	0.23
T2103096352	ORT-AF10-14-O-BH	Oral-tongue coat	Female	Adult	Blood-BHI (Aerobic)	99.83	0.6
T2103096353	ORT-AF10-15-O-BH	Oral-tongue coat	Female	Adult	Blood-BHI (Aerobic)	100	0
T2103096354	ORT-AF10-16-O-BH	Oral-tongue coat	Female	Adult	Blood-BHI (Aerobic)	99.34	0
T2103096355	ORT-AF10-18-O-BH	Oral-tongue coat	Female	Adult	Blood-BHI (Aerobic)	99.34	0
T2103096356	ORT-AF10-19-O-BH	Oral-tongue coat	Female	Adult	Blood-BHI (Aerobic)	100	0.47
T2103096357	ORT-AF10-20-O-BH	Oral-tongue coat	Female	Adult	Blood-BHI (Aerobic)	100	0
T2103096358	ORT-AF10-21-O-BH	Oral-tongue coat	Female	Adult	Blood-BHI (Aerobic)	98.34	0
T2103096359	ORT-AF10-22-O-BH	Oral-tongue coat	Female	Adult	Blood-BHI (Aerobic)	99.34	0
T2103096360	ORT-AF10-23-O-BH	Oral-tongue coat	Female	Adult	Blood-BHI (Aerobic)	99.32	0.17
T2103096362	ORT-AF10-25-O-BH	Oral-tongue coat	Female	Adult	Blood-BHI (Aerobic)	99.92	0.23
T2103096363	ORT-AF10-26-O-BH	Oral-tongue coat	Female	Adult	Blood-BHI (Aerobic)	99.88	0.15
T2103096366	ODP-AF10-C3	Oral-tooth	Female	Adult	BHI (Anaerobic)	98.46	0
T2103096369	ODP-AF10-C6	Oral-tooth	Female	Adult	BHI (Anaerobic)	99	0.09
T2103096370	ODP-AF10-C7	Oral-tooth	Female	Adult	BHI (Anaerobic)	100	0
T2103096374	ODP-AF10-C11	Oral-tooth	Female	Adult	BHI (Anaerobic)	100	0
T2103096376	ODP-AF10-C13	Oral-tooth	Female	Adult	BHI (Anaerobic)	100	0
T2103096378	ODP-AF10-C16	Oral-tooth	Female	Adult	BHI (Anaerobic)	100	0
T2103096379	ODP-AF10-C17	Oral-tooth	Female	Adult	BHI (Anaerobic)	100	0.47
T2103096381	ODP-AF10-C20	Oral-tooth	Female	Adult	BHI (Anaerobic)	100	0.47
T2103096382	ODP-AF10-C21	Oral-tooth	Female	Adult	BHI (Anaerobic)	99.82	0.28
T2103096383	ODP-AF10-C22	Oral-tooth	Female	Adult	BHI (Anaerobic)	99.53	0.47

T2103096384	ODP-AF10-C23	Oral-tooth	Female	Adult	BHI (Anaerobic)	99.82	0.2
T2103096385	ORT-AF10-C1	Oral-tongue coat	Female	Adult	BHI (Anaerobic)	99.82	1.13
T2103096389	ORT-AF10-C6	Oral-tongue coat	Female	Adult	BHI (Anaerobic)	98.92	1.1
T2103096391	ORT-AF10-C8	Oral-tongue coat	Female	Adult	BHI (Anaerobic)	100	0.47
T2103096393	ORT-AF10-C11	Oral-tongue coat	Female	Adult	BHI (Anaerobic)	99.87	0.2
T2103096394	ORT-AF10-C12	Oral-tongue coat	Female	Adult	BHI (Anaerobic)	99.87	0.2
T2103096395	ORT-AF10-C13	Oral-tongue coat	Female	Adult	BHI (Anaerobic)	99.49	0.68
T2103096397	ORT-AF10-C15	Oral-tongue coat	Female	Adult	BHI (Anaerobic)	98.92	0.99
T2103096401	ORS-AF10-C5	Oral- saliva	Female	Adult	BHI (Anaerobic)	99.76	0.47
T2103096403	ORS-AF10-C8	Oral- saliva	Female	Adult	BHI (Anaerobic)	99.82	0.2
T2103096404	ORS-AF10-C9	Oral- saliva	Female	Adult	BHI (Anaerobic)	100	0.47
T2103096405	ORS-AF10-C11	Oral- saliva	Female	Adult	BHI (Anaerobic)	100	0
T2103096406	ORS-AF10-C12	Oral- saliva	Female	Adult	BHI (Anaerobic)	99.82	0.54
T2103096407	ORS-AF10-C13	Oral- saliva	Female	Adult	BHI (Anaerobic)	99.82	0.54
T2103096409	ORS-AF10-C15	Oral- saliva	Female	Adult	BHI (Anaerobic)	99.66	0.07
T2103096412	ORS-AF10-C18	Oral- saliva	Female	Adult	BHI (Anaerobic)	100	0.17
T2103096413	ORS-AF10-C19	Oral- saliva	Female	Adult	BHI (Anaerobic)	99.74	0.46
T2103096414	ORS-AF10-C20	Oral- saliva	Female	Adult	BHI (Anaerobic)	99.74	0.46
T2103096415	ORS-AF10-1-BH	Oral- saliva	Female	Adult	Blood-BHI (Anaerobic)	100	0
T2103096416	ORS-AF10-2-BH	Oral- saliva	Female	Adult	Blood-BHI (Anaerobic)	100	0
T2103096417	ORS-AF10-3-BH	Oral- saliva	Female	Adult	Blood-BHI (Anaerobic)	100	0
T2103096419	ORS-AF10-5-BH	Oral- saliva	Female	Adult	Blood-BHI (Anaerobic)	99.82	1.13
T2103096421	ORS-AF10-7-BH	Oral- saliva	Female	Adult	Blood-BHI (Anaerobic)	99.82	0.28
T2103096422	ORS-AF10-8-BH	Oral- saliva	Female	Adult	Blood-BHI (Anaerobic)	99.82	1.13
T2103096423	ORS-AF10-9-BH	Oral- saliva	Female	Adult	Blood-BHI (Anaerobic)	99.78	0.95
T2103096424	ORS-AF10-10-BH	Oral- saliva	Female	Adult	Blood-BHI (Anaerobic)	99.74	0.46
T2103096425	ORS-AF10-11-BH	Oral- saliva	Female	Adult	Blood-BHI (Anaerobic)	99.74	0.46
T2103096426	ORS-AF10-12-BH	Oral- saliva	Female	Adult	Blood-BHI (Anaerobic)	99.45	0
T2103096427	ORS-AF10-13-BH	Oral- saliva	Female	Adult	Blood-BHI (Anaerobic)	99.82	1.13
T2103096428	ORS-AF10-14-BH	Oral- saliva	Female	Adult	Blood-BHI (Anaerobic)	99.45	0
T2103096436	ORS-AF10-22-BH	Oral- saliva	Female	Adult	Blood-BHI (Anaerobic)	99.87	0.2
T2103096438	ORS-AF10-24-BH	Oral- saliva	Female	Adult	Blood-BHI (Anaerobic)	99.45	0.46
T2103096330	ODP-AF10-20-O-BH	Oral-tooth	Female	Adult	Blood-BHI (Aerobic)	98.34	0
T2103096333	ODP-AF10-25-O-BH	Oral-tooth	Female	Adult	Blood-BHI (Aerobic)	99.87	0
T2103096329	ODP-AF10-19-O-BH	Oral-tooth	Female	Adult	Blood-BHI (Aerobic)	100	1.07
T2103096337	ODP-AF10-31-O-BH	Oral-tooth	Female	Adult	Blood-BHI (Aerobic)	99.63	0
T2103096336	ODP-AF10-30-O-BH	Oral-tooth	Female	Adult	Blood-BHI (Aerobic)	100	0.47
T2103096328	ODP-AF10-18-O-BH	Oral-tooth	Female	Adult	Blood-BHI (Aerobic)	99.92	0.23
T2103096346	ORT-AF10-07-O-BH	Oral-tongue coat	Female	Adult	Blood-BHI (Aerobic)	99.82	0.25
T2103096339	ODP-AF10-33-O-BH	Oral-tooth	Female	Adult	Blood-BHI (Aerobic)	100	0.47
T2103096344	ORT-AF10-05-O-BH	Oral-tongue coat	Female	Adult	Blood-BHI (Aerobic)	99.82	0.2
T2103096332	ODP-AF10-23-O-BH	Oral-tooth	Female	Adult	Blood-BHI (Aerobic)	99.34	0
T2103096331	ODP-AF10-21-O-BH	Oral-tooth	Female	Adult	Blood-BHI (Aerobic)	99.34	0
T2103096347	ORT-AF10-08-O-BH	Oral-tongue coat	Female	Adult	Blood-BHI (Aerobic)	100	0.58
T2102085873	ORS-AF08-6	Oral- saliva	Female	Adult	MPYG Medium (Anaerobic)	99.96	0.15
T2102085868	ORS-AF08-1	Oral- saliva	Female	Adult	MPYG Medium (Anaerobic)	99.2	0
T2102085869	ORS-AF08-2	Oral- saliva	Female	Adult	MPYG Medium (Anaerobic)	99.25	0.15
T2102085875	ORS-AF08-8	Oral- saliva	Female	Adult	MPYG Medium (Anaerobic)	100	0
T2102085876	ORS-AF08-9	Oral- saliva	Female	Adult	MPYG Medium (Anaerobic)	99.96	0.15
T2102085872	ORS-AF08-5	Oral- saliva	Female	Adult	MPYG Medium (Anaerobic)	99.49	0.17
T2102085931	ORS-AF08-10	Oral- saliva	Female	Adult	MPYG Medium (Anaerobic)	99.87	0.24
T2102085870	ORS-AF08-3	Oral- saliva	Female	Adult	MPYG Medium (Anaerobic)	100	0.07
T2102085936	ORS-AF08-15	Oral- saliva	Female	Adult	MPYG Medium (Anaerobic)	99.9	0.9
T2102085937	ORS-AF08-16	Oral- saliva	Female	Adult	MPYG Medium (Anaerobic)	100	0.37
T2102085935	ORS-AF08-14	Oral- saliva	Female	Adult	MPYG Medium (Anaerobic)	99.82	0.2
T2102085940	ORS-AF08-19	Oral- saliva	Female	Adult	MPYG Medium (Anaerobic)	99.82	0.2
T2102085938	ORS-AF08-17	Oral- saliva	Female	Adult	MPYG Medium (Anaerobic)	99.82	0.27
T2102085932	ORS-AF08-11	Oral- saliva	Female	Adult	MPYG Medium (Anaerobic)	99.32	0
T2102085939	ORS-AF08-18	Oral- saliva	Female	Adult	MPYG Medium (Anaerobic)	99	0.09
T2102085934	ORS-AF08-13	Oral- saliva	Female	Adult	MPYG Medium (Anaerobic)	99.82	0.2

Supplementary Table 2. The taxonomic information of 1,089 genomes in COGR.

Genome ID	Strain ID	Ez_type match	Similarity(%)	Phylum	GTDB genus	GTDB species('0' means no match)	Cluster id
BF3KA08004A	ORS-AF03-140	Enterococcus_casseliflavus	99.79	Bacillota	Enterococcus_D	Enterococcus_D casseliflavus	1
BF3KA09001A	ORS-AF04-126	Atopobium_parvulum	99.72	Actinomycetota	Lancefieldella	0	2
BF3KA09002A	ORS-AF04-178	Atopobium_parvulum	99.72	Actinomycetota	Lancefieldella	0	2
BF3KA09004	ORS-AF04-35	Streptococcus_sanguinis	99.86	Bacillota	Streptococcus	Streptococcus sanguinis_H	3
BF3KA09004A	ORS-AF04-180	Atopobium_parvulum	99.65	Actinomycetota	Lancefieldella	0	2
BF3KA09005	ORS-AF04-36	Streptococcus_salivarius	99.86	Bacillota	Streptococcus	Streptococcus salivarius	4
BF3KA09008A	ORS-AF04-232	Streptococcus_oralis	98.84	Bacillota	Streptococcus	Streptococcus xiaochunlingii	5
BF3KA09009A	ORS-AF04-233	Streptococcus_parasanguinis	99.37	Bacillota	Streptococcus	Streptococcus sp900766505	6
BF3KA09011A	ORS-AF04-252	Streptococcus_mitis	99.45	Bacillota	Streptococcus	0	7
BF3KA10007A	ORS-AF05-07	#N/A	#N/A	Bacillota	Streptococcus	0	8
BF3KA10010A	ORS-AF05-10	Streptococcus_salivarius	100.00	Bacillota	Streptococcus	Streptococcus salivarius	4
BF3KA10011A	ORS-AF05-11	Streptococcus_mitis	99.45	Bacillota	Streptococcus	0	9
BF3KA10012A	ORS-AF05-12	Streptococcus_salivarius	100.00	Bacillota	Streptococcus	Streptococcus salivarius	4
BF3KA10014A	ORS-AF05-220	Oribacterium_parvum	99.10	Bacillota	Oribacterium	0	10
BF3KA11001	ORS-AM04-31	Streptococcus_salivarius	99.86	Bacillota	Streptococcus	Streptococcus salivarius	4
BF3KA12001	ORS-AM05-116	Microbacterium_oxydans	99.79	Actinomycetota	Microbacterium	Microbacterium algeriense	12
BF3KA12001AB	ORS-AM05-183	Streptococcus_mitis	99.76	Bacillota	Streptococcus	Streptococcus mitis_AZ	11
BF3KA12001B	ORS-AM05-155	Streptococcus_salivarius	94.62	Bacillota	Streptococcus	Streptococcus salivarius	4
BF3KA12002	ORS-AM05-117	Rothia_dentocariosa	99.93	Actinomycetota	Rothia	Rothia dentocariosa	13
BF3KA12003A	ORS-AM05-344	Streptococcus_rubneri	99.51	Bacillota	Streptococcus	Streptococcus rubneri	14
BF3KA12004B	ORS-AM05-325	Neisseria_flavescens	99.04	Pseudomonadota	Neisseria	Neisseria flavescens_B	15
BF3KA12005AB	ORS-AM05-355	Streptococcus_rubneri	99.51	Bacillota	Streptococcus	Streptococcus rubneri	14
BF3KA12006A	ORS-AM05-478	Streptococcus_timonensis	99.86	Bacillota	Streptococcus	Streptococcus mitis_BG	16
BF3KA12007AB	ORS-AM05-484	Streptococcus_salivarius	100.00	Bacillota	Streptococcus	Streptococcus salivarius	4
BF3KA13001A	ORS-TM06-82	Gemella_sanguinis	99.86	Bacillota	Gemella	Gemella sanguinis	17
BF3KA13002A	ORS-TM06-83	Granulicatella_adiacens	99.72	Bacillota	Granulicatella	0	18
BF3KA13003A	ORS-TM06-84	Streptococcus_salivarius	99.86	Bacillota	Streptococcus	Streptococcus salivarius	4
BF3KA13009A	ORS-TM06-215	Veillonella_dispar	99.66	Bacillota	Veillonella	0	19
BF3KA13013A	ORS-TM06-219	Streptococcus_parasanguinis	99.79	Bacillota	Streptococcus	Streptococcus parasanguinis	20
BF3KA14002A	ORS-TM07-02	Enterococcus_faecalis	100.00	Bacillota	Enterococcus	Enterococcus faecalis	21
BF3KA14002B	ORS-TM07-230	Streptococcus_salivarius	97.68	Bacillota	Streptococcus	Streptococcus salivarius	4
BF3KA14003	ORS-TM07-215	Streptococcus_sinensis	98.65	Bacillota	Streptococcus	Streptococcus sinensis	24
BF3KA14003A	ORS-TM07-03	Granulicatella_adiacens	99.86	Bacillota	Granulicatella	Granulicatella adiacens	23
BF3KA14003AB	ORS-TM07-38	Streptococcus_parasanguinis	98.84	Bacillota	Streptococcus	0	22
BF3KA14004AB	ORS-TM07-39	Streptococcus_rubneri	99.86	Bacillota	Streptococcus	Streptococcus rubneri	14
BF3KA15001AB	ORS-AM08-50	Streptococcus_parasanguinis	99.31	Bacillota	Streptococcus	Streptococcus sp001813295	6
BF3KA15002A	ORS-AM08-24	Granulicatella_adiacens	99.72	Bacillota	Granulicatella	0	25
BF3KA15002AB	ORS-AM08-51	Granulicatella_adiacens	99.72	Bacillota	Granulicatella	Granulicatella sp001058355	18
BF3KA15004AB	ORS-AM08-167	Atopobium_parvulum	99.58	Actinomycetota	Lancefieldella	0	26
BF3KC09003A	ODP-AF04-151	Granulicatella_adiacens	99.92	Bacillota	Granulicatella	Granulicatella adiacens	23
BF3KC09004A	ODP-AF04-152	Granulicatella_adiacens	98.22	Bacillota	Granulicatella	Granulicatella adiacens	23
BF3KC09005	ODP-AF04-71	Streptococcus_salivarius	99.86	Bacillota	Streptococcus	Streptococcus salivarius	4
BF3KC09006A	ODP-AF04-188	Actinomyces_odontolyticus	98.86	Actinomycetota	Pauljensenia	Pauljensenia odontolytica_A	27
BF3KC09008	ODP-AF04-74	Rothia_dentocariosa	100.00	Actinomycetota	Rothia	Rothia dentocariosa	13
BF3KC09008A	ODP-AF04-238	Streptococcus_sanguinis	99.86	Bacillota	Streptococcus	Streptococcus sanguinis_H	3
BF3KC11001	ODP-AM04-38	Streptococcus_salivarius	99.86	Bacillota	Streptococcus	Streptococcus salivarius	4
BF3KC11001A	ODP-AM04-93	Prevotella_histicola	99.62	Bacteroidota	Prevotella	Prevotella histicola	28
BF3KC12001A	ODP-AM05-237	Streptococcus_salivarius	100.00	Bacillota	Streptococcus	Streptococcus salivarius	4
BF3KC12001AB	ODP-AM05-254	Streptococcus_sanguinis	99.86	Bacillota	Streptococcus	Streptococcus sanguinis_G	29
BF3KC12002	ODP-AM05-18	Rothia_dentocariosa	99.93	Actinomycetota	Rothia	Rothia dentocariosa	13
BF3KC12002AB	ODP-AM05-255	Granulicatella_adiacens	99.72	Bacillota	Granulicatella	Granulicatella sp905371865	30
BF3KC12003	ODP-AM05-19	Streptococcus_oralis	99.72	Bacillota	Streptococcus	Streptococcus oralis_B	31
BF3KC12004B	ODP-AM05-113	Rothia_aeria	99.86	Actinomycetota	Rothia	Rothia aeria	32
BF3KC12006	ODP-AM05-22	Granulicatella_adiacens	99.79	Bacillota	Granulicatella	Granulicatella adiacens	23
BF3KC12006A	ODP-AM05-411	Actinomyces_naeslundii	98.58	Actinomycetota	Actinomyces	Actinomyces naeslundii	33
BF3KC13001A	ODP-TM06-143	Streptococcus_salivarius	100.00	Bacillota	Streptococcus	Streptococcus salivarius	4
BF3KC13003A	ODP-TM06-145	Granulicatella_adiacens	95.51	Bacillota	Granulicatella	Granulicatella adiacens	23
BF3KC14001	ODP-TM07-197	Microbacterium_oxydans	99.79	Actinomycetota	Microbacterium	Microbacterium algeriense	12
BF3KC14001A	ODP-TM07-115	Granulicatella_adiacens	99.93	Bacillota	Granulicatella	Granulicatella adiacens	23
BF3KC14001AB	ODP-TM07-145	Granulicatella_adiacens	98.64	Bacillota	Granulicatella	Granulicatella adiacens	34
BF3KC14001B	ODP-TM07-209	Streptococcus_oralis	99.04	Bacillota	Streptococcus	Streptococcus oralis_T	35
BF3KC14002	ODP-TM07-198	Streptococcus_cristatus	99.72	Bacillota	Streptococcus	Streptococcus cristatus	36
BF3KC14003B	ODP-TM07-325	Microbacterium_oxydans	99.79	Actinomycetota	Microbacterium	Microbacterium algeriense	12
BF3KC15001A	ODP-AM08-279	Streptococcus_salivarius	100.00	Bacillota	Streptococcus	Streptococcus salivarius	4
BF3KC15002A	ODP-AM08-280	Granulicatella_adiacens	99.72	Bacillota	Granulicatella	0	25
BF3KC15004A	ODP-AM08-282	Abiotrophia_defectiva	98.24	Bacillota	Abiotrophia	Abiotrophia defectiva	37
BF3KC15004C	ODP-AM08-246	Rothia_dentocariosa	99.93	Actinomycetota	Rothia	Rothia dentocariosa	13
BF3KC15006A	ODP-AM08-284	Actinomyces_odontolyticus	99.57	Actinomycetota	Pauljensenia	Pauljensenia odontolytica	38
BF3KC15011A	ODP-AM08-377	Actinomyces_naeslundii	99.59	Actinomycetota	Actinomyces	Actinomyces naeslundii	33
BF3KC15013A	ODP-AM08-379	Actinomyces_naeslundii	99.59	Actinomycetota	Actinomyces	Actinomyces naeslundii	33
BF3KC15015A	ODP-AM08-381	Actinomyces_naeslundii	96.90	Actinomycetota	Actinomyces	Actinomyces oris	39
BF3KC15016A	ODP-AM08-382	Actinomyces_naeslundii	97.84	Actinomycetota	Actinomyces	Actinomyces naeslundii	33
BF3KC15020A	ODP-AM08-386	Streptococcus_sanguinis	99.65	Bacillota	Streptococcus	0	40
BF3KC16005	ODP-AF06-100	Microbacterium_oxydans	99.79	Actinomycetota	Microbacterium	Microbacterium algeriense	12
BF3KT08002	ORT-AF03-65	Rothia_mucilaginosa	99.58	Actinomycetota	Rothia	Rothia mucilaginosa_A	41
BF3KT08005	ORT-AF03-68	Gemella_sanguinis	99.93	Bacillota	Gemella	Gemella sanguinis	17
BF3KT09001	ORT-AF04-01	Streptococcus_salivarius	99.86	Bacillota	Streptococcus	Streptococcus salivarius	4
BF3KT09001A	ORT-AF04-115	Atopobium_parvulum	99.72	Actinomycetota	Lancefieldella	0	2
BF3KT09002A	ORT-AF04-116	Streptococcus_parasanguinis	98.91	Bacillota	Streptococcus	Streptococcus sp900766505	6
BF3KT09003A	ORT-AF04-169	Actinomyces_odontolyticus	99.69	Actinomycetota	Pauljensenia	Pauljensenia sp001064145	42
BF3KT09005A	ORT-AF04-215	Lactobacillus_paracasei	98.39	Bacillota	Lacticaseibacillus	Lacticaseibacillus paracasei	43
BF3KT09006A	ORT-AF04-216	Streptococcus_parasanguinis	98.91	Bacillota	Streptococcus	Streptococcus sp900766505	6
BF3KT09007A	ORT-AF04-246	Streptococcus_mitis	99.45	Bacillota	Streptococcus	0	7

BF3KT11001	ORT-AM04-01	Streptococcus_salivarius	99.86	Bacillota	Streptococcus	Streptococcus salivarius	4
BF3KT11001A	ORT-AM04-53	Streptococcus_salivarius	100.00	Bacillota	Streptococcus	Streptococcus salivarius	4
BF3KT11003A	ORT-AM04-76	Granulicatella_adiacens	98.22	Bacillota	Granulicatella	Granulicatella adiacens	23
BF3KT12001B	ORT-AM05-52	Neisseria_macacae	99.52	Pseudomonadota	Neisseria	Neisseria sicca_A	44
BF3KT12005	ORT-AM05-291	Streptococcus_salivarius	100.00	Bacillota	Streptococcus	Streptococcus salivarius	4
BF3KT12005AB	ORT-AM05-406	Actinomyces_odontolyticus	99.08	Actinomycetota	Pauljensenia	Pauljensenia sp902373545	45
BF3KT12006A	ORT-AM05-398	Solobacterium_moorei	99.44	Bacillota	Bulleidia	0	47
BF3KT12006AB	ORT-AM05-453	Streptococcus_constellatus	99.12	Bacillota	Streptococcus	Streptococcus constellatus	46
BF3KT12007A	ORT-AM05-445	Granulicatella_adiacens	98.36	Bacillota	Granulicatella	0	25
BF3KT12008A	ORT-AM05-446	Streptococcus_australis	99.52	Bacillota	Streptococcus	Streptococcus koreensis	48
BF3KT13002	ORT-TM06-63	Gemella_sanguinis	99.86	Bacillota	Gemella	Gemella sanguinis	17
BF3KT14001	ORT-TM07-175	Gemella_haemolysans	99.93	Bacillota	Gemella	Gemella haemolysans_B	49
BF3KT14001A	ORT-TM07-80	Granulicatella_adiacens	99.68	Bacillota	Granulicatella	0	25
BF3KT14001B	ORT-TM07-301	Microbacterium_oxydans	99.79	Actinomycetota	Microbacterium	Microbacterium algeriense	12
BF3KT14002AB	ORT-TM07-98	Granulicatella_adiacens	99.72	Bacillota	Granulicatella	Granulicatella sp001058355	18
BF3KT14003AB	ORT-TM07-99	Streptococcus_parasanguinis	98.84	Bacillota	Streptococcus	0	22
BF3KT15001	ORT-AM08-194	Streptococcus_salivarius	99.93	Bacillota	Streptococcus	Streptococcus salivarius	4
BF3KT15002	ORT-AM08-195	Streptococcus_oralis	99.86	Bacillota	Streptococcus	0	50
BF3KT15003B	ORT-AM08-219	Lactococcus_garvieae	100.00	Bacillota	Lactococcus	Lactococcus garvieae	51
BF3KT16001	ORT-AF06-01	Microbacterium_oxydans	99.79	Actinomycetota	Microbacterium	Microbacterium algeriense	12
GF3KA08002B	ORS-AF03-49	Actinomyces_naeslundii	97.64	Actinomycetota	Actinomyces	Actinomyces oris	39
GF3KA09001A	ORS-AF04-127	Streptococcus_cristatus	99.72	Bacillota	Streptococcus	Streptococcus cristatus_C	52
GF3KA09003A	ORS-AF04-129	Streptococcus_parasanguinis	99.84	Bacillota	Streptococcus	Streptococcus parasanguinis	20
GF3KA09004	ORS-AF04-41	Streptococcus_sanguinis	99.79	Bacillota	Streptococcus	Streptococcus sanguinis_H	3
GF3KA09004A	ORS-AF04-183	Atopobium_parvulum	99.72	Actinomycetota	Lancefieldella	0	2
GF3KA09005	ORS-AF04-42	Streptococcus_mitis	99.45	Bacillota	Streptococcus	0	9
GF3KA09006A	ORS-AF04-234	Streptococcus_parasanguinis	98.50	Bacillota	Streptococcus	Streptococcus parasanguinis_C	53
GF3KA10002A	ORS-AF05-14	Streptococcus_salivarius	99.86	Bacillota	Streptococcus	Streptococcus salivarius	4
GF3KA10003A	ORS-AF05-15	Streptococcus_salivarius	99.85	Bacillota	Streptococcus	Streptococcus salivarius	4
GF3KA10004A	ORS-AF05-16	Streptococcus_salivarius	99.86	Bacillota	Streptococcus	Streptococcus salivarius	4
GF3KA10006	ORS-AF05-242	Streptococcus_salivarius	99.86	Bacillota	Streptococcus	Streptococcus salivarius	4
GF3KA10007A	ORS-AF05-19	Gemella_haemolysans	98.03	Bacillota	Gemella	Gemella haemolysans_B	49
GF3KA11002A	ORS-AM04-85	Granulicatella_adiacens	93.51	Bacillota	Granulicatella	Granulicatella adiacens	23
GF3KA12001	ORS-AM05-121	Streptococcus_salivarius	94.62	Bacillota	Streptococcus	Streptococcus salivarius	4
GF3KA12002	ORS-AM05-122	Streptococcus_oralis	99.72	Bacillota	Streptococcus	Streptococcus oralis_B	31
GF3KA12004	ORS-AM05-124	Corynebacterium_argentoratense	99.79	Actinomycetota	Corynebacterium	Corynebacterium argentoratense	54
GF3KA12006AB	ORS-AM05-357	Streptococcus_parasanguinis	97.25	Bacillota	Streptococcus	0	22
GF3KA12007AB	ORS-AM05-485	Streptococcus_rubneri	99.51	Bacillota	Streptococcus	Streptococcus rubneri	14
GF3KA13001A	ORS-TM06-89	Streptococcus_cristatus	99.65	Bacillota	Streptococcus	Streptococcus cristatus	36
GF3KA13002	ORS-TM06-15	Streptococcus_australis	98.70	Bacillota	Streptococcus	Streptococcus xiaochunlingii	56
GF3KA13002A	ORS-TM06-90	Abiotrophia_defectiva	99.45	Bacillota	Abiotrophia	Abiotrophia sp001815865	55
GF3KA13003	ORS-TM06-16	Actinomyces_naeslundii	96.15	Actinomycetota	Actinomyces	Actinomyces oris_A	58
GF3KA13003A	ORS-TM06-91	Granulicatella_elegans	100.00	Bacillota	Granulicatella	0	57
GF3KA13005A	ORS-TM06-93	Granulicatella_adiacens	99.72	Bacillota	Granulicatella	0	18
GF3KA13006	ORS-TM06-240	Rothia_mucilaginosa	99.37	Actinomycetota	Rothia	Rothia mucilaginosa_B	59
GF3KA13006A	ORS-TM06-94	Granulicatella_elegans	100.00	Bacillota	Granulicatella	0	57
GF3KA13011A	ORS-TM06-222	Streptococcus_salivarius	100.00	Bacillota	Streptococcus	Streptococcus salivarius	4
GF3KA14001AB	ORS-TM07-40	Enterococcus_faecalis	100.00	Bacillota	Enterococcus	Enterococcus faecalis	21
GF3KA15001A	ORS-AM08-28	Streptococcus_australis	99.38	Bacillota	Streptococcus	0	60
GF3KA15003A	ORS-AM08-30	Atopobium_parvulum	99.58	Actinomycetota	Lancefieldella	0	26
GF3KA15005AB	ORS-AM08-168	Streptococcus_parasanguinis	99.86	Bacillota	Streptococcus	Streptococcus parasanguinis	20
GF3KA16003	ORS-AF06-69	Streptococcus_anginosus	100.00	Bacillota	Streptococcus	Streptococcus anginosus	61
GF3KC09002A	ODP-AF04-155	Granulicatella_adiacens	99.90	Bacillota	Granulicatella	Granulicatella adiacens	23
GF3KC09003	ODP-AF04-78	Rothia_mucilaginosa	99.51	Actinomycetota	Rothia	Rothia mucilaginosa_A	41
GF3KC09005A	ODP-AF04-158	Granulicatella_adiacens	93.62	Bacillota	Granulicatella	Granulicatella adiacens	23
GF3KC09008A	ODP-AF04-190	Actinomyces_odontolyticus	99.15	Actinomycetota	Pauljensenia	Pauljensenia odontolytica_A	27
GF3KC10001A	ODP-AF05-68	Streptococcus_parasanguinis	99.04	Bacillota	Streptococcus	Streptococcus parasanguinis_C	53
GF3KC10002A	ODP-AF05-69	Streptococcus_parasanguinis	99.04	Bacillota	Streptococcus	Streptococcus parasanguinis_C	53
GF3KC11001A	ODP-AM04-97	Prevotella_veroralis	97.93	Bacteroidota	Prevotella	Prevotella sp000257925	62
GF3KC11002A	ODP-AM04-98	Prevotella_histicola	99.86	Bacteroidota	Prevotella	Prevotella histicola	28
GF3KC11003A	ODP-AM04-99	Atopobium_parvulum	99.72	Actinomycetota	Lancefieldella	0	2
GF3KC11005A	ODP-AM04-101	Atopobium_parvulum	99.65	Actinomycetota	Lancefieldella	0	2
GF3KC12001	ODP-AM05-24	Streptococcus_gordonii	99.93	Bacillota	Streptococcus	Streptococcus gordonii	63
GF3KC12002	ODP-AM05-25	Streptococcus_mitis	99.79	Bacillota	Streptococcus	Streptococcus mitis_AZ	11
GF3KC12002A	ODP-AM05-415	Streptococcus_sanguinis	99.86	Bacillota	Streptococcus	Streptococcus sanguinis_G	29
GF3KC12002B	ODP-AM05-306	Neisseria_elongata	99.58	Pseudomonadota	Neisseria	Neisseria elongata	64
GF3KC12004B	ODP-AM05-321	Neisseria_cinerea	98.56	Pseudomonadota	Neisseria	Neisseria subflava	15
GF3KC12005AB	ODP-AM05-388	Gemella_morbillorum	98.30	Bacillota	Gemella	Gemella morbillorum	65
GF3KC12006A	ODP-AM05-419	Actinomyces_odontolyticus	99.29	Actinomycetota	Pauljensenia	Pauljensenia odontolytica_A	27
GF3KC12007A	ODP-AM05-420	Actinomyces_johnsonii	99.85	Actinomycetota	Actinomyces	Actinomyces johnsonii	66
GF3KC12007AB	ODP-AM05-461	Actinomyces_johnsonii	99.09	Actinomycetota	Actinomyces	Actinomyces johnsonii	66
GF3KC12010A	ODP-AM05-423	Leptotrichia_buccalis<br>/Lactococcus lactis	98.56/100	Fusobacteriota	Leptotrichia	Leptotrichia massiliensis	67
GF3KC12011A	ODP-AM05-501	Streptococcus_sanguinis	99.86	Bacillota	Streptococcus	Streptococcus sanguinis_G	29
GF3KC13001A	ODP-TM06-148	Streptococcus_rubneri	96.28	Bacillota	Streptococcus	0	68
GF3KC13002A	ODP-TM06-149	Streptococcus_rubneri	99.68	Bacillota	Streptococcus	0	22
GF3KC13003A	ODP-TM06-150	Streptococcus_rubneri	99.62	Bacillota	Streptococcus	0	68
GF3KC14001A	ODP-TM07-125	Granulicatella_adiacens	98.64	Bacillota	Granulicatella	Granulicatella adiacens	34
GF3KC14002	ODP-TM07-288	Microbacterium_oxydans	99.79	Actinomycetota	Microbacterium	Microbacterium algeriense	12
GF3KC14002AB	ODP-TM07-152	Granulicatella_adiacens	94.34	Bacillota	Granulicatella	Granulicatella adiacens	34
GF3KC15001A	ODP-AM08-289	Granulicatella_adiacens	99.66	Bacillota	Granulicatella	Granulicatella sp905371865	69
GF3KC15002	ODP-AM08-343	Streptococcus_intermedius	100.00	Bacillota	Streptococcus	Streptococcus intermedius	70
GF3KC15004A	ODP-AM08-292	Streptococcus_sanguinis	99.79	Bacillota	Streptococcus	Streptococcus sanguinis_H	3
GF3KC15006B	ODP-AM08-257	Actinomyces_naeslundii	96.96	Actinomycetota	Actinomyces	Actinomyces oris_A	58
GF3KC15009A	ODP-AM08-297	Abiotrophia_defectiva	99.59	Bacillota	Abiotrophia	Abiotrophia defectiva	37
GF3KT09001A	ORT-AF04-117	Streptococcus_parasanguinis	99.04	Bacillota	Streptococcus	Streptococcus parasanguinis_C	53
GF3KT09002A	ORT-AF04-118	Streptococcus_parasanguinis	99.04	Bacillota	Streptococcus	Streptococcus parasanguinis_C	53

GF3KT09003A	ORT-AF04-170	Atopobium_parvulum	99.72	Actinomycetota	Lancefieldella	0	2
GF3KT09005	ORT-AF04-192	Streptococcus_salivarius	99.86	Bacillota	Streptococcus	Streptococcus salivarius	4
GF3KT09005A	ORT-AF04-217	Streptococcus_parasanguinis	99.21	Bacillota	Streptococcus	Streptococcus parasanguinis_D	71
GF3KT10001A	ORT-AF05-103	Streptococcus_parasanguinis	99.84	Bacillota	Streptococcus	Streptococcus parasanguinis	20
GF3KT10002A	ORT-AF05-104	Atopobium_parvulum	99.72	Actinomycetota	Lancefieldella	0	2
GF3KT10003A	ORT-AF05-105	Atopobium_parvulum	99.72	Actinomycetota	Lancefieldella	0	2
GF3KT10004A	ORT-AF05-106	Streptococcus_salivarius	99.86	Bacillota	Streptococcus	Streptococcus salivarius	4
GF3KT10006A	ORT-AF05-108	Streptococcus_salivarius	99.86	Bacillota	Streptococcus	Streptococcus salivarius	4
GF3KT11001	ORT-AM04-05	Streptococcus_infantis	99.52	Bacillota	Streptococcus	0	72
GF3KT11002	ORT-AM04-06	Rothia_mucilaginosa	99.58	Actinomycetota	Rothia	Rothia mucilaginosa_B	59
GF3KT11003	ORT-AM04-07	Streptococcus_infantis	99.52	Bacillota	Streptococcus	Streptococcus infantis_H	73
GF3KT11003A	ORT-AM04-127	#N/A	#N/A	Bacillota	Streptococcus	Streptococcus gordonii	63
GF3KT12001	ORT-AM05-05	Streptococcus_timonensis	99.86	Bacillota	Streptococcus	0	74
GF3KT12002AB	ORT-AM05-94	Streptococcus_mitis	99.79	Bacillota	Streptococcus	Streptococcus mitis_AZ	11
GF3KT12002B	ORT-AM05-110	Rothia_mucilaginosa	99.51	Actinomycetota	Rothia	Rothia mucilaginosa_B	59
GF3KT12003AB	ORT-AM05-95	Lachnoanaerobaculum_umeaense	77.58	Bacillota	Lachnoanaerobaculum	0	75
GF3KT12004A	ORT-AM05-449	Streptococcus_timonensis	99.86	Bacillota	Streptococcus	Streptococcus mitis_BG	16
GF3KT12004AB	ORT-AM05-96	Granulicatella_adiacens	99.66	Bacillota	Granulicatella	0	25
GF3KT12005A	ORT-AM05-450	Streptococcus_australis	98.43	Bacillota	Streptococcus	Streptococcus koreensis	48
GF3KT12006AB	ORT-AM05-288	Granulicatella_adiacens	99.72	Bacillota	Granulicatella	0	25
GF3KT12008A	ORT-AM05-520	Actinomyces_odontolyticus	99.22	Actinomycetota	Pauljensenia	Pauljensenia sp018375675	76
GF3KT12009AB	ORT-AM05-455	Gemella_sanguinis	99.86	Bacillota	Gemella	Gemella sanguinis	17
GF3KT13001A	ORT-TM06-169	Streptococcus_rubneri	96.47	Bacillota	Streptococcus	0	68
GF3KT13002A	ORT-TM06-170	Granulicatella_adiacens	99.59	Bacillota	Granulicatella	0	77
GF3KT13003	ORT-TM06-66	Streptococcus_parasanguinis	99.35	Bacillota	Streptococcus	Streptococcus sp900766505	6
GF3KT14001	ORT-TM07-178	Microbacterium_oxydans	99.79	Actinomycetota	Microbacterium	Microbacterium algeriense	12
GF3KT15003	ORT-AM08-200	Streptococcus_salivarius	99.93	Bacillota	Streptococcus	Streptococcus salivarius	4
GF3KT15004B	ORT-AM08-223	Rothia_mucilaginosa	99.51	Actinomycetota	Rothia	Rothia mucilaginosa	78
GF3KT16003	ORT-AF06-92	Rothia_mucilaginosa	99.58	Actinomycetota	Rothia	Rothia mucilaginosa_B	59
HF3KA08001A	ORS-AF03-141	Streptococcus_mitis	99.18	Bacillota	Streptococcus	Streptococcus oralis_Z	79
HF3KA08001B	ORS-AF03-52	Rothia_dentocariosa	99.93	Actinomycetota	Rothia	Rothia dentocariosa	13
HF3KA08002A	ORS-AF03-142	Enterococcus_casseliflavus	99.79	Bacillota	Enterococcus_D	Enterococcus_D casseliflavus	1
HF3KA08006B	ORS-AF03-251	Actinomyces_naeslundii	96.76	Actinomycetota	Actinomyces	Actinomyces oris	39
HF3KA09001A	ORS-AF04-136	Streptococcus_salivarius	99.86	Bacillota	Streptococcus	Streptococcus salivarius	4
HF3KA09002A	ORS-AF04-137	Streptococcus_salivarius	99.86	Bacillota	Streptococcus	Streptococcus salivarius	4
HF3KA09005A	ORS-AF04-140	Granulicatella_adiacens	99.80	Bacillota	Granulicatella	Granulicatella adiacens	23
HF3KA10004A	ORS-AF05-23	Streptococcus_salivarius	100.00	Bacillota	Streptococcus	Streptococcus salivarius	4
HF3KA10005A	ORS-AF05-24	Streptococcus_salivarius	96.66	Bacillota	Streptococcus	Streptococcus salivarius	4
HF3KA11001A	ORS-AM04-86	Streptococcus_sanguinis	99.79	Bacillota	Streptococcus	Streptococcus sanguinis_H	3
HF3KA12001	ORS-AM05-127	Corynebacterium_argentoratense	99.79	Actinomycetota	Corynebacterium	Corynebacterium argentoratense	54
HF3KA12001A	ORS-AM05-172	Corynebacterium_argentoratense	99.79	Actinomycetota	Corynebacterium	Corynebacterium argentoratense	54
HF3KA12002	ORS-AM05-128	Streptococcus_constellatus	99.12	Bacillota	Streptococcus	Streptococcus constellatus	46
HF3KA12002AB	ORS-AM05-193	#N/A	#N/A	Bacillota	Streptococcus	Streptococcus koreensis	48
HF3KA12003AB	ORS-AM05-194	Streptococcus_mitis	99.72	Bacillota	Streptococcus	Streptococcus mitis_AZ	11
HF3KA12004B	ORS-AM05-167	Corynebacterium_argentoratense	99.79	Actinomycetota	Corynebacterium	Corynebacterium argentoratense	54
HF3KA12005AB	ORS-AM05-196	Rothia_dentocariosa	99.93	Actinomycetota	Rothia	Rothia dentocariosa	13
HF3KA12006A	ORS-AM05-374	Capnocytophaga_leadbetteri	99.09	Bacteroidota	Capnocytophaga	Capnocytophaga leadbetteri	81
HF3KA12006AB	ORS-AM05-358	Fusobacterium_periodonticum	99.71	Fusobacteriota	Fusobacterium	Fusobacterium periodonticum_D	80
HF3KA12009AB	ORS-AM05-381	Solobacterium_moorei	99.51	Bacillota	Bulleidia	Bulleidia sp015256775	82
HF3KA13001	ORS-TM06-19	Rothia_dentocariosa	99.93	Actinomycetota	Rothia	Rothia dentocariosa	13
HF3KA13002A	ORS-TM06-96	Streptococcus_cristatus	99.52	Bacillota	Streptococcus	Streptococcus cristatus_G	83
HF3KA13003A	ORS-TM06-97	Gemella_sanguinis	99.86	Bacillota	Gemella	Gemella sanguinis	17
HF3KA13005A	ORS-TM06-99	Granulicatella_adiacens	99.93	Bacillota	Granulicatella	Granulicatella adiacens	23
HF3KA13006A	ORS-TM06-100	Gemella_haemolysans	99.39	Bacillota	Gemella	0	84
HF3KA13012A	ORS-TM06-224	Streptococcus_salivarius	99.93	Bacillota	Streptococcus	Streptococcus salivarius	4
HF3KA13014A	ORS-TM06-226	Veillonella_dispar	99.73	Bacillota	Veillonella	Veillonella sp902834965	19
HF3KA14001	ORS-TM07-219	Streptococcus_mitis	99.65	Bacillota	Streptococcus	0	85
HF3KA14004A	ORS-TM07-11	Enterococcus_faecalis	100.00	Bacillota	Enterococcus	Enterococcus faecalis	21
HF3KA15001A	ORS-AM08-152	Actinomyces_odontolyticus	98.93	Actinomycetota	Pauljensenia	Pauljensenia sp902373545	45
HF3KA15001AB	ORS-AM08-56	Streptococcus_anginosus	99.66	Bacillota	Streptococcus	Streptococcus anginosus	61
HF3KA15004AB	ORS-AM08-171	Granulicatella_adiacens	99.72	Bacillota	Granulicatella	Granulicatella sp001058355	18
HF3KA15004B	ORS-AM08-101	Rothia_mucilaginosa	99.44	Actinomycetota	Rothia	Rothia mucilaginosa	78
HF3KA16008	ORS-AF06-121	Streptococcus_timonensis	98.84	Bacillota	Streptococcus	0	86
HF3KA16011	ORS-AF06-159	Streptococcus_rubneri	99.38	Bacillota	Streptococcus	Streptococcus xiaochunlingii	5
HF3KC09003	ODP-AF04-83	#N/A	#N/A	Bacillota	Streptococcus	0	8
HF3KC12001AB	ODP-AM05-262	Streptococcus_constellatus	99.12	Bacillota	Streptococcus	Streptococcus constellatus	46
HF3KC12002A	ODP-AM05-425	Fusobacterium_nucleatum	99.93	Fusobacteriota	Fusobacterium	Fusobacterium polymorphum	87
HF3KC12002AB	ODP-AM05-263	Streptococcus_oralis	99.79	Bacillota	Streptococcus	Streptococcus oralis_Y	35
HF3KC12003AB	ODP-AM05-390	Actinomyces_oris/Lactococcus_lactis	99.34/100	Actinomycetota	Actinomyces	Actinomyces oris	39
HF3KC12004AB	ODP-AM05-456	Lachnoanaerobaculum_umeaense	94.53	Bacillota	Lachnoanaerobaculum	Lachnoanaerobaculum gingivalis	88
HF3KC12005A	ODP-AM05-463	Capnocytophaga_sputigena	99.79	Bacteroidota	Capnocytophaga	Capnocytophaga sputigena	90
HF3KC12005AB	ODP-AM05-457	Streptococcus_pseudopneumoniae	99.79	Bacillota	Streptococcus	Streptococcus symci	89
HF3KC12005B	ODP-AM05-322	Rothia_mucilaginosa	99.37	Actinomycetota	Rothia	Rothia sp015265375	91
HF3KC12006AB	ODP-AM05-458	Streptococcus_pseudopneumoniae	99.79	Bacillota	Streptococcus	Streptococcus symci	89
HF3KC12007	ODP-AM05-296	Rothia_dentocariosa	99.93	Actinomycetota	Rothia	Rothia dentocariosa	13
HF3KC13001	ODP-TM06-47	Streptococcus_gordonii	99.66	Bacillota	Streptococcus	Streptococcus gordonii	63
HF3KC13001A	ODP-TM06-151	Streptococcus_salivarius	100.00	Bacillota	Streptococcus	Streptococcus salivarius	4
HF3KC13002A	ODP-TM06-152	Actinomyces_odontolyticus	99.19	Actinomycetota	Pauljensenia	Pauljensenia odontolytica_A	27
HF3KC13003A	ODP-TM06-153	Gemella_haemolysans	99.79	Bacillota	Gemella	0	84
HF3KC13005A	ODP-TM06-155	Granulicatella_adiacens	99.79	Bacillota	Granulicatella	Granulicatella adiacens	23
HF3KC14001AB	ODP-TM07-155	Granulicatella_adiacens	99.93	Bacillota	Granulicatella	Granulicatella adiacens	23
HF3KC14002	ODP-TM07-290	Microbacterium_oxydans	99.79	Actinomycetota	Microbacterium	Microbacterium algeriense	12
HF3KC14003AB	ODP-TM07-157	Veillonella_dispar	99.46	Bacillota	Veillonella	Veillonella parvula_A	92
HF3KC15001AB	ODP-AM08-403	Granulicatella_adiacens	99.79	Bacillota	Granulicatella	Granulicatella adiacens	23
HF3KC15002	ODP-AM08-350	Streptococcus_salivarius	100.00	Bacillota	Streptococcus	Streptococcus salivarius	4
HF3KC15002A	ODP-AM08-302	Streptococcus_salivarius	100.00	Bacillota	Streptococcus	Streptococcus salivarius	4

HF3KC15004A	ODP-AM08-304	Gemella_sanguinis	99.93	Bacillota	Gemella	Gemella sanguinis	17
HF3KC15009A	ODP-AM08-392	Fusobacterium_periodonticum	99.85	Fusobacteriota	Fusobacterium	Fusobacterium periodonticum_D	80
HF3KC15010A	ODP-AM08-393	Gemella_morbillorum	99.86	Bacillota	Gemella	Gemella morbillorum	65
HF3KC16002B	ODP-AF06-108	Streptococcus_mitis	97.07	Bacillota	Streptococcus	Streptococcus oralis_G	93
HF3KT08002	ORT-AF03-76	Gemella_haemolysans	99.66	Bacillota	Gemella	0	94
HF3KT08003A	ORT-AF03-129	Streptococcus_parasanguinis	99.32	Bacillota	Streptococcus	Streptococcus parasanguinis	20
HF3KT09001	ORT-AF04-11	Streptococcus_salivarius	99.86	Bacillota	Streptococcus	Streptococcus salivarius	4
HF3KT09002A	ORT-AF04-173	Prevotella_histicola	99.62	Bacteroidota	Prevotella	Prevotella histicola	28
HF3KT09003A	ORT-AF04-221	Streptococcus_parasanguinis	99.18	Bacillota	Streptococcus	Streptococcus sp900766505	6
HF3KT10001A	ORT-AF05-109	Enterococcus_faecalis	100.00	Bacillota	Enterococcus	Enterococcus faecalis	21
HF3KT10005A	ORT-AF05-113	Granulicatella_adiacens	99.72	Bacillota	Granulicatella	0	95
HF3KT10010A	ORT-AF05-230	Atopobium_parvulum	99.72	Actinomycetota	Lancefieldella	0	96
HF3KT11004	ORT-AM04-12	Gemella_haemolysans	99.39	Bacillota	Gemella	Gemella haemolysans_B	49
HF3KT12001A	ORT-AM05-89	Streptococcus_oralis	99.52	Bacillota	Streptococcus	Streptococcus oralis_Y	35
HF3KT12001B	ORT-AM05-54	Neisseria_macacae	99.52	Pseudomonadota	Neisseria	Neisseria sicca_A	44
HF3KT12002	ORT-AM05-111	Neisseria_cinerea	97.94	Pseudomonadota	Neisseria	Neisseria flavescens_B	15
HF3KT12003	ORT-AM05-314	Neisseria_elongata	99.79	Pseudomonadota	Neisseria	Neisseria elongata	64
HF3KT12003AB	ORT-AM05-235	Granulicatella_adiacens	99.66	Bacillota	Granulicatella	0	25
HF3KT12004	ORT-AM05-320	Corynebacterium_argentoratense	99.79	Actinomycetota	Corynebacterium	Corynebacterium argentoratense	54
HF3KT12004A	ORT-AM05-507	Streptococcus_salivarius	100.00	Bacillota	Streptococcus	Streptococcus salivarius	4
HF3KT12004B	ORT-AM05-317	Neisseria_elongata	99.79	Pseudomonadota	Neisseria	Neisseria elongata	64
HF3KT13001A	ORT-TM06-175	Streptococcus_salivarius	100.00	Bacillota	Streptococcus	Streptococcus salivarius	4
HF3KT13002A	ORT-TM06-176	Granulicatella_adiacens	99.72	Bacillota	Granulicatella	0	18
HF3KT13003A	ORT-TM06-177	Actinomyces_odontolyticus	99.22	Actinomycetota	Pauljensenia	Pauljensenia sp902373545	45
HF3KT14001	ORT-TM07-185	Streptococcus_rubneri	99.51	Bacillota	Streptococcus	0	98
HF3KT14001A	ORT-TM07-82	Actinomyces_odontolyticus	98.93	Actinomycetota	Pauljensenia	Pauljensenia sp018382595	97
HF3KT14002B	ORT-TM07-206	Rothia_mucilaginosa	99.37	Actinomycetota	Rothia	Rothia mucilaginosa_B	59
HF3KT15001AB	ORT-AM08-461	Granulicatella_adiacens	99.72	Bacillota	Granulicatella	Granulicatella sp001058355	18
HF3KT16001	ORT-AF06-04	Streptococcus_australis	98.57	Bacillota	Streptococcus	Streptococcus koreensis	48
HF3KT16004	ORT-AF06-93	Microbacterium_oxydans	99.79	Actinomycetota	Microbacterium	Microbacterium algeriense	12
HF3KT16005	ORT-AF06-141	Rothia_mucilaginosa	99.44	Actinomycetota	Rothia	Rothia sp001808955	78
IF3KA08003BA	ORS-AF03-170	Streptococcus_mitis	99.65	Bacillota	Streptococcus	Streptococcus mitis_AI	89
IF3KA09001BA	ORS-AF04-147	Actinomyces_odontolyticus	99.39	Actinomycetota	Pauljensenia	Pauljensenia sp001064145	42
IF3KA09003BA	ORS-AF04-237	Actinomyces_odontolyticus	99.08	Actinomycetota	Pauljensenia	Pauljensenia sp001064145	42
IF3KA09004A	ORS-AF04-133	Atopobium_parvulum	99.72	Actinomycetota	Lancefieldella	0	2
IF3KA09005A	ORS-AF04-134	Atopobium_parvulum	99.72	Actinomycetota	Lancefieldella	0	2
IF3KA11001	ORS-AM04-35	Streptococcus_salivarius	100.00	Bacillota	Streptococcus	Streptococcus salivarius	4
IF3KA12001AB	ORS-AM05-197	#N/A	#N/A	Bacillota	Streptococcus	0	85
IF3KA12002AB	ORS-AM05-198	Fusobacterium_periodonticum	99.85	Fusobacteriota	Fusobacterium	Fusobacterium pseudoperiodonticum	80
IF3KA12002B	ORS-AM05-342	Staphylococcus_hominis	99.72	Bacillota	Staphylococcus	Staphylococcus hominis	99
IF3KA12003	ORS-AM05-133	Streptococcus_mitis	99.48	Bacillota	Streptococcus	0	100
IF3KA12003A	ORS-AM05-177	Streptococcus_constellatus	99.12	Bacillota	Streptococcus	Streptococcus constellatus	46
IF3KA12004AB	ORS-AM05-382	Lactococcus_lactis /Actinomyces_odontolyticus	100/99.27	Actinomycetota	Pauljensenia	Pauljensenia sp902373545	45
IF3KA12007A	ORS-AM05-481	Streptococcus_pseudopneumoniae	96.86	Bacillota	Streptococcus	0	101
IF3KA12008AB	ORS-AM05-490	Streptococcus_rubneri	99.51	Bacillota	Streptococcus	Streptococcus rubneri	14
IF3KA12009AB	ORS-AM05-491	Streptococcus_oralis	99.79	Bacillota	Streptococcus	Streptococcus oralis_C	35
IF3KA13001	ORS-TM06-24	Streptococcus_salivarius	99.93	Bacillota	Streptococcus	Streptococcus salivarius	4
IF3KA13001B	ORS-TM06-04	Rothia_mucilaginosa	99.37	Actinomycetota	Rothia	Rothia mucilaginosa_B	59
IF3KA13002A	ORS-TM06-104	Abiotrophia_defectiva	99.86	Bacillota	Abiotrophia	Abiotrophia defectiva	37
IF3KA13002B	ORS-TM06-05	Streptococcus_anginosus	97.09	Bacillota	Streptococcus	Streptococcus anginosus	61
IF3KA13003A	ORS-TM06-105	Streptococcus_oralis	99.45	Bacillota	Streptococcus	0	102
IF3KA13003B	ORS-TM06-06	Streptococcus_gordonii	99.66	Bacillota	Streptococcus	Streptococcus gordonii	63
IF3KA13004	ORS-TM06-27	Streptococcus_gordonii	99.66	Bacillota	Streptococcus	Streptococcus gordonii	63
IF3KA13004A	ORS-TM06-106	Granulicatella_adiacens	99.72	Bacillota	Granulicatella	Granulicatella sp015264885	103
IF3KA13004B	ORS-TM06-07	Streptococcus_anginosus	99.59	Bacillota	Streptococcus	Streptococcus anginosus_C	104
IF3KA13005A	ORS-TM06-107	Actinomyces_odontolyticus	98.37	Actinomycetota	Pauljensenia	Pauljensenia sp000466265	105
IF3KA13005B	ORS-TM06-08	Rothia_mucilaginosa	99.37	Actinomycetota	Rothia	Rothia sp015265375	91
IF3KA13007A	ORS-TM06-109	Granulicatella_elegans	100.00	Bacillota	Granulicatella	0	57
IF3KA13008A	ORS-TM06-110	Gemella_haemolysans	99.72	Bacillota	Gemella	0	84
IF3KA13016A	ORS-TM06-231	Streptococcus_oralis	99.22	Bacillota	Streptococcus	Streptococcus sp004166885	98
IF3KA14004AB	ORS-TM07-51	Streptococcus_salivarius	100.00	Bacillota	Streptococcus	Streptococcus salivarius	4
IF3KA14005A	ORS-TM07-18	Streptococcus_infantis	99.45	Bacillota	Streptococcus	Streptococcus timonensis	16
IF3KA14005AB	ORS-TM07-52	Streptococcus_rubneri	99.45	Bacillota	Streptococcus	Streptococcus xiaochunlingii	5
IF3KA14005B	ORS-TM07-240	Streptococcus_oralis	99.84	Bacillota	Streptococcus	Streptococcus mitis_AZ	11
IF3KA14006A	ORS-TM07-19	Streptococcus_sanguinis	99.79	Bacillota	Streptococcus	Streptococcus sanguinis_H	3
IF3KA14006B	ORS-TM07-241	Streptococcus_sinensis	99.66	Bacillota	Streptococcus	Streptococcus sinensis	24
IF3KA14008A	ORS-TM07-21	Streptococcus_rubneri	99.86	Bacillota	Streptococcus	Streptococcus rubneri	14
IF3KA15001	ORS-AM08-15	Streptococcus_salivarius	99.93	Bacillota	Streptococcus	Streptococcus salivarius	4
IF3KA15001A	ORS-AM08-32	Streptococcus_anginosus	99.66	Bacillota	Streptococcus	Streptococcus anginosus	61
IF3KA15001AB	ORS-AM08-59	Streptococcus_anginosus	99.66	Bacillota	Streptococcus	Streptococcus anginosus	61
IF3KA15002A	ORS-AM08-33	Atopobium_parvulum	99.65	Actinomycetota	Lancefieldella	Lancefieldella sp000564995	26
IF3KA15002AB	ORS-AM08-60	Streptococcus_oralis	98.77	Bacillota	Streptococcus	Streptococcus xiaochunlingii	5
IF3KA15003A	ORS-AM08-34	Granulicatella_adiacens	99.72	Bacillota	Granulicatella	Granulicatella sp001058355	106
IF3KA15006A	ORS-AM08-153	Actinomyces_odontolyticus	98.59	Actinomycetota	Pauljensenia	Pauljensenia sp902373545	45
IF3KA15007A	ORS-AM08-154	Actinomyces_odontolyticus	99.29	Actinomycetota	Pauljensenia	Pauljensenia sp018382595	97
IF3KA15007B	ORS-AM08-119	Streptococcus_salivarius	100.00	Bacillota	Streptococcus	Streptococcus salivarius	4
IF3KC09001	ODP-AF04-87	Streptococcus_salivarius	100.00	Bacillota	Streptococcus	Streptococcus salivarius	4
IF3KC09001A	ODP-AF04-161	Granulicatella_adiacens	93.51	Bacillota	Granulicatella	Granulicatella adiacens	23
IF3KC09002	ODP-AF04-88	Streptococcus_mitis	99.45	Bacillota	Streptococcus	0	9
IF3KC10005A	ODP-AF05-77	Streptococcus_salivarius	99.82	Bacillota	Streptococcus	Streptococcus salivarius	4
IF3KC12001AB	ODP-AM05-264	Streptococcus_mitis	99.79	Bacillota	Streptococcus	Streptococcus mitis_AZ	11
IF3KC12001B	ODP-AM05-72	Streptococcus_salivarius	100.00	Bacillota	Streptococcus	Streptococcus salivarius	4
IF3KC12002	ODP-AM05-34	Streptococcus_anginosus	99.52	Bacillota	Streptococcus	Streptococcus anginosus	61
IF3KC12002AB	ODP-AM05-265	Streptococcus_anginosus	99.52	Bacillota	Streptococcus	Streptococcus anginosus	61
IF3KC12002B	ODP-AM05-73	Streptococcus_gordonii	99.93	Bacillota	Streptococcus	Streptococcus gordonii	63
IF3KC12003AB	ODP-AM05-266	Streptococcus_constellatus	99.79	Bacillota	Streptococcus	Streptococcus constellatus	46

IF3KC12004	ODP-AM05-444	Rothia_aeria	98.68	Actinomycetota	Rothia	Rothia aeria	32
IF3KC12004A	ODP-AM05-243	Streptococcus_sanguinis	99.65	Bacillota	Streptococcus	Streptococcus sanguinis_A	29
IF3KC12004AB	ODP-AM05-267	Streptococcus_mitis	99.79	Bacillota	Streptococcus	Streptococcus mitis_AZ	11
IF3KC12006A	ODP-AM05-503	Streptococcus_gordonii	100.00	Bacillota	Streptococcus	Streptococcus gordonii	63
IF3KC12006AB	ODP-AM05-269	Actinomyces_odontolyticus	99.29	Actinomycetota	Pauljensenia	Pauljensenia odontolytica_A	27
IF3KC12007A	ODP-AM05-533	Actinomyces_naeslundii	99.52	Actinomycetota	Actinomyces	Actinomyces naeslundii	33
IF3KC13001A	ODP-TM06-159	Streptococcus_oralis	99.45	Bacillota	Streptococcus	0	102
IF3KC13003	ODP-TM06-50	Streptococcus_gordonii	99.92	Bacillota	Streptococcus	Streptococcus gordonii	63
IF3KC13006A	ODP-TM06-164	Streptococcus_sanguinis	100.00	Bacillota	Streptococcus	Streptococcus sanguinis_H	3
IF3KC14001	ODP-TM07-201	Microbacterium_oxydans	99.79	Actinomycetota	Microbacterium	Microbacterium algeriense	12
IF3KC14001A	ODP-TM07-131	Granulicatella_adiacens	98.64	Bacillota	Granulicatella	0	34
IF3KC14001AB	ODP-TM07-158	Streptococcus_cristatus	99.72	Bacillota	Streptococcus	Streptococcus cristatus	36
IF3KC14002AB	ODP-TM07-159	Streptococcus_rubneri	99.51	Bacillota	Streptococcus	Streptococcus xiaochunlingii	5
IF3KC14004A	ODP-TM07-134	Veillonella_parvula	99.32	Bacillota	Veillonella	Veillonella parvula_A	92
IF3KC14005	ODP-TM07-296	Microbacterium_oxydans	99.79	Actinomycetota	Microbacterium	Microbacterium algeriense	12
IF3KC14005A	ODP-TM07-173	Microbacterium_oxydans	99.79	Actinomycetota	Microbacterium	Microbacterium algeriense	12
IF3KC14005AB	ODP-TM07-162	Streptococcus_mitis	99.45	Bacillota	Streptococcus	Streptococcus oralis	107
IF3KC14006A	ODP-TM07-271	Streptococcus_cristatus	99.72	Bacillota	Streptococcus	Streptococcus cristatus	36
IF3KC14007A	ODP-TM07-272	Streptococcus_mitis	99.45	Bacillota	Streptococcus	Streptococcus oralis	107
IF3KC15002A	ODP-AM08-308	Streptococcus_gordonii	99.66	Bacillota	Streptococcus	Streptococcus gordonii	63
IF3KC15003A	ODP-AM08-396	Abiotrophia_defectiva	99.66	Bacillota	Abiotrophia	Abiotrophia defectiva	37
IF3KC15005	ODP-AM08-466	Rothia_mucilaginosa	99.37	Actinomycetota	Rothia	0	109
IF3KC15005A	ODP-AM08-398	Streptococcus_oralis	99.72	Bacillota	Streptococcus	0	108
IF3KC16001B	ODP-AF06-62	Streptococcus_rubneri	98.76	Bacillota	Streptococcus	Streptococcus sp004166885	98
IF3KC16002	ODP-AF06-103	Granulicatella_adiacens	99.66	Bacillota	Granulicatella	Granulicatella adiacens	23
IF3KT08003	ORT-AF03-214	Streptococcus_infantis	99.32	Bacillota	Streptococcus	Streptococcus infantis	73
IF3KT08006BA	ORT-AF03-21	Streptococcus_salivarius	99.93	Bacillota	Streptococcus	Streptococcus salivarius	4
IF3KT09001	ORT-AF04-16	Streptococcus_infantis	98.91	Bacillota	Streptococcus	0	110
IF3KT09001A	ORT-AF04-172	Atopobium_parvulum	99.65	Actinomycetota	Lancefieldella	0	2
IF3KT09001BA	ORT-AF04-123	Actinomyces_odontolyticus	99.15	Actinomycetota	Pauljensenia	Pauljensenia sp001064145	42
IF3KT09002	ORT-AF04-193	Streptococcus_salivarius	99.86	Bacillota	Streptococcus	Streptococcus salivarius	4
IF3KT09003A	ORT-AF04-219	Streptococcus_parasanguinis	99.18	Bacillota	Streptococcus	Streptococcus sp900766505	6
IF3KT09003BA	ORT-AF04-125	Lactococcus_lactis /Actinomyces_odontolyticus	100/95.7	Actinomycetota	Pauljensenia	Pauljensenia sp001064145	42
IF3KT10001A	ORT-AF05-117	Enterococcus_faecalis	100.00	Bacillota	Enterococcus	Enterococcus faecalis	21
IF3KT10005A	ORT-AF05-121	Granulicatella_adiacens	98.43	Bacillota	Granulicatella	0	95
IF3KT11001A	ORT-AM04-57	Streptococcus_salivarius	100.00	Bacillota	Streptococcus	Streptococcus salivarius	4
IF3KT11003A	ORT-AM04-59	Streptococcus_oralis	99.11	Bacillota	Streptococcus	Streptococcus sp015256435	111
IF3KT12001	ORT-AM05-07	Streptococcus_salivarius	100.00	Bacillota	Streptococcus	Streptococcus salivarius	4
IF3KT12002AB	ORT-AM05-499	Streptococcus_rubneri	99.51	Bacillota	Streptococcus	Streptococcus rubneri	14
IF3KT12003A	ORT-AM05-281	Streptococcus_timonensis	99.79	Bacillota	Streptococcus	Streptococcus mitis_BG	16
IF3KT12003ABC	ORT-AM05-525	Streptococcus_mitis	99.79	Bacillota	Streptococcus	Streptococcus mitis_AZ	11
IF3KT12004A	ORT-AM05-282	Streptococcus_oralis	98.64	Bacillota	Streptococcus	Streptococcus sp002355895	112
IF3KT12004ABC	ORT-AM05-527	Streptococcus_mitis	99.79	Bacillota	Streptococcus	Streptococcus mitis_AZ	11
IF3KT12009A	ORT-AM05-511	Streptococcus_constellatus	99.12	Bacillota	Streptococcus	Streptococcus constellatus	46
IF3KT12009AC	ORT-AM05-512	Streptococcus_constellatus	99.12	Bacillota	Streptococcus	Streptococcus constellatus	46
IF3KT13002A	ORT-TM06-179	Streptococcus_salivarius	99.93	Bacillota	Streptococcus	Streptococcus salivarius	4
IF3KT13004A	ORT-TM06-181	Streptococcus_gordonii	99.66	Bacillota	Streptococcus	Streptococcus gordonii	63
IF3KT13004B	ORT-TM06-80	Rothia_mucilaginosa	99.37	Actinomycetota	Rothia	Rothia mucilaginosa_B	59
IF3KT13012A	ORT-TM06-189	Streptococcus_sanguinis	100.00	Bacillota	Streptococcus	Streptococcus sanguinis_C	113
IF3KT14001	ORT-TM07-187	Microbacterium_oxydans	99.79	Actinomycetota	Microbacterium	Microbacterium algeriense	12
IF3KT14001A	ORT-TM07-84	Streptococcus_salivarius	100.00	Bacillota	Streptococcus	Streptococcus salivarius	4
IF3KT14001AB	ORT-TM07-100	Streptococcus_salivarius	100.00	Bacillota	Streptococcus	Streptococcus salivarius	4
IF3KT14002AB	ORT-TM07-101	Actinomyces_odontolyticus /Gemella_sanguinis	98.79/99.93	Actinomycetota	Pauljensenia	Pauljensenia sp018382595	97
IF3KT14003AB	ORT-TM07-102	Actinomyces_odontolyticus	98.93	Actinomycetota	Pauljensenia	Pauljensenia sp018382595	97
IF3KT14004A	ORT-TM07-87	Streptococcus_parasanguinis	98.84	Bacillota	Streptococcus	0	22
IF3KT14006A	ORT-TM07-89	Streptococcus_timonensis	99.86	Bacillota	Streptococcus	Streptococcus timonensis	16
IF3KT14007A	ORT-TM07-90	Atopobium_parvulum	99.72	Actinomycetota	Lancefieldella	Lancefieldella sp000564995	114
IF3KT14008A	ORT-TM07-91	Streptococcus_parasanguinis	98.84	Bacillota	Streptococcus	0	22
IF3KT14008AB	ORT-TM07-107	Streptococcus_rubneri	99.51	Bacillota	Streptococcus	Streptococcus xiaochunlingii	5
IF3KT14009AB	ORT-TM07-108	Streptococcus_parasanguinis	98.84	Bacillota	Streptococcus	0	22
IF3KT15001	ORT-AM08-202	Streptococcus_salivarius	99.93	Bacillota	Streptococcus	Streptococcus salivarius	4
IF3KT15002B	ORT-AM08-226	Rothia_dentocariosa	100.00	Actinomycetota	Rothia	Rothia dentocariosa	13
IF3KT15004	ODP-AM08-337	Gemella_sanguinis	99.86	Bacillota	Gemella	Gemella sanguinis	17
IF3KT15004A	ORT-AM08-425	Streptococcus_anginosus	99.59	Bacillota	Streptococcus	Streptococcus anginosus	61
IF3KT15006	ORT-AM08-480	Rothia_mucilaginosa	99.58	Actinomycetota	Rothia	Rothia mucilaginosa	78
IF3KT15007A	ORT-AM08-428	Actinomyces_odontolyticus	98.93	Actinomycetota	Pauljensenia	Pauljensenia sp018382595	97
IF3KT15008AB	ORT-AM08-458	Granulicatella_adiacens	99.72	Bacillota	Granulicatella	0	25
IF3KT15013A	ORT-AM08-434	Streptococcus_australis	99.38	Bacillota	Streptococcus	0	60
IF3KT16001B	ORT-AF06-28	Microbacterium_oxydans	99.79	Actinomycetota	Microbacterium	Microbacterium algeriense	12
IF3KT16003B	ORT-AF06-30	Streptococcus_rubneri	99.38	Bacillota	Streptococcus	Streptococcus xiaochunlingii	5
IF3KT16004	ORT-AF06-10	Microbacterium_oxydans	99.79	Actinomycetota	Microbacterium	Microbacterium algeriense	12
IF3KT16005	ORT-AF06-36	Streptococcus_infantis	99.45	Bacillota	Streptococcus	Streptococcus infantis_H	73
JF3KA10001A	ORS-AF05-30	Lactobacillus_paracasei	99.90	Bacillota	Lacticaseibacillus	Lacticaseibacillus paracasei	43
JF3KA12003	ORS-AM05-138	Streptococcus_mitis	99.76	Bacillota	Streptococcus	Streptococcus mitis_AZ	11
JF3KA12004B	ORS-AM05-334	Neisseria_elongata	99.79	Pseudomonadota	Neisseria	Neisseria elongata	64
JF3KA12006AB	ORS-AM05-362	Fusobacterium_periodonticum	99.37	Fusobacteriota	Fusobacterium	Fusobacterium pseudoperiodonticum	80
JF3KA12006B	ORS-AM05-474	Rothia_mucilaginosa	99.51	Actinomycetota	Rothia	Rothia mucilaginosa_B	59
JF3KA12007AB	ORS-AM05-363	Streptococcus_sinensis	99.45	Bacillota	Streptococcus	Streptococcus sinensis	24
JF3KA13004	ORS-TM06-31	Rothia_dentocariosa	100.00	Actinomycetota	Rothia	Rothia dentocariosa	13
JF3KA14001AB	ORS-TM07-54	Enterococcus_faecalis	100.00	Bacillota	Enterococcus	Enterococcus faecalis	21
JF3KA15002AB	ORS-AM08-63	Streptococcus_mitis	99.38	Bacillota	Streptococcus	0	115
JF3KC08001A	ODP-AF03-102	Streptococcus_mitis	97.88	Bacillota	Streptococcus	Streptococcus oralis	107
JF3KC09001	ODP-AF04-91	Streptococcus_salivarius	100.00	Bacillota	Streptococcus	Streptococcus salivarius	4
JF3KC09004	ODP-AF04-94	Streptococcus_mitis	99.45	Bacillota	Streptococcus	0	9
JF3KC11001	ODP-AM04-107	Streptococcus_salivarius	100.00	Bacillota	Streptococcus	Streptococcus salivarius	4

JF3KC12001A	ODP-AM05-244	Streptococcus_anginosus	99.52	Bacillota	Streptococcus	Streptococcus anginosus	61
JF3KC12002AB	ODP-AM05-271	Granulicatella_adiacens	99.66	Bacillota	Granulicatella	0	34
JF3KC12002B	ODP-AM05-303	Neisseria_macacae	99.52	Pseudomonadota	Neisseria	Neisseria sicca_A	44
JF3KC12003AB	ODP-AM05-272	Granulicatella_adiacens	99.66	Bacillota	Granulicatella	Granulicatella sp905371865	30
JF3KC12004AB	ODP-AM05-391	Lactococcus_lactis /Actinomyces_oris	100/99.76	Actinomycetota	Actinomyces	Actinomyces oris_A	58
JF3KC12005A	ODP-AM05-504	Streptococcus_mitis	99.59	Bacillota	Streptococcus	0	116
JF3KC14002B	ODP-TM07-321	Microbacterium_oxydans	99.79	Actinomycetota	Microbacterium	Microbacterium algeriense	12
JF3KC16002	ODP-AF06-99	Streptococcus_mitis	99.79	Bacillota	Streptococcus	Streptococcus mitis_AK	101
JF3KC16004	ODP-AF06-105	Microbacterium_oxydans	99.79	Actinomycetota	Microbacterium	Microbacterium algeriense	12
JF3KT08002A	ORT-AF03-133	Fusobacterium_periodonticum	99.78	Fusobacteriota	Fusobacterium	Fusobacterium pseudoperiodonticum	80
JF3KT08002BA	ORT-AF03-24	Streptococcus_salivarius	100.00	Bacillota	Streptococcus	Streptococcus sp001556435	117
JF3KT09001A	ORT-AF04-174	Actinomyces_odontolyticus	99.69	Actinomycetota	Pauljensenia	Pauljensenia sp001064145	42
JF3KT09003	ORT-AF04-19	Streptococcus_salivarius	99.93	Bacillota	Streptococcus	Streptococcus salivarius	4
JF3KT09003A	ORT-AF04-225	Actinomyces_odontolyticus	80.15	Actinomycetota	Pauljensenia	Pauljensenia sp001064145	42
JF3KT10001	ORT-AF05-174	Rothia_mucilaginosa	99.51	Actinomycetota	Rothia	Rothia mucilaginosa_A	41
JF3KT10001A	ORT-AF05-123	Enterococcus_faecalis	100.00	Bacillota	Enterococcus	Enterococcus faecalis	21
JF3KT12003B	ORT-AM05-319	Neisseria_elongata	99.79	Pseudomonadota	Neisseria	Neisseria elongata	64
JF3KT12005AB	ORT-AM05-529	Streptococcus_mitis	99.38	Bacillota	Streptococcus	0	118
JF3KT12007A	ORT-AM05-514	Streptococcus_salivarius	100.00	Bacillota	Streptococcus	Streptococcus salivarius	4
JF3KT14002	ORT-TM07-192	Rothia_mucilaginosa	99.37	Actinomycetota	Rothia	Rothia mucilaginosa_B	59
JF3KT15001	ORT-AM08-205	Rothia_mucilaginosa	99.58	Actinomycetota	Rothia	Rothia mucilaginosa	78
JF3KT15001A	ORT-AM08-438	Streptococcus_salivarius	100.00	Bacillota	Streptococcus	Streptococcus salivarius	4
JF3KT15002	ORT-AM08-206	Streptococcus_oralis	97.14	Bacillota	Streptococcus	Streptococcus pseudopneumoniae_A	119
JF3KT15002B	ODP-AM08-363	Rothia_mucilaginosa	99.51	Actinomycetota	Rothia	Rothia mucilaginosa	78
MF3KA09001A	ORS-AF04-142	Streptococcus_oralis	99.11	Bacillota	Streptococcus	Streptococcus sp015256435	111
MF3KA09002A	ORS-AF04-143	Streptococcus_oralis	99.32	Bacillota	Streptococcus	0	120
MF3KA09005A	ORS-AF04-236	Streptococcus_sanguinis	99.93	Bacillota	Streptococcus	Streptococcus sanguinis	3
MF3KA11001	ORS-AM04-111	Granulicatella_adiacens	99.66	Bacillota	Granulicatella	0	25
MF3KA11001A	ORS-AM04-88	Actinomyces_odontolyticus	99.69	Actinomycetota	Pauljensenia	Pauljensenia sp001064145	42
MF3KA11003A	ORS-AM04-90	Atopobium_parvulum	99.72	Actinomycetota	Lancefieldella	0	2
MF3KA12001B	ORS-AM05-336	Staphylococcus_hominis	99.72	Bacillota	Staphylococcus	Staphylococcus hominis	99
MF3KA12002	ORS-AM05-140	#N/A	#N/A	Bacillota	Streptococcus	Streptococcus koreensis	48
MF3KA12002AB	ORS-AM05-205	Actinomyces_odontolyticus	99.01	Actinomycetota	Pauljensenia	Pauljensenia sp000278725	121
MF3KA12002B	ORS-AM05-475	Corynebacterium_argentoratense	99.79	Actinomycetota	Corynebacterium	Corynebacterium argentoratense	54
MF3KA12003AB	ORS-AM05-364	Solobacterium_moorei	98.27	Bacillota	Bulleidia	0	47
MF3KA12004AB	ORS-AM05-365	Streptococcus_timonensis	99.86	Bacillota	Streptococcus	Streptococcus mitis_BG	16
MF3KA12007AB	ORS-AM05-494	Streptococcus_salivarius	99.86	Bacillota	Streptococcus	Streptococcus salivarius	4
MF3KA12008AB	ORS-AM05-495	Streptococcus_constellatus	99.79	Bacillota	Streptococcus	Streptococcus constellatus	46
MF3KA13001A	ORS-TM06-111	Streptococcus_anginosus	98.24	Bacillota	Streptococcus	Streptococcus anginosus	61
MF3KA13001AB	ORS-TM06-118	Streptococcus_anginosus	98.24	Bacillota	Streptococcus	Streptococcus anginosus	61
MF3KA13002A	ORS-TM06-112	Streptococcus_parasanguinis	99.45	Bacillota	Streptococcus	Streptococcus parasanguinis	20
MF3KA13003A	ORS-TM06-113	Streptococcus_mitis	99.25	Bacillota	Streptococcus	0	122
MF3KA13004A	ORS-TM06-114	Gemella_haemolysans	99.72	Bacillota	Gemella	0	84
MF3KA13014A	ORS-TM06-235	Veillonella_tobetsuensis	99.89	Bacillota	Veillonella	Veillonella parvula_A	92
MF3KA14001	ORS-TM07-224	Streptococcus_salivarius	100.00	Bacillota	Streptococcus	Streptococcus salivarius	4
MF3KA14002	ORS-TM07-225	Streptococcus_mitis	99.65	Bacillota	Streptococcus	0	85
MF3KA14006B	ORS-TM07-255	Granulicatella_elegans	99.52	Bacillota	Granulicatella	0	123
MF3KA15001AB	ORS-AM08-69	Granulicatella_adiacens	99.72	Bacillota	Granulicatella	Granulicatella sp001058355	106
MF3KA15005B	ORS-AM08-136	Rothia_mucilaginosa	99.44	Actinomycetota	Rothia	Rothia mucilaginosa	78
MF3KC09001	ODP-AF04-99	Streptococcus_salivarius	99.86	Bacillota	Streptococcus	Streptococcus salivarius	4
MF3KC09001B	ODP-AF04-114	Lactobacillus_paracasei	99.90	Bacillota	Lacticaseibacillus	Lacticaseibacillus paracasei	43
MF3KC09002A	ODP-AF04-250	Streptococcus_intermedius	100.00	Bacillota	Streptococcus	Streptococcus intermedius	70
MF3KC11001A	ODP-AM04-44	Streptococcus_salivarius	100.00	Bacillota	Streptococcus	Streptococcus salivarius	4
MF3KC11002A	ODP-AM04-45	Streptococcus_cristatus	99.65	Bacillota	Streptococcus	0	124
MF3KC12001A	ODP-AM05-247	Streptococcus_sanguinis	99.65	Bacillota	Streptococcus	Streptococcus sanguinis_A	29
MF3KC12001AB	ODP-AM05-273	Streptococcus_anginosus	99.52	Bacillota	Streptococcus	Streptococcus anginosus	61
MF3KC12001B	ODP-AM05-76	Rothia_dentocariosa	99.93	Actinomycetota	Rothia	Rothia dentocariosa	13
MF3KC12002A	ODP-AM05-248	Streptococcus_anginosus	99.52	Bacillota	Streptococcus	Streptococcus anginosus	61
MF3KC12002AB	ODP-AM05-535	Streptococcus_mitis	99.79	Bacillota	Streptococcus	Streptococcus mitis_AZ	11
MF3KC12003	ODP-AM05-41	Streptococcus_anginosus	99.52	Bacillota	Streptococcus	Streptococcus anginosus	61
MF3KC12003A	ODP-AM05-249	Granulicatella_adiacens	99.66	Bacillota	Granulicatella	Granulicatella adiacens	23
MF3KC12003B	ODP-AM05-78	Streptococcus_gordonii	99.93	Bacillota	Streptococcus	Streptococcus gordonii	63
MF3KC12004A	ODP-AM05-429	Streptococcus_salivarius	100.00	Bacillota	Streptococcus	Streptococcus salivarius	4
MF3KC12006B	ODP-AM05-440	Corynebacterium_accolens	99.72	Actinomycetota	Corynebacterium	Corynebacterium accolens	125
MF3KC13003	ODP-TM06-55	Streptococcus_gordonii	99.66	Bacillota	Streptococcus	Streptococcus gordonii	63
MF3KC15001A	ODP-AM08-309	Streptococcus_cristatus	99.72	Bacillota	Streptococcus	Streptococcus cristatus	36
MF3KC15002A	ODP-AM08-310	Granulicatella_adiacens	99.72	Bacillota	Granulicatella	Granulicatella adiacens	23
MF3KC15010AB	ODP-AM08-335	Granulicatella_adiacens	99.72	Bacillota	Granulicatella	Granulicatella adiacens	23
MF3KT08001	ORT-AF03-215	Streptococcus_oralis	98.91	Bacillota	Streptococcus	0	126
MF3KT08001A	ORT-AF03-01	Streptococcus_parasanguinis	99.11	Bacillota	Streptococcus	Streptococcus parasanguinis_D	71
MF3KT08004A	ORT-AF03-04	Streptococcus_intermedius	100.00	Bacillota	Streptococcus	Streptococcus intermedius	70
MF3KT08008A	ORT-AF03-08	Streptococcus_parasanguinis	99.93	Bacillota	Streptococcus	Streptococcus parasanguinis	20
MF3KT09001	ORT-AF04-23	Streptococcus_salivarius	99.86	Bacillota	Streptococcus	Streptococcus salivarius	4
MF3KT09001A	ORT-AF04-222	Atopobium_parvulum	99.72	Actinomycetota	Lancefieldella	0	2
MF3KT09003A	ORT-AF04-247	Streptococcus_infantis	99.59	Bacillota	Streptococcus	0	127
MF3KT10001A	ORT-AF05-128	Enterococcus_faecalis	100.00	Bacillota	Enterococcus	Enterococcus faecalis	21
MF3KT10005A	ORT-AF05-132	Atopobium_rimae	99.86	Actinomycetota	Lancefieldella	Lancefieldella rimae	128
MF3KT10008A	ORT-AF05-232	Lactococcus_lactis /Actinomyces_odontolyticus	100/98.86	Actinomycetota	Pauljensenia	0	129
MF3KT11002A	ORT-AM04-65	Streptococcus_salivarius	99.86	Bacillota	Streptococcus	Streptococcus salivarius	4
MF3KT12001A	ORT-AM05-287	Streptococcus_mitis	99.79	Bacillota	Streptococcus	Streptococcus mitis_AZ	11
MF3KT12001AB	ORT-AM05-101	Granulicatella_adiacens	99.66	Bacillota	Granulicatella	0	25
MF3KT12002A	ORT-AM05-515	Streptococcus_salivarius	100.00	Bacillota	Streptococcus	Streptococcus salivarius	4
MF3KT12002AB	ORT-AM05-102	Streptococcus_mitis	99.79	Bacillota	Streptococcus	Streptococcus mitis_AZ	11
MF3KT12003A	ORT-AM05-521	Streptococcus_mitis	99.79	Bacillota	Streptococcus	Streptococcus mitis_AZ	11
MF3KT12004AB	ORT-AM05-104	Streptococcus_mitis	99.79	Bacillota	Streptococcus	Streptococcus mitis_AZ	11

MF3KT12004B	ORT-AM05-63	Streptococcus_salivarius	100.00	Bacillota	Streptococcus	Streptococcus salivarius	4
MF3KT12008B	ORT-AM05-310	Rothia_mucilaginosa	99.51	Actinomycetota	Rothia	Rothia mucilaginosa_B	59
MF3KT13001A	ORT-TM06-193	Streptococcus_mitis	97.34	Bacillota	Streptococcus	0	130
MF3KT13002A	ORT-TM06-194	Streptococcus_rubneri	98.76	Bacillota	Streptococcus	Streptococcus xiaochunlingii	5
MF3KT13003A	ORT-TM06-195	Streptococcus_gordonii	99.66	Bacillota	Streptococcus	Streptococcus gordonii	63
MF3KT13008A	ORT-TM06-200	Gemella_sanguinis	99.86	Bacillota	Gemella	Gemella sanguinis	17
MF3KT13011A	ORT-TM06-203	Streptococcus_parasanguinis	99.79	Bacillota	Streptococcus	Streptococcus parasanguinis	20
MF3KT14001B	ORT-TM07-207	Streptococcus_salivarius	100.00	Bacillota	Streptococcus	Streptococcus salivarius	4
MF3KT15001A	ORT-AM08-450	Streptococcus_anginosus	99.66	Bacillota	Streptococcus	Streptococcus anginosus	61
MF3KT15001AB	ORT-AM08-463	Streptococcus_anginosus	99.66	Bacillota	Streptococcus	Streptococcus anginosus	61
MF3KT15001B	ORT-AM08-229	Lactococcus_garvieae	100.00	Bacillota	Lactococcus	Lactococcus garvieae	51
MF3KT15004	ORT-AM08-210	Lactococcus_garvieae	100.00	Bacillota	Lactococcus	Lactococcus garvieae	51
MF3KT16001	ORT-AF06-11	Microbacterium_oxydans	99.79	Actinomycetota	Microbacterium	Microbacterium algeriense	12
MF3KT16003B	ORT-AF06-97	Rothia_mucilaginosa	99.58	Actinomycetota	Rothia	Rothia mucilaginosa_B	59
ODP-AM01-02BB	ODP-AM01-02BB	Streptococcus_mitis	99.65	Bacillota	Streptococcus	Streptococcus oralis	107
ODP-AM01-02O	ODP-AM01-02O	Neisseria_cinerea	98.35	Pseudomonadota	Neisseria	Neisseria subflava	15
ODP-AM01-04	ODP-AM01-04	Streptococcus_sinensis	99.72	Bacillota	Streptococcus	Streptococcus sinensis	24
ODP-AM01-04MBO	ODP-AM01-04MBO	Streptococcus_salivarius	99.93	Bacillota	Streptococcus	Streptococcus vestibularis	131
ODP-AM01-05	ODP-AM01-05	Gemella_morbillorum	98.98	Bacillota	Gemella	Gemella morbillorum	65
ODP-AM01-05BB	ODP-AM01-05BB	Actinomyces_hongkongensis	98.30	Actinomycetota	Pauljensenia	Pauljensenia hongkongensis	132
ODP-AM01-06	ODP-AM01-06	Fusobacterium_nucleatum	99.43	Fusobacteriota	Fusobacterium	0	133
ODP-AM01-08	ODP-AM01-08	Streptococcus_mitis	99.65	Bacillota	Streptococcus	Streptococcus oralis	107
ODP-AM01-10	ODP-AM01-10	Catonella_morbi	92.54	Bacillota	Catonella	0	134
ORS-AM01-01	ORS-AM01-01	Streptococcus_salivarius	99.93	Bacillota	Streptococcus	Streptococcus vestibularis	131
ORS-AM01-01BB	ORS-AM01-01BB	Actinomyces_naeslundii	97.23	Actinomycetota	Actinomyces	Actinomyces oris	39
ORS-AM01-01O	ORS-AM01-01O	Neisseria_subflava	99.77	Pseudomonadota	Neisseria	Neisseria subflava	15
ORS-AM01-02	ORS-AM01-02	Fusobacterium_periodonticum	99.30	Fusobacteriota	Fusobacterium	Fusobacterium pseudoperiodonticum	80
ORS-AM01-02BB	ORS-AM01-02BB	Streptococcus_infantis	99.25	Bacillota	Streptococcus	0	135
ORS-AM01-02BBO	ORS-AM01-02BBO	Actinomyces_naeslundii	97.23	Actinomycetota	Actinomyces	Actinomyces oris	39
ORS-AM01-02MB	ORS-AM01-02MB	Streptococcus_sanguinis	99.72	Bacillota	Streptococcus	Streptococcus sanguinis_A	29
ORS-AM01-03BB	ORS-AM01-03BB	Streptococcus_rubneri	99.45	Bacillota	Streptococcus	Streptococcus rubneri	14
ORS-AM01-04BBO	ORS-AM01-04BBO	Morococcus_cerebrosus	99.93	Pseudomonadota	Neisseria	Neisseria cerebrosa	136
ORS-AM01-04MB	ORS-AM01-04MB	Streptococcus_constellatus	99.93	Bacillota	Streptococcus	Streptococcus constellatus	46
ORS-AM01-13TBO	ORS-AM01-13TBO	Neisseria_flavescens	98.15	Pseudomonadota	Neisseria	Neisseria flavescens_B	15
ORT-AM01-01TBO	ORT-AM01-01TBO	Bacillus_cereus	100.00	Bacillota	Bacillus_A	Bacillus_A luti	137
ORT-AM01-02	ORT-AM01-02	Eubacterium_yurii	92.98	Bacillota	Peptoanaerobacter	0	138
ORT-AM01-03O	ORT-AM01-03O	Rothia_mucilaginosa	99.37	Actinomycetota	Rothia	Rothia mucilaginosa_B	59
ORT-AM01-05MB	ORT-AM01-05MB	Streptococcus_constellatus	99.93	Bacillota	Streptococcus	Streptococcus constellatus	46
ORT-AM01-06O	ORT-AM01-06O	Granulicatella_adiacens	99.66	Bacillota	Granulicatella	0	25
ORT-AM01-08MB	ORT-AM01-08MB	Streptococcus_cristatus	99.65	Bacillota	Streptococcus	Streptococcus sp902460355	139
ORT-AM01-12O	ORT-AM01-12O	Bacillus_aryabhattai	100.00	Bacillota	Priestia	Priestia megaterium_A	140
PF3KA08003A	ORS-AF03-153	Streptococcus_salivarius	100.00	Bacillota	Streptococcus	Streptococcus sp001556435	117
PF3KA08005A	ORS-AF03-155	Streptococcus_pseudopneumoniae	99.65	Bacillota	Streptococcus	Streptococcus sp000187745	141
PF3KA09001A	ORS-AF04-146	Streptococcus_salivarius	99.86	Bacillota	Streptococcus	Streptococcus salivarius	4
PF3KA10004A	ORS-AF05-45	Atopobium_parvulum	99.72	Actinomycetota	Lancefieldella	0	2
PF3KA11001A	ORS-AM04-42	Actinomyces_odontolyticus	98.37	Actinomycetota	Pauljensenia	Pauljensenia odontolytica_A	27
PF3KA12001	ORS-AM05-141	Streptococcus_mitis	99.72	Bacillota	Streptococcus	Streptococcus mitis_AZ	11
PF3KA12002AB	ORS-AM05-207	Fusobacterium_hwasookii	100.00	Fusobacteriota	Fusobacterium	Fusobacterium hwasookii	142
PF3KA12003AB	ORS-AM05-208	Fusobacterium_periodonticum	99.71	Fusobacteriota	Fusobacterium	Fusobacterium pseudoperiodonticum	80
PF3KA12005B	ORS-AM05-477	Rothia_mucilaginosa	99.51	Actinomycetota	Rothia	Rothia mucilaginosa_B	59
PF3KA12007	ORS-AM05-147	Rothia_dentocariosa	99.93	Actinomycetota	Rothia	Rothia dentocariosa	13
PF3KA13002	ORS-TM06-36	Streptococcus_mitis	95.23	Bacillota	Streptococcus	Streptococcus oralis_B	31
PF3KA13004B	ORS-TM06-246	Rothia_dentocariosa	99.93	Actinomycetota	Rothia	Rothia dentocariosa	13
PF3KA14002	ORS-TM07-227	Streptococcus_mitis	98.97	Bacillota	Streptococcus	0	143
PF3KA14002AB	ORS-TM07-61	Streptococcus_salivarius	100.00	Bacillota	Streptococcus	Streptococcus salivarius	4
PF3KA14003B	ORS-TM07-262	Microbacterium_oxydans	99.79	Actinomycetota	Microbacterium	Microbacterium algeriense	12
PF3KA14005AB	ORS-TM07-64	Actinomyces_odontolyticus	98.93	Actinomycetota	Pauljensenia	Pauljensenia sp018382595	97
PF3KA14007AB	ORS-TM07-66	Actinomyces_odontolyticus	99.19	Actinomycetota	Pauljensenia	Pauljensenia sp018382595	97
PF3KA14014AB	ORS-TM07-73	Enterococcus_faecalis	100.00	Bacillota	Enterococcus	Enterococcus faecalis	21
PF3KA15001A	ORS-AM08-42	Streptococcus_parasanguinis	99.72	Bacillota	Streptococcus	Streptococcus parasanguinis	20
PF3KA15002A	ORS-AM08-43	Atopobium_parvulum	99.65	Actinomycetota	Lancefieldella	Lancefieldella sp000564995	26
PF3KA15003A	ORS-AM08-44	Granulicatella_adiacens	99.72	Bacillota	Granulicatella	Granulicatella sp001058355	18
PF3KA15006AB	ORS-AM08-178	Actinomyces_odontolyticus	99.29	Actinomycetota	Pauljensenia	0	144
PF3KC08001A	ODP-AF03-106	Enterococcus_faecalis	100.00	Bacillota	Enterococcus	Enterococcus faecalis	21
PF3KC08007BA	ODP-AF03-248	Bacillus_paralicheniformis	100.00	Bacillota	Bacillus	Bacillus paralicheniformis	145
PF3KC09001	ODP-AF04-102	Streptococcus_salivarius	99.85	Bacillota	Streptococcus	Streptococcus salivarius	4
PF3KC09001A	ODP-AF04-243	Streptococcus_sanguinis	99.86	Bacillota	Streptococcus	Streptococcus sanguinis_H	3
PF3KC09002	ODP-AF04-103	Streptococcus_salivarius	99.86	Bacillota	Streptococcus	Streptococcus salivarius	4
PF3KC09002A	ODP-AF04-244	Granulicatella_adiacens	99.93	Bacillota	Granulicatella	Granulicatella adiacens	23
PF3KC09004	ODP-AF04-105	Gemella_haemolysans	98.03	Bacillota	Gemella	Gemella haemolysans_B	49
PF3KC09005	ODP-AF04-106	Streptococcus_salivarius	99.86	Bacillota	Streptococcus	Streptococcus salivarius	4
PF3KC10001A	ODP-AF05-85	Actinomyces_odontolyticus	99.15	Actinomycetota	Pauljensenia	Pauljensenia sp001064145	42
PF3KC11001A	ODP-AM04-46	Streptococcus_salivarius	100.00	Bacillota	Streptococcus	Streptococcus salivarius	4
PF3KC12001	ODP-AM05-43	Rothia_dentocariosa	99.93	Actinomycetota	Rothia	Rothia dentocariosa	13
PF3KC12001B	ODP-AM05-80	Streptococcus_oralis	99.66	Bacillota	Streptococcus	Streptococcus oralis_B	31
PF3KC12002A	ODP-AM05-432	Fusobacterium_nucleatum	98.82	Fusobacteriota	Fusobacterium	Fusobacterium animalis	146
PF3KC12003B	ODP-AM05-115	Rothia_mucilaginosa	99.44	Actinomycetota	Rothia	Rothia mucilaginosa_B	59
PF3KC12004A	ODP-AM05-434	Fusobacterium_nucleatum	99.93	Fusobacteriota	Fusobacterium	Fusobacterium polymorphum	87
PF3KC12005A	ODP-AM05-506	Streptococcus_pseudopneumoniae	99.79	Bacillota	Streptococcus	Streptococcus symci	89
PF3KC15001B	ODP-AM08-275	Streptococcus_salivarius	100.00	Bacillota	Streptococcus	Streptococcus salivarius	4
PF3KC16003	ODP-AF06-153	Microbacterium_oxydans	99.79	Actinomycetota	Microbacterium	Microbacterium algeriense	12
PF3KT08008BA	ORT-AF03-41	Actinomyces_odontolyticus	98.86	Actinomycetota	Pauljensenia	Pauljensenia sp001838165	147
PF3KT09001	ORT-AF04-26	Staphylococcus_epidermidis /Streptococcus_salivarius	100/99.86	Bacillota	Streptococcus	Streptococcus salivarius	4
PF3KT09003A	ORT-AF04-176	Prevotella_veroralis	97.93	Bacteroidota	Prevotella	Prevotella sp000257925	62
PF3KT09004A	ORT-AF04-177	Prevotella_histicola	99.86	Bacteroidota	Prevotella	Prevotella histicola	28
PF3KT09005A	ORT-AF04-227	Streptococcus_parasanguinis	99.25	Bacillota	Streptococcus	Streptococcus sp900766505	6

PF3KT10003A	ORT-AF05-233	Atopobium_parvulum	99.72	Actinomycetota	Lancefieldella	0	96
PF3KT12001	ORT-AM05-13	Streptococcus_mitis	99.79	Bacillota	Streptococcus	Streptococcus mitis_AZ	11
PF3KT12001AB	ORT-AM05-105	Granulicatella_adiacens	99.25	Bacillota	Granulicatella	0	25
PF3KT12002AB	ORT-AM05-106	Streptococcus_salivarius	99.86	Bacillota	Streptococcus	Streptococcus salivarius	4
PF3KT12003AB	ORT-AM05-500	Streptococcus_mitis	99.79	Bacillota	Streptococcus	Streptococcus mitis_AZ	11
PF3KT12003B	ORT-AM05-112	#N/A	#N/A	Bacillota	Streptococcus	Streptococcus gordonii	63
PF3KT12005B	ORT-AM05-304	Rothia_mucilaginosa	99.51	Actinomycetota	Rothia	Rothia mucilaginosa_B	59
PF3KT12006A	ORT-AM05-451	Streptococcus_australis	99.52	Bacillota	Streptococcus	Streptococcus koreensis	48
PF3KT14001	ORT-TM07-195	Microbacterium_oxydans	99.79	Actinomycetota	Microbacterium	Microbacterium algeriense	12
PF3KT14001A	ORT-TM07-93	Streptococcus_salivarius	100.00	Bacillota	Streptococcus	Streptococcus salivarius	4
PF3KT14001AB	ORT-TM07-110	Actinomyces_odontolyticus	98.93	Actinomycetota	Pauljensenia	Pauljensenia sp018382595	97
PF3KT14001B	ORT-TM07-310	Microbacterium_oxydans	99.79	Actinomycetota	Microbacterium	Microbacterium algeriense	12
PF3KT14002A	ORT-TM07-94	Granulicatella_adiacens	99.66	Bacillota	Granulicatella	0	25
PF3KT14003AB	ORT-TM07-112	Streptococcus_salivarius	99.52	Bacillota	Streptococcus	Streptococcus salivarius	4
PF3KT15003	ORT-AM08-214	Lactococcus_garvieae	100.00	Bacillota	Lactococcus	Lactococcus garvieae	51
PF3KT16002	ORT-AF06-14	Microbacterium_oxydans	99.79	Actinomycetota	Microbacterium	Microbacterium algeriense	12
PF3KT16002B	ORT-AF06-148	Rothia_mucilaginosa	99.44	Actinomycetota	Rothia	Rothia sp001808955	78
SF3KA08003A	ORS-AF03-239	Fusobacterium_nucleatum	97.76	Fusobacteriota	Fusobacterium	Fusobacterium polymorphum	87
SF3KA09001	ORS-AF04-52	Rothia_dentocariosa	100.00	Actinomycetota	Rothia	Rothia dentocariosa	13
SF3KA09003	ORS-AF04-54	Streptococcus_mitis	99.79	Bacillota	Streptococcus	Streptococcus oralis	107
SF3KA09008	ORS-AF04-59	Streptococcus_mitis	96.73	Bacillota	Streptococcus	Streptococcus oralis	107
SF3KA12006A	ORS-AM05-380	Capnocytophaga_leadbetteri	95.12	Bacteroidota	Capnocytophaga	Capnocytophaga leadbetteri	81
SF3KA12009AB	ORS-AM05-383	Actinomyces_oris	99.76	Actinomycetota	Actinomyces	Actinomyces oris_A	58
SF3KA12011AB	ORS-AM05-471	Eubacterium_infirmum	99.86	Bacillota	Eubacterium_B	Eubacterium_B infirmum	148
SF3KA13001	ORS-TM06-37	Neisseria_cinerea	98.42	Pseudomonadota	Neisseria	Neisseria flavescens_B	15
SF3KA13001A	ORS-TM06-130	Atopobium_parvulum	99.72	Actinomycetota	Lancefieldella	0	149
SF3KA13003	ORS-TM06-39	Streptococcus_australis	98.70	Bacillota	Streptococcus	Streptococcus xiaochunlingii	56
SF3KA13005A	ORS-TM06-134	Granulicatella_adiacens	99.86	Bacillota	Granulicatella	Granulicatella adiacens	23
SF3KA13011A	ORS-TM06-140	Veillonella_dispar	99.36	Bacillota	Veillonella	Veillonella parvula_A	92
SF3KA13014A	ORS-TM06-237	Prevotella_loescheii	99.17	Bacteroidota	Prevotella	Prevotella loescheii	150
SF3KA14003AB	ORS-TM07-76	Abiotrophia_defectiva	99.39	Bacillota	Abiotrophia	Abiotrophia sp001815865	55
SF3KA14004A	ORS-TM07-35	Enterococcus_faecalis	100.00	Bacillota	Enterococcus	Enterococcus faecalis	21
SF3KA14004AB	ORS-TM07-77	Abiotrophia_defectiva	99.64	Bacillota	Abiotrophia	Abiotrophia sp001815865	55
SF3KA14006AB	ORS-TM07-79	Abiotrophia_defectiva	99.39	Bacillota	Abiotrophia	Abiotrophia sp001815865	55
SF3KA14007B	ORS-TM07-269	Streptococcus_oralis	99.76	Bacillota	Streptococcus	Streptococcus mitis_AZ	11
SF3KA14008B	ORS-TM07-270	Granulicatella_adiacens	100.00	Bacillota	Granulicatella	0	34
SF3KA15001A	ORS-AM08-46	Granulicatella_adiacens	99.72	Bacillota	Granulicatella	Granulicatella sp001058355	106
SF3KA15002AB	ORS-AM08-79	Atopobium_parvulum	99.65	Actinomycetota	Lancefieldella	Lancefieldella sp000564995	26
SF3KA15003A	ORS-AM08-48	Granulicatella_adiacens	99.72	Bacillota	Granulicatella	Granulicatella sp001058355	106
SF3KA15003AB	ORS-AM08-180	Prevotella_veroralis	98.31	Bacteroidota	Prevotella	Prevotella sp000257925	62
SF3KA15004A	ORS-AM08-49	Streptococcus_australis	99.38	Bacillota	Streptococcus	0	60
SF3KA15005A	ORS-AM08-163	Streptococcus_sanguinis	98.09	Bacillota	Streptococcus	0	40
SF3KA16005	ORS-AF06-137	Rothia_mucilaginosa	99.58	Actinomycetota	Rothia	Rothia mucilaginosa_B	59
SF3KC09001	ODP-AF04-107	Streptococcus_salivarius	100.00	Bacillota	Streptococcus	Streptococcus salivarius	4
SF3KC09001A	ODP-AF04-166	Streptococcus_sanguinis	99.79	Bacillota	Streptococcus	Streptococcus sanguinis_H	3
SF3KC09002	ODP-AF04-108	Streptococcus_salivarius	96.66	Bacillota	Streptococcus	Streptococcus salivarius	4
SF3KC10002	ODP-AF05-197	Rothia_dentocariosa	100.00	Actinomycetota	Rothia	Rothia dentocariosa	13
SF3KC10002A	ODP-AF05-91	Lactococcus_lactis /Actinomyces_odontolyticus	100/95.7	Actinomycetota	Pauljensenia	Pauljensenia sp001064145	42
SF3KC10006A	ODP-AF05-95	Atopobium_parvulum	99.72	Actinomycetota	Lancefieldella	0	2
SF3KC10008	ODP-AF05-203	Streptococcus_anginosus	99.59	Bacillota	Streptococcus	Streptococcus anginosus	61
SF3KC11001A	ODP-AM04-51	Gemella_haemolysans	99.79	Bacillota	Gemella	Gemella haemolysans_A	151
SF3KC11002	ODP-AM04-150	Rothia_mucilaginosa	99.44	Actinomycetota	Rothia	Rothia mucilaginosa_B	59
SF3KC11002A	ODP-AM04-52	Granulicatella_adiacens	99.72	Bacillota	Granulicatella	0	34
SF3KC11004A	ODP-AM04-126	Neisseria_cinerea	97.94	Pseudomonadota	Neisseria	Neisseria flavescens_B	15
SF3KC12001	ODP-AM05-46	Neisseria_cinerea	98.56	Pseudomonadota	Neisseria	Neisseria subflava	15
SF3KC12002A	ODP-AM05-252	Streptococcus_pseudopneumoniae	99.50	Bacillota	Streptococcus	0	101
SF3KC12002AB	ODP-AM05-394	Streptococcus_sinensis	99.72	Bacillota	Streptococcus	Streptococcus sinensis	24
SF3KC12003AB	ODP-AM05-395	Streptococcus_constellatus	99.12	Bacillota	Streptococcus	Streptococcus constellatus	46
SF3KC12004	ODP-AM05-49	Streptococcus_mitis	99.79	Bacillota	Streptococcus	Streptococcus mitis_AZ	11
SF3KC12004AB	ODP-AM05-396	Fusobacterium_nucleatum	99.85	Fusobacteriota	Fusobacterium	Fusobacterium polymorphum	87
SF3KC12005AB	ODP-AM05-464	Leptotrichia_wadei	98.04	Fusobacteriota	Leptotrichia	Leptotrichia wadei_A	152
SF3KC13002A	ODP-TM06-166	Actinomyces_odontolyticus	98.86	Actinomycetota	Pauljensenia	Pauljensenia odontolytica_A	27
SF3KC13003A	ODP-TM06-167	Actinomyces_odontolyticus	96.81	Actinomycetota	Pauljensenia	Pauljensenia sp000185285	153
SF3KC14001B	ODP-TM07-212	Streptococcus_oralis	99.84	Bacillota	Streptococcus	Streptococcus mitis_AZ	11
SF3KC15002	ODP-AM08-250	Rothia_dentocariosa	99.93	Actinomycetota	Rothia	Rothia dentocariosa	13
SF3KC15004A	ODP-AM08-316	Abiotrophia_defectiva	99.66	Bacillota	Abiotrophia	Abiotrophia defectiva	37
SF3KC15013A	ODP-AM08-325	Granulicatella_adiacens	100.00	Bacillota	Granulicatella	Granulicatella adiacens	23
SF3KC16006	ODP-AF06-107	Streptococcus_oralis	99.32	Bacillota	Streptococcus	0	154
SF3KT08005A	ORT-AF03-190	Atopobium_parvulum	99.58	Actinomycetota	Lancefieldella	0	155
SF3KT09001	ORT-AF04-29	Streptococcus_salivarius	100.00	Bacillota	Streptococcus	Streptococcus salivarius	4
SF3KT09001A	ORT-AF04-121	Streptococcus_salivarius	99.82	Bacillota	Streptococcus	Streptococcus salivarius	4
SF3KT09002	ORT-AF04-30	Streptococcus_salivarius	99.86	Bacillota	Streptococcus	Streptococcus salivarius	4
SF3KT09003A	ORT-AF04-228	Streptococcus_salivarius	100.00	Bacillota	Streptococcus	Streptococcus salivarius	4
SF3KT09004A	ORT-AF04-248	Granulicatella_adiacens	93.00	Bacillota	Granulicatella	Granulicatella sp001058355	95
SF3KT10004A	ORT-AF05-139	Atopobium_parvulum	99.72	Actinomycetota	Lancefieldella	0	96
SF3KT10008A	ORT-AF05-234	Actinomyces_odontolyticus	99.29	Actinomycetota	Pauljensenia	Pauljensenia sp001064145	42
SF3KT11001	ORT-AM04-26	Rothia_mucilaginosa	99.58	Actinomycetota	Rothia	Rothia mucilaginosa_B	59
SF3KT11002A	ORT-AM04-73	Granulicatella_adiacens	99.92	Bacillota	Granulicatella	Granulicatella adiacens	23
SF3KT11005A	ORT-AM04-81	Granulicatella_adiacens	93.62	Bacillota	Granulicatella	Granulicatella adiacens	23
SF3KT12001AB	ORT-AM05-107	Granulicatella_adiacens	99.66	Bacillota	Granulicatella	0	25
SF3KT12001B	ORT-AM05-308	Rothia_mucilaginosa	99.44	Actinomycetota	Rothia	Rothia mucilaginosa_B	59
SF3KT12002A	ORT-AM05-404	Veillonella_tobetsuensis	99.52	Bacillota	Veillonella	Veillonella parvula_A	92
SF3KT12002AB	ORT-AM05-108	Streptococcus_salivarius	99.86	Bacillota	Streptococcus	Streptococcus salivarius	4
SF3KT12003AB	ORT-AM05-109	Granulicatella_adiacens	99.66	Bacillota	Granulicatella	0	25
SF3KT13001A	ORT-TM06-205	Streptococcus_salivarius	100.00	Bacillota	Streptococcus	Streptococcus salivarius	4
SF3KT13003A	ORT-TM06-207	Prevotella_melaninogenica	99.29	Bacteroidota	Prevotella	Prevotella sp000257925	62

SF3KT13004A	ORT-TM06-208	Atopobium_parvulum	99.72	Actinomycetota	Lancefieldella	0	149
SF3KT13006A	ORT-TM06-210	Prevotella_histicola	99.38	Bacteroidota	Prevotella	Prevotella histicola	28
SF3KT13007A	ORT-TM06-211	Actinomyces_odontolyticus	98.37	Actinomycetota	Pauljensenia	Pauljensenia sp000466265	105
SF3KT13008A	ORT-TM06-212	Prevotella_histicola	99.89	Bacteroidota	Prevotella	Prevotella histicola	28
SF3KT13012A	ORT-TM06-249	Granulicatella_adiacens	99.72	Bacillota	Granulicatella	Granulicatella sp015264885	103
SF3KT14001B	ORT-TM07-208	Rothia_mucilaginosa	99.51	Actinomycetota	Rothia	Rothia mucilaginosa_B	59
SF3KT15002	ORT-AM08-487	Rothia_mucilaginosa	99.58	Actinomycetota	Rothia	Rothia mucilaginosa	78
SF3KT16002	ORT-AF06-16	Streptococcus_infantis	99.38	Bacillota	Streptococcus	Streptococcus infantis_H	73
SF3KT16005	ORT-AF06-19	Microbacterium_oxydans	99.79	Actinomycetota	Microbacterium	Microbacterium algeriense	12
SF3KT16008	ORT-AF06-143	Rothia_mucilaginosa	99.58	Actinomycetota	Rothia	Rothia mucilaginosa	78
T2011065150	ORS-AM09-1-2-O-BH	Streptococcus_mitis	99.31	Bacillota	Streptococcus	0	156
T2011065152	ORS-AM09-3-O-BH	Actinomyces_naeslundii	96.83	Actinomycetota	Actinomyces	Actinomyces oris_A	58
T2011065153	ORS-AM09-4-O-BH	Streptococcus_parasanguinis	99.79	Bacillota	Streptococcus	Streptococcus parasanguinis	20
T2011065154	ORS-AM09-5-O-BH	Streptococcus_parasanguinis	98.50	Bacillota	Streptococcus	Streptococcus parasanguinis_C	53
T2011065155	ORS-AM09-6-O-BH	Streptococcus_parasanguinis	99.79	Bacillota	Streptococcus	Streptococcus parasanguinis	20
T2011065157	ORS-AM09-8-O-BH	Streptococcus_pseudopneumoniae	99.79	Bacillota	Streptococcus	Streptococcus symci	157
T2011065158	ORS-AM09-9-O-BH	Streptococcus_pseudopneumoniae	99.79	Bacillota	Streptococcus	Streptococcus symci	157
T2011065159	ORS-AM09-10-O-BH	Streptococcus_pseudopneumoniae	99.79	Bacillota	Streptococcus	Streptococcus symci	157
T2011065161	ORS-AM09-12-2-O-BH	Streptococcus_parasanguinis	99.11	Bacillota	Streptococcus	Streptococcus sp001813295	6
T2011065165	ORS-AM09-16-O-BH	Neisseria_macacae /Gemella_haemolysans	99.86/98.58	Pseudomonadota	Neisseria	Neisseria sicca_D	136
T2011065166	ORS-AM09-17-O-BH	Streptococcus_pseudopneumoniae	99.79	Bacillota	Streptococcus	Streptococcus symci	157
T2011065167	ORS-AM09-18-O-BH	Streptococcus_parasanguinis	99.04	Bacillota	Streptococcus	Streptococcus sp001813295	6
T2011065168	ORS-AM09-19-O-BH	Streptococcus_pseudopneumoniae	99.65	Bacillota	Streptococcus	0	158
T2011065169	ORS-AM09-20-O-BH	Streptococcus_cristatus	99.45	Bacillota	Streptococcus	Streptococcus cristatus	36
T2011065170	ORS-AM09-22-O-BH	Streptococcus_infantis	99.38	Bacillota	Streptococcus	Streptococcus infantis_H	73
T2011065171	ORS-AM09-23-O-BH	Actinomyces_naeslundii	97.10	Actinomycetota	Actinomyces	Actinomyces oris	39
T2011065172	ORS-AM09-24-O-BH	Streptococcus_oralis	98.97	Bacillota	Streptococcus	Streptococcus infantis_B	159
T2011065173	ORS-AM09-25-O-BH	Rothia_mucilaginosa	99.24	Actinomycetota	Rothia	Rothia mucilaginosa_B	59
T2011065174	ORS-AM09-26-O-BH	Streptococcus_parasanguinis	99.04	Bacillota	Streptococcus	Streptococcus sp001813295	6
T2011065176	ORS-AM09-28-O-BH	Streptococcus_parasanguinis /Haemophilus_parainfluenzae	99.25/99.24	Bacillota	Streptococcus	Streptococcus sp001813295	6
T2011065177	ORS-AM09-29-O-BH	Streptococcus_parasanguinis	99.79	Bacillota	Streptococcus	Streptococcus parasanguinis	20
T2011065178	ORS-AM09-30-O-BH	Actinomyces_naeslundii	99.03	Actinomycetota	Actinomyces	Actinomyces oris	39
T2011065179	ORS-AM09-31-1-O-BH	Neisseria_subflava	100.00	Pseudomonadota	Neisseria	Neisseria flavescens_B	15
T2011065180	ORS-AM09-31-2-O-BH	Neisseria_subflava	100.00	Pseudomonadota	Neisseria	Neisseria flavescens_B	15
T2011065182	ORS-AM09-33-O-BH	Streptococcus_salivarius	100.00	Bacillota	Streptococcus	Streptococcus salivarius	4
T2011065185	ORS-AM09-36-O-BH	Streptococcus_parasanguinis	98.44	Bacillota	Streptococcus	Streptococcus sp900766505	6
T2011065187	ORS-AM09-39-O-BH	Streptococcus_oralis	98.50	Bacillota	Streptococcus	Streptococcus infantis_B	159
T2011065188	ORS-AM09-40-O-BH	Streptococcus_oralis	99.31	Bacillota	Streptococcus	0	160
T2011065189	ORS-AM09-41-O-BH	Neisseria_subflava	100.00	Pseudomonadota	Neisseria	Neisseria flavescens_B	15
T2011065191	ORS-AM09-43-O-BH	Streptococcus_mitis	99.31	Bacillota	Streptococcus	Streptococcus oralis_BD	79
T2011065192	ORS-AM09-45-O-BH	Streptococcus_mitis	99.86	Bacillota	Streptococcus	0	161
T2011065196	ODP-AM09-1-1-O-BH	Streptococcus_oralis	99.65	Bacillota	Streptococcus	0	162
T2011065197	ODP-AM09-1-2-O-BH	Streptococcus_oralis	99.65	Bacillota	Streptococcus	0	162
T2011065200	ODP-AM09-3-O-BH	Actinomyces_naeslundii	98.97	Actinomycetota	Actinomyces	Actinomyces oris_A	58
T2011065201	ODP-AM09-4-O-BH	Streptococcus_sanguinis	99.72	Bacillota	Streptococcus	Streptococcus sanguinis_H	3
T2011065202	ODP-AM09-5-O-BH	Streptococcus_sanguinis	99.72	Bacillota	Streptococcus	Streptococcus sanguinis_H	3
T2011065206	ODP-AM09-9-O-BH	Streptococcus_pseudopneumoniae	99.79	Bacillota	Streptococcus	Streptococcus symci	157
T2011065207	ODP-AM09-10-O-BH	Streptococcus_oralis /Granulicatella_adiacens	98.57/99.72	Bacillota	Streptococcus	Streptococcus infantis_B	159
T2011065209	ODP-AM09-12-O-BH	Streptococcus_pseudopneumoniae	99.79	Bacillota	Streptococcus	Streptococcus symci	157
T2011065210	ODP-AM09-13-O-BH	Streptococcus_gordonii	99.66	Bacillota	Streptococcus	Streptococcus gordonii	63
T2011065213	ODP-AM09-16-O-BH	Streptococcus_mitis	99.59	Bacillota	Streptococcus	Streptococcus oralis_BA	163
T2011065214	ODP-AM09-17-O-BH	Streptococcus_parasanguinis	99.04	Bacillota	Streptococcus	Streptococcus sp001813295	6
T2011065216	ODP-AM09-19-O-BH	Bacillus_thuringiensis	100.00	Bacillota	Bacillus_A	Bacillus_A mobilis	164
T2011065217	ODP-AM09-20-O-BH	Streptococcus_pseudopneumoniae	99.79	Bacillota	Streptococcus	Streptococcus symci	157
T2011065220	ODP-AM09-23-O-BH	Streptococcus_parasanguinis	99.04	Bacillota	Streptococcus	Streptococcus sp001813295	6
T2011065222	ODP-AM09-25-O-BH	#N/A	#N/A	Bacillota	Streptococcus	Streptococcus sp001813295	6
T2011065225	ODP-AM09-28-O-BH	Actinomyces_naeslundii	96.83	Actinomycetota	Actinomyces	Actinomyces oris_A	58
T2011065226	ODP-AM09-29-O-BH	Streptococcus_sanguinis	99.93	Bacillota	Streptococcus	Streptococcus sanguinis	3
T2011065228	ODP-AM09-31-O-BH	Streptococcus_gordonii	99.66	Bacillota	Streptococcus	Streptococcus gordonii	63
T2011065230	ODP-AM09-33-O-BH	Streptococcus_gordonii	99.66	Bacillota	Streptococcus	Streptococcus gordonii	63
T2011065231	ODP-AM09-34-O-BH	Actinomyces_naeslundii	97.10	Actinomycetota	Actinomyces	Actinomyces oris	39
T2011065233	ODP-AM09-36-O-BH	Streptococcus_pseudopneumoniae /Gemella_haemolysans /Streptococcus_mitis	100/82.64/100	Bacillota	Streptococcus	Streptococcus symci	157
T2011065234	ODP-AM09-37-O-BH	Streptococcus_oralis	99.25	Bacillota	Streptococcus	0	120
T2011065235	ODP-AM09-38-O-BH	Streptococcus_pseudopneumoniae	99.79	Bacillota	Streptococcus	Streptococcus symci	157
T2011065236	ODP-AM09-39-O-BH	Streptococcus_pseudopneumoniae	95.23	Bacillota	Streptococcus	Streptococcus mitis_AC	85
T2011065238	ODP-AM09-41-O-BH	Streptococcus_rubneri	99.17	Bacillota	Streptococcus	Streptococcus xiaochunlingii	5
T2011065239	ODP-AM09-42-O-BH	Streptococcus_pseudopneumoniae	99.79	Bacillota	Streptococcus	Streptococcus symci	157
T2011065241	ODP-AM09-44-O-BH	Actinomyces_naeslundii	99.13	Actinomycetota	Actinomyces	Actinomyces oris_A	58
T2011065242	ODP-AM09-45-O-BH	Streptococcus_pseudopneumoniae	99.79	Bacillota	Streptococcus	Streptococcus symci	157
T2011065243	ODP-AM09-46-O-BH	Streptococcus_mitis	99.59	Bacillota	Streptococcus	Streptococcus oralis_BA	163
T2011065244	ODP-AM09-47-O-BH	Streptococcus_pseudopneumoniae	99.79	Bacillota	Streptococcus	Streptococcus symci	157
T2011065245	ODP-AM09-48-O-BH	Streptococcus_gordonii	99.66	Bacillota	Streptococcus	Streptococcus gordonii	63
T2011065246	ODP-AM09-49-O-BH	Streptococcus_mitis	99.31	Bacillota	Streptococcus	Streptococcus oralis_BD	79
T2011068060	ORS-AM09-2-BH	Actinomyces_odontolyticus	99.08	Actinomycetota	Pauljensenia	Pauljensenia sp001064145	42
T2011068063	ORS-AM09-5-BH	Lachnoanaerobaculum_umeaense	94.80	Bacillota	Lachnoanaerobaculum	Lachnoanaerobaculum sp000296385	165
T2011068065	ORS-AM09-7-BH	Granulicatella_adiacens	99.72	Bacillota	Granulicatella	Granulicatella sp001058355	95
T2011068069	ORS-AM09-12-BH	Actinomyces_odontolyticus	99.08	Actinomycetota	Pauljensenia	Pauljensenia sp001064145	42
T2011068071	ORS-AM09-16-BH	Streptococcus_mitis	99.59	Bacillota	Streptococcus	Streptococcus oralis_BA	163
T2011068073	ORS-AM09-18-BH	Actinomyces_odontolyticus	99.13	Actinomycetota	Pauljensenia	Pauljensenia sp000411415	166
T2011068074	ORS-AM09-19-BH	Gemella_sanguinis	99.86	Bacillota	Gemella	Gemella sanguinis	17
T2011068075	ORS-AM09-20-BH	Streptococcus_salivarius	99.86	Bacillota	Streptococcus	Streptococcus salivarius	4
T2011068076	ORS-AM09-22-BH	Actinomyces_odontolyticus /Enterococcus_faecium	98.73/99.42	Actinomycetota	Pauljensenia	Pauljensenia sp000278725	121
T2011068079	ORS-AM09-26-BH	Enterococcus_durans /Actinomyces_odontolyticus	74.08/98.37	Actinomycetota	Pauljensenia	0	167

T2011068080	ORS-AM09-27-BH	Streptococcus_sanguinis	99.72	Bacillota	Streptococcus	Streptococcus sanguinis_H	3
T2011068081	ORS-AM09-28-BH	Streptococcus_australis	99.38	Bacillota	Streptococcus	Streptococcus sp004166885	98
T2011068082	ORS-AM09-29-BH	Actinomyces_naeslundii	99.36	Actinomycetota	Actinomyces	Actinomyces oris_A	58
T2011068083	ORS-AM09-30-BH	Streptococcus_salivarius	99.86	Bacillota	Streptococcus	Streptococcus salivarius	4
T2011068084	ORS-AM09-31-BH	Actinomyces_odontolyticus	98.44	Actinomycetota	Pauljensenia	Pauljensenia sp000466265	105
T2011068085	ORS-AM09-32-BH	Gemella_haemolysans	99.93	Bacillota	Gemella	Gemella haemolysans_B	49
T2011068087	ODP-AM09-1-BH	Streptococcus_pseudopneumoniae	99.79	Bacillota	Streptococcus	Streptococcus symci	157
T2011068089	ODP-AM09-3-BH	Streptococcus_pseudopneumoniae	99.79	Bacillota	Streptococcus	Streptococcus symci	157
T2011068093	ODP-AM09-7-BH	Streptococcus_pseudopneumoniae	99.79	Bacillota	Streptococcus	Streptococcus symci	157
T2011068096	ODP-AM09-11-BH	Streptococcus_gordonii	99.66	Bacillota	Streptococcus	Streptococcus gordonii	63
T2011068099	ODP-AM09-14-BH	Streptococcus_cristatus	99.65	Bacillota	Streptococcus	0	168
T2011068107	ODP-AM09-22-BH	Streptococcus_pseudopneumoniae	99.79	Bacillota	Streptococcus	Streptococcus symci	157
T2011068108	ODP-AM09-23-BH	Streptococcus_mitis	99.65	Bacillota	Streptococcus	Streptococcus oralis	107
T2011068109	ODP-AM09-24-BH	Streptococcus_pseudopneumoniae	99.79	Bacillota	Streptococcus	Streptococcus symci	157
T2011068112	ODP-AM09-27-BH	Streptococcus_mitis	99.86	Bacillota	Streptococcus	0	161
T2011068113	ODP-AM09-30-BH	Streptococcus_parasanguinis	98.50	Bacillota	Streptococcus	Streptococcus parasanguinis_C	53
T2011068114	ODP-AM09-32-BH	Streptococcus_pseudopneumoniae	99.79	Bacillota	Streptococcus	Streptococcus symci	157
T2011068115	ODP-AM09-33-BH	Streptococcus_mitis	99.45	Bacillota	Streptococcus	Streptococcus mitis_AW	169
T2011068116	ODP-AM09-34-BH	Streptococcus_constellatus	99.12	Bacillota	Streptococcus	Streptococcus constellatus	46
T2011068119	ODP-AM09-37-BH	Actinomyces_odontolyticus	98.86	Actinomycetota	Pauljensenia	Pauljensenia odontolytica_A	27
T2011068120	ODP-AM09-39-BH	Streptococcus_gordonii	99.66	Bacillota	Streptococcus	Streptococcus gordonii	63
T2011068121	ODP-AM09-41-BH	Actinomyces_naeslundii	99.13	Actinomycetota	Actinomyces	Actinomyces oris	39
T2011068122	ODP-AM09-43-BH	Streptococcus_gordonii	99.66	Bacillota	Streptococcus	Streptococcus gordonii	63
T2101076727	ORT-AM09-2-O-104	Streptococcus_pseudopneumoniae	99.52	Bacillota	Streptococcus	0	170
T2101076728	ORT-AM09-3-O-104	Streptococcus_pseudopneumoniae	99.79	Bacillota	Streptococcus	Streptococcus symci	157
T2101076730	ORT-AM09-5-O-104	Streptococcus_parasanguinis	98.57	Bacillota	Streptococcus	Streptococcus parasanguinis_C	53
T2101076731	ORT-AM09-6-O-104	Streptococcus_mitis	99.04	Bacillota	Streptococcus	Streptococcus oralis_K	79
T2101076732	ORT-AM09-7-O-104	Streptococcus_sanguinis	99.86	Bacillota	Streptococcus	Streptococcus sanguinis_H	3
T2101076733	ORT-AM09-8-O-104	Streptococcus_infantis	99.59	Bacillota	Streptococcus	0	154
T2101076734	ORT-AM09-9-O-104	Streptococcus_mitis	99.11	Bacillota	Streptococcus	0	171
T2101076735	ORT-AM09-10-O-104	Granulicatella_adiacens	95.65	Bacillota	Granulicatella	0	77
T2101076737	ORT-AM09-12-O-104	Streptococcus_timonensis	99.04	Bacillota	Streptococcus	0	127
T2101076738	ORT-AM09-13-O-104	Streptococcus_australis	99.76	Bacillota	Streptococcus	0	68
T2101076739	ORT-AM09-14-O-104	Streptococcus_australis	96.46	Bacillota	Streptococcus	Streptococcus koreensis	48
T2101076741	ORT-AM09-18-O-104	Streptococcus_mitis	99.31	Bacillota	Streptococcus	0	172
T2101076743	ORT-AM09-20-O-104	Streptococcus_infantis	99.59	Bacillota	Streptococcus	0	154
T2101076744	ORT-AM09-21-O-104	Streptococcus_oralis	99.91	Bacillota	Streptococcus	Streptococcus oralis_AC	173
T2101076745	ORT-AM09-22-O-104	Streptococcus_mitis	99.65	Bacillota	Streptococcus	Streptococcus oralis	107
T2101076746	ORT-AM09-23-O-104	Streptococcus_australis	99.72	Bacillota	Streptococcus	0	68
T2101076747	ORT-AM09-24-O-104	Streptococcus_mitis	99.65	Bacillota	Streptococcus	Streptococcus oralis	107
T2101076748	ORT-AM09-25-O-104	Gemella_sanguinis	99.93	Bacillota	Gemella	Gemella sanguinis	17
T2101076749	ORT-AM09-26-O-104	Streptococcus_rubneri	99.90	Bacillota	Streptococcus	0	68
T2101076750	ORT-AM09-28-O-104	Streptococcus_rubneri	97.37	Bacillota	Streptococcus	0	68
T2101076751	ORT-AM09-29-O-104	Streptococcus_australis	99.14	Bacillota	Streptococcus	Streptococcus koreensis	48
T2101076754	ODP-AM09-2-O-104	Streptococcus_pseudopneumoniae	99.52	Bacillota	Streptococcus	0	170
T2101076755	ODP-AM09-3-O-104	Streptococcus_pseudopneumoniae	99.52	Bacillota	Streptococcus	0	170
T2101076756	ODP-AM09-4-O-104	Streptococcus_constellatus	99.12	Bacillota	Streptococcus	Streptococcus constellatus	46
T2101076757	ODP-AM09-5-O-104	Streptococcus_sanguinis	99.86	Bacillota	Streptococcus	Streptococcus sanguinis_H	3
T2101076758	ODP-AM09-6-O-104	Streptococcus_mitis	99.72	Bacillota	Streptococcus	Streptococcus sp900555155	107
T2101076761	ODP-AM09-9-O-104	Streptococcus_mitis	99.65	Bacillota	Streptococcus	Streptococcus oralis	107
T2101076762	ODP-AM09-10-O-104	Streptococcus_mitis	99.65	Bacillota	Streptococcus	Streptococcus oralis	107
T2101076764	ODP-AM09-12-O-104	Streptococcus_constellatus	99.86	Bacillota	Streptococcus	Streptococcus constellatus	46
T2101076767	ODP-AM09-16-O-104	Streptococcus_mitis	99.65	Bacillota	Streptococcus	Streptococcus oralis	107
T2101076768	ODP-AM09-18-O-104	Streptococcus_pseudopneumoniae	99.79	Bacillota	Streptococcus	Streptococcus symci	157
T2101076769	ODP-AM09-19-O-104	Streptococcus_pseudopneumoniae	99.79	Bacillota	Streptococcus	Streptococcus symci	157
T2101076770	ODP-AM09-20-O-104	Streptococcus_pseudopneumoniae	99.52	Bacillota	Streptococcus	0	170
T2101076771	ODP-AM09-21-O-104	Streptococcus_mitis	99.65	Bacillota	Streptococcus	Streptococcus sp900555155	107
T2101076772	ODP-AM09-22-O-104	Streptococcus_mitis	99.65	Bacillota	Streptococcus	Streptococcus sp900555155	107
T2101076773	ODP-AM09-23-O-104	Streptococcus_mitis	99.65	Bacillota	Streptococcus	Streptococcus oralis	107
T2101076774	ORS-AM09-1-O-104	Streptococcus_mitis /Streptococcus_timonensis	99.56/97.31	Bacillota	Streptococcus	0	86
T2101076775	ORS-AM09-2-O-104	Streptococcus_pseudopneumoniae	99.79	Bacillota	Streptococcus	0	174
T2101076776	ORS-AM09-3-O-104	Streptococcus_australis	99.76	Bacillota	Streptococcus	0	68
T2101076777	ORS-AM09-4-O-104	Streptococcus_mitis	99.52	Bacillota	Streptococcus	Streptococcus oralis	107
T2101076780	ORS-AM09-7-O-104	Streptococcus_gordonii	99.59	Bacillota	Streptococcus	Streptococcus gordonii	63
T2101076781	ORS-AM09-8-O-104	Streptococcus_salivarius	100.00	Bacillota	Streptococcus	Streptococcus salivarius	4
T2101076782	ORS-AM09-9-O-104	Streptococcus_mitis	99.52	Bacillota	Streptococcus	Streptococcus oralis	107
T2101076783	ORS-AM09-10-O-104	Streptococcus_sanguinis	100.00	Bacillota	Streptococcus	Streptococcus sanguinis_C	113
T2101076784	ORS-AM09-11-O-104	Streptococcus_mitis	99.65	Bacillota	Streptococcus	Streptococcus oralis	107
T2101076786	ORS-AM09-13-O-104	Streptococcus_mitis	99.31	Bacillota	Streptococcus	0	172
T2101076787	ORS-AM09-14-O-104	Streptococcus_mitis	99.31	Bacillota	Streptococcus	0	172
T2101076788	ORS-AM09-15-O-104	Streptococcus_salivarius	99.86	Bacillota	Streptococcus	Streptococcus salivarius	4
T2101076789	ORS-AM09-16-O-104	Streptococcus_salivarius	100.00	Bacillota	Streptococcus	Streptococcus salivarius	4
T2101076791	ORS-AM09-21-O-104	Streptococcus_salivarius	99.86	Bacillota	Streptococcus	Streptococcus salivarius	4
T2101076792	ORS-AM09-23-O-104	Streptococcus_parasanguinis	98.57	Bacillota	Streptococcus	Streptococcus parasanguinis_C	53
T2101076793	ORS-AM09-24-O-104	Streptococcus_australis	99.45	Bacillota	Streptococcus	0	68
T2101076794	ORS-AM09-27-O-104	Streptococcus_mitis	99.65	Bacillota	Streptococcus	Streptococcus oralis	107
T2101076795	ORS-AF08-1-MRS	Streptococcus_oralis	99.32	Bacillota	Streptococcus	0	120
T2101076796	ORS-AF08-2-MRS	Streptococcus_salivarius	100.00	Bacillota	Streptococcus	Streptococcus salivarius	4
T2101076797	ORS-AF08-3-MRS	Granulicatella_adiacens	99.72	Bacillota	Granulicatella	0	77
T2101076798	ORS-AF08-4-MRS	Streptococcus_mitis	99.65	Bacillota	Streptococcus	Streptococcus oralis	107
T2101076799	ORS-AF08-5-MRS	Streptococcus_salivarius	100.00	Bacillota	Streptococcus	Streptococcus salivarius	4
T2101076801	ORS-AF08-7-MRS	Streptococcus_salivarius	99.86	Bacillota	Streptococcus	Streptococcus salivarius	4
T2101076802	ORS-AF08-8-MRS	Granulicatella_adiacens /Streptococcus_pseudopneumoniae	99.66/99.52	Bacillota	Streptococcus	0	170
T2101076803	ORS-AF08-9-MRS	Prevotella_melaninogenica	99.68	Bacteroidota	Prevotella	Prevotella melaninogenica	175
T2101076804	ORS-AF08-12-MRS	Streptococcus_pseudopneumoniae	99.79	Bacillota	Streptococcus	Streptococcus symci	157

T2101076805	ORS-AF08-14-MRS	Granulicatella_adiacens	99.59	Bacillota	Granulicatella	Granulicatella sp001058355	106
T2101076807	ORS-AF08-16-MRS	Streptococcus_timonensis	98.91	Bacillota	Streptococcus	0	176
T2101076808	ORS-AF08-17-MRS	Streptococcus_pseudopneumoniae	99.79	Bacillota	Streptococcus	Streptococcus symci	157
T2101076809	ORS-AF08-18-MRS	Streptococcus_australis	96.53	Bacillota	Streptococcus	Streptococcus koreensis	48
T2101076810	ORS-AF08-19-MRS	Prevotella_melaninogenica	97.86	Bacteroidota	Prevotella	Prevotella melaninogenica	175
T2101076811	ORS-AF08-20-MRS	Streptococcus_salivarius	99.86	Bacillota	Streptococcus	Streptococcus salivarius	4
T2101076813	ORS-AF08-23-MRS	Streptococcus_salivarius	100.00	Bacillota	Streptococcus	Streptococcus salivarius	4
T2101076815	ORS-AF08-25-MRS	Streptococcus_australis	96.53	Bacillota	Streptococcus	Streptococcus koreensis	48
T2101076817	ORS-AF08-27-MRS	Actinomyces_odontolyticus /Granulicatella_adiacens	98.86/99.72	Bacillota	Granulicatella	Granulicatella sp001058355	106
T2101076820	ORS-AF08-30-MRS	Streptococcus_salivarius	100.00	Bacillota	Streptococcus	Streptococcus salivarius	4
T2101076821	ODP-AF08-1-MRS	Streptococcus_sanguinis	99.86	Bacillota	Streptococcus	Streptococcus sanguinis_H	3
T2101076822	ODP-AF08-2-MRS	Streptococcus_anginosus	99.66	Bacillota	Streptococcus	Streptococcus anginosus_C	104
T2101076825	ODP-AF08-7-MRS	Streptococcus_mitis	99.65	Bacillota	Streptococcus	Streptococcus oralis	107
T2101076826	ODP-AF08-8-MRS	Streptococcus_mitis	99.65	Bacillota	Streptococcus	Streptococcus oralis	107
T2101076827	ODP-AF08-10-MRS	Streptococcus_mitis	99.52	Bacillota	Streptococcus	Streptococcus oralis	107
T2101076828	ODP-AF08-11-MRS	Streptococcus_sanguinis	99.93	Bacillota	Streptococcus	Streptococcus sanguinis	3
T2101076829	ODP-AF08-13-MRS	Streptococcus_constellatus	99.86	Bacillota	Streptococcus	Streptococcus constellatus	46
T2101076831	ODP-AF08-15-MRS	Streptococcus_anginosus	99.66	Bacillota	Streptococcus	Streptococcus anginosus	61
T2101076832	ODP-AF08-16-MRS	Streptococcus_constellatus	99.86	Bacillota	Streptococcus	Streptococcus constellatus	46
T2101076833	ODP-AF08-17-MRS	Streptococcus_sanguinis	100.00	Bacillota	Streptococcus	Streptococcus sanguinis_C	113
T2101076834	ODP-AF08-18-MRS	Streptococcus_sanguinis	99.86	Bacillota	Streptococcus	Streptococcus sanguinis_H	3
T2101076835	ODP-AF08-20-MRS	Streptococcus_constellatus	99.86	Bacillota	Streptococcus	Streptococcus constellatus	46
T2101076836	ODP-AF08-21-MRS	Streptococcus_mitis	99.52	Bacillota	Streptococcus	Streptococcus oralis	107
T2101076837	ODP-AF08-22-MRS	Veillonella_parvula	99.73	Bacillota	Veillonella	Veillonella sp900757715	177
T2101076838	ODP-AF08-23-MRS	Streptococcus_mitis	99.65	Bacillota	Streptococcus	Streptococcus oralis	107
T2101076840	ODP-AF08-27-MRS	Streptococcus_sanguinis	99.86	Bacillota	Streptococcus	Streptococcus sanguinis_H	3
T2101076841	ODP-AF08-28-MRS	Granulicatella_adiacens	88.51	Bacillota	Granulicatella	Granulicatella adiacens	23
T2101076844	ODP-AF08-35-MRS	Streptococcus_mitis	99.52	Bacillota	Streptococcus	Streptococcus oralis	107
T2101076845	ODP-AF08-36-MRS	Streptococcus_mitis	99.65	Bacillota	Streptococcus	Streptococcus oralis	107
T2101076846	ODP-AF08-37-MRS	Granulicatella_adiacens	99.86	Bacillota	Granulicatella	Granulicatella adiacens	23
T2101076847	ODP-AF08-38-MRS	Streptococcus_oralis	99.70	Bacillota	Streptococcus	Streptococcus mitis_AZ	11
T2101076848	ODP-AF08-39-MRS	Streptococcus_pseudopneumoniae	99.52	Bacillota	Streptococcus	0	170
T2101076849	ODP-AF08-40-MRS	Streptococcus_constellatus	99.86	Bacillota	Streptococcus	Streptococcus constellatus	46
T2101076850	ODP-AF08-41-MRS	Streptococcus_mitis	99.65	Bacillota	Streptococcus	Streptococcus oralis	107
T2101076851	ODP-AF08-42-MRS	Streptococcus_pseudopneumoniae	99.52	Bacillota	Streptococcus	0	170
T2101076854	ORT-AF08-11-MRS	Veillonella_atypica	99.52	Bacillota	Veillonella	Veillonella atypica	178
T2101076857	ORT-AF08-19-MRS	Streptococcus_sanguinis	99.86	Bacillota	Streptococcus	Streptococcus sanguinis_H	3
T2101076858	ORT-AF08-24-MRS	Streptococcus_salivarius	99.86	Bacillota	Streptococcus	Streptococcus salivarius	4
T2101076859	ORT-AF08-28-MRS	Streptococcus_constellatus	99.86	Bacillota	Streptococcus	Streptococcus constellatus	46
T2101076860	ORT-AF08-32-MRS	Streptococcus_constellatus	99.86	Bacillota	Streptococcus	Streptococcus constellatus	46
T2101076861	ORT-AF08-34-MRS	Streptococcus_mitis	99.52	Bacillota	Streptococcus	Streptococcus oralis	107
T2101076862	ORT-AF08-35-MRS	Streptococcus_parasanguinis	98.57	Bacillota	Streptococcus	Streptococcus parasanguinis_C	53
T2101076866	ORT-AF08-43-MRS	Streptococcus_mitis	99.65	Bacillota	Streptococcus	Streptococcus sp900555155	107
T2101078470	ORT-AM09-5D4	Veillonella_atypica	99.12	Bacillota	Veillonella	0	179
T2101078472	ORT-AM09-5D6	Bifidobacterium_dentium	99.58	Actinomycetota	Bifidobacterium	Bifidobacterium dentium	180
T2101078474	ODP-AM09-2D1	Streptococcus_salivarius	99.86	Bacillota	Streptococcus	Streptococcus salivarius	4
T2101078475	ODP-AM09-2D2	Streptococcus_salivarius	99.86	Bacillota	Streptococcus	Streptococcus salivarius	4
T2101078477	ODP-AM09-2D4A	Streptococcus_salivarius	99.86	Bacillota	Streptococcus	Streptococcus salivarius	4
T2101078478	ODP-AM09-2D4B	Streptococcus_salivarius	99.86	Bacillota	Streptococcus	Streptococcus salivarius	4
T2101078479	ODP-AM09-2D5	Streptococcus_salivarius	100.00	Bacillota	Streptococcus	Streptococcus salivarius	4
T2101078481	ODP-AM09-2D6	Enterococcus_faecalis	100.00	Bacillota	Enterococcus	Enterococcus faecalis	21
T2101078490	ODP-AM09-2D16	Streptococcus_salivarius	99.86	Bacillota	Streptococcus	Streptococcus salivarius	4
T2101078492	ODP-AM09-2D18	Veillonella_dispar	90.32	Bacillota	Veillonella	Veillonella atypica	178
T2101078494	ODP-AM09-2D20	Streptococcus_salivarius	99.86	Bacillota	Streptococcus	Streptococcus salivarius	4
T2101078495	ODP-AM09-2D21	Veillonella_atypica	92.59	Bacillota	Veillonella	Veillonella atypica	178
T2101078500	ODP-AF08-11-O-MRS	Streptococcus_anginosus	99.72	Bacillota	Streptococcus	Streptococcus anginosus	61
T2101078504	ODP-AF08-18-O-MRS	Streptococcus_salivarius	100.00	Bacillota	Streptococcus	Streptococcus salivarius	4
T2101078508	ODP-AF08-25-O-MRS	Streptococcus_anginosus	99.72	Bacillota	Streptococcus	Streptococcus anginosus	61
T2101078509	ODP-AF08-26-O-MRS	Streptococcus_sanguinis	99.91	Bacillota	Streptococcus	Streptococcus sanguinis_H	3
T2101078510	ODP-AF08-27-O-MRS	Streptococcus_anginosus	99.72	Bacillota	Streptococcus	Streptococcus anginosus	61
T2101078518	ORT-AF08-12-O-MRS	Streptococcus_salivarius	100.00	Bacillota	Streptococcus	Streptococcus salivarius	4
T2101078520	ORT-AF08-14-O-MRS	Streptococcus_anginosus	99.72	Bacillota	Streptococcus	Streptococcus anginosus_C	104
T2101078525	ORT-AF08-20-O-MRS	Streptococcus_sanguinis	99.93	Bacillota	Streptococcus	Streptococcus sanguinis_H	3
T2101078526	ORT-AF08-21-O-MRS	Streptococcus_mitis	99.65	Bacillota	Streptococcus	Streptococcus oralis	107
T2101081681	ORS-AM09-1-104	Streptococcus_pseudopneumoniae	99.79	Bacillota	Streptococcus	0	158
T2101081682	ORS-AM09-2-104	Lachnoanaerobaculum_umeaense	93.46	Bacillota	Lachnoanaerobaculum	Lachnoanaerobaculum sp000296385	165
T2101081683	ORS-AM09-3-104	Gemella_haemolysans	99.72	Bacillota	Gemella	0	181
T2101081684	ORS-AM09-4-104	Streptococcus_oralis	99.25	Bacillota	Streptococcus	Streptococcus sp013394695	120
T2101081685	ORS-AM09-5-104	Streptococcus_mitis	99.11	Bacillota	Streptococcus	Streptococcus mitis_O	182
T2101081686	ORS-AM09-6-104	Streptococcus_parasanguinis	99.80	Bacillota	Streptococcus	Streptococcus sp900766505	6
T2101081688	ORS-AM09-8-104	Streptococcus_oralis	98.24	Bacillota	Streptococcus	0	160
T2101081689	ORS-AM09-9-104	Streptococcus_pseudopneumoniae	99.79	Bacillota	Streptococcus	Streptococcus symci	157
T2101081690	ORS-AM09-10-104	Streptococcus_mitis	99.31	Bacillota	Streptococcus	Streptococcus pseudopneumoniae_M	115
T2101081692	ORS-AM09-12-104	Streptococcus_salivarius	99.79	Bacillota	Streptococcus	Streptococcus salivarius	4
T2101081693	ORS-AM09-13-104	Streptococcus_parasanguinis	99.11	Bacillota	Streptococcus	Streptococcus sp001813295	6
T2101081695	ORS-AM09-15-104	Streptococcus_parasanguinis	92.36	Bacillota	Streptococcus	Streptococcus sp001813295	183
T2101081696	ORS-AM09-16-104	Streptococcus_mitis	99.59	Bacillota	Streptococcus	Streptococcus oralis_BA	163
T2101081698	ORS-AM09-18-104	Streptococcus_oralis	98.91	Bacillota	Streptococcus	Streptococcus infantis_B	159
T2101081699	ORS-AM09-19-104	Streptococcus_pseudopneumoniae	99.79	Bacillota	Streptococcus	Streptococcus symci	157
T2101081702	ORS-AM09-22-104	Streptococcus_pseudopneumoniae	99.79	Bacillota	Streptococcus	Streptococcus mitis_AC	85
T2101081708	ODP-AM09-3-104	Streptococcus_pseudopneumoniae	99.79	Bacillota	Streptococcus	Streptococcus symci	157
T2101081710	ODP-AM09-5-104	Streptococcus_mitis	99.59	Bacillota	Streptococcus	Streptococcus oralis_BA	163
T2101081711	ODP-AM09-6-104	Streptococcus_pseudopneumoniae	99.59	Bacillota	Streptococcus	0	158
T2101081713	ODP-AM09-8-104	Streptococcus_parasanguinis	98.91	Bacillota	Streptococcus	Streptococcus parasanguinis_E	6
T2101081714	ODP-AM09-9-104	Streptococcus_pseudopneumoniae	99.59	Bacillota	Streptococcus	0	158
T2101081715	ODP-AM09-10-104	Streptococcus_mitis	100.00	Bacillota	Streptococcus	Streptococcus symci	157

T2101081716	ODP-AM09-11-104	Streptococcus_anginosus	98.44	Bacillota	Streptococcus	Streptococcus anginosus_C	104
T2101081717	ODP-AM09-12-104	Streptococcus_pseudopneumoniae	99.65	Bacillota	Streptococcus	0	158
T2101081718	ODP-AM09-13-104	Streptococcus_pseudopneumoniae	99.59	Bacillota	Streptococcus	0	158
T2101081719	ODP-AM09-14-104	Streptococcus_oralis	99.32	Bacillota	Streptococcus	0	184
T2101081720	ODP-AM09-15-104	Streptococcus_pseudopneumoniae	99.79	Bacillota	Streptococcus	Streptococcus symci	157
T2101081722	ODP-AM09-17-104	Streptococcus_mitis	99.65	Bacillota	Streptococcus	Streptococcus oralis	107
T2101081723	ODP-AM09-18-104	Streptococcus_gordonii	99.66	Bacillota	Streptococcus	Streptococcus gordonii	63
T2101081725	ODP-AM09-20-104	Streptococcus_pseudopneumoniae	99.79	Bacillota	Streptococcus	0	158
T2101081726	ODP-AM09-21-104	Streptococcus_pseudopneumoniae	99.65	Bacillota	Streptococcus	0	158
T2101081727	ODP-AM09-22-104	Streptococcus_gordonii	99.66	Bacillota	Streptococcus	Streptococcus gordonii	63
T2101081728	ODP-AM09-23-104	Streptococcus_mitis	99.59	Bacillota	Streptococcus	Streptococcus oralis_BA	163
T2101081729	ODP-AM09-24-104	Streptococcus_parasanguinis	99.11	Bacillota	Streptococcus	Streptococcus sp001813295	6
T2101081731	ODP-AM09-26-104	Streptococcus_mitis	99.65	Bacillota	Streptococcus	Streptococcus oralis	107
T2101081732	ODP-AM09-27-104	Streptococcus_mitis	99.59	Bacillota	Streptococcus	Streptococcus oralis_BA	163
T2101081734	ORT-AM09-3-104	Streptococcus_oralis	99.25	Bacillota	Streptococcus	Streptococcus sp013394695	120
T2101081735	ORT-AM09-5-104	Streptococcus_parasanguinis	98.84	Bacillota	Streptococcus	Streptococcus sp900766505	6
T2101081736	ORT-AM09-6-104	Streptococcus_parasanguinis	99.04	Bacillota	Streptococcus	Streptococcus sp001813295	6
T2101081738	ORT-AM09-8-104	Streptococcus_oralis	98.44	Bacillota	Streptococcus	Streptococcus sp013394695	120
T2101081739	ORT-AM09-9-104	Streptococcus_parasanguinis	98.50	Bacillota	Streptococcus	Streptococcus parasanguinis_C	53
T2101081740	ORT-AM09-10-104	Streptococcus_oralis	99.07	Bacillota	Streptococcus	Streptococcus infantis_B	159
T2101081744	ORT-AM09-14-104	Lactococcus_lactis /Actinomyces_odontolyticus	89.97/98.94	Actinomycetota	Pauljensenia	Pauljensenia sp000411415	166
T2101081746	ORT-AM09-17-104	Streptococcus_parasanguinis	99.79	Bacillota	Streptococcus	Streptococcus parasanguinis	20
T2101081747	ORT-AM09-19-104	Streptococcus_parasanguinis	98.44	Bacillota	Streptococcus	Streptococcus sp900766505	6
T2101081750	ORT-AM09-23-104	Streptococcus_oralis	98.57	Bacillota	Streptococcus	Streptococcus infantis_B	159
T2102085941	ORS-AF08-20	Streptococcus_pseudopneumoniae	99.04	Bacillota	Streptococcus	0	170
T2102085942	ORS-AF08-21	Streptococcus_mitis	99.65	Bacillota	Streptococcus	Streptococcus oralis	107
T2102085943	ORS-AF08-22	Streptococcus_mitis	99.65	Bacillota	Streptococcus	Streptococcus oralis	107
T2102085944	ORS-AF08-23	Streptococcus_timonensis	96.86	Bacillota	Streptococcus	0	176
T2102085945	ORS-AF08-24	Streptococcus_mitis	99.64	Bacillota	Streptococcus	Streptococcus oralis	107
T2102085947	ORT-AF08-2	Streptococcus_anginosus	99.66	Bacillota	Streptococcus	Streptococcus anginosus	61
T2102085948	ORT-AF08-3	#N/A	#N/A	Bacillota	Streptococcus	Streptococcus constellatus	46
T2102085949	ORT-AF08-4	Streptococcus_oralis	99.77	Bacillota	Streptococcus	Streptococcus oralis_AC	173
T2102085950	ORT-AF08-5	Streptococcus_timonensis	99.72	Bacillota	Streptococcus	0	176
T2102085951	ORT-AF08-6	#N/A	#N/A	Bacillota	Streptococcus	Streptococcus salivarius	4
T2102085952	ORT-AF08-7	Streptococcus_mitis	99.11	Bacillota	Streptococcus	0	171
T2102085953	ORT-AF08-8	Streptococcus_salivarius	71.23	Bacillota	Streptococcus	Streptococcus salivarius	4
T2102085954	ORT-AF08-9	Streptococcus_salivarius	99.72	Bacillota	Streptococcus	Streptococcus salivarius	4
T2102085955	ORT-AF08-10	Streptococcus_infantis	99.45	Bacillota	Streptococcus	0	185
T2102085956	ORT-AF08-11	Streptococcus_mitis	99.32	Bacillota	Streptococcus	Streptococcus oralis	107
T2102085957	ORT-AF08-12	Streptococcus_salivarius	99.86	Bacillota	Streptococcus	Streptococcus salivarius	4
T2102085958	ORT-AF08-13	Streptococcus_mitis	99.65	Bacillota	Streptococcus	Streptococcus oralis	107
T2102085960	ORT-AF08-15	Streptococcus_salivarius	99.86	Bacillota	Streptococcus	Streptococcus salivarius	4
T2102085961	ORT-AF08-16	Streptococcus_mitis	99.04	Bacillota	Streptococcus	Streptococcus oralis_K	79
T2102085962	ORT-AF08-17	Streptococcus_infantis	99.45	Bacillota	Streptococcus	0	185
T2102085963	ORT-AF08-18	Streptococcus_australis	94.69	Bacillota	Streptococcus	0	68
T2102085964	ORT-AF08-19	Streptococcus_anginosus	83.05	Bacillota	Streptococcus	Streptococcus anginosus_C	104
T2102085965	ODP-AF08-2	Streptococcus_mitis	99.81	Bacillota	Streptococcus	Streptococcus oralis	107
T2102085966	ODP-AF08-3	Streptococcus_constellatus	99.86	Bacillota	Streptococcus	Streptococcus constellatus	46
T2102085968	ORS-AM09-5D1B	Veillonella_parvula	99.32	Bacillota	Veillonella	Veillonella parvula_A	92
T2102085969	ORS-AM09-5D2A	Veillonella_tobetsuensis	99.61	Bacillota	Veillonella	Veillonella parvula_A	92
T2102085974	ODP-AM09-2D3B	Veillonella_atypica	99.52	Bacillota	Veillonella	Veillonella atypica	178
T2102085975	ORS-AF08-13B	Granulicatella_adiacens	99.72	Bacillota	Granulicatella	0	106
T2102085978	ORT-AF08-8-MRS	Rothia_mucilaginosa	99.58	Actinomycetota	Rothia	Rothia mucilaginosa_B	59
T2102085980	ODP-AF08-20	Enterococcus_faecalis /Actinomyces_oris	99.93/96.8	Bacillota	Enterococcus	Enterococcus faecalis	21
T2103096290	ORS-AF10-1-O-BH	Actinomyces_naeslundii	99.36	Actinomycetota	Actinomyces	Actinomyces oris	39
T2103096291	ORS-AF10-5-O-BH	Actinomyces_naeslundii	98.04	Actinomycetota	Actinomyces	Actinomyces oris	39
T2103096292	ORS-AF10-6-O-BH	Neisseria_cinerea	97.88	Pseudomonadota	Neisseria	Neisseria flavescens_B	15
T2103096301	ORS-AF10-17-O-BH	Neisseria_cinerea	97.88	Pseudomonadota	Neisseria	Neisseria flavescens_B	15
T2103096302	ORS-AF10-18-O-BH	Rothia_dentocariosa	100.00	Actinomycetota	Rothia	Rothia dentocariosa	13
T2103096304	ORS-AF10-20-O-BH	Streptococcus_oralis	99.66	Bacillota	Streptococcus	Streptococcus sp900550895	186
T2103096306	ORS-AF10-23-O-BH	Actinomyces_naeslundii	99.29	Actinomycetota	Actinomyces	Actinomyces oris	39
T2103096307	ORS-AF10-24-O-BH	Neisseria_cinerea	97.88	Pseudomonadota	Neisseria	Neisseria flavescens_B	15
T2103096310	ORS-AF10-28-O-BH	Actinomyces_naeslundii /Gemella_haemolysans	97.37/99.93	Actinomycetota	Actinomyces	Actinomyces oris	39
T2103096316	ODP-AF10-3-O-BH	Actinomyces_naeslundii	97.97	Actinomycetota	Actinomyces	Actinomyces oris	39
T2103096318	ODP-AF10-6-O-BH	Rothia_dentocariosa	99.51	Actinomycetota	Rothia	Rothia dentocariosa	13
T2103096320	ODP-AF10-9-O-BH	Rothia_dentocariosa	99.93	Actinomycetota	Rothia	Rothia dentocariosa	13
T2103096321	ODP-AF10-10-O-BH	Rothia_dentocariosa	99.93	Actinomycetota	Rothia	Rothia dentocariosa	13
T2103096323	ODP-AF10-12-O-BH	Actinomyces_naeslundii	99.36	Actinomycetota	Actinomyces	Actinomyces oris	39
T2103096327	ODP-AF10-17-O-BH	Neisseria_macacae	100.00	Pseudomonadota	Neisseria	Neisseria sicca_B	187
T2103096348	ORT-AF10-10-O-BH	Streptococcus_salivarius	98.70	Bacillota	Streptococcus	Streptococcus salivarius	4
T2103096349	ORT-AF10-11-O-BH	Neisseria_cinerea	97.88	Pseudomonadota	Neisseria	Neisseria flavescens_B	15
T2103096352	ORT-AF10-14-O-BH	Streptococcus_oralis	99.11	Bacillota	Streptococcus	Streptococcus oralis_T	35
T2103096353	ORT-AF10-15-O-BH	Streptococcus_oralis	98.57	Bacillota	Streptococcus	Streptococcus infantis_B	159
T2103096354	ORT-AF10-16-O-BH	Rothia_dentocariosa	99.93	Actinomycetota	Rothia	Rothia dentocariosa	13
T2103096355	ORT-AF10-18-O-BH	Rothia_dentocariosa	99.93	Actinomycetota	Rothia	Rothia dentocariosa	13
T2103096356	ORT-AF10-19-O-BH	Actinomyces_naeslundii	97.37	Actinomycetota	Actinomyces	Actinomyces oris	39
T2103096357	ORT-AF10-20-O-BH	Streptococcus_gordonii	99.66	Bacillota	Streptococcus	Streptococcus gordonii	63
T2103096358	ORT-AF10-21-O-BH	Rothia_aeria	99.86	Actinomycetota	Rothia	Rothia aeria	32
T2103096359	ORT-AF10-22-O-BH	Rothia_dentocariosa	100.00	Actinomycetota	Rothia	Rothia dentocariosa	13
T2103096360	ORT-AF10-23-O-BH	Streptococcus_rubneri	96.45	Bacillota	Streptococcus	Streptococcus sp902363395	98
T2103096362	ORT-AF10-25-O-BH	Neisseria_cinerea	97.88	Pseudomonadota	Neisseria	Neisseria flavescens_B	15
T2103096363	ORT-AF10-26-O-BH	Streptococcus_salivarius	99.86	Bacillota	Streptococcus	Streptococcus salivarius	4
T2103096366	ODP-AF10-C3	Abiotrophia_defectiva	99.59	Bacillota	Abiotrophia	Abiotrophia defectiva	37
T2103096369	ODP-AF10-C6	Streptococcus_constellatus	99.12	Bacillota	Streptococcus	Streptococcus constellatus	46
T2103096370	ODP-AF10-C7	Capnocytophaga_ochracea	98.11	Bacteroidota	Capnocytophaga	Capnocytophaga ochracea	190

T2103096374	ODP-AF10-C11	Capnocytophaga_ochracea	97.77	Bacteroidota	Capnocytophaga	Capnocytophaga ochracea	190
T2103096376	ODP-AF10-C13	Capnocytophaga_ochracea	98.34	Bacteroidota	Capnocytophaga	Capnocytophaga ochracea	190
T2103096378	ODP-AF10-C16	Fusobacterium_nucleatum	99.85	Fusobacteriota	Fusobacterium	Fusobacterium polymorphum	87
T2103096379	ODP-AF10-C17	Actinomyces_naeslundii	98.78	Actinomycetota	Actinomyces	Actinomyces naeslundii	33
T2103096381	ODP-AF10-C20	Actinomyces_odontolyticus	99.19	Actinomycetota	Pauljensenia	Pauljensenia odontolytica_A	27
T2103096382	ODP-AF10-C21	Streptococcus_mitis	99.65	Bacillota	Streptococcus	Streptococcus sp900555155	107
T2103096383	ODP-AF10-C22	Actinomyces_naeslundii	99.29	Actinomycetota	Actinomyces	Actinomyces oris	39
T2103096384	ODP-AF10-C23	Streptococcus_pseudopneumoniae	99.59	Bacillota	Streptococcus	Streptococcus mitis_BB	188
T2103096385	ORT-AF10-C1	Streptococcus_pseudopneumoniae	99.72	Bacillota	Streptococcus	Streptococcus symci	157
T2103096389	ORT-AF10-C6	#N/A	#N/A	Bacillota	Bacillus_A	Bacillus_A tropicus	191
T2103096391	ORT-AF10-C8	Actinomyces_naeslundii	98.32	Actinomycetota	Actinomyces	Actinomyces naeslundii	33
T2103096393	ORT-AF10-C11	Streptococcus_oralis	99.66	Bacillota	Streptococcus	Streptococcus sp900550895	186
T2103096394	ORT-AF10-C12	Streptococcus_oralis	99.66	Bacillota	Streptococcus	Streptococcus sp900550895	186
T2103096395	ORT-AF10-C13	Streptococcus_rubneri	97.04	Bacillota	Streptococcus	0	68
T2103096397	ORT-AF10-C15	#N/A	#N/A	Bacillota	Bacillus_A	Bacillus_A tropicus	191
T2103096401	ORS-AF10-C5	Actinomyces_naeslundii	88.56	Actinomycetota	Actinomyces	Actinomyces oris	39
T2103096403	ORS-AF10-C8	Streptococcus_pseudopneumoniae	99.52	Bacillota	Streptococcus	Streptococcus mitis_BB	188
T2103096404	ORS-AF10-C9	Actinomyces_naeslundii	98.31	Actinomycetota	Actinomyces	Actinomyces oris	39
T2103096405	ORS-AF10-C11	#N/A	#N/A	Bacteroidota	Capnocytophaga	Capnocytophaga ochracea	190
T2103096406	ORS-AF10-C12	Streptococcus_oralis	99.38	Bacillota	Streptococcus	0	192
T2103096407	ORS-AF10-C13	Streptococcus_oralis	99.38	Bacillota	Streptococcus	0	192
T2103096409	ORS-AF10-C15	Streptococcus_infantis	99.25	Bacillota	Streptococcus	0	193
T2103096412	ORS-AF10-C18	Streptococcus_parasanguinis	98.84	Bacillota	Streptococcus	Streptococcus sp900766505	6
T2103096413	ORS-AF10-C19	Streptococcus_mitis	99.59	Bacillota	Streptococcus	0	194
T2103096414	ORS-AF10-C20	Streptococcus_mitis	99.65	Bacillota	Streptococcus	0	194
T2103096415	ORS-AF10-1-BH	Streptococcus_parasanguinis	99.04	Bacillota	Streptococcus	Streptococcus parasanguinis_C	53
T2103096416	ORS-AF10-2-BH	Streptococcus_parasanguinis	98.97	Bacillota	Streptococcus	Streptococcus parasanguinis_C	53
T2103096417	ORS-AF10-3-BH	Streptococcus_oralis	99.11	Bacillota	Streptococcus	Streptococcus sp013394695	184
T2103096419	ORS-AF10-5-BH	Streptococcus_pseudopneumoniae	99.72	Bacillota	Streptococcus	Streptococcus symci	157
T2103096421	ORS-AF10-7-BH	Streptococcus_mitis	99.65	Bacillota	Streptococcus	Streptococcus oralis	107
T2103096422	ORS-AF10-8-BH	Streptococcus_pseudopneumoniae	99.72	Bacillota	Streptococcus	Streptococcus symci	157
T2103096423	ORS-AF10-9-BH	Streptococcus_mitis	99.31	Bacillota	Streptococcus	Streptococcus mitis_AC	170
T2103096424	ORS-AF10-10-BH	Streptococcus_mitis	99.65	Bacillota	Streptococcus	0	194
T2103096425	ORS-AF10-11-BH	Streptococcus_mitis	99.65	Bacillota	Streptococcus	0	194
T2103096426	ORS-AF10-12-BH	Granulicatella_adiacens	99.32	Bacillota	Granulicatella	Granulicatella sp001058355	95
T2103096427	ORS-AF10-13-BH	Streptococcus_pseudopneumoniae	99.72	Bacillota	Streptococcus	Streptococcus symci	157
T2103096428	ORS-AF10-14-BH	Granulicatella_adiacens	99.32	Bacillota	Granulicatella	Granulicatella sp001058355	95
T2103096436	ORS-AF10-22-BH	Streptococcus_oralis	99.66	Bacillota	Streptococcus	Streptococcus sp900550895	186
T2103096438	ORS-AF10-24-BH	Granulicatella_adiacens	99.72	Bacillota	Granulicatella	Granulicatella sp001071995	195
T2103096330	ODP-AF10-20-O-BH	Rothia_aeria	99.86	Actinomycetota	Rothia	Rothia aeria	32
T2103096333	ODP-AF10-25-O-BH	Neisseria_macacae	100.00	Pseudomonadota	Neisseria	Neisseria sicca_B	187
T2103096329	ODP-AF10-19-O-BH	Actinomyces_oris	99.34	Actinomycetota	Actinomyces	Actinomyces oris	39
T2103096337	ODP-AF10-31-O-BH	Enterococcus_faecalis	100.00	Bacillota	Enterococcus	Enterococcus faecalis	21
T2103096336	ODP-AF10-30-O-BH	Actinomyces_naeslundii	97.37	Actinomycetota	Actinomyces	Actinomyces oris	39
T2103096328	ODP-AF10-18-O-BH	Neisseria_cinerea	97.88	Pseudomonadota	Neisseria	Neisseria flavescens_B	15
T2103096346	ORT-AF10-07-O-BH	Streptococcus_mitis	99.65	Bacillota	Streptococcus	0	189
T2103096339	ODP-AF10-33-O-BH	Actinomyces_naeslundii	99.59	Actinomycetota	Actinomyces	Actinomyces naeslundii	33
T2103096344	ORT-AF10-05-O-BH	Streptococcus_pseudopneumoniae	99.52	Bacillota	Streptococcus	Streptococcus mitis_BB	188
T2103096332	ODP-AF10-23-O-BH	Rothia_dentocariosa	99.93	Actinomycetota	Rothia	Rothia dentocariosa	13
T2103096331	ODP-AF10-21-O-BH	Rothia_dentocariosa	100.00	Actinomycetota	Rothia	Rothia dentocariosa	13
T2103096347	ORT-AF10-08-O-BH	Streptococcus_parasanguinis	99.76	Bacillota	Streptococcus	Streptococcus parasanguinis	20
T2102085873	ORS-AF08-6	Streptococcus_salivarius	99.86	Bacillota	Streptococcus	Streptococcus salivarius	4
T2102085868	ORS-AF08-1	Streptococcus_timonensis	97.75	Bacillota	Streptococcus	0	176
T2102085869	ORS-AF08-2	Streptococcus_salivarius	99.86	Bacillota	Streptococcus	Streptococcus salivarius	4
T2102085875	ORS-AF08-8	Streptococcus_oralis	99.18	Bacillota	Streptococcus	0	154
T2102085876	ORS-AF08-9	Streptococcus_salivarius	99.86	Bacillota	Streptococcus	Streptococcus salivarius	4
T2102085872	ORS-AF08-5	Streptococcus_rubneri	91.91	Bacillota	Streptococcus	Streptococcus koreensis	68
T2102085931	ORS-AF08-10	Streptococcus_mitis	99.65	Bacillota	Streptococcus	Streptococcus sp900555155	107
T2102085870	ORS-AF08-3	Streptococcus_infantis	99.52	Bacillota	Streptococcus	0	154
T2102085936	ORS-AF08-15	Streptococcus_salivarius	100.00	Bacillota	Streptococcus	Streptococcus salivarius	4
T2102085937	ORS-AF08-16	Streptococcus_sanguinis	100.00	Bacillota	Streptococcus	Streptococcus sanguinis_H	3
T2102085935	ORS-AF08-14	Streptococcus_mitis	99.65	Bacillota	Streptococcus	Streptococcus oralis	107
T2102085940	ORS-AF08-19	Streptococcus_mitis	99.52	Bacillota	Streptococcus	Streptococcus oralis	107
T2102085938	ORS-AF08-17	Streptococcus_mitis	99.52	Bacillota	Streptococcus	Streptococcus oralis	107
T2102085932	ORS-AF08-11	Streptococcus_timonensis	99.04	Bacillota	Streptococcus	0	127
T2102085939	ORS-AF08-18	Streptococcus_constellatus	99.05	Bacillota	Streptococcus	Streptococcus constellatus	46
T2102085934	ORS-AF08-13	Streptococcus_mitis	86.48	Bacillota	Streptococcus	Streptococcus oralis	107

Supplementary Table 3. The functional annotation of the COGR genomes (BGC with known function)

Genome	Cluster	Reference BGC	Product	Identity (%)
ORS-AF05-12	4	https://mibig.secondarymetabolites.org/go/BGC0001209/1	streptide	1
ORT-AM08-194	4	https://mibig.secondarymetabolites.org/go/BGC0001929/1	WGK	0.8
ORS-AF04-41	3	https://mibig.secondarymetabolites.org/go/BGC0000556/1	streptin	0.9
ORS-AF05-14	4	https://mibig.secondarymetabolites.org/go/BGC0001209/1	streptide	1
ORS-AF05-16	4	https://mibig.secondarymetabolites.org/go/BGC0001209/1	streptide	1
ORS-AF06-69	61	https://mibig.secondarymetabolites.org/go/BGC0000566/1	streptolysin S	0.77
ORT-AM08-200	4	https://mibig.secondarymetabolites.org/go/BGC0001929/1	WGK	1
ORS-AM04-86	3	https://mibig.secondarymetabolites.org/go/BGC0000556/1	streptin	0.9
ORS-AM05-342	99	https://mibig.secondarymetabolites.org/go/BGC0000944/1	staphyloferrin A	0.75
ORS-AM08-15	4	https://mibig.secondarymetabolites.org/go/BGC0001929/1	WGK	0.8
ODP-AF05-77	4	https://mibig.secondarymetabolites.org/go/BGC0001209/1	streptide	1
ORT-AF03-21	4	https://mibig.secondarymetabolites.org/go/BGC0001788/1	suicin 65	1
ORT-AF03-21	4	https://mibig.secondarymetabolites.org/go/BGC0001209/1	streptide	1
ORT-AM08-202	4	https://mibig.secondarymetabolites.org/go/BGC0001929/1	WGK	0.8
ODP-AF04-91	4	https://mibig.secondarymetabolites.org/go/BGC0001209/1	streptide	1
ORT-AF03-24	117	https://mibig.secondarymetabolites.org/go/BGC0000548/1	salivaricin A	0.87
ORT-AF03-24	117	https://mibig.secondarymetabolites.org/go/BGC0001788/1	suicin 65	1
ORT-AF04-19	4	https://mibig.secondarymetabolites.org/go/BGC0000556/1	streptin	0.9
ORT-AF04-19	4	https://mibig.secondarymetabolites.org/go/BGC0001788/1	suicin 65	1
ORS-AF04-236	3	https://mibig.secondarymetabolites.org/go/BGC0000556/1	streptin	0.9
ORS-AM05-336	99	https://mibig.secondarymetabolites.org/go/BGC0000944/1	staphyloferrin A	0.75
ODP-AF04-99	4	https://mibig.secondarymetabolites.org/go/BGC0001209/1	streptide	1
ORT-AM01-01TBO	137	https://mibig.secondarymetabolites.org/go/BGC0000942/1	petrobactin	1
ORS-AF03-153	117	https://mibig.secondarymetabolites.org/go/BGC0000548/1	salivaricin A	0.87
ORS-AF03-153	117	https://mibig.secondarymetabolites.org/go/BGC0001788/1	suicin 65	1
ORS-AF03-155	141	https://mibig.secondarymetabolites.org/go/BGC0001929/1	WGK	0.8
ODP-AF04-103	4	https://mibig.secondarymetabolites.org/go/BGC0001209/1	streptide	1
ODP-AF04-166	3	https://mibig.secondarymetabolites.org/go/BGC0000556/1	streptin	0.9
ORT-AF04-29	4	https://mibig.secondarymetabolites.org/go/BGC0000556/1	streptin	0.9
ORT-AF04-29	4	https://mibig.secondarymetabolites.org/go/BGC0001788/1	suicin 65	0.9
ORT-AF04-121	4	https://mibig.secondarymetabolites.org/go/BGC0001209/1	streptide	1
ORT-AF04-228	4	https://mibig.secondarymetabolites.org/go/BGC0000556/1	streptin	0.9
ORT-AF04-228	4	https://mibig.secondarymetabolites.org/go/BGC0001788/1	suicin 65	0.9
ORS-AM09-33-O-BH	4	https://mibig.secondarymetabolites.org/go/BGC0001788/1	suicin 65	1
ODP-AM09-19-O-BH	164	https://mibig.secondarymetabolites.org/go/BGC0001356/1	paeninodin	1
ODP-AM09-19-O-BH	164	https://mibig.secondarymetabolites.org/go/BGC0000942/1	petrobactin	1
ODP-AM09-29-O-BH	3	https://mibig.secondarymetabolites.org/go/BGC0000556/1	streptin	0.7
ORS-AM09-20-BH	4	https://mibig.secondarymetabolites.org/go/BGC0001788/1	suicin 65	1
ORS-AM09-20-BH	4	https://mibig.secondarymetabolites.org/go/BGC0001209/1	streptide	1
ORS-AM09-30-BH	4	https://mibig.secondarymetabolites.org/go/BGC0001788/1	suicin 65	1
ORS-AM09-30-BH	4	https://mibig.secondarymetabolites.org/go/BGC0001209/1	streptide	1
ORS-AM09-8-O-104	4	https://mibig.secondarymetabolites.org/go/BGC0001788/1	suicin 65	1
ORS-AM09-16-O-104	4	https://mibig.secondarymetabolites.org/go/BGC0001788/1	suicin 65	1
ORS-AF08-2-MRS	4	https://mibig.secondarymetabolites.org/go/BGC0001788/1	suicin 65	1
ORS-AF08-5-MRS	4	https://mibig.secondarymetabolites.org/go/BGC0001788/1	suicin 65	1
ORS-AF08-23-MRS	4	https://mibig.secondarymetabolites.org/go/BGC0001788/1	suicin 65	1
ORS-AF08-30-MRS	4	https://mibig.secondarymetabolites.org/go/BGC0001788/1	suicin 65	1
ODP-AM09-2D1	4	https://mibig.secondarymetabolites.org/go/BGC0001788/1	suicin 65	1
ODP-AM09-2D1	4	https://mibig.secondarymetabolites.org/go/BGC0001209/1	streptide	1
ODP-AM09-2D2	4	https://mibig.secondarymetabolites.org/go/BGC0001788/1	suicin 65	1
ODP-AM09-2D2	4	https://mibig.secondarymetabolites.org/go/BGC0001209/1	streptide	1
ODP-AM09-2D4A	4	https://mibig.secondarymetabolites.org/go/BGC0001788/1	suicin 65	1
ODP-AM09-2D4A	4	https://mibig.secondarymetabolites.org/go/BGC0001209/1	streptide	1
ODP-AM09-2D4B	4	https://mibig.secondarymetabolites.org/go/BGC0001209/1	streptide	1
ODP-AM09-2D4B	4	https://mibig.secondarymetabolites.org/go/BGC0001788/1	suicin 65	1
ODP-AM09-2D5	4	https://mibig.secondarymetabolites.org/go/BGC0001788/1	suicin 65	1

ODP-AM09-2D16	4	https://mibig.secondarymetabolites.org/go/BGC0001788/1	suicin 65	1
ODP-AM09-2D16	4	https://mibig.secondarymetabolites.org/go/BGC0001209/1	streptide	1
ODP-AM09-2D20	4	https://mibig.secondarymetabolites.org/go/BGC0001788/1	suicin 65	1
ODP-AF08-18-O-MRS	4	https://mibig.secondarymetabolites.org/go/BGC0001788/1	suicin 65	1
ORT-AF08-12-O-MRS	4	https://mibig.secondarymetabolites.org/go/BGC0001788/1	suicin 65	1
ORS-AM09-12-104	4	https://mibig.secondarymetabolites.org/go/BGC0001788/1	suicin 65	1
ORS-AM09-12-104	4	https://mibig.secondarymetabolites.org/go/BGC0001929/1	WGK	0.8
ORT-AF10-10-O-BH	4	https://mibig.secondarymetabolites.org/go/BGC0001209/1	streptide	1
ORT-AF10-26-O-BH	4	https://mibig.secondarymetabolites.org/go/BGC0001929/1	WGK	0.8
ORT-AF10-C6	191	https://mibig.secondarymetabolites.org/go/BGC0000942/1	petrobactin	1
ORT-AF10-C6	191	https://mibig.secondarymetabolites.org/go/BGC0001356/1	paeninodin	1
ORT-AF10-C15	191	https://mibig.secondarymetabolites.org/go/BGC0001356/1	paeninodin	1
ORT-AF10-C15	191	https://mibig.secondarymetabolites.org/go/BGC0000942/1	petrobactin	1

Supplementary Table 3. The functional annotation of the COGR genomes (VFDB)

Genome	Cluster	VF_name	VF_ID	Vf_category
ODP-AF03-102	107	PI-2	VF0530	Adherence
ODP-AF03-102	107	Capsule	VF0144	Immune modulation
ODP-AF03-102	107	Neuraminidase	VF0148	Exoenzyme
ODP-AF03-106	21	Capsule	VF0361	Immune modulation
ODP-AF03-106	21	EfaA	VF0354	Adherence
ODP-AF03-106	21	Ebp pili	VF0538	Adherence
ODP-AF03-106	21	Hyaluronidase	VF0359	Exoenzyme
ODP-AF03-106	21	ClpP	VF0074	Stress survival
ODP-AF03-248	145	ClpP	VF0074	Stress survival
ODP-AF03-248	145	BslA	VF0411	Regulation
ODP-AF03-248	145	ClpC	VF0072	Stress survival
ODP-AF04-105	49	IgA1 protease	VF0147	Immune modulation
ODP-AF04-105	49	ClpP	VF0074	Stress survival
ODP-AF04-105	49	PsaA	VF0151	Nutritional/Metabolic factor
ODP-AF04-114	43	ClpP	VF0074	Stress survival
ODP-AF04-151	23	ClpP	VF0074	Stress survival
ODP-AF04-151	23	GroEL	VF0594	Adherence
ODP-AF04-152	23	ClpP	VF0074	Stress survival
ODP-AF04-152	23	GroEL	VF0594	Adherence
ODP-AF04-155	23	ClpP	VF0074	Stress survival
ODP-AF04-155	23	GroEL	VF0594	Adherence
ODP-AF04-158	23	ClpP	VF0074	Stress survival
ODP-AF04-158	23	GroEL	VF0594	Adherence
ODP-AF04-161	23	ClpP	VF0074	Stress survival
ODP-AF04-161	23	GroEL	VF0594	Adherence
ODP-AF04-166	3	PavB	VF0524	Adherence
ODP-AF04-244	23	ClpP	VF0074	Stress survival
ODP-AF04-244	23	GroEL	VF0594	Adherence
ODP-AF04-83	8	Capsule	VF0144	Immune modulation
ODP-AF04-83	8	Capsule	VF0361	Immune modulation
ODP-AF04-83	8	Neuraminidase	VF0148	Exoenzyme
ODP-AF04-83	8	IgA1 protease	VF0147	Immune modulation
ODP-AF04-83	8	PfbA	VF0525	Adherence
ODP-AF04-88	9	Capsule	VF0144	Immune modulation
ODP-AF04-88	9	PfbA	VF0525	Adherence
ODP-AF04-88	9	CBPs	VF0145	Adherence
ODP-AF04-88	9	Capsule	VF0361	Immune modulation
ODP-AF04-94	9	Capsule	VF0144	Immune modulation
ODP-AF04-94	9	PfbA	VF0525	Adherence
ODP-AF04-94	9	Capsule	VF0361	Immune modulation
ODP-AF04-94	9	CBPs	VF0145	Adherence
ODP-AF05-203	61	Capsule	VF0144	Immune modulation
ODP-AF05-203	61	SpaP	VF0526	Adherence
ODP-AF06-103	23	GroEL	VF0594	Adherence
ODP-AF06-103	23	ClpP	VF0074	Stress survival
ODP-AF06-107	154	PavB	VF0524	Adherence
ODP-AF06-108	93	PI-2	VF0530	Adherence
ODP-AF06-108	93	Capsule	VF0144	Immune modulation
ODP-AF06-108	93	PavB	VF0524	Adherence
ODP-AF06-108	93	PfbA	VF0525	Adherence
ODP-AF06-99	101	Capsule	VF0144	Immune modulation
ODP-AF06-99	101	Capsule	VF0361	Immune modulation
ODP-AF06-99	101	CBPs	VF0145	Adherence
ODP-AF08-10-MRS	107	Capsule	VF0144	Immune modulation
ODP-AF08-10-MRS	107	PI-2	VF0530	Adherence
ODP-AF08-10-MRS	107	PavB	VF0524	Adherence
ODP-AF08-11-O-MRS	61	Capsule	VF0144	Immune modulation
ODP-AF08-13-MRS	46	Capsule	VF0144	Immune modulation
ODP-AF08-16-MRS	46	Capsule	VF0144	Immune modulation
ODP-AF08-17-MRS	113	PavB	VF0524	Adherence
ODP-AF08-18-O-MRS	4	PavB	VF0524	Adherence
ODP-AF08-20-MRS	46	Capsule	VF0144	Immune modulation
ODP-AF08-20	21	Capsule	VF0361	Immune modulation
ODP-AF08-20	21	EfaA	VF0354	Adherence
ODP-AF08-20	21	ClpP	VF0074	Stress survival
ODP-AF08-21-MRS	107	Capsule	VF0144	Immune modulation
ODP-AF08-21-MRS	107	PavB	VF0524	Adherence
ODP-AF08-21-MRS	107	PI-2	VF0530	Adherence
ODP-AF08-22-MRS	177	ClpP	VF0074	Stress survival
ODP-AF08-23-MRS	107	Capsule	VF0144	Immune modulation
ODP-AF08-23-MRS	107	PI-2	VF0530	Adherence
ODP-AF08-23-MRS	107	PavB	VF0524	Adherence
ODP-AF08-25-O-MRS	61	Capsule	VF0144	Immune modulation
ODP-AF08-27-O-MRS	61	Capsule	VF0144	Immune modulation
ODP-AF08-28-MRS	23	GroEL	VF0594	Adherence
ODP-AF08-28-MRS	23	Autolysin	VF0143	Exoenzyme
ODP-AF08-28-MRS	23	ClpP	VF0074	Stress survival
ODP-AF08-2-MRS	104	SpaP	VF0526	Adherence
ODP-AF08-2	107	PavB	VF0524	Adherence
ODP-AF08-2	107	Capsule	VF0144	Immune modulation
ODP-AF08-2	107	PI-2	VF0530	Adherence
ODP-AF08-35-MRS	107	Capsule	VF0144	Immune modulation
ODP-AF08-35-MRS	107	PI-2	VF0530	Adherence
ODP-AF08-35-MRS	107	PavB	VF0524	Adherence
ODP-AF08-36-MRS	107	Capsule	VF0144	Immune modulation
ODP-AF08-36-MRS	107	PI-2	VF0530	Adherence
ODP-AF08-36-MRS	107	PavB	VF0524	Adherence
ODP-AF08-37-MRS	23	GroEL	VF0594	Adherence
ODP-AF08-37-MRS	23	ClpP	VF0074	Stress survival
ODP-AF08-38-MRS	11	PI-2	VF0530	Adherence
ODP-AF08-38-MRS	11	Capsule	VF0144	Immune modulation
ODP-AF08-38-MRS	11	Neuraminidase	VF0148	Exoenzyme
ODP-AF08-38-MRS	11	PavB	VF0524	Adherence
ODP-AF08-39-MRS	170	Capsule	VF0144	Immune modulation
ODP-AF08-39-MRS	170	CBPs	VF0145	Adherence
ODP-AF08-39-MRS	170	PfbA	VF0525	Adherence
ODP-AF08-3	46	PavB	VF0524	Adherence
ODP-AF08-3	46	Capsule	VF0144	Immune modulation
ODP-AF08-40-MRS	46	Capsule	VF0144	Immune modulation
ODP-AF08-41-MRS	107	Capsule	VF0144	Immune modulation
ODP-AF08-41-MRS	107	PavB	VF0524	Adherence
ODP-AF08-41-MRS	107	PI-2	VF0530	Adherence
ODP-AF08-42-MRS	170	PfbA	VF0525	Adherence
ODP-AF08-42-MRS	170	CBPs	VF0145	Adherence
ODP-AF08-42-MRS	170	Capsule	VF0144	Immune modulation
ODP-AF08-7-MRS	107	Capsule	VF0144	Immune modulation
ODP-AF08-7-MRS	107	PI-2	VF0530	Adherence
ODP-AF08-7-MRS	107	PavB	VF0524	Adherence
ODP-AF08-8-MRS	107	Capsule	VF0144	Immune modulation
ODP-AF08-8-MRS	107	PI-2	VF0530	Adherence
ODP-AF08-8-MRS	107	PavB	VF0524	Adherence
ODP-AF10-17-O-BH	187	Polar flagella	VF0473	Motility
ODP-AF10-17-O-BH	187	MntABC	VF0455	Stress survival
ODP-AF10-17-O-BH	187	Type IV pili	VF0075	Adherence
ODP-AF10-18-O-BH	15	Polar flagella	VF0473	Motility
ODP-AF10-18-O-BH	15	Capsule	VF0079	Immune modulation
ODP-AF10-18-O-BH	15	MntABC	VF0455	Stress survival
ODP-AF10-18-O-BH	15	HmbR	VF0048	Nutritional/Metabolic factor
ODP-AF10-18-O-BH	15	MtrCDE	VF0451	ntimicrobial activity/Competitive advantage

ODP-AF10-18-O-BH	15	Type IV pili	VF0075	Adherence
ODP-AF10-25-O-BH	187	Polar flagella	VF0473	Motility
ODP-AF10-25-O-BH	187	MntABC	VF0455	Stress survival
ODP-AF10-25-O-BH	187	Type IV pili	VF0075	Adherence
ODP-AF10-31-O-BH	21	SprE	VF0358	Exoenzyme
ODP-AF10-31-O-BH	21	Gelatinase	VF0357	Exoenzyme
ODP-AF10-31-O-BH	21	Fsr	VF0360	Biofilm
ODP-AF10-31-O-BH	21	EfaA	VF0354	Adherence
ODP-AF10-31-O-BH	21	ClpP	VF0074	Stress survival
ODP-AF10-31-O-BH	21	Capsule	VF0361	Immune modulation
ODP-AF10-C21	107	PfbA	VF0525	Adherence
ODP-AF10-C21	107	PI-2	VF0530	Adherence
ODP-AF10-C21	107	Capsule	VF0144	Immune modulation
ODP-AF10-C21	107	Neuraminidase	VF0148	Exoenzyme
ODP-AF10-C21	107	PavB	VF0524	Adherence
ODP-AF10-C23	188	Capsule	VF0144	Immune modulation
ODP-AF10-C23	188	Neuraminidase	VF0148	Exoenzyme
ODP-AF10-C23	188	PfbA	VF0525	Adherence
ODP-AF10-C23	188	PavB	VF0524	Adherence
ODP-AF10-C3	37	ClpP	VF0074	Stress survival
ODP-AF10-C6	46	Capsule	VF0144	Immune modulation
ODP-AM01-02BB	107	PI-2	VF0530	Adherence
ODP-AM01-02BB	107	Capsule	VF0144	Immune modulation
ODP-AM01-02BB	107	Neuraminidase	VF0148	Exoenzyme
ODP-AM01-02O	15	Capsule	VF0079	Immune modulation
ODP-AM01-02O	15	MntABC	VF0455	Stress survival
ODP-AM01-05	65	ClpP	VF0074	Stress survival
ODP-AM01-06	133	K1 capsule	VF0239	Invasion
ODP-AM01-08	107	PI-2	VF0530	Adherence
ODP-AM01-08	107	Capsule	VF0144	Immune modulation
ODP-AM01-08	107	Neuraminidase	VF0148	Exoenzyme
ODP-AM01-10	134	ClpP	VF0074	Stress survival
ODP-AM01-10	134	LPS	VF0367	Immune modulation
ODP-AM01-10	134	Capsule	VF0144	Immune modulation
ODP-AM04-107	4	PavB	VF0524	Adherence
ODP-AM04-126	15	MtrCDE	VF0451	ntimicrobial activity/Competitive advantage
ODP-AM04-126	15	Capsule	VF0079	Immune modulation
ODP-AM04-126	15	MntABC	VF0455	Stress survival
ODP-AM04-45	124	PavB	VF0524	Adherence
ODP-AM04-46	4	PavB	VF0524	Adherence
ODP-AM04-51	151	ClpP	VF0074	Stress survival
ODP-AM04-51	151	PsaA	VF0151	Nutritional/Metabolic factor
ODP-AM04-52	34	GroEL	VF0594	Adherence
ODP-AM04-52	34	ClpP	VF0074	Stress survival
ODP-AM05-19	31	Capsule	VF0144	Immune modulation
ODP-AM05-19	31	PavB	VF0524	Adherence
ODP-AM05-22	23	ClpP	VF0074	Stress survival
ODP-AM05-244	61	SpaP	VF0526	Adherence
ODP-AM05-244	61	Capsule	VF0144	Immune modulation
ODP-AM05-248	61	SpaP	VF0526	Adherence
ODP-AM05-248	61	Capsule	VF0144	Immune modulation
ODP-AM05-249	23	ClpP	VF0074	Stress survival
ODP-AM05-249	23	GroEL	VF0594	Adherence
ODP-AM05-24	63	PavB	VF0524	Adherence
ODP-AM05-252	101	Capsule	VF0144	Immune modulation
ODP-AM05-252	101	PfbA	VF0525	Adherence
ODP-AM05-252	101	CBPs	VF0145	Adherence
ODP-AM05-252	101	PavB	VF0524	Adherence
ODP-AM05-252	101	Capsule	VF0361	Immune modulation
ODP-AM05-255	30	GroEL	VF0594	Adherence
ODP-AM05-255	30	ClpP	VF0074	Stress survival
ODP-AM05-25	11	Capsule	VF0144	Immune modulation
ODP-AM05-25	11	Neuraminidase	VF0148	Exoenzyme
ODP-AM05-25	11	PavB	VF0524	Adherence
ODP-AM05-262	46	Capsule	VF0144	Immune modulation
ODP-AM05-263	35	Capsule	VF0144	Immune modulation
ODP-AM05-263	35	Neuraminidase	VF0148	Exoenzyme
ODP-AM05-263	35	PavB	VF0524	Adherence
ODP-AM05-264	11	Capsule	VF0144	Immune modulation
ODP-AM05-264	11	Neuraminidase	VF0148	Exoenzyme
ODP-AM05-264	11	PavB	VF0524	Adherence
ODP-AM05-265	61	SpaP	VF0526	Adherence
ODP-AM05-265	61	Capsule	VF0144	Immune modulation
ODP-AM05-266	46	Capsule	VF0144	Immune modulation
ODP-AM05-267	11	Capsule	VF0144	Immune modulation
ODP-AM05-267	11	Neuraminidase	VF0148	Exoenzyme
ODP-AM05-267	11	PavB	VF0524	Adherence
ODP-AM05-271	34	GroEL	VF0594	Adherence
ODP-AM05-271	34	ClpP	VF0074	Stress survival
ODP-AM05-272	30	GroEL	VF0594	Adherence
ODP-AM05-272	30	ClpP	VF0074	Stress survival
ODP-AM05-273	61	SpaP	VF0526	Adherence
ODP-AM05-273	61	Capsule	VF0144	Immune modulation
ODP-AM05-303	44	Polar flagella	VF0473	Motility
ODP-AM05-303	44	MtrCDE	VF0451	ntimicrobial activity/Competitive advantage
ODP-AM05-303	44	MntABC	VF0455	Stress survival
ODP-AM05-303	44	Type IV pili	VF0075	Adherence
ODP-AM05-306	64	HmbR	VF0048	Nutritional/Metabolic factor
ODP-AM05-306	64	Capsule	VF0079	Immune modulation
ODP-AM05-306	64	MntABC	VF0455	Stress survival
ODP-AM05-321	15	MtrCDE	VF0451	ntimicrobial activity/Competitive advantage
ODP-AM05-321	15	MntABC	VF0455	Stress survival
ODP-AM05-321	15	Polar flagella	VF0473	Motility
ODP-AM05-34	61	SpaP	VF0526	Adherence
ODP-AM05-34	61	Capsule	VF0144	Immune modulation
ODP-AM05-388	65	ClpP	VF0074	Stress survival
ODP-AM05-395	46	Capsule	VF0144	Immune modulation
ODP-AM05-41	61	SpaP	VF0526	Adherence
ODP-AM05-41	61	Capsule	VF0144	Immune modulation
ODP-AM05-440	125	WhiB3	VF0288	Regulation
ODP-AM05-456	88	Capsule	VF0144	Immune modulation
ODP-AM05-456	88	ClpP	VF0074	Stress survival
ODP-AM05-456	88	Capsule I	VF0436	Immune modulation
ODP-AM05-457	89	Neuraminidase	VF0148	Exoenzyme
ODP-AM05-457	89	Capsule	VF0144	Immune modulation
ODP-AM05-458	89	Neuraminidase	VF0148	Exoenzyme
ODP-AM05-458	89	Capsule	VF0144	Immune modulation
ODP-AM05-46	15	MntABC	VF0455	Stress survival
ODP-AM05-46	15	MtrCDE	VF0451	ntimicrobial activity/Competitive advantage
ODP-AM05-46	15	Polar flagella	VF0473	Motility
ODP-AM05-49	11	Capsule	VF0144	Immune modulation
ODP-AM05-49	11	Neuraminidase	VF0148	Exoenzyme
ODP-AM05-49	11	PavB	VF0524	Adherence
ODP-AM05-503	63	PavB	VF0524	Adherence
ODP-AM05-504	116	CBPs	VF0145	Adherence
ODP-AM05-504	116	Capsule	VF0144	Immune modulation
ODP-AM05-504	116	Neuraminidase	VF0148	Exoenzyme
ODP-AM05-506	89	Neuraminidase	VF0148	Exoenzyme
ODP-AM05-506	89	Capsule	VF0144	Immune modulation
ODP-AM05-535	11	Capsule	VF0144	Immune modulation
ODP-AM05-535	11	Neuraminidase	VF0148	Exoenzyme

ODP-AM05-535	11	PavB	VF0524	Adherence
ODP-AM05-73	63	PavB	VF0524	Adherence
ODP-AM05-78	63	PavB	VF0524	Adherence
ODP-AM05-80	31	Capsule	VF0144	Immune modulation
ODP-AM05-80	31	PavB	VF0524	Adherence
ODP-AM08-275	4	PavB	VF0524	Adherence
ODP-AM08-280	25	GroEL	VF0594	Adherence
ODP-AM08-280	25	ClpP	VF0074	Stress survival
ODP-AM08-282	37	ClpP	VF0074	Stress survival
ODP-AM08-289	69	GroEL	VF0594	Adherence
ODP-AM08-289	69	ClpP	VF0074	Stress survival
ODP-AM08-297	37	ClpP	VF0074	Stress survival
ODP-AM08-304	17	ClpP	VF0074	Stress survival
ODP-AM08-308	63	PavB	VF0524	Adherence
ODP-AM08-309	36	PavB	VF0524	Adherence
ODP-AM08-310	23	ClpP	VF0074	Stress survival
ODP-AM08-316	37	ClpP	VF0074	Stress survival
ODP-AM08-325	23	ClpP	VF0074	Stress survival
ODP-AM08-335	23	ClpP	VF0074	Stress survival
ODP-AM08-337	17	ClpP	VF0074	Stress survival
ODP-AM08-337	17	PavB	VF0524	Adherence
ODP-AM08-393	65	ClpP	VF0074	Stress survival
ODP-AM08-396	37	ClpP	VF0074	Stress survival
ODP-AM08-398	108	Autolysin	VF0143	Exoenzyme
ODP-AM08-398	108	Capsule	VF0144	Immune modulation
ODP-AM08-398	108	Neuraminidase	VF0148	Exoenzyme
ODP-AM08-398	108	PavB	VF0524	Adherence
ODP-AM08-403	23	ClpP	VF0074	Stress survival
ODP-AM09-10-104	157	Capsule	VF0144	Immune modulation
ODP-AM09-10-104	157	PfbA	VF0525	Adherence
ODP-AM09-10-104	157	Neuraminidase	VF0148	Exoenzyme
ODP-AM09-10-O-104	107	PI-2	VF0530	Adherence
ODP-AM09-10-O-104	107	Capsule	VF0144	Immune modulation
ODP-AM09-10-O-104	107	PavB	VF0524	Adherence
ODP-AM09-10-O-BH	159	Neuraminidase	VF0148	Exoenzyme
ODP-AM09-11-104	104	SpaP	VF0526	Adherence
ODP-AM09-11-BH	63	PavB	VF0524	Adherence
ODP-AM09-1-1-O-BH	162	Neuraminidase	VF0148	Exoenzyme
ODP-AM09-1-1-O-BH	162	Capsule	VF0144	Immune modulation
ODP-AM09-1-1-O-BH	162	PavB	VF0524	Adherence
ODP-AM09-12-104	158	PfbA	VF0525	Adherence
ODP-AM09-12-104	158	Neuraminidase	VF0148	Exoenzyme
ODP-AM09-12-O-104	46	Capsule	VF0144	Immune modulation
ODP-AM09-1-2-O-BH	162	Neuraminidase	VF0148	Exoenzyme
ODP-AM09-1-2-O-BH	162	Capsule	VF0144	Immune modulation
ODP-AM09-1-2-O-BH	162	PavB	VF0524	Adherence
ODP-AM09-12-O-BH	157	Capsule	VF0144	Immune modulation
ODP-AM09-12-O-BH	157	Neuraminidase	VF0148	Exoenzyme
ODP-AM09-12-O-BH	157	PfbA	VF0525	Adherence
ODP-AM09-13-104	158	PfbA	VF0525	Adherence
ODP-AM09-13-104	158	Neuraminidase	VF0148	Exoenzyme
ODP-AM09-13-O-BH	63	PavB	VF0524	Adherence
ODP-AM09-14-104	184	Autolysin	VF0143	Exoenzyme
ODP-AM09-14-104	184	Capsule	VF0144	Immune modulation
ODP-AM09-14-BH	168	Capsule	VF0274	Immune modulation
ODP-AM09-14-BH	168	PavB	VF0524	Adherence
ODP-AM09-15-104	157	Capsule	VF0144	Immune modulation
ODP-AM09-15-104	157	Neuraminidase	VF0148	Exoenzyme
ODP-AM09-15-104	157	PfbA	VF0525	Adherence
ODP-AM09-16-O-104	107	Capsule	VF0144	Immune modulation
ODP-AM09-16-O-104	107	PI-2	VF0530	Adherence
ODP-AM09-16-O-104	107	PavB	VF0524	Adherence
ODP-AM09-16-O-BH	163	Neuraminidase	VF0148	Exoenzyme
ODP-AM09-16-O-BH	163	PfbA	VF0525	Adherence
ODP-AM09-16-O-BH	163	CBPs	VF0145	Adherence
ODP-AM09-16-O-BH	163	Capsule	VF0144	Immune modulation
ODP-AM09-16-O-BH	163	Capsule	VF0361	Immune modulation
ODP-AM09-17-104	107	Capsule	VF0144	Immune modulation
ODP-AM09-17-104	107	PI-2	VF0530	Adherence
ODP-AM09-17-104	107	Neuraminidase	VF0148	Exoenzyme
ODP-AM09-18-104	63	PavB	VF0524	Adherence
ODP-AM09-18-O-104	157	Capsule	VF0144	Immune modulation
ODP-AM09-18-O-104	157	Neuraminidase	VF0148	Exoenzyme
ODP-AM09-18-O-104	157	PavB	VF0524	Adherence
ODP-AM09-19-O-104	157	Capsule	VF0144	Immune modulation
ODP-AM09-19-O-104	157	Neuraminidase	VF0148	Exoenzyme
ODP-AM09-19-O-104	157	PavB	VF0524	Adherence
ODP-AM09-19-O-BH	164	Isocitrate lyase	VF0253	Others
ODP-AM09-19-O-BH	164	ClpP	VF0074	Stress survival
ODP-AM09-19-O-BH	164	ClpC	VF0072	Stress survival
ODP-AM09-19-O-BH	164	Petrobactin	VF0584	Nutritional/Metabolic factor
ODP-AM09-1-BH	157	Capsule	VF0144	Immune modulation
ODP-AM09-1-BH	157	Neuraminidase	VF0148	Exoenzyme
ODP-AM09-1-BH	157	PfbA	VF0525	Adherence
ODP-AM09-20-104	158	PfbA	VF0525	Adherence
ODP-AM09-20-O-104	170	PfbA	VF0525	Adherence
ODP-AM09-20-O-104	170	CBPs	VF0145	Adherence
ODP-AM09-20-O-104	170	Capsule	VF0144	Immune modulation
ODP-AM09-20-O-BH	157	Capsule	VF0144	Immune modulation
ODP-AM09-20-O-BH	157	Neuraminidase	VF0148	Exoenzyme
ODP-AM09-20-O-BH	157	PfbA	VF0525	Adherence
ODP-AM09-21-104	158	PfbA	VF0525	Adherence
ODP-AM09-21-O-104	107	PI-2	VF0530	Adherence
ODP-AM09-21-O-104	107	Capsule	VF0144	Immune modulation
ODP-AM09-21-O-104	107	Neuraminidase	VF0148	Exoenzyme
ODP-AM09-21-O-104	107	PavB	VF0524	Adherence
ODP-AM09-22-104	63	PavB	VF0524	Adherence
ODP-AM09-22-BH	157	Capsule	VF0144	Immune modulation
ODP-AM09-22-BH	157	PfbA	VF0525	Adherence
ODP-AM09-22-BH	157	Neuraminidase	VF0148	Exoenzyme
ODP-AM09-22-O-104	107	Neuraminidase	VF0148	Exoenzyme
ODP-AM09-22-O-104	107	PI-2	VF0530	Adherence
ODP-AM09-22-O-104	107	Capsule	VF0144	Immune modulation
ODP-AM09-22-O-104	107	PavB	VF0524	Adherence
ODP-AM09-23-104	163	Neuraminidase	VF0148	Exoenzyme
ODP-AM09-23-104	163	PfbA	VF0525	Adherence
ODP-AM09-23-104	163	CBPs	VF0145	Adherence
ODP-AM09-23-104	163	Capsule	VF0361	Immune modulation
ODP-AM09-23-104	163	Capsule	VF0144	Immune modulation
ODP-AM09-23-104	163	PavB	VF0524	Adherence
ODP-AM09-23-BH	107	PI-2	VF0530	Adherence
ODP-AM09-23-BH	107	Capsule	VF0144	Immune modulation
ODP-AM09-23-BH	107	Neuraminidase	VF0148	Exoenzyme
ODP-AM09-23-O-104	107	Capsule	VF0144	Immune modulation
ODP-AM09-23-O-104	107	PI-2	VF0530	Adherence
ODP-AM09-23-O-104	107	PavB	VF0524	Adherence
ODP-AM09-24-BH	157	Capsule	VF0144	Immune modulation
ODP-AM09-24-BH	157	PfbA	VF0525	Adherence
ODP-AM09-24-BH	157	Neuraminidase	VF0148	Exoenzyme
ODP-AM09-26-104	107	PI-2	VF0530	Adherence
ODP-AM09-26-104	107	Capsule	VF0144	Immune modulation

ODP-AM09-26-104	107	Neuraminidase	VF0148	Exoenzyme
ODP-AM09-27-104	163	Neuraminidase	VF0148	Exoenzyme
ODP-AM09-27-104	163	PfbA	VF0525	Adherence
ODP-AM09-27-104	163	CBPs	VF0145	Adherence
ODP-AM09-27-104	163	Capsule	VF0361	Immune modulation
ODP-AM09-27-104	163	Capsule	VF0144	Immune modulation
ODP-AM09-27-BH	161	Capsule	VF0144	Immune modulation
ODP-AM09-27-BH	161	CBPs	VF0145	Adherence
ODP-AM09-2D18	178	ClpP	VF0074	Stress survival
ODP-AM09-2D21	178	ClpP	VF0074	Stress survival
ODP-AM09-2D3B	178	ClpP	VF0074	Stress survival
ODP-AM09-2D6	21	Fsr	VF0360	Biofilm
ODP-AM09-2D6	21	Gelatinase	VF0357	Exoenzyme
ODP-AM09-2D6	21	SprE	VF0358	Exoenzyme
ODP-AM09-2D6	21	ClpP	VF0074	Stress survival
ODP-AM09-2D6	21	EfaA	VF0354	Adherence
ODP-AM09-2D6	21	Capsule	VF0361	Immune modulation
ODP-AM09-2-O-104	170	PfbA	VF0525	Adherence
ODP-AM09-2-O-104	170	CBPs	VF0145	Adherence
ODP-AM09-2-O-104	170	Capsule	VF0144	Immune modulation
ODP-AM09-3-104	157	Capsule	VF0144	Immune modulation
ODP-AM09-3-104	157	Neuraminidase	VF0148	Exoenzyme
ODP-AM09-3-104	157	PfbA	VF0525	Adherence
ODP-AM09-31-O-BH	63	PavB	VF0524	Adherence
ODP-AM09-32-BH	157	Capsule	VF0144	Immune modulation
ODP-AM09-32-BH	157	Neuraminidase	VF0148	Exoenzyme
ODP-AM09-32-BH	157	PfbA	VF0525	Adherence
ODP-AM09-33-BH	169	Capsule	VF0144	Immune modulation
ODP-AM09-33-BH	169	Capsule	VF0361	Immune modulation
ODP-AM09-33-BH	169	PsaA	VF0151	Nutritional/Metabolic factor
ODP-AM09-33-BH	169	CBPs	VF0145	Adherence
ODP-AM09-33-O-BH	63	PavB	VF0524	Adherence
ODP-AM09-34-BH	46	Capsule	VF0144	Immune modulation
ODP-AM09-36-O-BH	157	Capsule	VF0144	Immune modulation
ODP-AM09-36-O-BH	157	Neuraminidase	VF0148	Exoenzyme
ODP-AM09-36-O-BH	157	PfbA	VF0525	Adherence
ODP-AM09-38-O-BH	157	Capsule	VF0144	Immune modulation
ODP-AM09-38-O-BH	157	Neuraminidase	VF0148	Exoenzyme
ODP-AM09-38-O-BH	157	PfbA	VF0525	Adherence
ODP-AM09-39-BH	63	PavB	VF0524	Adherence
ODP-AM09-39-O-BH	85	Capsule	VF0144	Immune modulation
ODP-AM09-39-O-BH	85	CBPs	VF0145	Adherence
ODP-AM09-39-O-BH	85	Neuraminidase	VF0148	Exoenzyme
ODP-AM09-39-O-BH	85	PfbA	VF0525	Adherence
ODP-AM09-3-BH	157	Capsule	VF0144	Immune modulation
ODP-AM09-3-BH	157	Neuraminidase	VF0148	Exoenzyme
ODP-AM09-3-BH	157	PfbA	VF0525	Adherence
ODP-AM09-3-O-104	170	PfbA	VF0525	Adherence
ODP-AM09-3-O-104	170	CBPs	VF0145	Adherence
ODP-AM09-3-O-104	170	Capsule	VF0144	Immune modulation
ODP-AM09-42-O-BH	157	Capsule	VF0144	Immune modulation
ODP-AM09-42-O-BH	157	Neuraminidase	VF0148	Exoenzyme
ODP-AM09-42-O-BH	157	PfbA	VF0525	Adherence
ODP-AM09-43-BH	63	PavB	VF0524	Adherence
ODP-AM09-45-O-BH	157	Capsule	VF0144	Immune modulation
ODP-AM09-45-O-BH	157	Neuraminidase	VF0148	Exoenzyme
ODP-AM09-45-O-BH	157	PfbA	VF0525	Adherence
ODP-AM09-46-O-BH	163	Neuraminidase	VF0148	Exoenzyme
ODP-AM09-46-O-BH	163	PfbA	VF0525	Adherence
ODP-AM09-46-O-BH	163	CBPs	VF0145	Adherence
ODP-AM09-46-O-BH	163	PavB	VF0524	Adherence
ODP-AM09-46-O-BH	163	Capsule	VF0361	Immune modulation
ODP-AM09-46-O-BH	163	Capsule	VF0144	Immune modulation
ODP-AM09-47-O-BH	157	Capsule	VF0144	Immune modulation
ODP-AM09-47-O-BH	157	Neuraminidase	VF0148	Exoenzyme
ODP-AM09-47-O-BH	157	PfbA	VF0525	Adherence
ODP-AM09-48-O-BH	63	PavB	VF0524	Adherence
ODP-AM09-49-O-BH	79	Neuraminidase	VF0148	Exoenzyme
ODP-AM09-49-O-BH	79	Capsule	VF0144	Immune modulation
ODP-AM09-4-O-104	46	Capsule	VF0144	Immune modulation
ODP-AM09-5-104	163	Neuraminidase	VF0148	Exoenzyme
ODP-AM09-5-104	163	PfbA	VF0525	Adherence
ODP-AM09-5-104	163	CBPs	VF0145	Adherence
ODP-AM09-5-104	163	Capsule	VF0144	Immune modulation
ODP-AM09-5-104	163	Capsule	VF0361	Immune modulation
ODP-AM09-5-O-BH	3	PavB	VF0524	Adherence
ODP-AM09-6-104	158	PfbA	VF0525	Adherence
ODP-AM09-6-O-104	107	Neuraminidase	VF0148	Exoenzyme
ODP-AM09-6-O-104	107	PI-2	VF0530	Adherence
ODP-AM09-6-O-104	107	Capsule	VF0144	Immune modulation
ODP-AM09-6-O-104	107	PavB	VF0524	Adherence
ODP-AM09-7-BH	157	Capsule	VF0144	Immune modulation
ODP-AM09-7-BH	157	Neuraminidase	VF0148	Exoenzyme
ODP-AM09-7-BH	157	PfbA	VF0525	Adherence
ODP-AM09-9-104	158	PfbA	VF0525	Adherence
ODP-AM09-9-104	158	Neuraminidase	VF0148	Exoenzyme
ODP-AM09-9-O-104	107	Capsule	VF0144	Immune modulation
ODP-AM09-9-O-104	107	PI-2	VF0530	Adherence
ODP-AM09-9-O-104	107	PavB	VF0524	Adherence
ODP-AM09-9-O-BH	157	Capsule	VF0144	Immune modulation
ODP-AM09-9-O-BH	157	PfbA	VF0525	Adherence
ODP-AM09-9-O-BH	157	Neuraminidase	VF0148	Exoenzyme
ODP-TM06-145	23	ClpP	VF0074	Stress survival
ODP-TM06-145	23	GroEL	VF0594	Adherence
ODP-TM06-153	84	ClpP	VF0074	Stress survival
ODP-TM06-153	84	IgA1 protease	VF0147	Immune modulation
ODP-TM06-153	84	PsaA	VF0151	Nutritional/Metabolic factor
ODP-TM06-155	23	GroEL	VF0594	Adherence
ODP-TM06-155	23	ClpP	VF0074	Stress survival
ODP-TM06-159	102	Neuraminidase	VF0148	Exoenzyme
ODP-TM06-159	102	Capsule	VF0144	Immune modulation
ODP-TM06-47	63	PavB	VF0524	Adherence
ODP-TM06-50	63	PavB	VF0524	Adherence
ODP-TM06-55	63	PavB	VF0524	Adherence
ODP-TM07-115	23	GroEL	VF0594	Adherence
ODP-TM07-115	23	ClpP	VF0074	Stress survival
ODP-TM07-125	34	GroEL	VF0594	Adherence
ODP-TM07-125	34	ClpP	VF0074	Stress survival
ODP-TM07-131	34	GroEL	VF0594	Adherence
ODP-TM07-131	34	ClpP	VF0074	Stress survival
ODP-TM07-134	92	ClpP	VF0074	Stress survival
ODP-TM07-145	34	GroEL	VF0594	Adherence
ODP-TM07-145	34	ClpP	VF0074	Stress survival
ODP-TM07-152	34	GroEL	VF0594	Adherence
ODP-TM07-152	34	ClpP	VF0074	Stress survival
ODP-TM07-155	23	ClpP	VF0074	Stress survival
ODP-TM07-155	23	GroEL	VF0594	Adherence
ODP-TM07-157	92	ClpP	VF0074	Stress survival
ODP-TM07-158	36	PavB	VF0524	Adherence
ODP-TM07-162	107	Capsule	VF0144	Immune modulation
ODP-TM07-162	107	PI-2	VF0530	Adherence

ODP-TM07-162	107	Neuraminidase	VF0148	Exoenzyme
ODP-TM07-162	107	PavB	VF0524	Adherence
ODP-TM07-198	36	PavB	VF0524	Adherence
ODP-TM07-209	35	Capsule	VF0144	Immune modulation
ODP-TM07-209	35	Neuraminidase	VF0148	Exoenzyme
ODP-TM07-209	35	PavB	VF0524	Adherence
ODP-TM07-212	11	Capsule	VF0144	Immune modulation
ODP-TM07-212	11	PI-2	VF0530	Adherence
ODP-TM07-212	11	Neuraminidase	VF0148	Exoenzyme
ODP-TM07-212	11	PavB	VF0524	Adherence
ODP-TM07-271	36	PavB	VF0524	Adherence
ODP-TM07-272	107	PI-2	VF0530	Adherence
ODP-TM07-272	107	Capsule	VF0144	Immune modulation
ODP-TM07-272	107	Neuraminidase	VF0148	Exoenzyme
ODP-TM07-272	107	PavB	VF0524	Adherence
ORS-AF03-140	1	ClpP	VF0074	Stress survival
ORS-AF03-141	79	Neuraminidase	VF0148	Exoenzyme
ORS-AF03-141	79	Capsule	VF0144	Immune modulation
ORS-AF03-142	1	ClpP	VF0074	Stress survival
ORS-AF03-155	141	PavB	VF0524	Adherence
ORS-AF03-155	141	CBPs	VF0145	Adherence
ORS-AF03-155	141	Capsule	VF0361	Immune modulation
ORS-AF03-155	141	PfbA	VF0525	Adherence
ORS-AF03-170	89	Capsule	VF0144	Immune modulation
ORS-AF03-170	89	Neuraminidase	VF0148	Exoenzyme
ORS-AF04-127	52	PavB	VF0524	Adherence
ORS-AF04-140	23	PfbA	VF0525	Adherence
ORS-AF04-140	23	ClpP	VF0074	Stress survival
ORS-AF04-140	23	GroEL	VF0594	Adherence
ORS-AF04-142	111	Neuraminidase	VF0148	Exoenzyme
ORS-AF04-142	111	Autolysin	VF0143	Exoenzyme
ORS-AF04-142	111	Capsule	VF0144	Immune modulation
ORS-AF04-252	7	PfbA	VF0525	Adherence
ORS-AF04-252	7	Capsule	VF0144	Immune modulation
ORS-AF04-252	7	Neuraminidase	VF0148	Exoenzyme
ORS-AF04-41	3	PavB	VF0524	Adherence
ORS-AF04-42	9	Capsule	VF0144	Immune modulation
ORS-AF04-42	9	PfbA	VF0525	Adherence
ORS-AF04-42	9	Capsule	VF0361	Immune modulation
ORS-AF04-42	9	CBPs	VF0145	Adherence
ORS-AF04-54	107	PI-2	VF0530	Adherence
ORS-AF04-54	107	Capsule	VF0144	Immune modulation
ORS-AF04-54	107	Neuraminidase	VF0148	Exoenzyme
ORS-AF04-54	107	PavB	VF0524	Adherence
ORS-AF04-59	107	PI-2	VF0530	Adherence
ORS-AF04-59	107	Capsule	VF0144	Immune modulation
ORS-AF04-59	107	Neuraminidase	VF0148	Exoenzyme
ORS-AF04-59	107	PavB	VF0524	Adherence
ORS-AF05-07	8	Capsule	VF0144	Immune modulation
ORS-AF05-07	8	Capsule	VF0361	Immune modulation
ORS-AF05-07	8	Neuraminidase	VF0148	Exoenzyme
ORS-AF05-07	8	IgA1 protease	VF0147	Immune modulation
ORS-AF05-07	8	PfbA	VF0525	Adherence
ORS-AF05-11	9	Capsule	VF0144	Immune modulation
ORS-AF05-11	9	PfbA	VF0525	Adherence
ORS-AF05-11	9	CBPs	VF0145	Adherence
ORS-AF05-11	9	Capsule	VF0361	Immune modulation
ORS-AF05-19	49	IgA1 protease	VF0147	Immune modulation
ORS-AF05-19	49	ClpP	VF0074	Stress survival
ORS-AF05-19	49	PsaA	VF0151	Nutritional/Metabolic factor
ORS-AF05-220	10	Capsule	VF0144	Immune modulation
ORS-AF05-30	43	ClpP	VF0074	Stress survival
ORS-AF06-121	86	Capsule	VF0144	Immune modulation
ORS-AF06-121	86	Neuraminidase	VF0148	Exoenzyme
ORS-AF06-121	86	PavB	VF0524	Adherence
ORS-AF06-159	5	PfbA	VF0525	Adherence
ORS-AF06-69	61	SLS	VF0251	Exotoxin
ORS-AF08-10	107	Capsule	VF0144	Immune modulation
ORS-AF08-10	107	Neuraminidase	VF0148	Exoenzyme
ORS-AF08-10	107	PavB	VF0524	Adherence
ORS-AF08-10	107	PI-2	VF0530	Adherence
ORS-AF08-11	127	PavB	VF0524	Adherence
ORS-AF08-11	127	PfbA	VF0525	Adherence
ORS-AF08-12-MRS	157	Capsule	VF0144	Immune modulation
ORS-AF08-12-MRS	157	Neuraminidase	VF0148	Exoenzyme
ORS-AF08-12-MRS	157	PavB	VF0524	Adherence
ORS-AF08-13B	106	GroEL	VF0594	Adherence
ORS-AF08-13B	106	ClpP	VF0074	Stress survival
ORS-AF08-13	107	Capsule	VF0144	Immune modulation
ORS-AF08-13	107	PI-2	VF0530	Adherence
ORS-AF08-13	107	PavB	VF0524	Adherence
ORS-AF08-14-MRS	106	GroEL	VF0594	Adherence
ORS-AF08-14-MRS	106	ClpP	VF0074	Stress survival
ORS-AF08-14	107	Capsule	VF0144	Immune modulation
ORS-AF08-14	107	PavB	VF0524	Adherence
ORS-AF08-14	107	PI-2	VF0530	Adherence
ORS-AF08-15	4	PavB	VF0524	Adherence
ORS-AF08-16-MRS	176	Neuraminidase	VF0148	Exoenzyme
ORS-AF08-16-MRS	176	PavB	VF0524	Adherence
ORS-AF08-17-MRS	157	Capsule	VF0144	Immune modulation
ORS-AF08-17-MRS	157	Neuraminidase	VF0148	Exoenzyme
ORS-AF08-17-MRS	157	PavB	VF0524	Adherence
ORS-AF08-17	107	Capsule	VF0144	Immune modulation
ORS-AF08-17	107	PavB	VF0524	Adherence
ORS-AF08-17	107	CBPs	VF0145	Adherence
ORS-AF08-17	107	PI-2	VF0530	Adherence
ORS-AF08-18	46	Capsule	VF0144	Immune modulation
ORS-AF08-19	107	Capsule	VF0144	Immune modulation
ORS-AF08-19	107	PavB	VF0524	Adherence
ORS-AF08-19	107	PI-2	VF0530	Adherence
ORS-AF08-1-MRS	120	PavB	VF0524	Adherence
ORS-AF08-1	176	Neuraminidase	VF0148	Exoenzyme
ORS-AF08-20	170	Capsule	VF0144	Immune modulation
ORS-AF08-20	170	CBPs	VF0145	Adherence
ORS-AF08-21	107	Capsule	VF0144	Immune modulation
ORS-AF08-21	107	PavB	VF0524	Adherence
ORS-AF08-21	107	PI-2	VF0530	Adherence
ORS-AF08-22	107	PI-2	VF0530	Adherence
ORS-AF08-22	107	Capsule	VF0144	Immune modulation
ORS-AF08-22	107	PavB	VF0524	Adherence
ORS-AF08-23	176	Neuraminidase	VF0148	Exoenzyme
ORS-AF08-24	107	Capsule	VF0144	Immune modulation
ORS-AF08-24	107	PavB	VF0524	Adherence
ORS-AF08-24	107	PI-2	VF0530	Adherence
ORS-AF08-27-MRS	106	ClpP	VF0074	Stress survival
ORS-AF08-27-MRS	106	GroEL	VF0594	Adherence
ORS-AF08-2-MRS	4	PavB	VF0524	Adherence
ORS-AF08-3-MRS	77	ClpP	VF0074	Stress survival
ORS-AF08-3-MRS	77	GroEL	VF0594	Adherence
ORS-AF08-4-MRS	107	Capsule	VF0144	Immune modulation
ORS-AF08-4-MRS	107	PI-2	VF0530	Adherence

ORS-AF08-4-MRS	107	PavB	VF0524	Adherence
ORS-AF08-8-MRS	170	Capsule	VF0144	Immune modulation
ORS-AF08-8-MRS	170	CBPs	VF0145	Adherence
ORS-AF08-8-MRS	170	PfbA	VF0525	Adherence
ORS-AF08-8	154	PavB	VF0524	Adherence
ORS-AF10-10-BH	194	Capsule	VF0144	Immune modulation
ORS-AF10-10-BH	194	PfbA	VF0525	Adherence
ORS-AF10-10-BH	194	Neuraminidase	VF0148	Exoenzyme
ORS-AF10-10-BH	194	Capsule	VF0361	Immune modulation
ORS-AF10-11-BH	194	Capsule	VF0144	Immune modulation
ORS-AF10-11-BH	194	PfbA	VF0525	Adherence
ORS-AF10-11-BH	194	Neuraminidase	VF0148	Exoenzyme
ORS-AF10-11-BH	194	Capsule	VF0361	Immune modulation
ORS-AF10-12-BH	95	ClpP	VF0074	Stress survival
ORS-AF10-12-BH	95	GroEL	VF0594	Adherence
ORS-AF10-13-BH	157	Capsule	VF0144	Immune modulation
ORS-AF10-13-BH	157	Neuraminidase	VF0148	Exoenzyme
ORS-AF10-14-BH	95	ClpP	VF0074	Stress survival
ORS-AF10-14-BH	95	GroEL	VF0594	Adherence
ORS-AF10-17-O-BH	15	Polar flagella	VF0473	Motility
ORS-AF10-17-O-BH	15	Capsule	VF0079	Immune modulation
ORS-AF10-17-O-BH	15	MntABC	VF0455	Stress survival
ORS-AF10-17-O-BH	15	HmbR	VF0048	Nutritional/Metabolic factor
ORS-AF10-17-O-BH	15	MtrCDE	VF0451	ntimicrobial activity/Competitive advantage
ORS-AF10-17-O-BH	15	Type IV pili	VF0075	Adherence
ORS-AF10-20-O-BH	186	Capsule	VF0144	Immune modulation
ORS-AF10-20-O-BH	186	PI-2	VF0530	Adherence
ORS-AF10-20-O-BH	186	Neuraminidase	VF0148	Exoenzyme
ORS-AF10-20-O-BH	186	PfbA	VF0525	Adherence
ORS-AF10-20-O-BH	186	PavB	VF0524	Adherence
ORS-AF10-22-BH	186	PI-2	VF0530	Adherence
ORS-AF10-22-BH	186	Capsule	VF0144	Immune modulation
ORS-AF10-22-BH	186	Neuraminidase	VF0148	Exoenzyme
ORS-AF10-22-BH	186	PfbA	VF0525	Adherence
ORS-AF10-22-BH	186	PavB	VF0524	Adherence
ORS-AF10-24-BH	195	GroEL	VF0594	Adherence
ORS-AF10-24-BH	195	ClpP	VF0074	Stress survival
ORS-AF10-24-O-BH	15	Polar flagella	VF0473	Motility
ORS-AF10-24-O-BH	15	Capsule	VF0079	Immune modulation
ORS-AF10-24-O-BH	15	MntABC	VF0455	Stress survival
ORS-AF10-24-O-BH	15	HmbR	VF0048	Nutritional/Metabolic factor
ORS-AF10-24-O-BH	15	MtrCDE	VF0451	ntimicrobial activity/Competitive advantage
ORS-AF10-24-O-BH	15	Type IV pili	VF0075	Adherence
ORS-AF10-3-BH	184	Capsule	VF0144	Immune modulation
ORS-AF10-3-BH	184	PfbA	VF0525	Adherence
ORS-AF10-5-BH	157	Capsule	VF0144	Immune modulation
ORS-AF10-5-BH	157	Neuraminidase	VF0148	Exoenzyme
ORS-AF10-5-BH	157	PfbA	VF0525	Adherence
ORS-AF10-6-O-BH	15	Capsule	VF0079	Immune modulation
ORS-AF10-6-O-BH	15	Polar flagella	VF0473	Motility
ORS-AF10-6-O-BH	15	MntABC	VF0455	Stress survival
ORS-AF10-6-O-BH	15	HmbR	VF0048	Nutritional/Metabolic factor
ORS-AF10-6-O-BH	15	MtrCDE	VF0451	ntimicrobial activity/Competitive advantage
ORS-AF10-6-O-BH	15	Type IV pili	VF0075	Adherence
ORS-AF10-7-BH	107	Capsule	VF0144	Immune modulation
ORS-AF10-7-BH	107	PI-2	VF0530	Adherence
ORS-AF10-7-BH	107	PfbA	VF0525	Adherence
ORS-AF10-7-BH	107	Neuraminidase	VF0148	Exoenzyme
ORS-AF10-7-BH	107	PavB	VF0524	Adherence
ORS-AF10-8-BH	157	Capsule	VF0144	Immune modulation
ORS-AF10-8-BH	157	Neuraminidase	VF0148	Exoenzyme
ORS-AF10-8-BH	157	PfbA	VF0525	Adherence
ORS-AF10-9-BH	170	Capsule	VF0144	Immune modulation
ORS-AF10-9-BH	170	CBPs	VF0145	Adherence
ORS-AF10-9-BH	170	Capsule	VF0361	Immune modulation
ORS-AF10-9-BH	170	PfbA	VF0525	Adherence
ORS-AF10-C12	192	CBPs	VF0145	Adherence
ORS-AF10-C13	192	CBPs	VF0145	Adherence
ORS-AF10-C13	192	PavB	VF0524	Adherence
ORS-AF10-C15	193	Capsule	VF0144	Immune modulation
ORS-AF10-C19	194	PfbA	VF0525	Adherence
ORS-AF10-C19	194	Neuraminidase	VF0148	Exoenzyme
ORS-AF10-C19	194	Capsule	VF0144	Immune modulation
ORS-AF10-C19	194	Capsule	VF0361	Immune modulation
ORS-AF10-C20	194	Capsule	VF0144	Immune modulation
ORS-AF10-C20	194	PfbA	VF0525	Adherence
ORS-AF10-C20	194	Neuraminidase	VF0148	Exoenzyme
ORS-AF10-C20	194	Capsule	VF0361	Immune modulation
ORS-AF10-C8	188	Capsule	VF0144	Immune modulation
ORS-AF10-C8	188	PfbA	VF0525	Adherence
ORS-AF10-C8	188	Neuraminidase	VF0148	Exoenzyme
ORS-AF10-C8	188	PavB	VF0524	Adherence
ORS-AM01-01O	15	MtrCDE	VF0451	ntimicrobial activity/Competitive advantage
ORS-AM01-01O	15	MntABC	VF0455	Stress survival
ORS-AM01-02BB	135	Neuraminidase	VF0148	Exoenzyme
ORS-AM01-02BB	135	PavB	VF0524	Adherence
ORS-AM01-04BBO	136	MntABC	VF0455	Stress survival
ORS-AM01-04BBO	136	Type IV pili	VF0075	Adherence
ORS-AM01-04BBO	136	Polar flagella	VF0473	Motility
ORS-AM01-04MB	46	Capsule	VF0144	Immune modulation
ORS-AM01-13TBO	15	Polar flagella	VF0473	Motility
ORS-AM01-13TBO	15	Capsule	VF0079	Immune modulation
ORS-AM01-13TBO	15	MtrCDE	VF0451	ntimicrobial activity/Competitive advantage
ORS-AM01-13TBO	15	MntABC	VF0455	Stress survival
ORS-AM01-13TBO	15	HmbR	VF0048	Nutritional/Metabolic factor
ORS-AM04-111	25	GroEL	VF0594	Adherence
ORS-AM04-111	25	ClpP	VF0074	Stress survival
ORS-AM04-35	4	PavB	VF0524	Adherence
ORS-AM04-85	23	ClpP	VF0074	Stress survival
ORS-AM04-85	23	GroEL	VF0594	Adherence
ORS-AM04-86	3	PavB	VF0524	Adherence
ORS-AM05-122	31	Capsule	VF0144	Immune modulation
ORS-AM05-128	46	Capsule	VF0144	Immune modulation
ORS-AM05-133	100	Neuraminidase	VF0148	Exoenzyme
ORS-AM05-138	11	Capsule	VF0144	Immune modulation
ORS-AM05-138	11	Neuraminidase	VF0148	Exoenzyme
ORS-AM05-138	11	PavB	VF0524	Adherence
ORS-AM05-141	11	Capsule	VF0144	Immune modulation
ORS-AM05-141	11	Neuraminidase	VF0148	Exoenzyme
ORS-AM05-141	11	PavB	VF0524	Adherence
ORS-AM05-177	46	Capsule	VF0144	Immune modulation
ORS-AM05-183	11	Capsule	VF0144	Immune modulation
ORS-AM05-183	11	Neuraminidase	VF0148	Exoenzyme
ORS-AM05-183	11	PavB	VF0524	Adherence
ORS-AM05-194	11	Capsule	VF0144	Immune modulation
ORS-AM05-194	11	Neuraminidase	VF0148	Exoenzyme
ORS-AM05-194	11	PavB	VF0524	Adherence
ORS-AM05-197	85	Capsule	VF0144	Immune modulation
ORS-AM05-197	85	CBPs	VF0145	Adherence
ORS-AM05-197	85	PfbA	VF0525	Adherence
ORS-AM05-197	85	PavB	VF0524	Adherence

ORS-AM05-325	15	Polar flagella	VF0473	Motility
ORS-AM05-325	15	Type IV pili	VF0075	Adherence
ORS-AM05-325	15	MtrCDE	VF0451	ntimicrobial activity/Competitive advantage
ORS-AM05-325	15	MntABC	VF0455	Stress survival
ORS-AM05-334	64	MntABC	VF0455	Stress survival
ORS-AM05-334	64	Capsule	VF0079	Immune modulation
ORS-AM05-336	99	ClpP	VF0074	Stress survival
ORS-AM05-336	99	ClpC	VF0072	Stress survival
ORS-AM05-342	99	ClpC	VF0072	Stress survival
ORS-AM05-342	99	ClpP	VF0074	Stress survival
ORS-AM05-365	16	PavB	VF0524	Adherence
ORS-AM05-381	82	Capsule	VF0144	Immune modulation
ORS-AM05-471	148	ClpP	VF0074	Stress survival
ORS-AM05-471	148	GroEL	VF0594	Adherence
ORS-AM05-478	16	PavB	VF0524	Adherence
ORS-AM05-481	101	Capsule	VF0144	Immune modulation
ORS-AM05-481	101	PfbA	VF0525	Adherence
ORS-AM05-481	101	CBPs	VF0145	Adherence
ORS-AM05-481	101	Capsule	VF0361	Immune modulation
ORS-AM05-481	101	PavB	VF0524	Adherence
ORS-AM05-491	35	Capsule	VF0144	Immune modulation
ORS-AM05-491	35	Neuraminidase	VF0148	Exoenzyme
ORS-AM05-491	35	PavB	VF0524	Adherence
ORS-AM05-495	46	Capsule	VF0144	Immune modulation
ORS-AM08-171	18	GroEL	VF0594	Adherence
ORS-AM08-171	18	ClpP	VF0074	Stress survival
ORS-AM08-24	25	GroEL	VF0594	Adherence
ORS-AM08-24	25	ClpP	VF0074	Stress survival
ORS-AM08-34	106	ClpP	VF0074	Stress survival
ORS-AM08-34	106	GroEL	VF0594	Adherence
ORS-AM08-44	18	ClpP	VF0074	Stress survival
ORS-AM08-44	18	GroEL	VF0594	Adherence
ORS-AM08-46	106	ClpP	VF0074	Stress survival
ORS-AM08-46	106	GroEL	VF0594	Adherence
ORS-AM08-48	106	GroEL	VF0594	Adherence
ORS-AM08-48	106	ClpP	VF0074	Stress survival
ORS-AM08-51	18	ClpP	VF0074	Stress survival
ORS-AM08-51	18	GroEL	VF0594	Adherence
ORS-AM08-63	115	Neuraminidase	VF0148	Exoenzyme
ORS-AM08-69	106	ClpP	VF0074	Stress survival
ORS-AM08-69	106	GroEL	VF0594	Adherence
ORS-AM09-10-104	115	PavB	VF0524	Adherence
ORS-AM09-10-O-104	113	PavB	VF0524	Adherence
ORS-AM09-10-O-BH	157	Capsule	VF0144	Immune modulation
ORS-AM09-10-O-BH	157	Neuraminidase	VF0148	Exoenzyme
ORS-AM09-10-O-BH	157	PfbA	VF0525	Adherence
ORS-AM09-1-104	158	PfbA	VF0525	Adherence
ORS-AM09-1-104	158	Neuraminidase	VF0148	Exoenzyme
ORS-AM09-11-O-104	107	Capsule	VF0144	Immune modulation
ORS-AM09-11-O-104	107	PI-2	VF0530	Adherence
ORS-AM09-11-O-104	107	PavB	VF0524	Adherence
ORS-AM09-1-2-O-BH	156	Capsule	VF0144	Immune modulation
ORS-AM09-1-2-O-BH	156	PavB	VF0524	Adherence
ORS-AM09-13-O-104	172	Neuraminidase	VF0148	Exoenzyme
ORS-AM09-14-O-104	172	Neuraminidase	VF0148	Exoenzyme
ORS-AM09-16-104	163	PfbA	VF0525	Adherence
ORS-AM09-16-104	163	Neuraminidase	VF0148	Exoenzyme
ORS-AM09-16-104	163	CBPs	VF0145	Adherence
ORS-AM09-16-104	163	Capsule	VF0361	Immune modulation
ORS-AM09-16-104	163	Capsule	VF0144	Immune modulation
ORS-AM09-16-104	163	PavB	VF0524	Adherence
ORS-AM09-16-BH	163	Neuraminidase	VF0148	Exoenzyme
ORS-AM09-16-BH	163	PfbA	VF0525	Adherence
ORS-AM09-16-BH	163	CBPs	VF0145	Adherence
ORS-AM09-16-BH	163	Capsule	VF0361	Immune modulation
ORS-AM09-16-BH	163	Capsule	VF0144	Immune modulation
ORS-AM09-16-O-BH	136	MntABC	VF0455	Stress survival
ORS-AM09-16-O-BH	136	Type IV pili	VF0075	Adherence
ORS-AM09-16-O-BH	136	Polar flagella	VF0473	Motility
ORS-AM09-16-O-BH	136	MtrCDE	VF0451	ntimicrobial activity/Competitive advantage
ORS-AM09-17-O-BH	157	Capsule	VF0144	Immune modulation
ORS-AM09-17-O-BH	157	Neuraminidase	VF0148	Exoenzyme
ORS-AM09-17-O-BH	157	PfbA	VF0525	Adherence
ORS-AM09-18-104	159	Neuraminidase	VF0148	Exoenzyme
ORS-AM09-19-104	157	Capsule	VF0144	Immune modulation
ORS-AM09-19-104	157	PfbA	VF0525	Adherence
ORS-AM09-19-104	157	Neuraminidase	VF0148	Exoenzyme
ORS-AM09-19-BH	17	ClpP	VF0074	Stress survival
ORS-AM09-19-O-BH	158	PfbA	VF0525	Adherence
ORS-AM09-19-O-BH	158	Neuraminidase	VF0148	Exoenzyme
ORS-AM09-1-O-104	86	Neuraminidase	VF0148	Exoenzyme
ORS-AM09-1-O-104	86	Capsule	VF0144	Immune modulation
ORS-AM09-20-O-BH	36	PavB	VF0524	Adherence
ORS-AM09-2-104	165	ClpP	VF0074	Stress survival
ORS-AM09-22-104	85	Capsule	VF0144	Immune modulation
ORS-AM09-22-104	85	CBPs	VF0145	Adherence
ORS-AM09-22-104	85	Neuraminidase	VF0148	Exoenzyme
ORS-AM09-22-104	85	PfbA	VF0525	Adherence
ORS-AM09-22-O-BH	73	Neuraminidase	VF0148	Exoenzyme
ORS-AM09-22-O-BH	73	PavB	VF0524	Adherence
ORS-AM09-24-O-BH	159	Neuraminidase	VF0148	Exoenzyme
ORS-AM09-24-O-BH	159	PavB	VF0524	Adherence
ORS-AM09-27-O-104	107	PI-2	VF0530	Adherence
ORS-AM09-27-O-104	107	Capsule	VF0144	Immune modulation
ORS-AM09-27-O-104	107	PavB	VF0524	Adherence
ORS-AM09-2-O-104	174	Capsule	VF0144	Immune modulation
ORS-AM09-2-O-104	174	Neuraminidase	VF0148	Exoenzyme
ORS-AM09-3-104	181	ClpP	VF0074	Stress survival
ORS-AM09-31-1-O-BH	15	Capsule	VF0079	Immune modulation
ORS-AM09-31-1-O-BH	15	Polar flagella	VF0473	Motility
ORS-AM09-31-1-O-BH	15	MntABC	VF0455	Stress survival
ORS-AM09-31-2-O-BH	15	Capsule	VF0079	Immune modulation
ORS-AM09-31-2-O-BH	15	Polar flagella	VF0473	Motility
ORS-AM09-31-2-O-BH	15	MntABC	VF0455	Stress survival
ORS-AM09-32-BH	49	ClpP	VF0074	Stress survival
ORS-AM09-32-BH	49	PsaA	VF0151	Nutritional/Metabolic factor
ORS-AM09-32-BH	49	IgA1 protease	VF0147	Immune modulation
ORS-AM09-39-O-BH	159	Neuraminidase	VF0148	Exoenzyme
ORS-AM09-4-104	120	PavB	VF0524	Adherence
ORS-AM09-41-O-BH	15	Capsule	VF0079	Immune modulation
ORS-AM09-41-O-BH	15	Polar flagella	VF0473	Motility
ORS-AM09-41-O-BH	15	MntABC	VF0455	Stress survival
ORS-AM09-43-O-BH	79	Neuraminidase	VF0148	Exoenzyme
ORS-AM09-43-O-BH	79	Capsule	VF0144	Immune modulation
ORS-AM09-45-O-BH	161	Capsule	VF0144	Immune modulation
ORS-AM09-45-O-BH	161	CBPs	VF0145	Adherence
ORS-AM09-4-O-104	107	Capsule	VF0144	Immune modulation
ORS-AM09-4-O-104	107	PI-2	VF0530	Adherence
ORS-AM09-4-O-104	107	PavB	VF0524	Adherence
ORS-AM09-5-104	182	PfbA	VF0525	Adherence
ORS-AM09-5-104	182	PavB	VF0524	Adherence

ORS-AM09-5-104	182	IgA1 protease	VF0147	Immune modulation
ORS-AM09-5-104	182	Capsule	VF0144	Immune modulation
ORS-AM09-5-104	182	Capsule	VF0361	Immune modulation
ORS-AM09-5-BH	165	ClpP	VF0074	Stress survival
ORS-AM09-5D1B	92	ClpP	VF0074	Stress survival
ORS-AM09-5D2A	92	ClpP	VF0074	Stress survival
ORS-AM09-7-BH	95	ClpP	VF0074	Stress survival
ORS-AM09-7-BH	95	GroEL	VF0594	Adherence
ORS-AM09-7-O-104	63	PavB	VF0524	Adherence
ORS-AM09-8-O-BH	157	Capsule	VF0144	Immune modulation
ORS-AM09-8-O-BH	157	Neuraminidase	VF0148	Exoenzyme
ORS-AM09-8-O-BH	157	PfbA	VF0525	Adherence
ORS-AM09-9-104	157	Capsule	VF0144	Immune modulation
ORS-AM09-9-104	157	PfbA	VF0525	Adherence
ORS-AM09-9-104	157	Neuraminidase	VF0148	Exoenzyme
ORS-AM09-9-O-104	107	Capsule	VF0144	Immune modulation
ORS-AM09-9-O-104	107	PI-2	VF0530	Adherence
ORS-AM09-9-O-104	107	PavB	VF0524	Adherence
ORS-AM09-9-O-BH	157	Capsule	VF0144	Immune modulation
ORS-AM09-9-O-BH	157	Neuraminidase	VF0148	Exoenzyme
ORS-AM09-9-O-BH	157	PfbA	VF0525	Adherence
ORS-TM06-05	61	Capsule	VF0144	Immune modulation
ORS-TM06-06	63	PavB	VF0524	Adherence
ORS-TM06-07	104	SpaP	VF0526	Adherence
ORS-TM06-100	84	ClpP	VF0074	Stress survival
ORS-TM06-100	84	IgA1 protease	VF0147	Immune modulation
ORS-TM06-100	84	PsaA	VF0151	Nutritional/Metabolic factor
ORS-TM06-104	37	ClpP	VF0074	Stress survival
ORS-TM06-105	102	Neuraminidase	VF0148	Exoenzyme
ORS-TM06-105	102	Capsule	VF0144	Immune modulation
ORS-TM06-106	103	ClpP	VF0074	Stress survival
ORS-TM06-106	103	GroEL	VF0594	Adherence
ORS-TM06-109	57	ClpP	VF0074	Stress survival
ORS-TM06-110	84	ClpP	VF0074	Stress survival
ORS-TM06-110	84	IgA1 protease	VF0147	Immune modulation
ORS-TM06-110	84	PsaA	VF0151	Nutritional/Metabolic factor
ORS-TM06-111	61	Capsule	VF0144	Immune modulation
ORS-TM06-114	84	PsaA	VF0151	Nutritional/Metabolic factor
ORS-TM06-114	84	ClpP	VF0074	Stress survival
ORS-TM06-114	84	IgA1 protease	VF0147	Immune modulation
ORS-TM06-118	61	Capsule	VF0144	Immune modulation
ORS-TM06-134	23	GroEL	VF0594	Adherence
ORS-TM06-134	23	ClpP	VF0074	Stress survival
ORS-TM06-140	92	ClpP	VF0074	Stress survival
ORS-TM06-15	56	PavB	VF0524	Adherence
ORS-TM06-215	19	ClpP	VF0074	Stress survival
ORS-TM06-224	4	PavB	VF0524	Adherence
ORS-TM06-226	19	ClpP	VF0074	Stress survival
ORS-TM06-235	92	ClpP	VF0074	Stress survival
ORS-TM06-27	63	PavB	VF0524	Adherence
ORS-TM06-36	31	Capsule	VF0144	Immune modulation
ORS-TM06-36	31	PavB	VF0524	Adherence
ORS-TM06-37	15	Capsule	VF0079	Immune modulation
ORS-TM06-37	15	MtrCDE	VF0451	ntimicrobial activity/Competitive advantage
ORS-TM06-37	15	HmbR	VF0048	Nutritional/Metabolic factor
ORS-TM06-37	15	MntABC	VF0455	Stress survival
ORS-TM06-82	17	ClpP	VF0074	Stress survival
ORS-TM06-83	18	ClpP	VF0074	Stress survival
ORS-TM06-83	18	GroEL	VF0594	Adherence
ORS-TM06-89	36	PavB	VF0524	Adherence
ORS-TM06-89	36	Capsule	VF0144	Immune modulation
ORS-TM06-90	55	ClpP	VF0074	Stress survival
ORS-TM06-91	57	ClpP	VF0074	Stress survival
ORS-TM06-93	18	ClpP	VF0074	Stress survival
ORS-TM06-93	18	GroEL	VF0594	Adherence
ORS-TM06-94	57	ClpP	VF0074	Stress survival
ORS-TM06-97	17	ClpP	VF0074	Stress survival
ORS-TM06-99	23	GroEL	VF0594	Adherence
ORS-TM06-99	23	ClpP	VF0074	Stress survival
ORS-TM07-02	21	Ebp pili	VF0538	Adherence
ORS-TM07-02	21	EfaA	VF0354	Adherence
ORS-TM07-02	21	Capsule	VF0361	Immune modulation
ORS-TM07-02	21	Hyaluronidase	VF0359	Exoenzyme
ORS-TM07-02	21	ClpP	VF0074	Stress survival
ORS-TM07-03	23	ClpP	VF0074	Stress survival
ORS-TM07-03	23	GroEL	VF0594	Adherence
ORS-TM07-11	21	EfaA	VF0354	Adherence
ORS-TM07-11	21	Ebp pili	VF0538	Adherence
ORS-TM07-11	21	Capsule	VF0361	Immune modulation
ORS-TM07-11	21	ClpP	VF0074	Stress survival
ORS-TM07-11	21	Hyaluronidase	VF0359	Exoenzyme
ORS-TM07-215	24	PavB	VF0524	Adherence
ORS-TM07-219	85	CBPs	VF0145	Adherence
ORS-TM07-219	85	PfbA	VF0525	Adherence
ORS-TM07-219	85	Capsule	VF0144	Immune modulation
ORS-TM07-225	85	Capsule	VF0144	Immune modulation
ORS-TM07-225	85	CBPs	VF0145	Adherence
ORS-TM07-225	85	PfbA	VF0525	Adherence
ORS-TM07-227	143	PI-2	VF0530	Adherence
ORS-TM07-227	143	Neuraminidase	VF0148	Exoenzyme
ORS-TM07-227	143	PavB	VF0524	Adherence
ORS-TM07-227	143	Capsule	VF0144	Immune modulation
ORS-TM07-227	143	Capsule	VF0361	Immune modulation
ORS-TM07-240	11	PI-2	VF0530	Adherence
ORS-TM07-240	11	Capsule	VF0144	Immune modulation
ORS-TM07-240	11	Neuraminidase	VF0148	Exoenzyme
ORS-TM07-240	11	PavB	VF0524	Adherence
ORS-TM07-255	123	ClpP	VF0074	Stress survival
ORS-TM07-255	123	PavB	VF0524	Adherence
ORS-TM07-255	123	PI-2	VF0530	Adherence
ORS-TM07-269	11	PI-2	VF0530	Adherence
ORS-TM07-269	11	Capsule	VF0144	Immune modulation
ORS-TM07-269	11	Neuraminidase	VF0148	Exoenzyme
ORS-TM07-269	11	PavB	VF0524	Adherence
ORS-TM07-270	34	ClpP	VF0074	Stress survival
ORS-TM07-270	34	GroEL	VF0594	Adherence
ORS-TM07-35	21	Ebp pili	VF0538	Adherence
ORS-TM07-35	21	EfaA	VF0354	Adherence
ORS-TM07-35	21	Capsule	VF0361	Immune modulation
ORS-TM07-35	21	Hyaluronidase	VF0359	Exoenzyme
ORS-TM07-35	21	ClpP	VF0074	Stress survival
ORS-TM07-40	21	Capsule	VF0361	Immune modulation
ORS-TM07-40	21	EfaA	VF0354	Adherence
ORS-TM07-40	21	Ebp pili	VF0538	Adherence
ORS-TM07-40	21	Hyaluronidase	VF0359	Exoenzyme
ORS-TM07-40	21	ClpP	VF0074	Stress survival
ORS-TM07-54	21	Ebp pili	VF0538	Adherence
ORS-TM07-54	21	EfaA	VF0354	Adherence
ORS-TM07-54	21	Capsule	VF0361	Immune modulation
ORS-TM07-54	21	ClpP	VF0074	Stress survival
ORS-TM07-54	21	Hyaluronidase	VF0359	Exoenzyme

ORS-TM07-73	21	Capsule	VF0361	Immune modulation
ORS-TM07-73	21	EfaA	VF0354	Adherence
ORS-TM07-73	21	Ebp pili	VF0538	Adherence
ORS-TM07-73	21	ClpP	VF0074	Stress survival
ORS-TM07-73	21	Hyaluronidase	VF0359	Exoenzyme
ORS-TM07-76	55	ClpP	VF0074	Stress survival
ORS-TM07-77	55	ClpP	VF0074	Stress survival
ORS-TM07-79	55	ClpP	VF0074	Stress survival
ORT-AF03-04	70	PavB	VF0524	Adherence
ORT-AF03-215	126	Autolysin	VF0143	Exoenzyme
ORT-AF03-215	126	PavB	VF0524	Adherence
ORT-AF03-68	17	ClpP	VF0074	Stress survival
ORT-AF03-76	94	ClpP	VF0074	Stress survival
ORT-AF03-76	94	IgA1 protease	VF0147	Immune modulation
ORT-AF03-76	94	PsaA	VF0151	Nutritional/Metabolic factor
ORT-AF04-16	110	Neuraminidase	VF0148	Exoenzyme
ORT-AF04-215	43	ClpP	VF0074	Stress survival
ORT-AF04-246	7	Capsule	VF0144	Immune modulation
ORT-AF04-246	7	PfbA	VF0525	Adherence
ORT-AF04-246	7	Neuraminidase	VF0148	Exoenzyme
ORT-AF04-247	127	Autolysin	VF0143	Exoenzyme
ORT-AF04-247	127	Neuraminidase	VF0148	Exoenzyme
ORT-AF04-248	95	GroEL	VF0594	Adherence
ORT-AF04-248	95	ClpP	VF0074	Stress survival
ORT-AF05-109	21	Ebp pili	VF0538	Adherence
ORT-AF05-109	21	Capsule	VF0361	Immune modulation
ORT-AF05-109	21	Hyaluronidase	VF0359	Exoenzyme
ORT-AF05-109	21	EfaA	VF0354	Adherence
ORT-AF05-109	21	ClpP	VF0074	Stress survival
ORT-AF05-113	95	ClpP	VF0074	Stress survival
ORT-AF05-113	95	GroEL	VF0594	Adherence
ORT-AF05-117	21	Ebp pili	VF0538	Adherence
ORT-AF05-117	21	Capsule	VF0361	Immune modulation
ORT-AF05-117	21	Hyaluronidase	VF0359	Exoenzyme
ORT-AF05-117	21	EfaA	VF0354	Adherence
ORT-AF05-117	21	ClpP	VF0074	Stress survival
ORT-AF05-121	95	GroEL	VF0594	Adherence
ORT-AF05-121	95	ClpP	VF0074	Stress survival
ORT-AF05-123	21	Capsule	VF0361	Immune modulation
ORT-AF05-123	21	EfaA	VF0354	Adherence
ORT-AF05-123	21	Ebp pili	VF0538	Adherence
ORT-AF05-123	21	Hyaluronidase	VF0359	Exoenzyme
ORT-AF05-123	21	ClpP	VF0074	Stress survival
ORT-AF05-128	21	Ebp pili	VF0538	Adherence
ORT-AF05-128	21	EfaA	VF0354	Adherence
ORT-AF05-128	21	Capsule	VF0361	Immune modulation
ORT-AF05-128	21	Hyaluronidase	VF0359	Exoenzyme
ORT-AF05-128	21	ClpP	VF0074	Stress survival
ORT-AF06-16	73	Autolysin	VF0143	Exoenzyme
ORT-AF06-16	73	Capsule	VF0144	Immune modulation
ORT-AF06-16	73	Neuraminidase	VF0148	Exoenzyme
ORT-AF06-16	73	PavB	VF0524	Adherence
ORT-AF06-30	5	PfbA	VF0525	Adherence
ORT-AF06-36	73	Neuraminidase	VF0148	Exoenzyme
ORT-AF06-36	73	PavB	VF0524	Adherence
ORT-AF08-10	185	Capsule	VF0144	Immune modulation
ORT-AF08-11-MRS	178	ClpP	VF0074	Stress survival
ORT-AF08-11	107	Capsule	VF0144	Immune modulation
ORT-AF08-11	107	PI-2	VF0530	Adherence
ORT-AF08-11	107	PavB	VF0524	Adherence
ORT-AF08-12-O-MRS	4	PavB	VF0524	Adherence
ORT-AF08-13	107	PavB	VF0524	Adherence
ORT-AF08-13	107	PI-2	VF0530	Adherence
ORT-AF08-13	107	Capsule	VF0144	Immune modulation
ORT-AF08-14-O-MRS	104	SpaP	VF0526	Adherence
ORT-AF08-14-O-MRS	104	PavB	VF0524	Adherence
ORT-AF08-16	79	Capsule	VF0144	Immune modulation
ORT-AF08-16	79	PavB	VF0524	Adherence
ORT-AF08-16	79	Neuraminidase	VF0148	Exoenzyme
ORT-AF08-17	185	Capsule	VF0144	Immune modulation
ORT-AF08-17	185	PavB	VF0524	Adherence
ORT-AF08-19	104	PavB	VF0524	Adherence
ORT-AF08-21-O-MRS	107	Capsule	VF0144	Immune modulation
ORT-AF08-21-O-MRS	107	PI-2	VF0530	Adherence
ORT-AF08-21-O-MRS	107	PavB	VF0524	Adherence
ORT-AF08-28-MRS	46	Capsule	VF0144	Immune modulation
ORT-AF08-32-MRS	46	Capsule	VF0144	Immune modulation
ORT-AF08-34-MRS	107	Capsule	VF0144	Immune modulation
ORT-AF08-34-MRS	107	PavB	VF0524	Adherence
ORT-AF08-34-MRS	107	PI-2	VF0530	Adherence
ORT-AF08-3	46	Capsule	VF0144	Immune modulation
ORT-AF08-43-MRS	107	Neuraminidase	VF0148	Exoenzyme
ORT-AF08-43-MRS	107	PI-2	VF0530	Adherence
ORT-AF08-43-MRS	107	Capsule	VF0144	Immune modulation
ORT-AF08-43-MRS	107	PavB	VF0524	Adherence
ORT-AF08-4	173	Capsule	VF0144	Immune modulation
ORT-AF08-4	173	Neuraminidase	VF0148	Exoenzyme
ORT-AF08-5	176	Neuraminidase	VF0148	Exoenzyme
ORT-AF08-5	176	PavB	VF0524	Adherence
ORT-AF08-7	171	PavB	VF0524	Adherence
ORT-AF10-05-O-BH	188	Capsule	VF0144	Immune modulation
ORT-AF10-05-O-BH	188	PavB	VF0524	Adherence
ORT-AF10-05-O-BH	188	Neuraminidase	VF0148	Exoenzyme
ORT-AF10-05-O-BH	188	PfbA	VF0525	Adherence
ORT-AF10-07-O-BH	189	Capsule	VF0144	Immune modulation
ORT-AF10-07-O-BH	189	Capsule	VF0361	Immune modulation
ORT-AF10-11-O-BH	15	Polar flagella	VF0473	Motility
ORT-AF10-11-O-BH	15	Capsule	VF0079	Immune modulation
ORT-AF10-11-O-BH	15	MntABC	VF0455	Stress survival
ORT-AF10-11-O-BH	15	HmbR	VF0048	Nutritional/Metabolic factor
ORT-AF10-11-O-BH	15	MtrCDE	VF0451	ntimicrobial activity/Competitive advantage
ORT-AF10-11-O-BH	15	Type IV pili	VF0075	Adherence
ORT-AF10-14-O-BH	35	Capsule	VF0144	Immune modulation
ORT-AF10-14-O-BH	35	Neuraminidase	VF0148	Exoenzyme
ORT-AF10-14-O-BH	35	PfbA	VF0525	Adherence
ORT-AF10-15-O-BH	159	Neuraminidase	VF0148	Exoenzyme
ORT-AF10-20-O-BH	63	PavB	VF0524	Adherence

Supplementary Table 4a. *Streptococcus* genomes with compele pathway of quorum sensing

Strain	Sampling	Completeness of each pathway<br>Streptococcus-1	Streptococcus-2	Streptococcus-3
ODP-AM09-2-O-104	ODP	1	1	0.8
ODP-AM09-9-O-BH	ODP	1	1	0.8
ORS-AM09-22-104	ORS	1	1	0.8
ORS-TM06-36	ORS	1	1	0.8
ODP-AM09-12-104	ODP	1	1	0.8
ORS-AM05-491	ORS	1	0.833333333	0.8
ORS-AM09-4-O-104	ORS	1	1	0.8
ORT-AM05-511	ORT	0.8	0.333333333	1
ORT-AF08-32-MRS	ORT	0.8	0.333333333	1
ORS-AF08-4-MRS	ORS	1	1	0.8
ORT-AM05-512	ORT	0.8	0.333333333	1
ODP-TM07-162	ODP	1	0.833333333	0.8
ODP-AF08-21-MRS	ODP	1	1	0.8
ODP-AF06-108	ODP	1	0.833333333	0.6
ODP-AM09-3-O-104	ODP	1	1	0.8
ODP-AF08-16-MRS	ODP	0.8	0.333333333	1
ORS-AF03-170	ORS	1	1	0.8
ORT-AF10-07-O-BH	ORT	1	0.833333333	0.8
ODP-AM01-08	ODP	1	0.833333333	0.8
ODP-AF08-42-MRS	ODP	1	1	0.8
ORT-AF10-C1	ORT	1	1	0.8
ORS-AF08-8-MRS	ORS	1	1	0.8
ODP-TM07-209	ODP	1	0.833333333	0.6
ODP-AM09-20-O-104	ODP	1	1	0.8
ODP-AF08-23-MRS	ODP	1	1	0.8
ODP-AF08-36-MRS	ODP	1	1	0.8
ODP-AF08-38-MRS	ODP	1	1	0.8
ORS-AM05-133	ORS	1	0.833333333	0.6
ODP-AM09-26-104	ODP	1	0.833333333	0.8
ORS-AM09-9-O-BH	ORS	1	1	0.8
ODP-AM09-27-BH	ODP	1	1	0.8
ORT-AF08-3	ORT	0.4	0	1
ORT-AM09-24-O-104	ORT	1	1	0.8
ORS-AF08-10	ORS	1	1	0.8
ODP-AM05-262	ODP	0.8	0.333333333	1
ODP-AM09-9-104	ODP	1	1	0.8
ORS-AF08-24	ORS	1	1	0.8
ODP-AM05-395	ODP	0.8	0.333333333	1
ORS-AM05-197	ORS	1	1	0.8
ODP-AM05-504	ODP	1	1	0.8
ODP-AM09-20-104	ODP	1	1	0.8
ODP-AM09-5-104	ODP	1	1	0.8
ODP-AM05-19	ODP	1	1	0.8
ODP-AF06-99	ODP	1	1	0.8
ORS-AF05-11	ORS	1	1	0.8
ODP-AF08-13-MRS	ODP	0.8	0.333333333	1
ODP-AM09-18-O-104	ODP	1	1	0.8
ORS-AM09-19-O-BH	ORS	1	1	0.8
ODP-AF04-83	ODP	1	1	0.8
ORS-AF10-13-BH	ORS	1	1	0.8
ODP-AF08-41-MRS	ODP	1	1	0.8
ORS-AM09-8-104	ORS	1	1	0.8
ODP-AM09-10-O-104	ODP	1	1	0.8
ORS-AF10-10-BH	ORS	1	1	0.8
ORT-AM08-195	ORT	1	0.833333333	0.6
ODP-AM09-45-O-BH	ODP	1	1	0.8
ODP-AM05-457	ODP	1	1	0.8
ODP-AM08-343	ODP	0.8	0.333333333	1
ODP-AM09-49-O-BH	ODP	1	0.833333333	0.6
ORS-AF10-C19	ORS	1	1	0.8
ORS-AM09-45-O-BH	ORS	1	1	0.8
ORT-AM09-22-O-104	ORT	1	1	0.8
ORT-AM05-453	ORT	0.8	0.333333333	1
ORT-AF08-13	ORT	1	1	0.8
ODP-AM09-1-BH	ODP	1	1	0.8
ODP-AF10-C21	ODP	1	0.833333333	0.6
ODP-AM09-24-BH	ODP	1	1	0.8
ORS-AM09-1-2-O-BH	ORS	1	1	0.8
ORS-AM09-1-104	ORS	1	1	0.8
ODP-AF03-102	ODP	1	1	0.8
ORS-AM09-40-O-BH	ORS	1	1	0.8
ODP-AM09-3-104	ODP	1	1	0.8
ORS-AM09-14-O-104	ORS	1	1	0.8
ODP-AM09-20-O-BH	ODP	1	1	0.8
ORS-AF08-12-MRS	ORS	1	1	0.8
ORS-AM08-63	ORS	1	0.833333333	0.8
ORS-AF08-20	ORS	1	1	0.8
ODP-AM09-17-104	ODP	1	0.833333333	0.8
ODP-AM09-36-O-BH	ODP	1	1	0.8
ORS-AF05-07	ORS	1	1	0.8
ORS-AF08-17-MRS	ORS	1	1	0.8
ORS-AF10-C20	ORS	1	1	0.8
ORT-AF08-28-MRS	ORT	0.8	0.333333333	1
ORT-AF08-21-O-MRS	ORT	1	1	0.8
ORS-AM09-13-O-104	ORS	1	1	0.8
ODP-AM09-19-O-104	ODP	1	1	0.8
ORS-AF10-C8	ORS	1	1	0.8
ORT-AF10-C12	ORT	1	0.833333333	0.8
ORS-AM05-481	ORS	1	1	0.8
ODP-AF08-8-MRS	ODP	1	1	0.8
ORS-AF04-59	ORS	1	1	0.8
ODP-AM09-6-O-104	ODP	1	1	0.8
ORS-AF08-22	ORS	1	1	0.8
ORT-AM09-3-O-104	ORT	1	1	0.8
ODP-AM09-34-BH	ODP	0.8	0.333333333	1
ODP-AF10-C6	ODP	0.8	0.333333333	1
ORS-AF08-21	ORS	1	1	0.8
ODP-AM09-3-BH	ODP	1	1	0.8
ORS-AF10-9-BH	ORS	1	1	0.8
ODP-AM09-6-104	ODP	1	1	0.8
ODP-AM09-21-O-104	ODP	1	1	0.8
ORS-AF10-22-BH	ORS	1	0.833333333	0.8
ORS-TM07-219	ORS	1	1	0.8
ODP-AM09-46-O-BH	ODP	1	1	0.8
ODP-AF08-40-MRS	ODP	0.8	0.333333333	1
ORS-AF08-13	ORS	1	1	0.8
ORT-AF10-C11	ORT	1	0.833333333	0.8

ODP-AM09-23-BH	ODP	1	0.833333333	0.8
ORT-AM05-89	ORT	1	0.833333333	0.8
ODP-AM09-16-O-104	ODP	1	1	0.8
ODP-AM05-263	ODP	1	1	0.8
ORS-AF10-C13	ORS	1	1	0.8
ODP-AM09-13-104	ODP	1	1	0.8
ORS-AM09-10-104	ORS	1	0.833333333	0.8
ORT-AF08-43-MRS	ORT	1	1	0.8
ORS-AF10-11-BH	ORS	1	1	0.8
ORS-TM07-225	ORS	1	1	0.8
ODP-AM09-38-O-BH	ODP	1	1	0.8
ORT-AM05-05	ORT	1	0.833333333	0.6
ODP-AM05-266	ODP	0.8	0.333333333	1
ODP-AM09-39-O-BH	ODP	1	1	0.8
ORS-AM09-9-O-104	ORS	1	1	0.8
ODP-AF08-39-MRS	ODP	1	1	0.8
ORS-AM09-10-O-BH	ORS	1	1	0.8
ORS-AM09-8-O-BH	ORS	1	1	0.8
ODP-AM05-458	ODP	1	1	0.8
ODP-AM09-33-BH	ODP	1	1	0.8
ODP-AM09-21-104	ODP	1	1	0.8
ODP-AM09-22-BH	ODP	1	1	0.8
ORT-AF10-05-O-BH	ORT	1	1	0.8
ORS-AM09-11-O-104	ORS	1	1	0.8
ORT-AF08-34-MRS	ORT	1	1	0.8
ODP-AM09-47-O-BH	ODP	1	1	0.8
ORS-AM09-17-O-BH	ORS	1	1	0.8
ODP-AF08-10-MRS	ODP	1	1	0.8
ODP-AM09-1-1-O-BH	ODP	1	1	0.8
ODP-AM09-15-104	ODP	1	1	0.8
ODP-AM09-10-104	ODP	1	1	0.8
ODP-AF08-35-MRS	ODP	1	1	0.8
ODP-AM05-506	ODP	1	1	0.8
ODP-AF08-2	ODP	1	1	0.8
ODP-TM07-272	ODP	1	0.833333333	0.8
ODP-AF04-88	ODP	1	1	0.8
ODP-AM09-32-BH	ODP	1	1	0.8
ORT-AM09-18-O-104	ORT	1	1	0.8
ODP-AM09-9-O-104	ODP	1	1	0.8
ODP-AF04-250	ODP	0.8	0.333333333	1
ODP-AM09-23-104	ODP	1	1	0.8
ODP-AM09-1-2-O-BH	ODP	1	1	0.8
ODP-AF08-20-MRS	ODP	0.8	0.333333333	1
ODP-AF08-7-MRS	ODP	1	1	0.8
ORS-AF10-7-BH	ORS	1	0.833333333	0.6
ORT-AF08-11	ORT	1	1	0.8
ORT-AM09-6-O-104	ORT	1	1	0.8
ODP-AF10-C23	ODP	1	1	0.8
ORS-AF10-20-O-BH	ORS	1	0.833333333	0.8
ODP-AF04-94	ODP	1	1	0.8
ORS-AF08-14	ORS	1	1	0.8
ORT-AF03-04	ORT	0.8	0.333333333	1
ORS-AM09-43-O-BH	ORS	1	0.666666667	0.4
ORS-AM09-19-104	ORS	1	1	0.8
ODP-AM09-12-O-BH	ODP	1	1	0.8
ODP-AM09-27-104	ODP	1	1	0.8
ODP-AM05-80	ODP	1	1	0.8
ODP-AM09-16-O-BH	ODP	1	1	0.8
ORS-AF10-8-BH	ORS	1	1	0.8
ORS-AM09-27-O-104	ORS	1	1	0.8
ORS-AM05-122	ORS	1	1	0.8
ORS-AM09-16-104	ORS	1	1	0.8
ORS-TM06-113	ORS	1	1	0.8
ODP-AM09-23-O-104	ODP	1	1	0.8
ORS-AM05-128	ORS	0.8	0.333333333	1
ORS-AF10-5-BH	ORS	1	1	0.8
ODP-AM09-12-O-104	ODP	0.8	0.333333333	1
ORT-AM09-2-O-104	ORT	1	1	0.8
ODP-AM09-42-O-BH	ODP	1	1	0.8
ODP-AF08-3	ODP	0.8	0.333333333	1
ORS-AF08-19	ORS	1	1	0.8
ORS-AM05-177	ORS	0.8	0.333333333	1
ORS-AF10-C12	ORS	1	1	0.8
ORS-AF04-42	ORS	1	1	0.8
ORS-AF04-54	ORS	1	1	0.8
ODP-AM05-252	ODP	1	1	0.8
ORT-AF10-14-O-BH	ORT	1	0.833333333	0.6
ORS-AM09-2-O-104	ORS	1	1	0.8
ORT-AM05-529	ORT	1	0.833333333	0.6
ORT-AF08-16	ORT	1	1	0.8
ORS-AM09-5-104	ORS	1	1	0.8
ODP-AM09-22-O-104	ODP	1	1	0.8
ORS-AF03-155	ORS	1	1	1
ORS-AF08-17	ORS	1	1	0.8
ORS-AF03-141	ORS	1	1	0.8
ORS-AM09-16-BH	ORS	1	1	0.8
ORS-AM09-9-104	ORS	1	1	0.8
ODP-AM09-7-BH	ODP	1	1	0.8
ODP-AM01-02BB	ODP	1	0.833333333	0.8
ORS-AM05-495	ORS	0.8	0.333333333	1

Supplementary Table 4b. The data of crystal violet assay (normalized)

Strain	Sampling	A1	A2	A3	A4	A5	A6	A7	A8	A9	A10	A11	A12
S. constellatus_ODP-AF08-16-MRS	ODP	0.02805	0.02805	0.02315	0.03295	0.03	0.0278	0.03	0.0365	0.04815	0.17375	0.05935	0.08825
S. salivarius_ODP-AM09-2D5	ODP	0.07165	0.05555	0.14275	0.09655	0.0411	0.0711	0.0307	0.0445	0.06065	0.08325	0.08485	0.05355
S. salivarius_ORS-AF08-23-MRS	ORS	0.14755	0.13835	0.10995	0.09735	0.0548	0.0796	0.0275	0.0294	0.05335	0.23385	0.27105	0.22535
S. oralis_ORS-AF08-4-MRS	ORS	0.01275	0.03285	0.03605	0.04785	0.0148	0.03	0.022	0.0074	0.01465	0.04705	0.05835	0.05205
S. mitis_ORS-AM05-478	ORS	-0.00695	0.01335	0.00285	0.02285	0.0149	0.0349	0.0124	0.0201	0.01235	0.03095	0.00565	0.00905
S. salivarius_ORT-AF10-26-O-BH	ORT	0.05475	0.05685	0.08235	0.12985	0.0181	0.0303	0.0157	0.0566	0.06155	0.07175	0.07725	0.08015

Supplementary Table 5. The degree of clusters in correlation network analysis

Cluster Name	Cluster	degree
Streptococcus unknow_160	160	98
Streptococcus unknow_130	130	95
Streptococcus unknow_100	100	94
Streptococcus unknow_172	172	94
Streptococcus unknow_122	122	94
Streptococcus unknow_156	156	93
Streptococcus unknow_118	118	88
Streptococcus unknow_126	126	86
Streptococcus unknow_7	7	85
Streptococcus unknow_108	108	83
Streptococcus oralis_173	173	83
Streptococcus pseudopneumoniae/unknow_115	115	83
Streptococcus unknow_162	162	82
Streptococcus unknow_102	102	82
Streptococcus sp015256435_111	111	82
Streptococcus unknow_50	50	82
Streptococcus unknow_86	86	81
Streptococcus oralis_31	31	80
Granulicatella adiacens_23	23	78
Streptococcus oralis_35	35	78
Streptococcus sp900550895_186	186	77
Streptococcus sp002355895_112	112	76
Streptococcus oralis_79	79	76
Streptococcus unknow_143	143	75
Streptococcus unknow_168	168	74
Streptococcus unknow_124	124	74
Streptococcus cristatus_52	52	73
Streptococcus mitis_11	11	72
Streptococcus cristatus_36	36	72
Streptococcus oralis_93	93	72
Granulicatella sp905371865_69	69	70
Granulicatella adiacens/unknow_34	34	69
Streptococcus sp902460355_139	139	69
Streptococcus unknow_193	193	69
Streptococcus mitis/unknow_101	101	68
Streptococcus unknow_174	174	68
Streptococcus unknow_116	116	68
Streptococcus symci_157	157	68
Streptococcus mitis/symci_89	89	68
Streptococcus unknow_194	194	67
Streptococcus oralis/unknow_107	107	67
Streptococcus xiaochunlingii_56	56	67
Streptococcus unknow_189	189	66
Streptococcus gordonii_63	63	66
Streptococcus unknow_9	9	66
Streptococcus mitis/unknow_170	170	66
Streptococcus mitis_85	85	66
Streptococcus unknow_161	161	66
Streptococcus oralis_163	163	65
Streptococcus unknow_8	8	65
Streptococcus unknow_72	72	65
Streptococcus sinensis_24	24	64
Streptococcus cristatus_83	83	63
Streptococcus unknow_40	40	63
Streptococcus unknow_192	192	62
Streptococcus mitis_169	169	62

Streptococcus pseudopneumoniae_119	119	62
Streptococcus infantis_73	73	61
Streptococcus mitis_188	188	60
Streptococcus unknow_171	171	60
Streptococcus intermedius_70	70	60
Gemella unknow_84	84	58
Streptococcus unknow_110	110	58
Streptococcus unknow_127	127	58
Streptococcus sanguinis_113	113	58
Gemella haemolysans_151	151	57
Streptococcus mitis_182	182	57
Granulicatella unknow_106	106	56
Streptococcus unknow_154	154	56
Gemella unknow_94	94	55
Granulicatella sp905371865_30	30	55
Streptococcus unknow_135	135	55
Streptococcus sp000187745_141	141	53
Streptococcus unknow_158	158	53
Streptococcus sanguinis_29	29	53
Granulicatella unknow_18	18	52
Granulicatella sp015264885_103	103	51
Streptococcus unknow_176	176	51
Streptococcus sanguinis_3	3	51
Gemella unknow_181	181	50
Granulicatella sp001071995_195	195	50
Streptococcus anginosus_104	104	50
Streptococcus unknow_185	185	50
Streptococcus unknow_98	98	50
Streptococcus unknow_60	60	49
Granulicatella unknow_95	95	48
Streptococcus unknow_22	22	48
Gemella haemolysans_49	49	47
Streptococcus xiaochunlingii_5	5	47
Streptococcus unknow_184	184	45
Streptococcus koreensis/unknow_68	68	45
Streptococcus infantis_159	159	44
Streptococcus sp001813295_183	183	43
Streptococcus unknow_120	120	42
Streptococcus parasanguinis_53	53	42
Granulicatella unknow_123	123	41
Streptococcus parasanguinis_20	20	40
Granulicatella unknow_57	57	38
Streptococcus parasanguinis_71	71	38
Streptococcus parasanguinis/unknow_6	6	37
Pauljensenia sp000411415_166	166	33
Gemella morbillorum_65	65	32
Rothia mucilaginosa/unknow_78	78	30
Rothia mucilaginosa_59	59	30
Veillonella unknow_19	19	30
Streptococcus anginosus_61	61	30
Rothia sp015265375_91	91	29
Bulleidia sp015256775_82	82	29
Granulicatella unknow_77	77	29
Streptococcus koreensis_48	48	29
Lancefieldella unknow_155	155	27
Lancefieldella unknow_96	96	27
Lancefieldella unknow_149	149	27
Lancefieldella sp000564995_114	114	27

Rothia unknow_109	109	27
Streptococcus timonensis/mitis_16	16	27
Lancefieldella unknow_26	26	26
Lancefieldella unknow_2	2	26
Streptococcus rubneri_14	14	26
Pauljensenia sp001064145_42	42	25
Veillonella atypica_178	178	24
Veillonella unknow_179	179	24
Granulicatella unknow_25	25	24
Streptococcus unknow_74	74	24
Rothia aeria_32	32	22
Pauljensenia sp001838165_147	147	22
Pauljensenia sp000185285_153	153	22
Rothia mucilaginosa_41	41	21
Pauljensenia sp018382595_97	97	20
Pauljensenia sp000466265_105	105	19
Pauljensenia sp902373545_45	45	17
Pauljensenia sp000278725_121	121	17
Prevotella histicola_28	28	17
Gemella sanguinis_17	17	17
Streptococcus constellatus_46	46	17
Streptococcus sp001556435_117	117	17
Pauljensenia odontolytica_38	38	16
Rothia dentocariosa_13	13	15
Streptococcus salivarius_4	4	15
Actinomyces naeslundii_33	33	14
Actinomyces johnsonii_66	66	13
Capnocytophaga sputigena_90	90	13
Pauljensenia unknow_144	144	12
Leptotrichia massiliensis_67	67	12
Pauljensenia sp018375675_76	76	11
Prevotella sp000257925_62	62	11
Prevotella melaninogenica_175	175	11
Lachnoanaerobaculum unknow_75	75	11
Pauljensenia unknow_167	167	10
Pauljensenia odontolytica_27	27	10
Actinomyces oris_58	58	9
Neisseria elongata_64	64	9
Actinomyces oris_39	39	8
Bulleidia unknow_47	47	8
Streptococcus vestibularis_131	131	8
Lachnoanaerobaculum gingivalis_88	88	8
Corynebacterium accolens_125	125	7
Pauljensenia hongkongensis_132	132	7
Bacillus tropicus_191	191	7
Eubacterium infirmum_148	148	7
Lachnoanaerobaculum sp000296385_165	165	7
Fusobacterium unknow_133	133	7
Fusobacterium polymorphum_87	87	7
Fusobacterium hwasookii_142	142	7
sobacterium pseudoperiodonticum/periodonticum_	80	7
Priestia megaterium_140	140	6
Staphylococcus hominis_99	99	6
Capnocytophaga ochracea_190	190	5
Bacillus mobilis_164	164	5
Enterococcus faecalis_21	21	5
Enterococcus casseliflavus_1	1	5
Oribacterium unknow_10	10	5

Neisseria sicca_44	44	5
Fusobacterium animalis_146	146	5
Corynebacterium argentoratense_54	54	4
Prevotella loescheii_150	150	4
Neisseria sicca_187	187	4
Leptotrichia wadei_152	152	4
Catonella unknow_134	134	4
Microbacterium algeriense_12	12	3
Capnocytophaga leadbetteri_81	81	3
Neisseria cerebrosa/sicca_136	136	3
Veillonella sp900757715_177	177	2
Veillonella parvula_92	92	2
Lancefieldella rimae_128	128	2
Peptoanaerobacter unknow_138	138	2
Neisseria subflava/flavescens_15	15	2
Bacillus paralicheniformis_145	145	1
Bacillus luti_137	137	1
Abiotrophia defectiva_37	37	1
Abiotrophia sp001815865_55	55	1

Supplementary Table 6. Culture media used for isolation of the oral bacteria

Noted: Since aerobic and anaerobic bacteria both exist in the oral cavity, we used both aerobic and anaerobic culture conditions.
1. For all the anaerobic culture conditions, we additionally supplemented 0.50 g/L Cysteine-HCl x H_2O, 0.25 g/L Na_2S, and 1 mg/L resazurin in each medium formulation listed below.
2. For the blood-media, we additionally supplemented 2.5% sterile defibrinated sheep blood in the corresponding steriled media.
3. As for the blood-enriched BHI medium, we firstly inoculated the samples to 5 mL of the sterile sheep blood for 2-3 days, and used blood-BHI medium for bacteria isolation.

Medium 1. BHI medium	
**Component**	**Amount/L**
Brain infusion solids	12.5 g
Beef heart infusion solids	5.0 g
Proteose peptone	10 g
Glucose	2.0 g
Sodium chloride	5.0 g
Disodium phosphate	2.5 g
Agar	15.0 g
pH	7.4

Medium 2. Columbia medium	
**Component**	**Amount/L**
Casein Tryptone	10.0 g
Pepsin Hydrolytes	5.0 g
Heart Pancreatin Hydrolytes	3.0 g
Yeast Extract	5.0 g
Corn starch	1.0 g
NaCl	5.0 g
Noble Agar	15.0 g
pH	7.3 ± 0.2

Medium 3. H medium	
**Component**	**Amount/L**
Tryptone	10.0 g
Sodium Chloride	8.0 g
Agar	15.0 g
pH	7.0

Medium 4. J medium	
**Component**	**Amount/L**
Beef Dip Powder	10.0 g
Peptone	10.0 g
Lactose	10.0 g
Sodium citrate	20.0 g
Ferric citrate	1.0 g
Sodium deoxycholate	5.0 g
Agar	15.0 g
Neutral red	0.02 g
pH	7.3 ± 0.2

Medium 5. TSB medium	
**Component**	**Amount/L**
Tryptone	17.0 g
Soytone	3.0 g
Glucose	2.5 g
Sodium Chloride	5.0 g
Dipotassium Phosphate	2.5 g
Agar	15.0 g
pH	7.3 ± 0.2

Medium 6. Spore medium	
**Component**	**Amount/L**
Yeast Extract	1.0 g
Beef extract	1.0 g
Tryptone Peptone	2.0 g
Glucose	10.0 g
FeSO4	0.001 g
Agar	15.0 g
pH	7.2

Medium 7. SCH medium	
**Component**	**Amount/L**
Tryptone Peptone	8.2 g
Peptone	2.5 g
Peptone from soya	1.0 g
Glucose	5.8 g
Yeast extract	5.0 g
NaCl	1.7 g
NaHCO3	0.8 g
Cysteine-HCl·H2O	0.4 g
Haemin	0.01 g
Tris	15.0 g
Agar	15.0 g
pH	7.2

Medium 8. MRS medium	
**Component**	**Amount/L**
Peptone	10.0 g
Yeast extract	5.0 g
Meat extract	10.0 g
Glucose	20.0 g
Polysorbate 80	1.0 g
Sodium acetate	5.0 g
Magnesium sulfate	0.1 g
Manganese sulfate	0.05 g
Disodium phosphate	2.0 g
Agar	15.0 g
pH	6.2

Medium 9. MPYG medium	
**Component**	**Amount/L**
Trypticase peptone	5.00 g
Peptone	3.00 g
Peptone from soya	2.00 g
Polypeptone	1.00 g
Yeast extract	10.00 g
Beef extract	5.00 g
Glucose	5.00 g
Tween 80	0.50 mL
Maltose	0.50 g
Cellobiose	0.50 g
Starch, soluble	0.50 g
Glycerol	0.50 mL
K2HPO4	2.00 g
Salt solution (see below)	40.00 mL
Trace element(see below)	10.00 mL
Vitamin solution(see below)	10.00 mL
Haemin solution (see below)	10.00 mL
Vitamin K1 solution (see below	0.20 mL
Agar	15 g
**Salt solution(DSMZ Salt solution):**	
CaCl2 x 2 H2O	0.25 g
MgSO4 x 7 H2O	0.50 g
K2HPO4	1.00 g
KH2PO4	1.00 g
NaHCO3	10.00 g
NaCl	2.00 g
**Trace element solution(DSMZ Trace element solution):**	
Nitrilotriacetic acid	1.50 g
MgSO4 x 7 H2O	3.00 g
MnSO4 x H2O	0.50 g
NaCl	1.00 g
FeSO4 x 7 H2O	0.10 g
CoSO4 x 7 H2O	0.18 g
CaCl2 x 2 H2O	0.10 g
ZnSO4 x 7 H2O	0.18 g
CuSO4 x 5 H2O	0.01 g
KAl(SO4)2 x 12 H2O	0.02 g
H3BO3	0.01 g
Na2MoO4 x 2 H2O	0.01 g
NiCl2 x 6 H2O	0.03 g
Na2SeO3 x 5 H2O	0.30 mg
**Vitamin solution:**	
Biotin	2.00 mg
Folic acid	2.00 mg
Pyridoxine-HCl	10.00 mg
Thiamine-HCl x 2 H2O	5.00 mg
Riboflavin	5.00 mg
Nicotinic acid	5.00 mg
D-Ca-pantothenate	5.00 mg
Vitamin B12	0.10 mg
p-Aminobenzoic acid	5.00 mg
Lipoic acid	5.00 mg
Reference: https://doi.org/10.1038/s41587-018-0008-8	
