# Supplementary material for: A catalog of bacterial reference genomes from cultivated human oral bacteria
Source: NPJ Biofilms Microbiomes. 2023 Jul 3;9:45. doi: 10.1038/s41522-023-00414-3 (PMC10318035; doi:10.1038/s41522-023-00414-3)
Supplement: Supplementary file 2 — Reporting Summary [file 41522_2023_414_MOESM2_ESM.pdf]

nature portfolio

Corresponding author(s): Karsten Kristiansen, Liang Xiao, and Yuanqiang Zou

Last updated by author(s): May 10, 2023

Reporting Summary

Nature Portfolio wishes to improve the reproducibility of the work that we publish. This form provides structure for consistency and transparency in reporting. For further information on Nature Portfolio policies, see our Editorial Policies and the Editorial Policy Checklist.

Statistics

For all statistical analyses, confirm that the following items are present in the figure legend, table legend, main text, or Methods section.

n/a	Confirmed	
☐	☒	The exact sample size (*n*) for each experimental group/condition, given as a discrete number and unit of measurement
☐	☒	A statement on whether measurements were taken from distinct samples or whether the same sample was measured repeatedly
☐	☒	The statistical test(s) used AND whether they are one- or two-sided<br>*Only common tests should be described solely by name; describe more complex techniques in the Methods section.*
☐	☒	A description of all covariates tested
☐	☒	A description of any assumptions or corrections, such as tests of normality and adjustment for multiple comparisons
☐	☒	A full description of the statistical parameters including central tendency (e.g. means) or other basic estimates (e.g. regression coefficient) AND variation (e.g. standard deviation) or associated estimates of uncertainty (e.g. confidence intervals)
☐	☒	For null hypothesis testing, the test statistic (e.g. F, t, r) with confidence intervals, effect sizes, degrees of freedom and P value noted<br>*Give P values as exact values whenever suitable.*
☐	☒	For Bayesian analysis, information on the choice of priors and Markov chain Monte Carlo settings
☐	☒	For hierarchical and complex designs, identification of the appropriate level for tests and full reporting of outcomes
☐	☒	Estimates of effect sizes (e.g. Cohen's *d*, Pearson's *r*), indicating how they were calculated

Our web collection on statistics for biologists contains articles on many of the points above.

Software and code

Policy information about availability of computer code

Data collection	No software was used.
Data analysis	CheckM (v1.1.2); RNAmmer (v1.2); MOTHUR(v1.45.3); GTDB-TK (v1.5.0); fastANI (v1.32); iTOL (v6.5.6); Prokka (v1.14.6); MMseqs (V13.45111); eggNOG-mapper (v2); dbCAN (v2.0); antiSMASH (v6.0); Cytoscape (v3.8.2); Rgi (5.2.0); BLAST (v2.2.26); Fastp (v0.23.1); Bowtie (v2.4.4); Kraken (v 2.1.2); Bracken (v2.6.2)

For manuscripts utilizing custom algorithms or software that are central to the research but not yet described in published literature, software must be made available to editors and reviewers. We strongly encourage code deposition in a community repository (e.g. GitHub). See the Nature Portfolio guidelines for submitting code & software for further information.

Data

Policy information about availability of data

All manuscripts must include a data availability statement. This statement should provide the following information, where applicable:

- Accession codes, unique identifiers, or web links for publicly available datasets
- A description of any restrictions on data availability
- For clinical datasets or third party data, please ensure that the statement adheres to our policy

The data that support the findings of this study have been deposited into CNGB Sequence Archive (CNSA) of China National GeneBank DataBase (CNGBdb) with accession number CNP0003047. All the bacterial strains in COGR have been deposited in China National GeneBank (CNGB), a non-profit, public-service-oriented

organization in China.The datasets used in this study include human oral metagenome sequencing data of a Chinese cohort (a part of 4D-SZ) (https://db.cngb.org/search/project/CNP0000687/, and https://db.cngb.org/search/project/CNP0001221/), and oral metagenomes from healthy control individuals and RA patients (https://www.ebi.ac.uk/ena/browser/view/PRJEB6997).

Research involving human participants, their data, or biological material

Policy information about studies with human participants or human data. See also policy information about sex, gender (identity/presentation), and sexual orientation and race, ethnicity and racism.

Reporting on sex and gender	Sex / gender was not included in our study design and analysis.
Reporting on race, ethnicity, or other socially relevant groupings	Race, ethnicity or other socially relevant groupings was not included in our study design and analysis.
Population characteristics	This study recruited 13 healthy human from China.
Recruitment	We published recruitment information through posters, and participants signed up voluntarily. All participants were recruited in ShenZhen, China. 39 oral samples were collected from 13 healthy volunteers not taking any antibiotics in the last six months prior to sampling or suffering from oral diseases such as aphthous ulcerations and caries. The volunteers were instructed not to brush teeth, drink alcohol, or eat spicy food within 12 hours prior to sample collection.
Ethics oversight	The sample collection was approved by the Institutional Review Board on Bioethics and Biosafety of BGI under the number BGI-IRB 20106-T1.

Note that full information on the approval of the study protocol must also be provided in the manuscript.

Field-specific reporting

Please select the one below that is the best fit for your research. If you are not sure, read the appropriate sections before making your selection.

☒ Life sciences ☐ Behavioural & social sciences ☐ Ecological, evolutionary & environmental sciences

For a reference copy of the document with all sections, see nature.com/documents/nr-reporting-summary-flat.pdf

Life sciences study design

All studies must disclose on these points even when the disclosure is negative.

Sample size	This study collected 39 oral samples including saliva, tongue, and dental plague, isolated ~2,000 bacterial isolates, and generated 1,089 high quality genomes. No sample size calculation was performed, but the sample size and amount of generated sequence data are larger than currently published studies of similar nature.
Data exclusions	Genomes with < 95% completeness or > 5% contamination were excluded. Quality controls used to exclude genomes were based on previously published criteria.
Replication	Genomic data are publicly available, so analyzes can be reproduced using the data and software described in the Methods.
Randomization	Randomization is applied when we select strains from the same cluster for sequencing based on a threshold of 98.7% identity of the 16S rRNA gene sequence.
Blinding	Blinding is not necessary for this study, because is not influenced by the subjective factors of the subjects or researchers.

Reporting for specific materials, systems and methods

We require information from authors about some types of materials, experimental systems and methods used in many studies. Here, indicate whether each material, system or method listed is relevant to your study. If you are not sure if a list item applies to your research, read the appropriate section before selecting a response.

Materials & experimental systems

n/a	Involved in the study
☒	☐ Antibodies
☒	☐ Eukaryotic cell lines
☒	☐ Palaeontology and archaeology
☒	☐ Animals and other organisms
☒	☐ Clinical data
☒	☐ Dual use research of concern
☒	☐ Plants

Methods

n/a	Involved in the study
☒	☐ ChIP-seq
☒	☐ Flow cytometry
☒	☐ MRI-based neuroimaging
